# Ligands for Protein Fibrils of Amyloid-β, α‑Synuclein, and Tau

**DOI:** 10.1021/acs.chemrev.4c00838

**Published:** 2025-05-06

**Authors:** Timothy S. Chisholm, Christopher A. Hunter

**Affiliations:** Yusuf Hamied Department of Chemistry, 2152 University of Cambridge, Cambridge CB2 1EW, U.K.

## Abstract

Amyloid fibrils are characteristic features of many neurodegenerative diseases, including Alzheimer’s disease and Parkinson’s disease. The use of small molecule ligands that bind to amyloid fibrils underpins both fundamental research aiming to better understand the pathology of neurodegenerative disease, and clinical research aiming to develop diagnostic tools for these diseases. To date, a large number of amyloid-binding ligands have been reported in the literature, predominantly targeting protein fibrils composed of amyloid-β (Aβ), tau, and α-synuclein (αSyn) fibrils. Fibrils formed by a particular protein can adopt a range of possible morphologies, but protein fibrils formed *in vivo* possess disease-specific morphologies, highlighting the need for morphology-specific amyloid-binding ligands. This review details the morphologies of Aβ, tau, and αSyn fibril polymorphs that have been reported as a result of structural work and describes a database of amyloid-binding ligands containing 4,288 binding measurements for 2,404 unique compounds targeting Aβ, tau, or αSyn fibrils.

## Graphical abstract

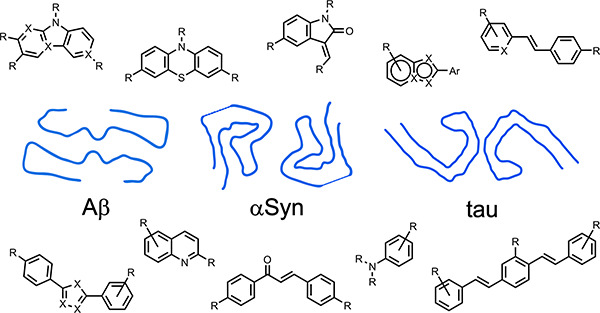

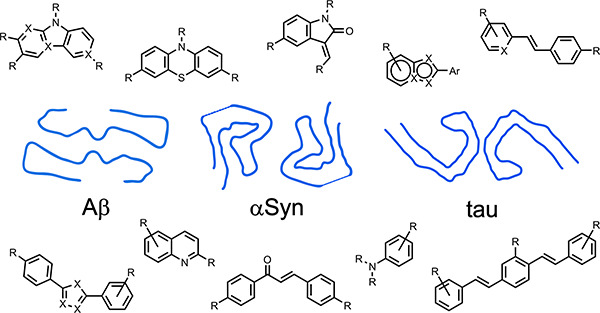

## 1 Introduction

Neurodegenerative diseases are one of the most significant health challenges faced by society.^1–5^ The majority of these diseases can be broadly classified by clinical presentation as dementias, such as Alzheimer’s disease (AD), and movement disorders, such as Parkinson’s disease (PD).^6^ Dementias are embodied by a decline in cognitive functions including memory, judgement, and problem-solving. These symptoms are often accompanied by a deterioration in social behavior and emotional control. Movement disorders can display a range of symptoms including tremors, imbalance, and muscle spasms.^7^

Most neurodegenerative diseases are characterized, and defined, by the accumulation of abnormal protein deposits within the brain.^6^ The formation of these protein aggregates, or amyloids, represents a significant deviation from the normally precise structure and role of proteins. The function of proteins is derived from their three-dimensional folded structure, which can be subdivided into four categories: 1) primary structure, referring to the ordered linear sequence of constituent amino acids, 2) secondary structure, referring to the local three-dimensional conformation of protein subunits, 3) tertiary structure, referring to the overall three-dimensional shape of the protein, and 4) quaternary structure, referring to the assembly of multiple individual polypeptide chains to form a combined functional protein. Given the vast number of conformations available to a polypeptide containing even 100 residues (∼10^30^), the folding process is inevitably error prone and can give rise to misfolded states.^8^ A balance of protein synthesis, protein degradation, and molecular chaperones helps maintain protein homeostasis and avoid aberrant folding.^9^ Despite this combination of cellular processes, pathological protein misfolding and aggregation occurs and is observed in a range of protein misfolding diseases, including many neurodegenerative diseases. Most neurodegenerative diseases involve aggregates formed by three proteins: amyloid-β (Aβ), tau, and α-synuclein (αSyn) (Table 1).

**Table 1:** Common Neurodegenerative Diseases Involving Amyloid-β (Aβ), Tau, and α-Synuclein (αSyn), and the Amyloidogenic Protein Involved

Disease	Protein
Alzheimer’s disease (AD)	Amyloid-β and tau (3R and 4R)
Pick’s disease (PiD)	Tau (3R)
Corticobasal degeneration (CBD)	Tau (4R)
Progressive supranuclear palsy (PSP)	Tau (4R)
Chronic traumatic encephalopathy (CTE)	Tau (3R and 4R)
Parkinson’s disease (PD)	α-synuclein
Multiple system atrophy (MSA)	α-synuclein
Dementia with Lewy bodies (DLB)	α-synuclein

Structural studies of protein fibrils have been historically challenging due to their aggregative, polymorphic, and partially disordered nature, leading to difficulties in obtaining crystalline samples.^10^ Early fibril structures were obtained by X-ray crystallography of steric zippers: short peptides that readily aggregated into β-sheets.^11^ The structures of fibrils comprised of longer peptide segments, and ultimately full-length protein sequences, were later obtained using primarily solid-state nuclear magnetic resonance (ssNMR) spectroscopy and cryogenic electron microscopy (cryo-EM).^10,12^ Cryo-EM has since become the gold-standard technique for the structural determination of protein fibrils and has been used to report a range of structures of Aβ, αSyn and tau aggregates, formed both *in vitro* and *in vivo*.

In general, protein fibrils are composed of one or more β-sheet rich protofilaments that are, in turn, composed of misfolded protein monomers.^13–15^ Recent work in this area has demonstrated that polymorphism is a key feature of protein fibrils. Fibrils have structures determined by the underlying pathology when formed *in vivo*, and the structure of fibrils formed *in vitro* is dependent on the aggregation conditions used. Polymorphism can arise due to variations in the structure of individual protofilaments, the number of protofilaments, and the arrangement of protofilaments.^13,15–20^ Fibril structure is therefore not dictated by the primary sequence of the polypeptide alone, but is a complex function dependent on the local chemical and physical environment. A given set of conditions can lead to a mixture of polymorphs that can show either distinct or continuous variation in structural features.^21^

The ability of protein fibrils to induce aggregation of the native protein monomer can be exploited *in vitro* to accelerate fibrillization and to bias the formation of a subset of fibril polymorphs.^22–24^ Seed amplification assays (SAAs) are a tool based on this premise.^25–27^ In brief, SAAs involve the addition of a large excess of monomeric protein to a solution containing fibrils formed from the same protein. The fibrils seed aggregation of the monomeric protein, and through repeated rounds of agitation then rest, the concentration of fibril in the sample increases. Two main types of SAAs are used: real time quaking-induced conversion assay (RT-QuIC), where agitation is achieved by shaking the solution, and protein misfolding cyclic amplification (PMCA), where agitation is achieved by sonicating the solution.^25^ SAAs have become a powerful tool in the field for detecting minute quantities of fibrils and for increasing the concentration of a fibril sample for study.

Post-translational modifications (PTMs) are also known to influence the aggregation of amyloid proteins.^28–31^ The presence and location of specific PTMs are associated with different diseases, as well as the disease stage.^28,32–35^ PTMs are proposed to influence aggregation by affecting protein-protein and protein-solvent interactions and the conformation of the monomeric or oligomeric protein.^30,36–40^ PTMs do not necessarily need to be present on amino acids within the fibril core to influence fibril structure. αSyn fibrils with different C-terminal truncations show morphological differences despite having similar residues in the amyloid core.^41^ However, *in vivo*, the presence of PTMs will also influence the interaction of amyloid proteins and aggregates with various cellular processes and machinery.^28,29,31,42^ The role of PTMs in ligand binding is also not fully understood, and no cryo-EM structures have been obtained for fibrils where PTMs interact with the ligands.

## 2 Fibril Structures

We have compiled structures of Aβ, αSyn, and tau fibrils available from the RCSB Protein Data Bank.^41,43–136^ Here, we provide a brief overview of these structures, but more detailed analyses and comparisons of many of these fibril structures have been reported.^107,137–139^ The figures and tables below detail the structures of only those fibrils that have been isolated directly from biological material or that have been generated from biological material using SAAs. A summary of the structures of fibrils formed *in vitro* is tabulated in Tables S1–S10 in the Supporting Information.

### 2.1 α-Synuclein

Human αSyn is a 140-residue protein encoded by the SNCA gene that is highly expressed throughout the nervous system.^140^ Aggregates of αSyn in the brain are the defining feature of the synucleinopathies, of which the most common are Parkinson’s disease (PD), dementia with Lewy bodies (DLB), and multiple system atrophy (MSA).^141,142^ PD and DLB are characterized by round, dense neuronal inclusions of aggregated αSyn, termed Lewy bodies (LBs), and abnormal neurites containing aggregated αSyn, termed Lewy neurites.^6,143–149^ In MSA, αSyn aggregates are predominantly found within oligodendroglia and are termed glial cytoplasmic inclusions (GCIs).^150^ αSyn also appears in senile plaques (SPs) in AD where the protein was first discovered.^151^

A large number of structures have been reported for αSyn fibrils formed *in vitro* (Table S1) (refs 41, 44, 52, 55, 57–59, 76, 88, 91, 93,^94^,^97^,^99^,^101^,^102^,^110^,^117^,^120^,^122–125^,^130^,^134–136^, and^152^ ). The morphology of these fibrils is dependent on several factors during the aggregation reaction, including the buffer conditions used, the pH, and whether the solution is agitated. The inclusion of small quantities of preformed αSyn fibrils to seed aggregation was also sometimes used and influenced both the rate of aggregation and the fibril morphology formed. The importance of these factors on fibril structure has been appreciated prior to cryo-EM structures being reported.^153–157^

Structures of fibrils formed from αSyn mutants with the amino acid substitutions A53E, A53T, E46K, H50Q, and G51D have also been solved.^58,59,93,110,120,123^ Fibrils formed from these sequences show key differences in their structure, as well as in their chemical and biological properties. The insertion of MAAAEKT after residue 22, associated with juvenile onset synucleinopathy, also produced a unique morphology.^88^

Other modifications of αSyn have been shown to affect fibril structure. C-terminal truncations led to a similar amyloid core but with some differences in overall morphology, protease resistance, and seeding activity.^41,57^ An N-terminal truncation produced a unique fibril structure composed of two asymmetric protofilaments, whereas N-terminal acetylation did not appear to appreciably influence fibril morphology.^122^ Sequences prepared with phosphorylation at Tyr39 or glycosylation at Ser87 (O-GlcNAc) produced unique morphologies.^91,94^ Both of these PTMs are in β-sheet regions within their respective structures. Fibrils have also been formed in the presence of various additives including heparin, monomeric tau protein, lipids, and calcium and iron ions.^76,101,117,131–133,135,136,152^ These additives had varying effects on both the rate of aggregation and the morphology formed.

Several fibril structures directly isolated from the brain of patients suffering from synucleinopathies have been reported (Table 2, Table S2, Figure 1).^88,112,113^ A common protein fold across PD, Parkinson’s disease dementia (PDD), and DLB was discovered and dubbed the “Lewy fold”.^112^ The structure of this protein fold was distinct from that found in MSA or juvenile onset synucleinopathy (JOS).^88,113^ Two morphologies of fibrils were observed in MSA, each consisting of two different protofilaments, whereas the two morphologies observed in JOS were composed of either a single protofilament or a pair of protofilaments. These reported structures also contained additional bound non-proteinaceous factors.

**Table 2:** Details of αSyn Fibrils Formed *In Vivo* That Have Structures Reported in the PDB^88,112,113^

Study	Pathogenic origin	PDB
Yang, Y. *et al.* 2023	JOS (SNCA mutation)	8BQV, 8BQW
Yang, Y. *et al*. 2022	Lewy fold (PD, PDD, DLB)	8A9L
Schweighauser, M. *et al*. 2020	MSA	6XYO, 6XYP, 6XYQ

**Figure 1:**
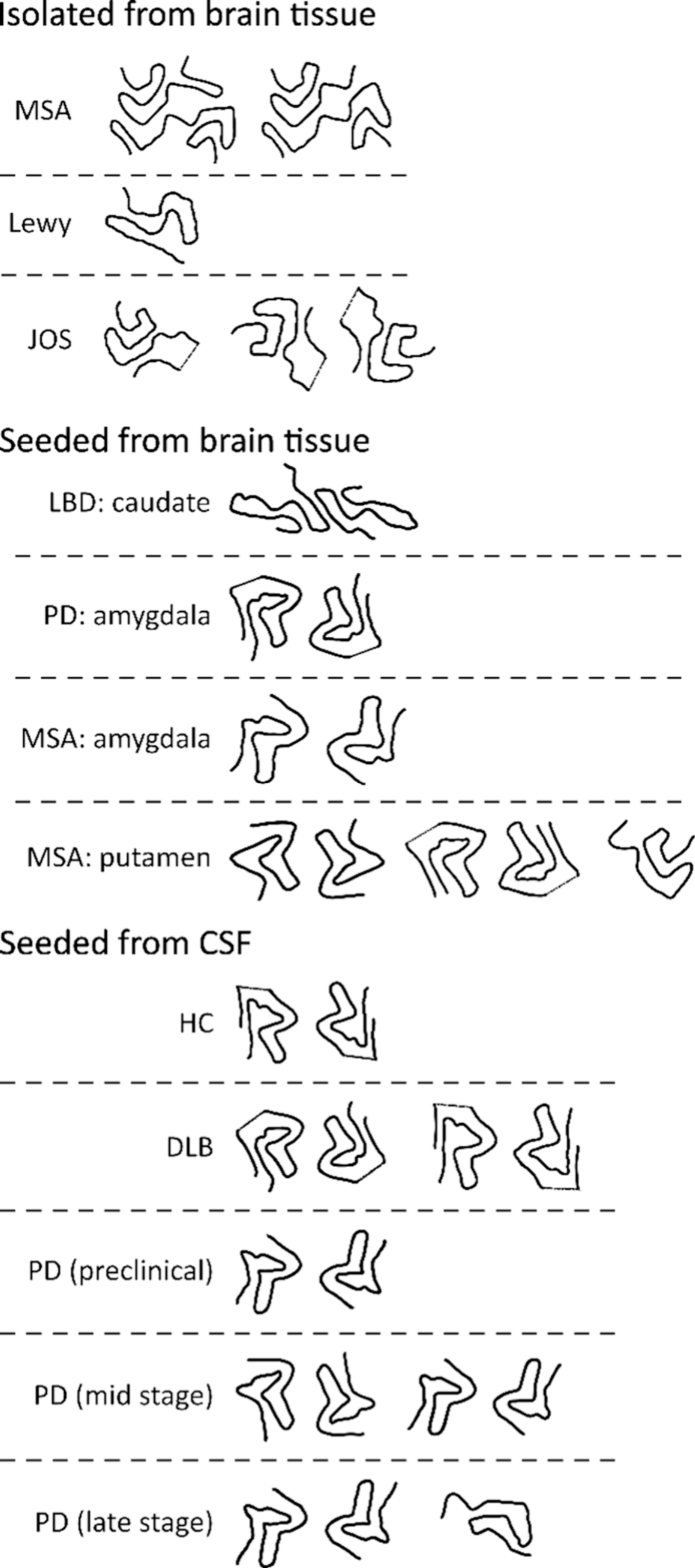
A summary of the PDB structures reported for αSyn fibrils formed *in vivo* or formed using SAAs with biological seeds. Only the path of the peptide backbone is shown. HC: healthy control.

A number of αSyn fibril structures have been reported for samples derived from SAAs seeded with biological samples (Table 3, Table S3, Figure 1).^51,96,100,126,158^ Two studies have amplified fibrils from MSA brain tissue, and while some structural similarities were observed, the global morphology was different.^96,152^ This difference may be a result of the amplification conditions used (real-time quaking-induced conversion (RT-QuIC) vs protein misfolding cyclic amplification (PMCA)) or due to the seeds being obtained from different brain regions (amygdala vs putamen). The morphology of these fibrils also differed from those isolated directly from MSA brain tissue.^113^

**Table 3:** Details of αSyn Fibrils Formed Using SAAs with Biological Seeds That Have Structures Reported in the PDB^a^,^51,96,100,126,158^

Study	Pathogenic origin of seeds	Biosample	Seeding method	Monomer	PDB
Dhavale, D. D. *et al.* 2024	LBD	Brain tissue (caudate)	PMCA	αSyn (1-140)	8FPT
Frieg, B. *et al.* 2022	PD	Brain tissue (amygdala)	PMCA	N-term acetylated αSyn (1-140)	7OZG
Frieg, B. *et al.* 2022	MSA	Brain tissue (amygdala)	PMCA	N-term acetylated αSyn (1-140)	7OZH
Lövestam, S. *et al.* 2021	MSA	Brain tissue (putamen)	RT-QuIC	αSyn (1-140)	7NCA, 7NCG, 7NCH, 7NCI, 7NCJ, 7NCK
Sokratian, A. *et al.* 2022	HC	CSF	RT-QuIC	αSyn (1-140)	8CYR
Sokratian, A. *et al.* 2022	DLB	CSF	RT-QuIC	αSyn (1-140)	8CYS, 8CYT, 8CYV, 8CYW, 8CYX, 8CYY, 8CZ0, 8CZ1, 8CZ2, 8CZ3, 8CZ6
Fan, Y *et al.* 2023	PD (preclinical)	CSF	RT-QuIC	N-term acetylated αSyn (1-140)	8H03, 7V47
Fan, Y *et al.* 2023	PD (mid stage)	CSF	RT-QuIC	N-term acetylated αSyn (1-140)	7XO0, 7XO1, 7XO2, 7XO3
Fan, Y *et al.* 2023	PD (late stage)	CSF	RT-QuIC	N-term acetylated αSyn (1-140)	8HO4, 8HO5, 7V48, 7V49

a HC: Healthy control. CSF: Cerebrospinal fluid.

Fibrils amplified from the brain of both PD and MSA patients shared structural features but are distinct to the structure of fibrils isolated directly from the brain of a PD patient.^126^ However, fibrils amplified from the CSF of PD patients were found to be similar to fibrils amplified from the brain of PD patients, one of the polymorphs that was isolated directly from the brain of MSA patients, and some of the αSyn fibril morphologies that have been formed *in vitro*.^51,113^ The fibrils amplified from CSF were also shown to differ based on disease stage; the major polymorph formed was largely conserved between early, mid, and late stage PD, with some changes at the protofilament interface.^51^ However, different minor polymorphs were found based on the stage of PD.

Fibrils were also amplified from the CSF of ten DLB patients.^100^ Two distinct classes of fibril morphology were identified. Within these classes there were variations in structural features including β-sheet stacking and the inter-β-strand arrangement, giving rise to six types of assemblies overall. The proportion of the two classes present in each amplified sample also varied. These structures differed from another report of fibrils amplified from the brain of a DLB patient.^158^

### 2.2 Tau

Tau protein exists as six major isoforms produced from alternative mRNA splicing and is observed in neurodegenerative diseases such as corticobasal degeneration (CBD), progressive supranuclear palsy (PSP), chronic traumatic encephalopathy (CTE), Pick’s disease (PiD), and AD. Three of the tau isoforms contain three conserved repeats of an approximately 32-residue microtubule binding repeat region (3R tau), and three isoforms have four conserved repeats (4R tau). The type of tau isoform present is both cell-specific and disease-specific. PiD is a 3R tauopathy, PSP and CBD are 4R tauopathies, and both 3R and 4R tau isoforms are observed in CTE and AD. In AD, tau is most commonly found as intracellular aggregates termed neurofibrillary tangles (NFTs).

A large range of structures have been obtained for tau fibrils prepared *in vitro* under different buffer conditions (Table S4).^45,60,71,72,77,81,84,96,106,109,114,127^ Fibril morphology was shown to be dependent on the presence of different divalent and monovalent cations, the presence of anionic co-factors (such as heparin and RNA), salt concentration, and protein length. Pseudophosphorylation, achieved by mutating specific serine and threonine residues to aspartate, also influenced fibril structure despite these mutations taking place in the C-terminal domain outside of the β-sheet core.^47^ Conditions were found that produced fibrils *in vitro* with morphologies matching those observed in AD, CTE, and globular glial tauopathy (GGT).^47^ The ability to access *in vivo* morphologies through *in vitro* aggregation under specific solution conditions will be of significant use to the field.

Multiple structures have been obtained for tau fibrils isolated directly from the brain of patients with several tauopathies (Figure 2, Table 4, Table S5) (refs 43, 64, 66–70, 72, 78, 79, 89, 90, 95,^98^,^105^,^107^,^111^,^115^, and^116^ ). Many of these reported structures contain additional bound non-proteinaceous factors, similar to the reported structures of biological αSyn fibrils.

**Figure 2:**
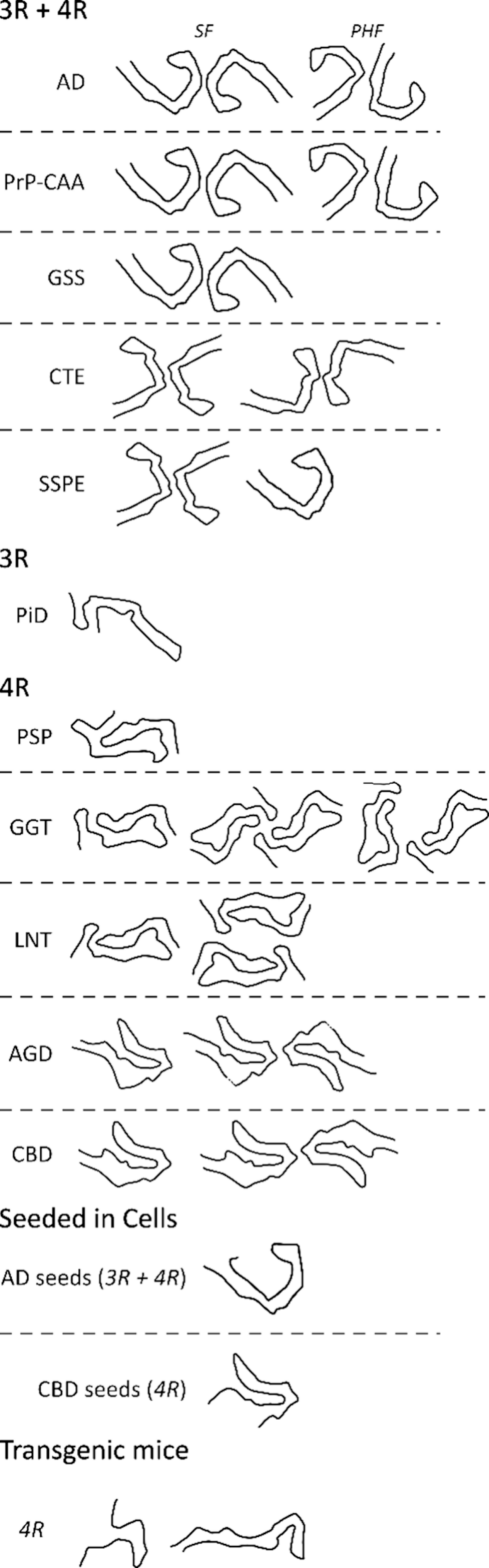
A summary of the PDB structures reported for tau fibrils formed *in vivo*, including in transgenic mouse models and cell models *via* seeding. Only the path of the peptide backbone is shown.

**Table 4:** Details of Tau Fibrils Formed *In Vivo* That Have Structures Reported in the PDB^a^,^43,64,66–70,72,78,79,89,90,95,98,105,107,111,115,116^

Study	Pathogenic origin	Dominant tau isoform	PDB
Stern, A. M. *et al.* 2023	AD (PHF)	3R + 4R	8AZU
Fowler, S. L. *et al.* 2023	AD (PHF)	3R + 4R	8BGV
Seidler, P. M. *et al.* 2022	AD (PHF)	3R + 4R	7UPE
Shi, Y. *et al.* 2021	AD (PHF)	3R + 4R	7NRQ
Arakhamia, T. *et al.* 2020	AD (PHF)	3R + 4R	6VHL
Falcon, B. *et al.* 2018	AD (PHF)	3R + 4R	6HRE
Fitzpatrick, A. W.P. *et al.* 2017	AD (PHF)	3R + 4R	5O3L
Fitzpatrick, A. W.P. *et al.* 2017	AD (PHF)	3R + 4R	5O3O*
Fowler, S. L. *et al.* 2023	AD (PHF, from EV)	3R + 4R	8BGS
Falcon, B. *et al.* 2018	AD (SF)	3R + 4R	6HRF
Shi, Y. *et al.* 2021	AD (SF)	3R + 4R	7NRS, 7NRT
Arakhamia, T. *et al.* 2020	AD (SF)	3R + 4R	6VI3
Fitzpatrick, A. W.P. *et al.* 2017	AD (SF)	3R + 4R	5O3T
Hallinan, G. I. *et al.* 2021	PrP-CAA	3R + 4R	7MKF, 7MKG
Hallinan, G. I. *et al.* 2021	GSS	3R + 4R	7MKH
Lövestam, S. *et al.* 2024	CTE	3R + 4R	8QJJ
Falcon, B. *et al.* 2019	CTE	3R + 4R	6NWP, 6NWQ
Qi, C. *et al.* 2023	SSPE	3R + 4R	8CAQ, 8CAX
Schweighauser, M. *et al.* 2023	PiD	3R	8P34 [Table-fn t4fn2]
Falcon, B. *et al.* 2018	PiD	3R	6GX5
Chang, A. *et al.* 2022	PSP	4R	7U0Z
Shi, Y. *et al.* 2021	PSP	4R	7P65
Shi, Y. *et al.* 2021	GGT	4R	7P66, 7P67, 7P68
Shi, Y. *et al.* 2021	LNT (GPT fold)	4R	7P6A, 7P6B, 7P6C
Shi, Y. *et al.* 2021	AGD	4R	7P6D, 7P6E
Arakhamia, T. *et al.* 2020	CBD	4R	6VH7
Arakhamia, T. *et al.* 2020	CBD	4R	6VHA
Zhang, W. *et al.* 2020	CBD	4R	6TJO, 6TJX

a Pronase-treated.

# ΔK281 mutant.

Several 3R + 4R tauopathies have been studied, most notably AD. The structures of paired helical filaments (PHFs) and straight filaments (SFs) were reported by several studies across multiple patients and are relatively consistent. PHFs isolated from extracellular vesicles had the same structure as those isolated directly from brain homogenates, and the core of PHFs was shown to be pronase resistant.^66,115^

Tau fibrils composed of both 3R and 4R tau are also found in prion protein cerebral amyloid angiopathy (PrP-CAA) and Gerstmann–Sträussler–Scheinker (GSS) disease. Two fibril morphologies were observed in PrP-CAA with structures resembling PHFs and SFs, whereas a single morphology resembling PHFs was found in GSS.^105^ Two different fibril morphologies were observed in CTE, which were structurally identical to fibrils from subacute sclerosing panencephalitis (SSPE).^79^

Several unique fibril morphologies have been observed in 3R and 4R tauopathies. Tau fibrils from PiD had a unique morphology not seen in other tauopathies.^111^ Tau fibrils isolated from individuals with the ΔK281 deletion mutation had an identical morphology to those isolated from sporadic Pick’s disease.^159^ Tau fibrils from PSP, GGT, and limbic-predominant neuronal inclusion body 4R tauopathy (LNT) shared some similarities in the protein fold of individual protofilaments with a three-layered core but had distinct overall morphologies.^107^ The identification of a unique fold, termed the GGT-PSP-Tau (GPT) fold, was the basis for the definition of LNT as a distinct disease.^107^ Two morphologies of tau fibrils were reported in CBD and argyrophilic grain disease (AGD) that were distinct from the other 4R tauopathies.^90,95,107^ The AGD fold also appeared to be present in aging related tau astrogliopathy (ARTAG). These similarities and differences in tau fibril morphology were used as the basis of a proposed structure-based classification of tauopathies to complement clinical diagnosis and neuropathology.^107^

Some structures of tau fibrils have also been reported from other biological systems (Table 5, Table S6). Phosphorylated 4R tau fibrils from a transgenic mouse model had a unique morphology.^69^ Fibrils from SH-SY5Y cells that were seeded with either AD or CBD brain extracts had morphologies resembling the parent fibrils.^68^ Several structures of biological fibrils with bound ligands have also been reported (Table S7).^64,67,70,98^

**Table 5:** Details of Tau Fibrils Formed in Mice or Cells That Have Structures Reported in the PDB^68,69^

Study	Pathogenic origin	Dominant tau isoform	Notes	PDB
Schweighauser, M. *et al.* 2023	Transgenic mouse	4R	P301S	8Q92, 8Q96
Tarutani, A. *et al.* 2023	AD	3R + 4R	Seeded in SH-SY5Y cells	8ORE
Tarutani, A. *et al.* 2023	CBD	4R	Seeded in SH-SY5Y cells	8ORF, 8ORG

### 2.3 Amyloid-β

Amyloid-β (Aβ) peptide is produced via the proteolytic processing of the transmembrane protein, amyloid precursor protein (APP).^160^ Multiple isoforms of Aβ between 37 and 49 residues in length are produced from this processing, with the most common isoforms being Aβ­(1-42) followed by Aβ­(1-40).^161–163^ Aβ is the primary component of amyloid plaques, also referred to as senile plaques (SPs), which are a hallmark of AD neuropathology.^164–166^ Multiple types of these extracellular aggregates have been described, with diffuse plaques and dense core plaques being the most common.^167,168^ Aggregates of Aβ also form in cerebral blood vessels, referred to as cerebral amyloid angiopathy (CAA), which is found in most AD cases to some degree.^168^

A range of structures of Aβ fibrils formed *in vitro* have been obtained (Table S8) (refs 48–50, 53, 65, 73, 75, 82, 83, 85, 86,^92^,^104^,^108^, and^119^ ). The structures of several short Aβ fragments were solved upon crystallization.^50,73,104,108^ Three structures of Aβ­(1-42) fibrils have been reported, including one with a disaccharide-modified tyrosine side chain, and all have unique morphologies.^49,75,92^ Several structures of Aβ­(1-40) fibrils have also been reported, again with different morphologies in every case. These fibrils were formed under various conditions, such as in the presence or absence of seeds^49,53,83,85,86^ or vesicles,^65,119^ and with common mutations associated with AD.^48,83,108^ Fibrils formed from a combination of Aβ­(1-40) and Aβ­(1-42) also have a unique morphology.^82^

Structures of Aβ fibrils formed *in vivo* in both humans and mice have been reported (Figure 3, Table 6, Table 7, Table S9, Table S10).^43,46,54,56,61–63,87,121^ From human tissue, fibrils formed from both Aβ­(1-40) and Aβ­(1-42) have been described, including fibrils formed from individuals with E22Q and E22G mutations, and Aβ­(1-40) fibrils associated with CAA. Broadly, four different types of Aβ­(1-40) fibrils and two different types of Aβ­(1-42) fibrils have been described. One of these Aβ­(1-42) fibril morphologies also appeared to be present in familial AD, ARTAG, PDD, DLB, frontotemporal dementia (FTD), and pathological aging (PA).^61^ An E22G mutation resulted in Aβ­(1-42) fibrils with a morphology found in sporadic AD and Aβ­(1-40) fibrils with a unique morphology.^62^ An E22Q mutation also produced Aβ­(1-40) fibrils with a unique morphology.^46^ Aβ fibrils from transgenic mouse models have been studied, some of which harboured AD-associated mutations within the Aβ sequence.^54,62,121^ Some of these mouse models generated Aβ fibrils with structures resembling those found in patients with AD, whilst novel morphologies were found in others.

**Figure 3:**
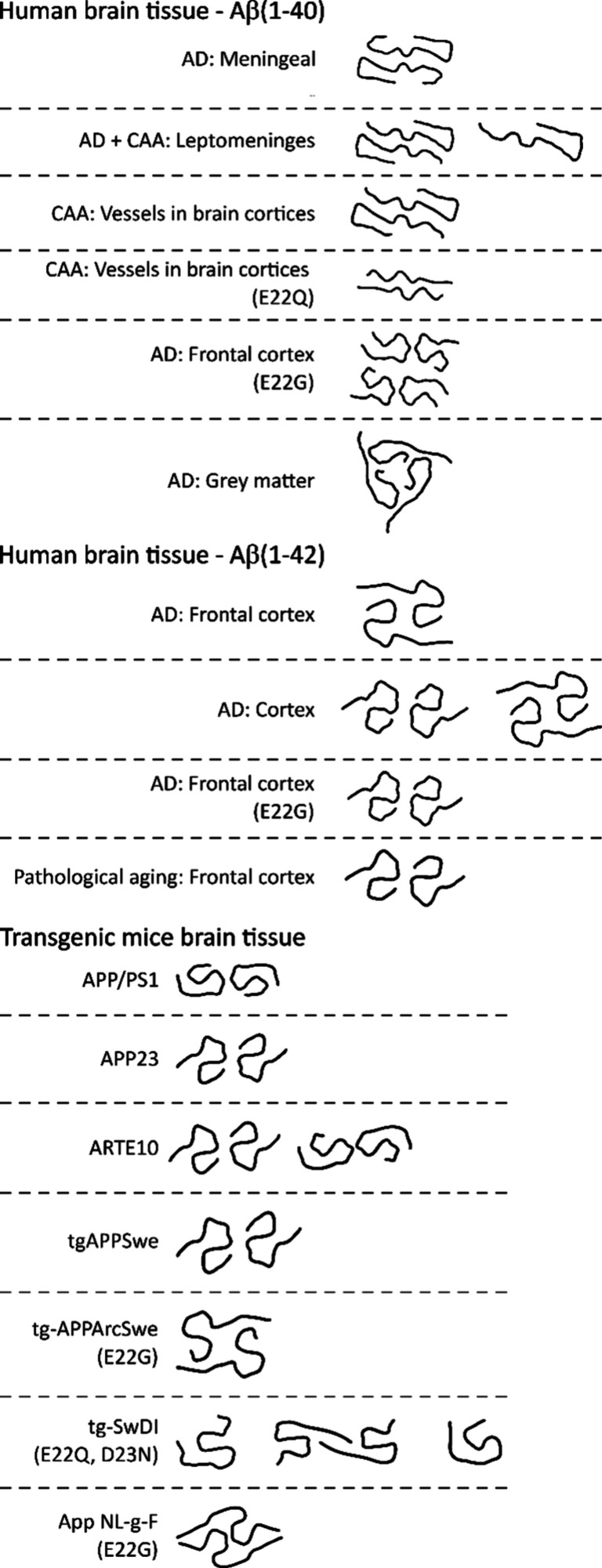
A summary of the PDB structures reported for Aβ fibrils formed *in vivo*, including in transgenic mouse models. Only the path of the peptide backbone is shown.

**Table 6:** Details of Aβ Fibrils Formed In Vivo That Have Structures Reported in the PDB^43,46,56,61–63,87^

Study	Protein	Protein modifications	Pathological origin	PDB
Fu, Z. *et al.* 2024	Aβ(1-40)		CAA (vessels in brain cortices)	8FF2
Fu, Z. *et al.* 2024	Aβ(1-40)	E22Q	CAA (familial Dutch-type) (vessels in brain cortices)	8FF3
Yang, Y. *et al.* 2023	Aβ(1-40)		AD and CAA (leptomeninges)	8QN6, 8QN7
Yang, Y. *et al.* 2023	Aβ(1-42)	E22G	AD (frontal cortex)	8BFZ
Yang, Y. *et al.* 2023	Aβ(1-40)	E22G	AD (frontal cortex)	8BG0
Stern, A. M. *et al.* 2023	Aβ(1-42)		AD (cortex)	8AZS, 8AZT
Yang, Y. *et al.* 2022	Aβ(1-42)		AD (frontal cortex)	7Q4B
Yang, Y. *et al.* 2022	Aβ(1-42)		PA (frontal cortex)	7Q4M
Kollmer, M. *et al.* 2019	Aβ(1-40)		AD (meningeal)	6SHS
Lu, J.-X. *et al.* 2013	Aβ(1-40)		AD (grey matter)	2M4J

**Table 7:** Details of Aβ Fibrils Formed *In Vivo* in Transgenic Mouse Models That Have Structures Reported in the PDB^54,62,121^

Study	Protein modifications	Pathological origin	PDB
Zielinksi, M. et al. 2023		Transgenic mouse (APP23)	8OL2
Zielinksi, M. et al. 2023		Transgenic mouse (APP/PS1)	8OL3
Zielinksi, M. et al. 2023		Transgenic mouse (ARTE10)	8OL5, 8OLO
Zielinksi, M. et al. 2023		Transgenic mouse (tgAPPSwe)	8OL6
Zielinksi, M. et al. 2023	E22G	Transgenic mouse (tg-APPArcSwe)	8OL7
Zielinksi, M. et al. 2023	E22Q, D23N	Transgenic mouse (tg-SwDI)	8OLG, 8OLN, 8OLQ
Leistner, C. et al. 2023	E22G	Transgenic mouse (App NL-G-F)	8BFA, 8BFB
Yang, Y. et al. 2023	E22G	Transgenic mouse (App NL-G-F)	8BG9

## 3 Small-Molecule Binding Sites on Protein Fibrils

Given the importance of protein fibrils in disease, small molecule ligands that bind to these biological assemblies have been extensively investigated as both research tools and potential diagnostics.^137,169–172^ However, the binding modes of small molecule ligands to protein fibrils remain poorly understood. Developing an accurate understanding of the interactions between ligands and fibrils is complicated by the possibility of ligands binding along fibril grooves in a stacked configuration, the presence of multiple binding sites, and the polymorphism of protein fibrils. This uncertainty around the interactions that occur during ligand binding is reflected by a lack of clear SAR during many medicinal chemistry research efforts, and subsequent difficulties in developing selective ligands. Nevertheless, three general approaches have been undertaken to understand fibril binding sites: structural, computational, and ligand-based.

### 3.1 Structural Approaches

A number of structures have been reported for ligands bounds to tau and αSyn fibrils (Figures 4–6). For tau fibrils, structures have been reported for **GTP-1** (also known as **T808** or **AV-680** without deuterium atoms), epigallocatechin gallate (**EGCG**), and **APN-1607** (also known as **PM-PBB3**) bound to PHFs, **APN-1607** bound to SFs, and **flortaucipir** (also known as **AV-1451** or **T807**) bound to fibrils from a patient with CTE (Figures 4 and 6).^64,67,70,98^ **APN-1607** was bound axially, oriented along the length of the PHFs and SFs. In contrast, the other three ligands were oriented parallel to the individual protein monomers but bound in an extended stacked arrangement along the fibril axis. **GTP-1**, **APN-1607**, and **flortaucipir** all bound within the C-shaped cleft of the fibril, whereas **EGCG** bound along the protofilament interface. A similar stacking arrangement was observed for ligands bound to αSyn fibrils, where an extensive study investigated the structural binding mode of nine different ligands (Figures 5 and 6).^102^ In all cases, the bound complex was prepared using micromolar or millimolar concentrations of the ligand. However, these ligands have reported binding affinities that are orders of magnitude stronger (nanomolar to micromolar). The binding modes captured in these structures may therefore not be the high-affinity binding modes that binding assays report on or that would be relevant were these ligands used as PET tracers *in vivo*.

**Figure 4:**
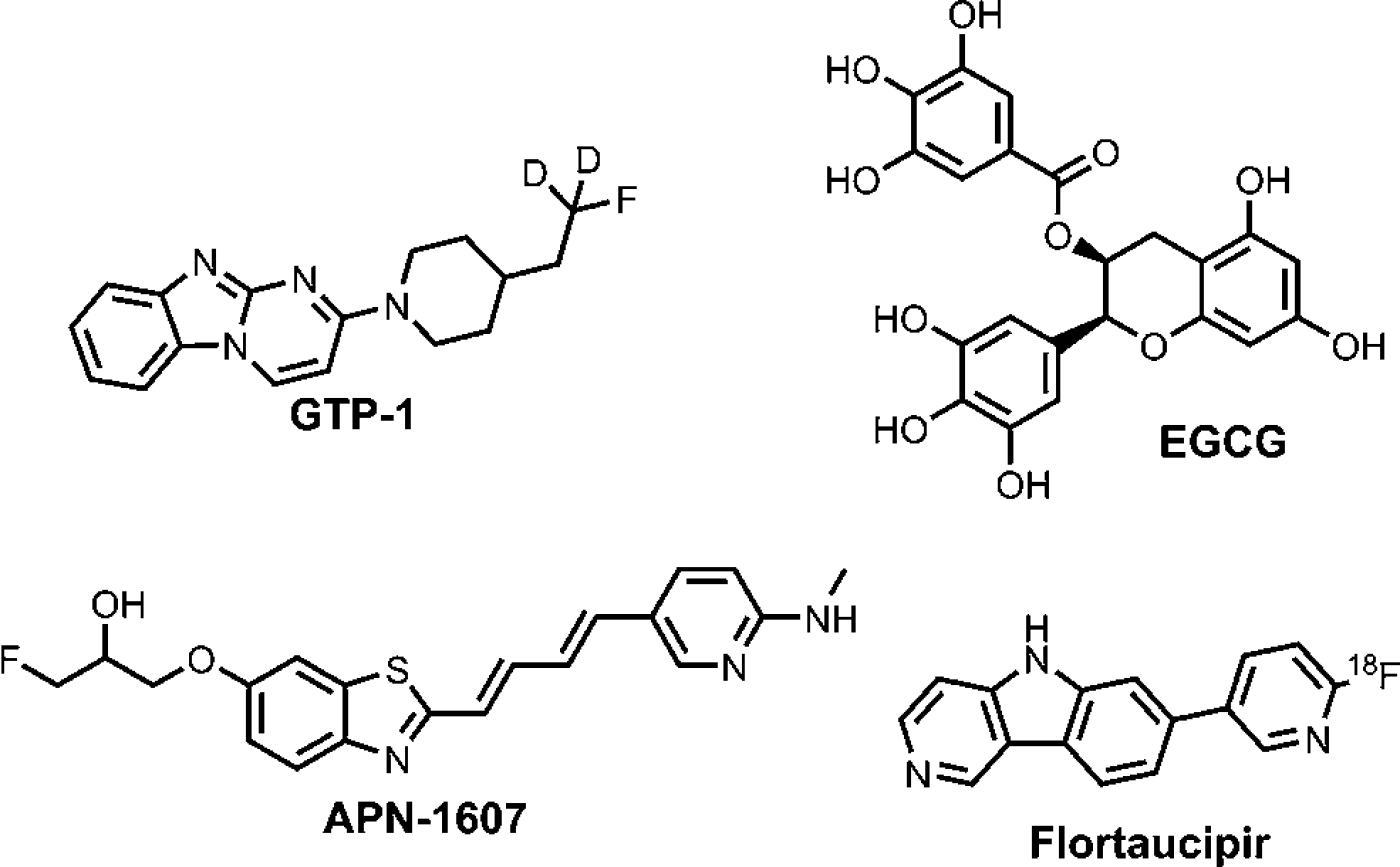
Ligands bound to tau fibrils in reported cryo-EM structures.

**Figure 5:**
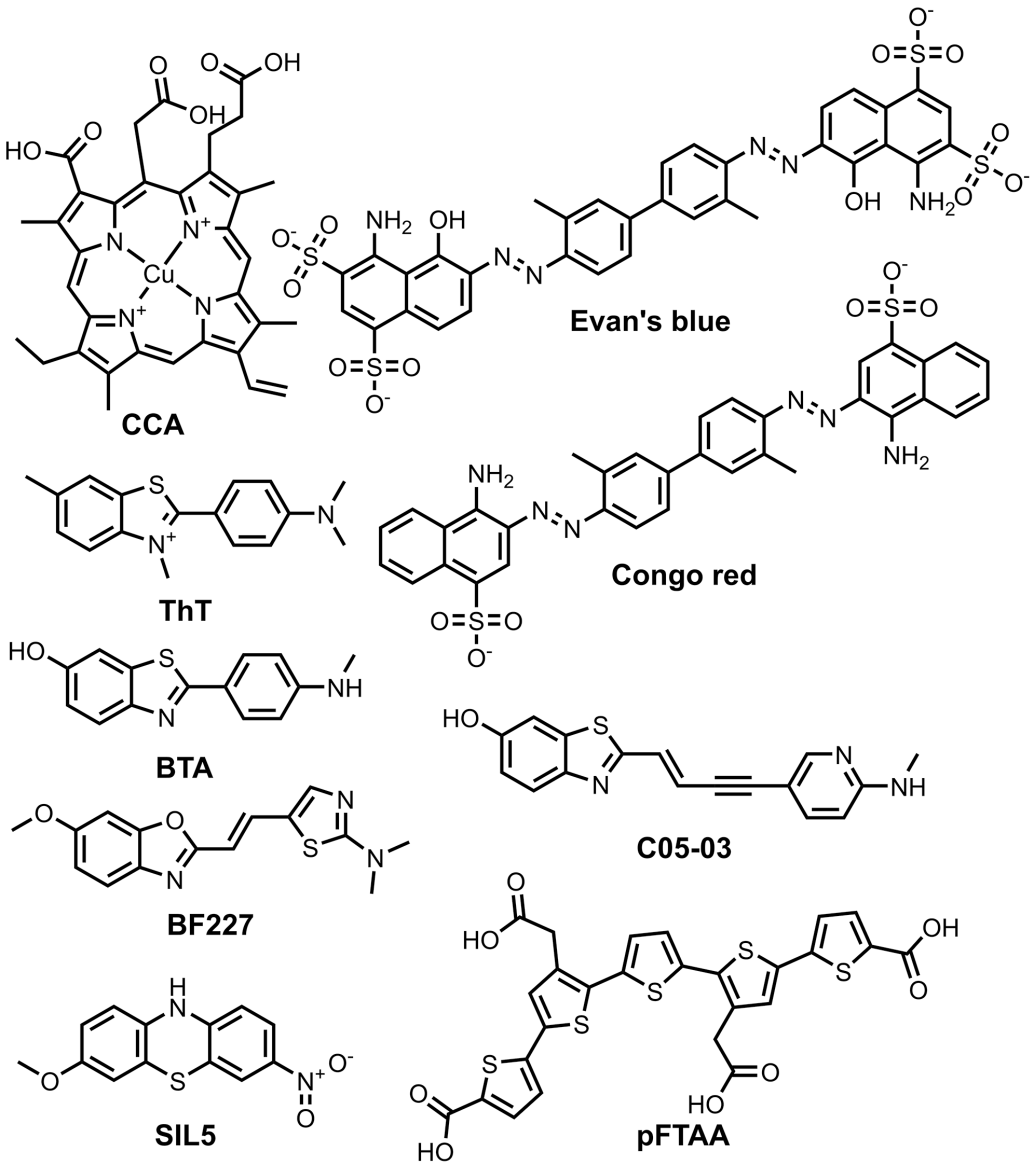
Ligands bound to αSyn fibrils in reported cryo-EM structures.

**Figure 6:**
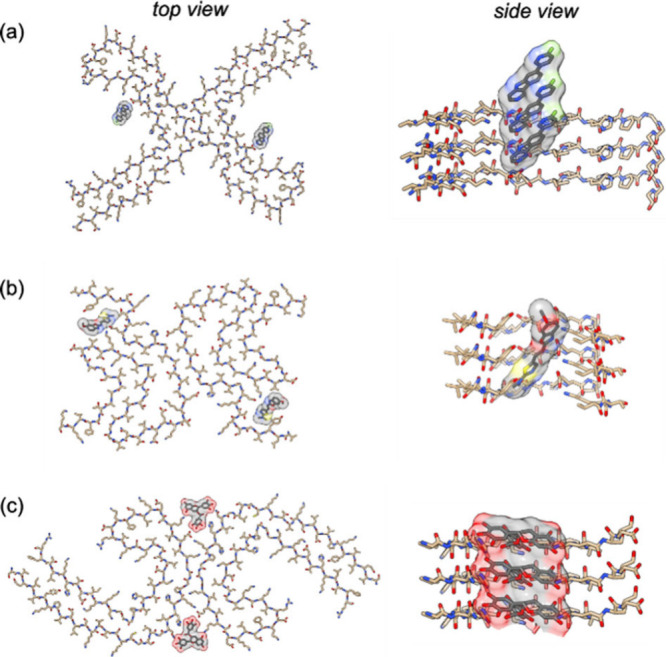
Selected cryo-EM structures of ligands bound to fibrils.^64,70,102^ (a) Tau fibrils from CTE bound to **flortaucipir** (PDB: 8BYN). (b) Tau PHF’s bound to **EGCG** (PDB: 7UPG). (c) αSyn fibrils bound to **BF227** (PDB: 7YNP).

These structures suggest that the binding of small molecules to fibrils can be thought of as binding to continuous grooves rather than to discrete sites. As multiple grooves exist on the surface of fibrils, the observed non-competitive (or partially competitive) binding of some ligands to protein fibrils can still be rationalized.^137^ One result of the groove-like nature of these sites is that ligand–ligand interactions may play an important role in binding. However, it remains unclear if the highest-affinity binding modes involve these interactions. If ligand–ligand interactions prove essential to high-affinity binding, then small changes in the helical twist or rise of the fibril structure could significantly influence binding affinity and impart selectivity between morphologies.

### 3.2 Computational Approaches

A relatively small number of computational studies have been performed that explicitly consider the presence of multiple binding sites when modelling the interactions between small molecule ligands and protein fibrils. The interactions of a structurally diverse set of ligands with both Aβ­(1-40) and Aβ­(1-42) fibrils have been studied, and the calculations were able to reproduce some experimental observations by considering the presence of multiple binding sites.^173,174^ A similar approach has been undertaken for αSyn and tau fibrils, and some of the predicted binding properties were additionally verified by structural work.^67,175–179^ However, these studies invoke the presence of binding sites contained within the interior of the protein fold, and it is unclear how accessible these binding sites are in fibrils.

For αSyn fibrils, a computational approach supported by experimental validation led to a three-site model being proposed.^180^ Docking of a structurally diverse set of ligands was performed, and candidate binding sites were identified. Photoaffinity labelling with one of the ligands confirmed a key residue involved in binding at one site, and competition assays then indicated that different ligands targeted different binding sites. The use of supporting experimental data is necessary if computational approaches aim to precisely identify the binding site on a fibril. These approaches are therefore also reliant on both the quality of the structure used and the ability to reliably produce fibrils with a given structure for experimental validation.

Several studies have also developed quantitative structure–activity relationship (QSAR) models to understand the binding of ligands to different fibrils.^181–186^ This approach has yielded some insights into the properties of binding sites on fibrils, but in general, clear SAR has proved challenging to determine. One key reason for this difficulty may be that large datasets of measurements use binding constants from assays that report on different binding sites. Each binding site may have different or conflicting pharmacophore features, rendering it challenging to create accurate QSAR models. Most successful studies either focus on a small set of structurally similar analogues^182–186^ or use data obtained through competition binding assays that report on a smaller subset of binding sites.^181^

### 3.3 Ligand-Based Approaches

The use of fibril-binding ligands to indirectly report on ligand binding sites is another useful approach, although this method does not provide direct structural information about the binding site. By performing competition binding assays using multiple ligands, it can be determined whether different ligands share the same or different binding sites. Zhuang *et al.* first demonstrated that *bis*styryl benzenes and fused benzoheterocycles each independently bind to Aβ­(1-40) and Aβ­(1-42) fibrils but do not compete with one another, and thus target different binding sites.^187^ An analysis of reported competition binding assays in the literature was performed to create models of binding sites based on what structural classes of ligands bind to each site.^137^ This approach was performed for Aβ­(1-40), Aβ­(1-42), tau, and αSyn fibrils, as well as for AD brain homogenates. Competition assays have also been performed using a diverse set of ligands on several morphologies of αSyn fibrils.^188,189^ This ligand profiling approach is useful for characterizing the types of binding sites present based on what ligands bind to each site and for identifying what binding assays can be used to distinguish different fibril morphologies.

## 4 Database of Fibril-Binding Ligands

A large number of small-molecule ligands for protein fibrils have been reported in the literature. Here, we provide an overview of ligands that have been reported until 2024. The focus is on the binding properties of ligands to protein fibrils, rather than on their *in vivo* or pharmacological properties. We have updated the database we have previously reported^137^ with more recent measurements to now include 4,288 measurements of 2,404 unique compounds from the scientific literature (refs 70, 102, 171, 172, 180, 181, 184, 187–648 ). The criteria for inclusion in the database were 1) a reported chemical structure, 2) a measurement of amyloid binding affinity, i.e. a dissociation (*K_d_*) or inhibition (*K_i_*) constant, or the half maximal inhibitory concentration (IC_50_), and 3) a fibril target related to Aβ, tau, or αSyn. Metal complexes were not included.

Approximately half of the database reports on binding to Aβ fibrils formed *in vitro*, although recent years have seen a shift in focus toward ligands for tau and αSyn fibrils formed *in vitro* (Table 8). There is also a significant amount of data on binding to AD brain homogenates. Protein fibrils are referred to by the name of the protein; e.g., Aβ­(1-40) refers to fibrils of Aβ­(1-40). When monomers or other forms of the protein are discussed, this is stated.

**Table 8:** Distribution of Fibril Type in the Database^a^

Fibril or source^a^	Number of data points
Aβ(1-40)	854
Aβ(1-42)	1278
Tau	331
αSyn	745
AD brain homogenates	792
Other	288

a Aβ­(1-40), Aβ­(1-42), tau, and αSyn all refer to the fibrillar species formed *in vitro*.

The method used to prepare fibrils for binding assays varies between publications, and small changes in conditions can have a significant effect on the polymorph formed. Binding assays reported for fibrils composed of the same protein may therefore report on different fibril polymorphs, potentially leading to differences in measured binding affinity.^188,189,341,400,649^ Caution should therefore be applied when comparing measurements obtained from different studies. In this review, comparisons between dissociation constants are made either (a) using dissociation constants from a single paper or (b) when several datapoints support such a comparison across multiple papers.

All “*K*” values quoted refer to the *K_d_* or *K_i_* reported. Reported IC_50_ values are converted to a *K* value only when the dissociation constant of the competing ligand has been reported in the same publication. Where multiple *K* values are reported for the same ligand-fibril combination, the average value is used in the graphical plots. Most of the reported *K* values in the database are in the nanomolar range, with smaller numbers of picomolar or micromolar values (Figure 7).

**Figure 7:**
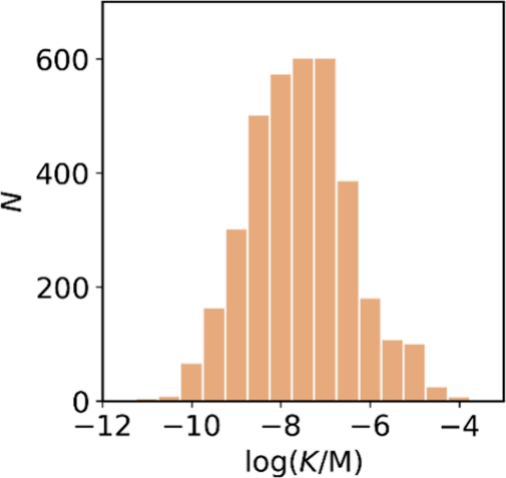
Frequency distribution of log­(K/M) values in the database plotted as the number of ligands (N) with measured log­(K/M) values that fall within successive windows of 0.5 log units.

The ligands exhibit a large degree of structural diversity, but the most common structural cores are illustrated in Figure 8. For each structural class, separate subsections of the review will elaborate on the effects of substituents on amyloid binding properties. Some ligands belong to multiple structural classes. For example, stilbenes functionalized with an amino group are also aminoaryl derivatives, and some ligands contain two different cores conjugated together, e.g. a carbazole and a quinoline. In these cases, the ligand is discussed in one subsection depending, for example, on whether it is part of a structure–activity relationship (SAR) study for that structural class.

**Figure 8:**
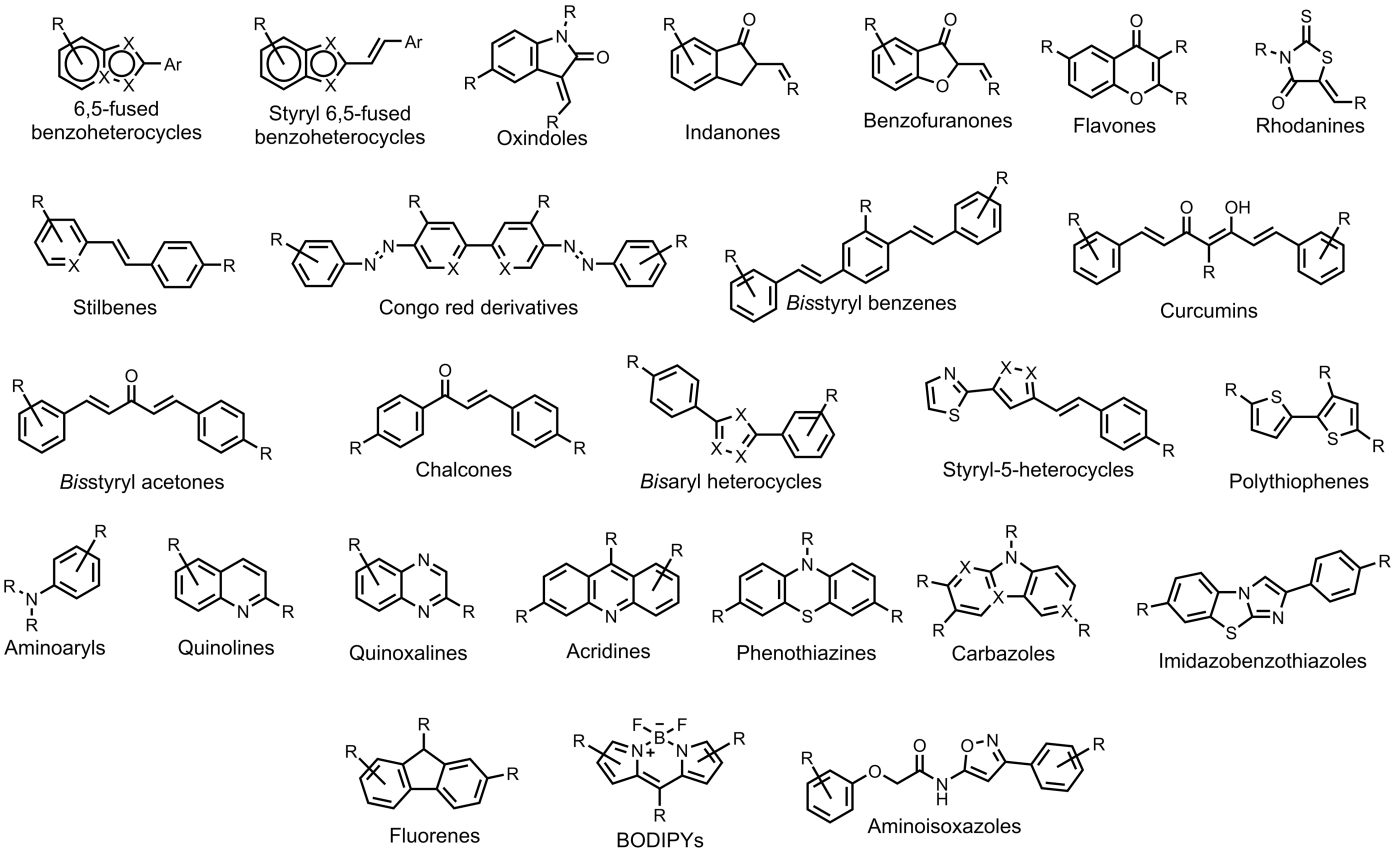
The most common structural classes of amyloid ligands in the database.

## 5 6,5-Fused Benzoheterocycles

6,5-Fused benzoheterocycles represent the largest class of amyloid ligands reported and encompass several different heterocyclic cores as summarized in Figure 9.

**Figure 9:**
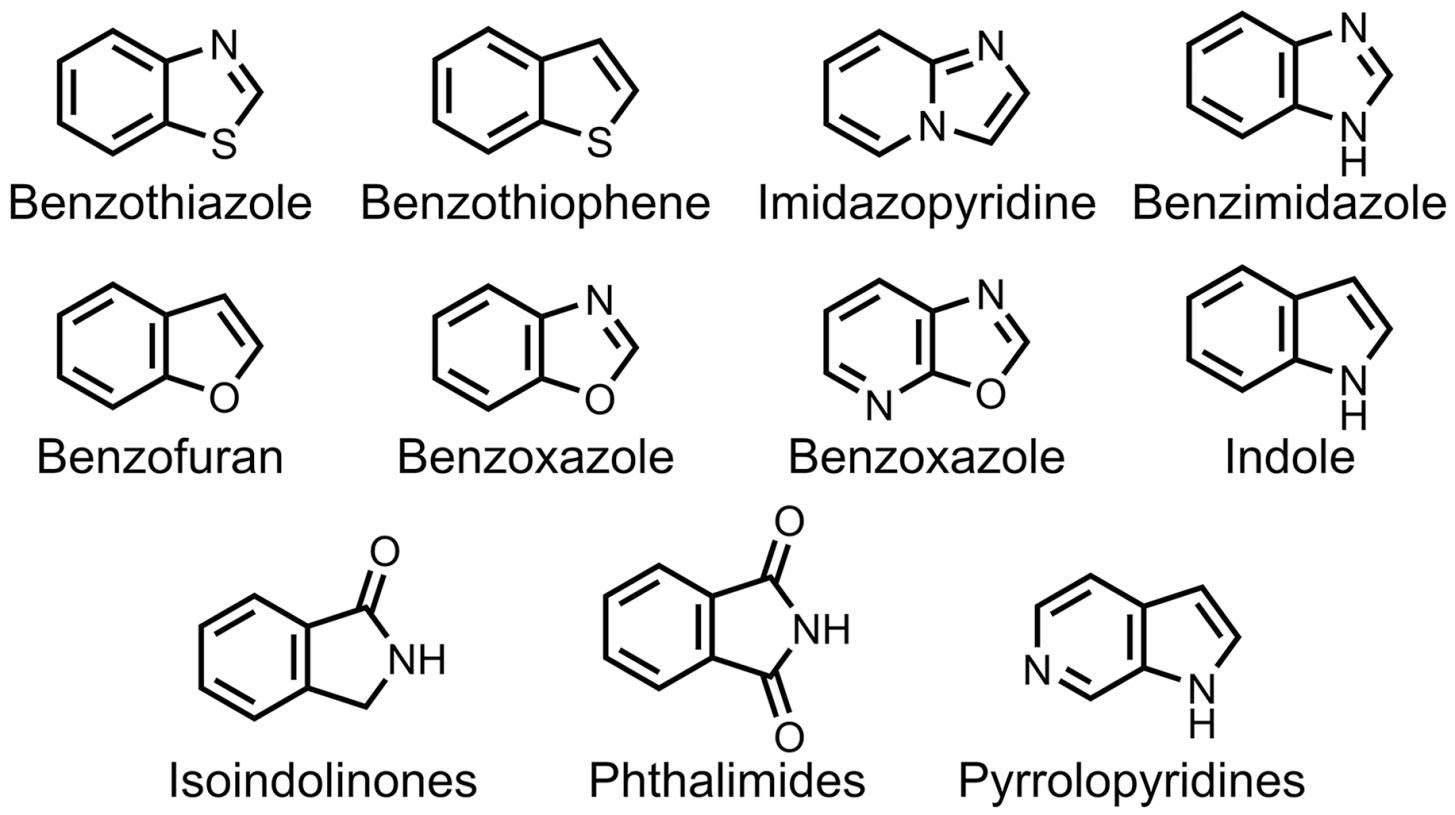
Common 6,5-fused heterocycle structural scaffolds for amyloid ligands.

### 5.1 Charged Fused Benzoheterocycles

Thioflavin T (**ThT**, **1**) is the most common ligand used for detecting protein aggregates (Figure 10).^650–653^ **ThT** undergoes a shift in emission wavelength and increase in fluorescence quantum yield upon binding, making **ThT** well suited for fluorescent imaging. However, **ThT** has poor selectivity and interacts with other hydrophobic biological macromolecules such as albumin and DNA.^654–656^ **ThT** also exhibits variable, micromolar binding affinities for protein fibrils, with reported *K* values of 10 nM to 2,600 nM for binding to Aβ isoforms, and from 15 nM to 12,000 nM for binding to αSyn. One possible source of this variation is that **ThT** forms micelles and self-fluoresces at concentrations above 4–5 μM in aqueous solution, making it challenging to accurately measure micromolar *K* values.^657,658^ Additionally, commercially supplied **ThT** is often impure, with dye content as low as 65%, so purification by recrystallization is required for accurate quantitative measurements.^271^

**Figure 10:**
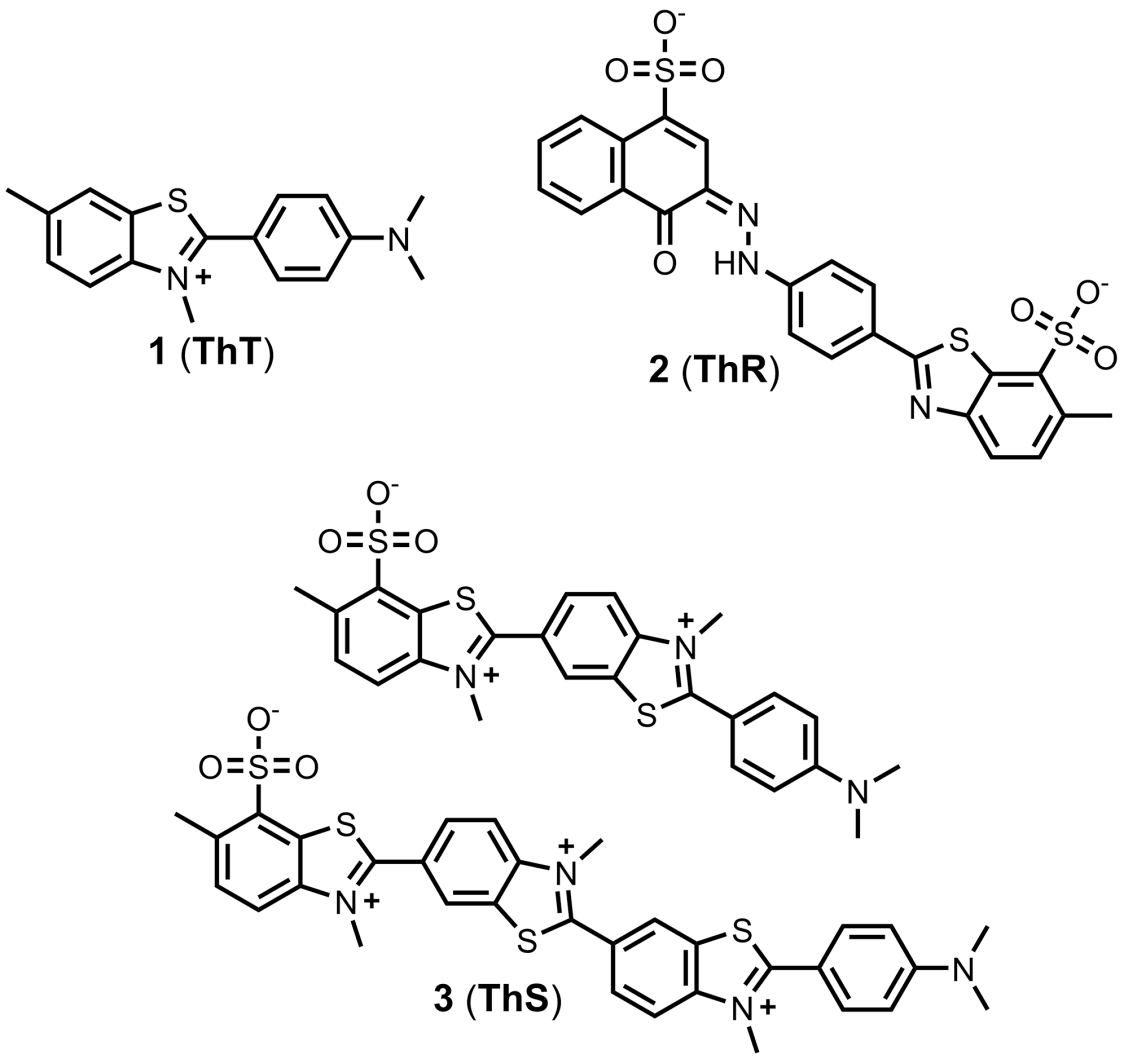
Structures of charged benzothiazole analogues (ThS is a mixture of different compounds, and the two major components are shown).

As shown in Figure 10, Thiazine red (**ThR**, **2**) is another common fluorescent dye and shows selectivity for Aβ­(1-40) (*K =* 49 nM) and tau (*K =* 18 nM) relative to αSyn (*K =* 5,500 nM). A third fluorescent dye, Thioflavin S (**ThS**, **3**), is a mixture of several components that bind both Aβ­(1-42) (*K =* 1,000 nM) and tau (*K =* 260-1,900 nM).^475^ These three ligands are commonly used in fluorescence competition assays to measure the affinities of non-fluorescent compounds.

Several other charged fused benzoheterocycle ligands have been prepared based on a benzothiazole or indole scaffold. The binding affinities are summarized in Figure 11. In these graphs normalized frequency refers to the number of different molecules that bind to a given target with a given affinity, scaled so that the total area under the distribution is equal to 1 for each target. The structures of these charged ligands are shown in Figure 12 and Figure 13. These ligands have primarily been screened with Aβ and generally show lower affinities for αSyn and higher affinities for tau.

**Figure 11:**
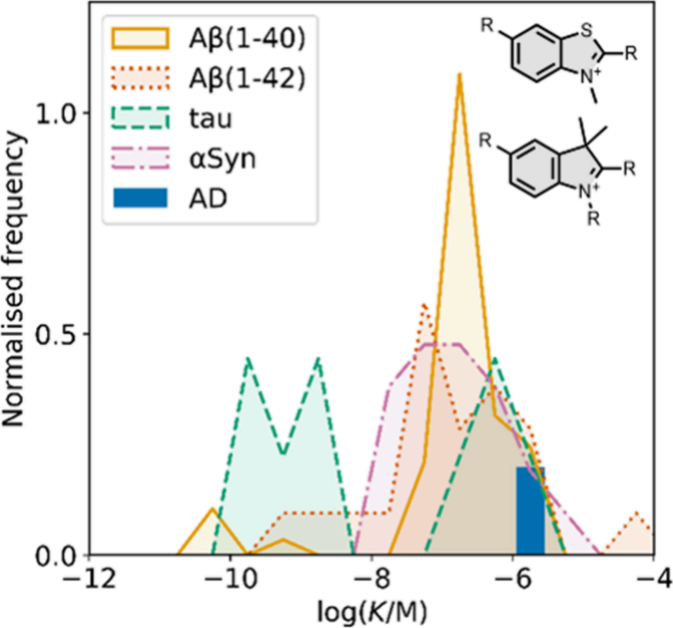
Distribution of binding affinities for the interaction of 87 charged fused benzoheterocycle ligands with amyloids: Aβ­(1-40) (solid yellow line, 57 ligands), Aβ­(1-42) (dotted orange line, 21 ligands), tau (dashed green line, 9 ligands), αSyn (dash-dot purple line, 21 ligands) and AD brain homogenates (blue bar, 1 ligand). Bars are used to represent three or fewer ligands. For ligands where several binding affinities have been reported, the average value was used.

**Figure 12:**
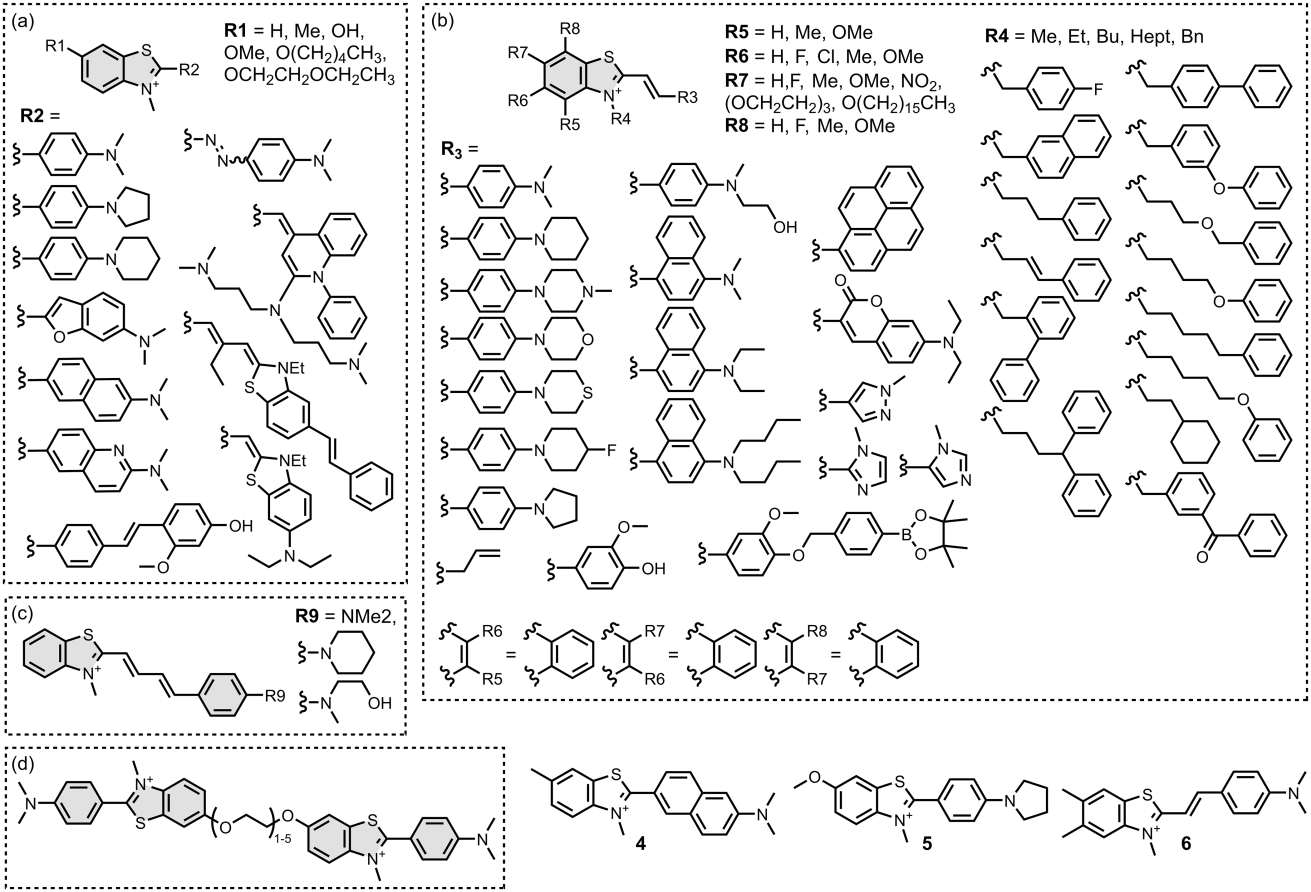
Charged benzothiazole ligands.

**Figure 13:**
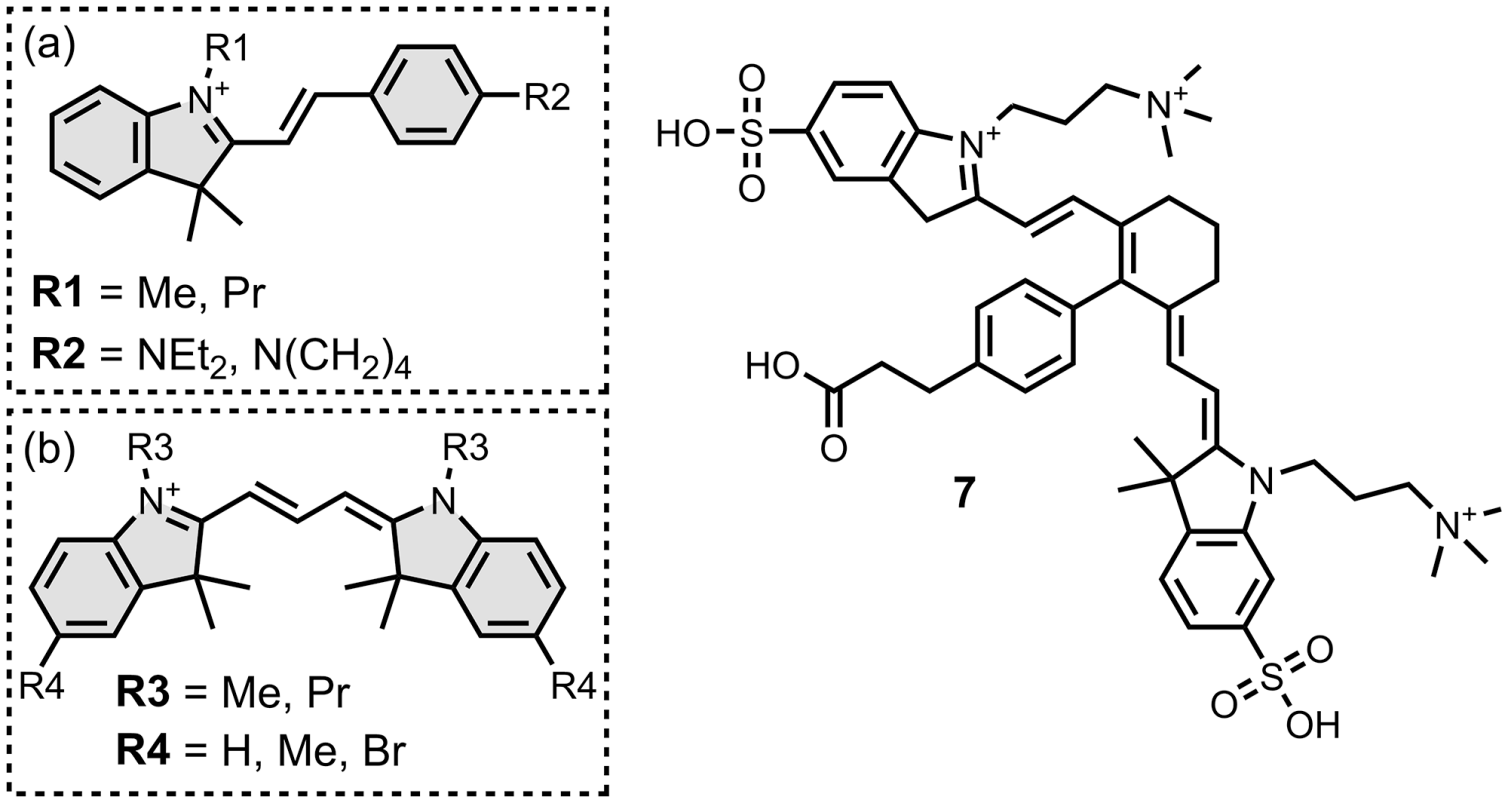
Other charged fused benzoheterocycle ligands.

Modifying a charged benzothiazole core at R1 and R2 in Figure 12a led to a broad range of dissociation constants for Aβ (*K =* 22–2,700 nM), with **4** having the highest affinity (*K* = 22 nM) (Figure 12).^255,385,386,431,601,620^ Linking two benzothiazole rings together with alkene linkers produced high-nanomolar dissociation constants for αSyn (*K* = 560-640 nM), as did substituting the dimethylamino group for a pyrrolidine ring (**5**, *K =* 700 nM).^532,620^

A number of charged styryl derivatives shown in Figure 12b were also prepared and screened with both Aβ and αSyn.^224,226,269,305,370,384,437,632,643^ No clear SAR was observed at R3 for binding to Aβ, except that incorporating 5-membered aromatic heterocycles prevented binding. Most substituents at R4 were tolerated, and extending the benzothiazole core to a three-ring system did not notably affect affinity. At R6 and R7, methylation improved affinity, with **6** showing the strongest binding (*K* = 8 nM). For binding to αSyn, the choice of substituent both at R3 and on the benzene ring (R5–R8) could significantly impact affinity, although no clear SAR was apparent. However, extending the alkene linker to a diene appeared to improve affinity (Figure 12c). ThT dimers connected by PEG linkers of varying lengths were designed as multivalent ligands and demonstrated an improved affinity for Aβ­(1-40) (*K =* 34–120 nM) largely independent of linker length (Figure 12d).^386^

Several charged indole ligands were also prepared, as shown in Figure 13, and all exhibited high affinities for tau (*K =* 0.2–3 nM) and Aβ­(1-40) (*K =* 0.1–0.7 nM).^195,310^ The ligand **7** was studied as an NIR probe for imaging amyloid *in vivo*, but it had only a modest affinity for Aβ (*K =* 94-176 nM).^447^

### 5.2 Benzothiazoles

Neutral benzothiazole ligands are the most studied fused benzoheterocycle derivatives. Figure 14 shows the binding affinities measured for all benzothiazole ligands. Neutral benzothiazole analogues generally exhibited a higher affinity for amyloid fibrils than charged analogues.^626^ Benzothiazole ligands have primarily been screened with Aβ, and many low- and sub-nanomolar ligands have been reported.

**Figure 14:**
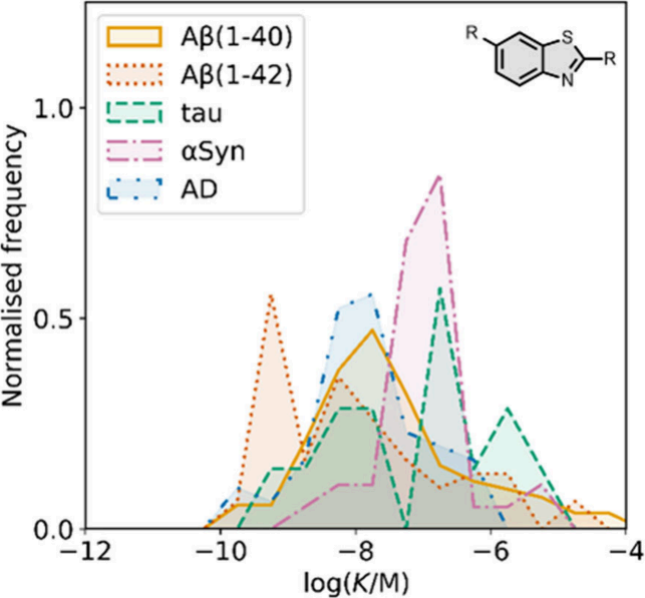
Distribution of binding affinities for the interaction of 229 benzothiazole ligands with amyloids: Aβ­(1-40) (solid yellow line, 106 ligands), Aβ­(1-42) (dotted orange line, 61 ligands), tau (dashed green line, 14 ligands), αSyn (dash-dot purple line, 38 ligands) and AD brain homogenates (dash-dot-dot blue line, 61 ligands). For ligands where several binding affinities have been reported, the average value was used.

Benzothiazole ligands have been prepared with the benzothiazole group both directly attached to a pendant aromatic ring and attached via a linker. Figure 15 summarizes the chemical structures of benzothiazole ligands prepared without a linker. While **ThT** exhibits micromolar dissociation constants for Aβ­(1-40), the neutral analogue **8** has an affinity orders of magnitude higher (*K =* 4 nM). **8** is structurally similar to **9** (commonly known as Pittsburgh-B, **PiB**), which is widely used in imaging studies.^451,641,659–661^ The related ligand **213** (commonly known as **flutemetamol**) is FDA approved for assessing amyloid load by PET imaging.

**Figure 15:**
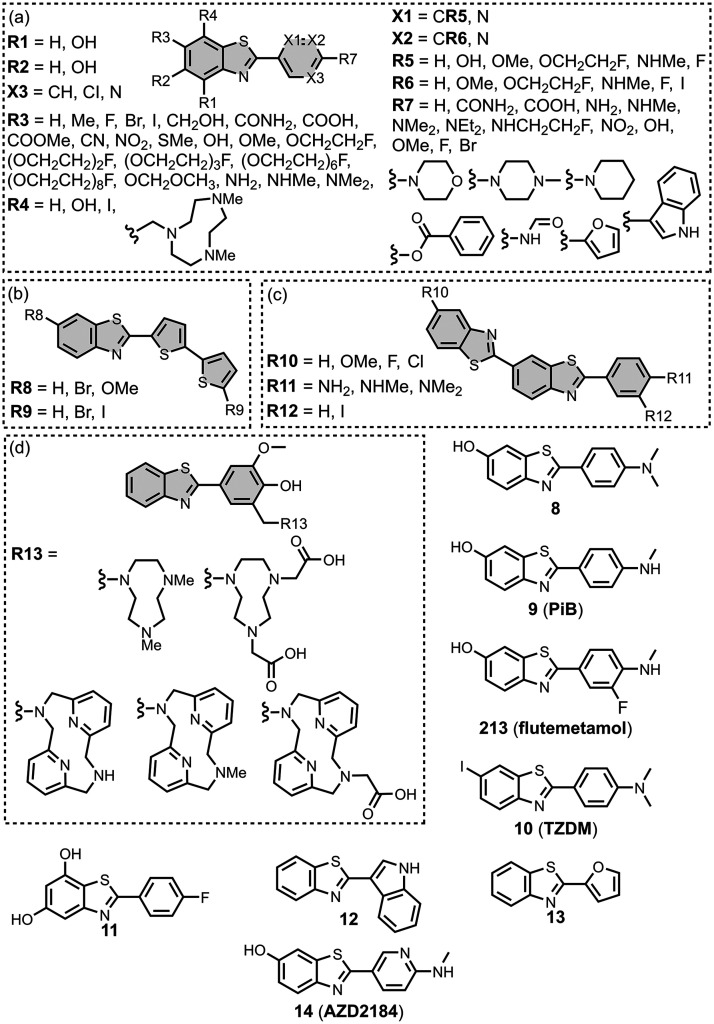
Benzothiazole ligands with a directly conjugated aryl ring.

As shown in Figure 15a, substituents at R3 on the benzothiazole ring have been widely explored and are generally well tolerated (refs 202, 228, 261, 353, 362, 389, 432, 469,^473^,^570^, and^583^ ). Some substituents reduced binding affinity, such as a carboxylate group which abolished binding to AD brain homogenates (*K >* 4,000 nM).^570^ A fluoroethoxy group at R3 gave nanomolar affinities for Aβ­(1-40) and AD brain homogenates and is a useful handle for ^18^F imaging.^375,569,642^ The iodo group at R3 in **10** (commonly known as **TZDM**) was incorporated for imaging, and the ligand appeared to target two sites on Aβ­(1-40) (*K_1_* = 1 nM, *K_2_* = 37 nM).^662^ Dibenzothiazoles were prepared by installing a second benzothiazole ring at R3, which also had nanomolar affinities for AD brain homogenates (Figure 15c).^331^ Hydroxyl substituents have been introduced at other locations on the benzothiazole ring shown in Figure 15a (R1, R2, and R4) and generally did not affect affinities for AD brain homogenates, except for the dihydroxy analogue **11**, which weakened binding (*K =* 800 nM).^570,583^

Amino groups are most commonly incorporated at R7 in Figure 15a. The effect of methylating this amino group depended on the substituent at R3. For compounds with no substituent or a hydroxyl group at R3, a dimethylamino group at R7 increased the binding affinity to Aβ­(1-40) (*K <* 4 nM) by an order of magnitude compared to the *des*methyl analogue (*K =* 37–100 nM).^567^ For a methyl group at R3, dimethylation of an amino group at R7 decreased the binding affinity (*K =* 64–140 nM) by nearly an order of magnitude compared to the *des*methyl analogue (*K =* 10–30 nM).^626,663^ When the R3 substituent was bromine or nitrile, dimethylation of the R7 amino group also slightly reduced the affinity compared to the mono or *des*methyl analogue.^567^ Incorporating nitrogen heterocycles or a methoxy group at R7 did not affect the binding affinity to Aβ­(1-40) or Aβ­(1-42).^187,353,362,469^ Halogen substituents at R7 had a variable effect on the binding affinity, whereas fluoroalkyl groups were well tolerated.^570,581^

Replacement of the pendant benzene ring with pyrimidine, pyridine, or dithiophene ring systems in Figure 15a and Figure 15b was generally well tolerated (*K =* 0.5–30 nM), although some pyrimidines and other heterocycle derivatives (**12**, **13**) had lower affinities (*K =* 50–130 nM).^264,459,565,579,581^ The pyridine derivative **14** (also known as **AZD2184**) appeared to target two different sites on Aβ­(1-40) with nanomolar affinities (8 and 70 nM).^264^ For the benzene derivatives in Figure 15a, an iodo group at R6 generally maintained or improved the binding affinity for Aβ­(1-40).^202,353,564^ Fluoro substituents at the R5 and R6 positions were well tolerated for binding to Aβ­(1-40), but larger fluoroethoxy substituents significantly reduced the binding affinity (>600 nM). Derivatives with azamacrocycles on the pendant aryl ring were also prepared, as shown in Figure 15d, and generally had mid nanomolar dissociation constants (*K* = 11–320 nM).^192,359,436^

Figure 16 summarizes the chemical structures of BTA ligands bearing a linker connecting the pendant aryl ring. The azo linkers shown in Figure 16a and Figure 16b generally led to nanomolar affinities for Aβ and tau (*K =* 0.5–20 nM) and were selective over binding to αSyn.^195,310,333,427,437^ In Figure 16a, an FPEG_3_ chain at R1 reduced the affinity for both fibril types (*K =* 45 nM) compared to a methoxy or iodo substituent (*K =* 0.5–7 nM).^209^ Increasing *N*-methylation at R2 improved both the affinity and selectivity for tau over Aβ­(1-42).^484^ Several ligands with imine linkers, shown in Figure 16c, were screened with AD brain homogenates (*K =* 4–510 nM), with a dimethylamino group at R5 being preferred.^268^

**Figure 16:**
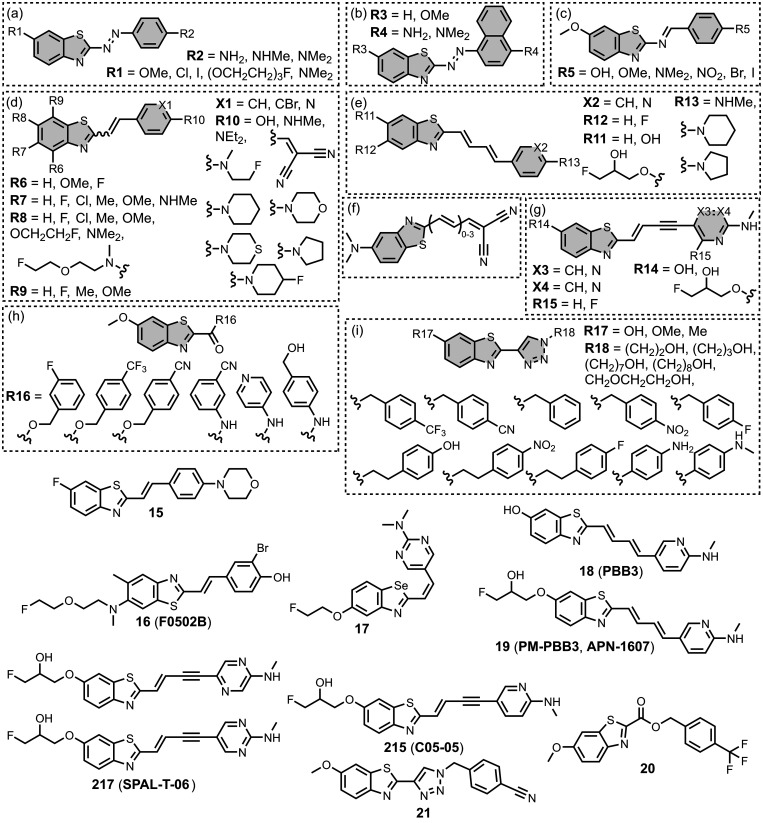
Benzothiazole ligands connected to an aryl ring via a linker.

The styrene derivates shown in Figure 16d, typically with *trans* alkene linkers, exhibited a range of affinities for different fibril types.^224,286,427,437,452,499,587,664^ A SAR study for binding to αSyn showed that substituents at R6 and R9 were generally disfavored, but at R7 and R8 they yielded similar affinities.^224^ The group at R10 significantly affected affinity, with **15** showing the strongest binding (*K* = 10 nM). The derivative **16** (commonly known as **F0502B**) was also prepared and showed strong binding to αSyn in PD and DLB brain tissue (*K* = 4–10 nM).^664^ Another ligand with a benzoselenazole core and a *cis* linker, **17**, showed excellent nanomolar affinities for Aβ­(1-42) (*K =* 1.6 nM) and AD brain homogenates (*K =* 1.7 nM).^379^

Extending the alkene linker, as shown in Figure 16e, also produced high affinity ligands, most notably **18** (commonly known as **PBB3**) and **19** (commonly known as **PM-PBB3** or **APN-1607**), which bind to AD, DLB, and PSP brain homogenates with nanomolar affinities (*K =* 1–6 nM).^102,224,341,391,395,400^ This class of ligands also generally showed selective binding to αSyn over Aβ for fibrils formed *in vitro*.^224^

Figure 16f shows ligands with a conjugated malonitrile group, which afforded mid nanomolar affinities for Aβ­(1-42) (*K =* 40–150 nM) and αSyn (*K =* 48–353 nM) and NIR emissions (λ_em_ = 671–770 nm).^463^ As shown in Figure 16g, an alkyne linker was also incorporated in several derivatives.^102,391^ These molecules were investigated as αSyn ligands, with several showing high nanomolar affinities to αSyn aggregates in MSA, DLB, and PD, most notably **215** (commonly known as **C05-05**) and **217** (commonly known as **SPAL-T-0**6).^647,648,665^

Figure 16h and Figure 16i summarize the ligands with triazole, ester, and amide linkers. These ligands partially displaced **PBB3** from AD brain homogenates, which binds to multiple sites on tau.^593^ Amide linkages in Figure 16h produced lower-affinity ligands (*K =* 220–760 nM), whereas the ester derivative **20** exhibited strong binding (*K =* 2.4 nM). In Figure 16i at R17, both hydroxy and methoxy groups were well tolerated and led to nanomolar affinities. When appending an aromatic ring at R18, a one or two carbon spacer improved affinity, and the ligand **21** showed the strongest binding (*K =* 0.5 nM). Otherwise, hydroxyalkyl chains up to heptanol produced modest nanomolar affinities (*K =* 29-60 nM). Notably the triazole derivatives in Figure 16i labelled Aβ plaques but not NFTs in AD brain sections, whereas both the amide and ester derivatives in Figure 16h labelled NFTs.

Figure 17 shows the dimeric benzothiazole ligands that have been prepared. Dimers joined via the thiazole ring were prepared with a similar length to *bis*styryl benzene ligands, which are discussed later (Figure 17a). These dimers bound to Aβ­(1-42) with an affinity (0.5–1.4 nM) similar to the affinity of **PiB** (*K =* 0.8 nM), which was measured in the same publication.^366^ Sanna *et al.* prepared dimers with BTA head groups joined on the benzene ring with different length PEG spacers (Figure 17b).^434^ Binding affinity was dependent on the length of the PEG linker. A PEG_9_ linker afforded the highest affinity ligand for αSyn (*K =* 2.7 nM) whereas a PEG_15_ linker afforded the strongest binding for Aβ­(1-42) (*K =* 7 nM) and tau (*K =* 110 nM). Selectivity for different aggregates could therefore be achieved by matching the length of the linker to the spacing of two different binding sites on the aggregate. Dimers prepared with a macrocyclic linker were also prepared (Figure 17c), although these had relatively poor affinities for Aβ (*K* = 290–1,300 nM).^343,359^

**Figure 17:**
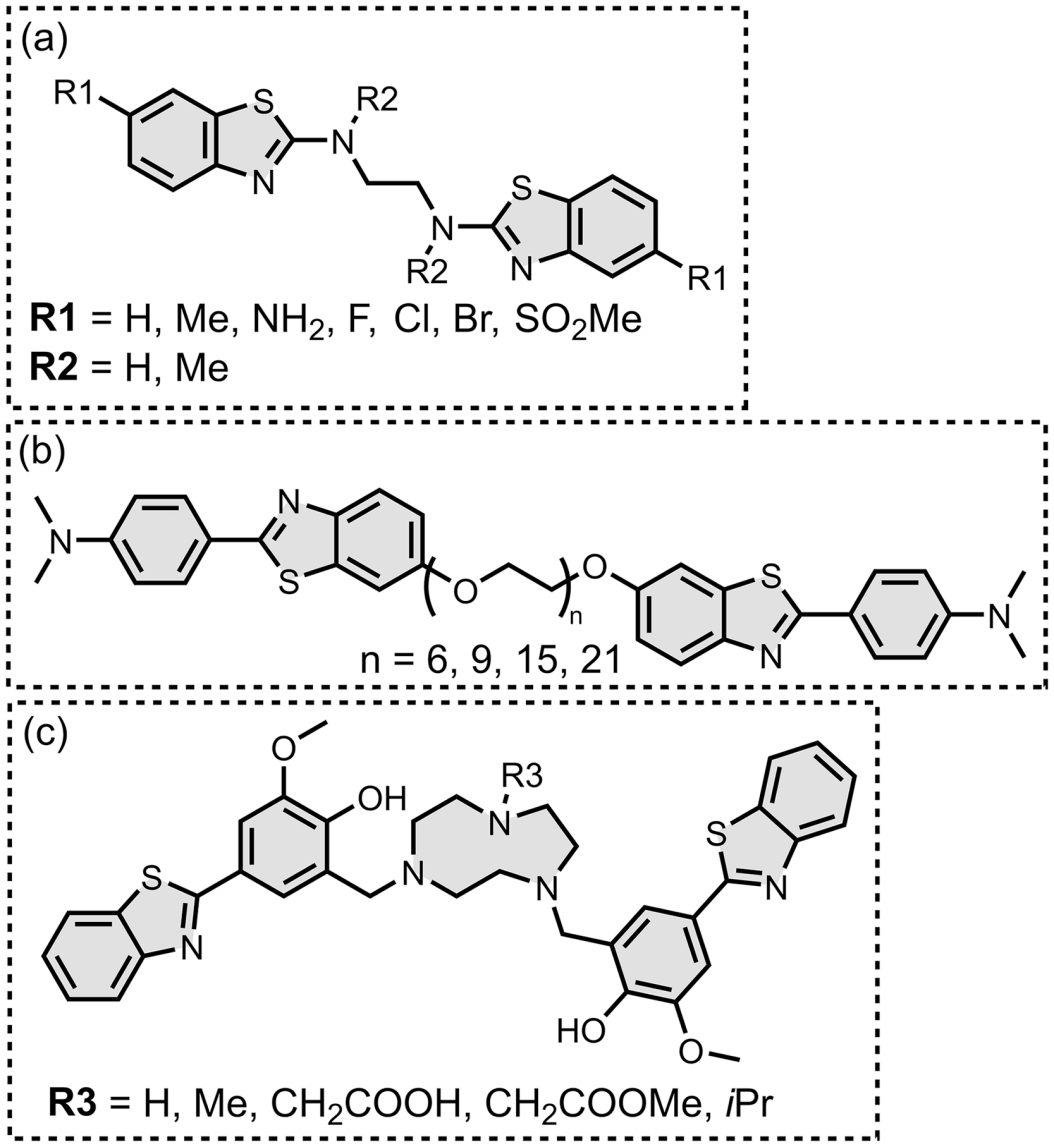
Dimeric benzothiazole ligands.

### 5.3 Benzothiophenes

Several benzothiophene derivatives have been prepared. The binding affinities are summarized in Figure 18, and the chemical structures are summarized in Figure 19.^574^ All compounds had binding affinities in the range 0.3–3 nM for Aβ­(1-40) and Aβ­(1-42).

**Figure 18:**
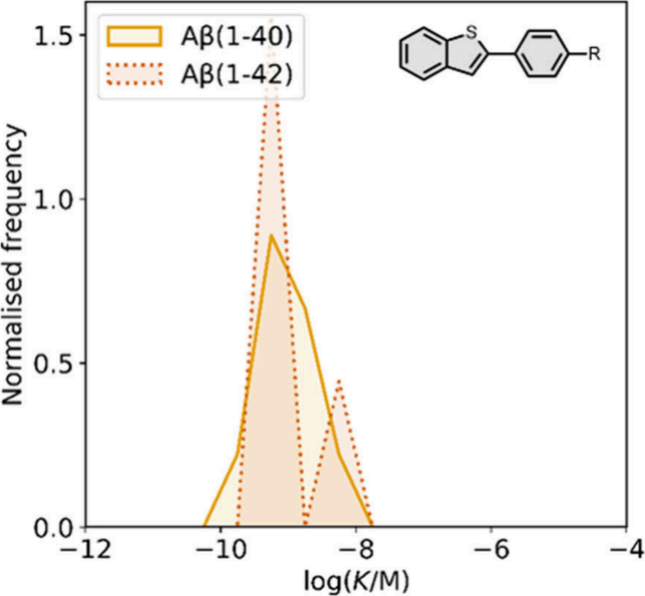
Distribution of binding affinities for the interaction of 9 benzothiophene ligands with amyloids: Aβ­(1-40) (solid yellow line, 9 ligands) and Aβ­(1-42) (dotted orange line, 9 ligands).

**Figure 19:**
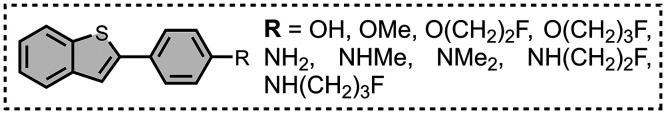
Benzothiophene ligands.

### 5.4 Imidazopyridines

The binding affinities of the imidazopyridine derivatives prepared are summarized in Figure 20. Most binding studies for imidazopyridines have been carried out with Aβ­(1-40) and AD brain homogenates showing mid- to low-nanomolar affinities. Figure 21 provides an overview of imidazopyridine analogues that have been investigated. An imidazopyridine scaffold was first reported for the iodinated ligand **22** (commonly known as **IMPY**).^398^ Radioiodination of iodine-functionalised ligands like **IMPY** allows for SPECT imaging, typically using ^123^I or ^125^I. **IMPY** showed strong labelling in AD gray matter homogenates and was selective over homogenates from AD white matter and healthy controls.^274^ Binding studies showed that **IMPY** had nanomolar dissociation constants for Aβ isoforms and AD brain homogenates (*K =* 1–30 nM), and a micromolar dissociation constant for tau.

**Figure 20:**
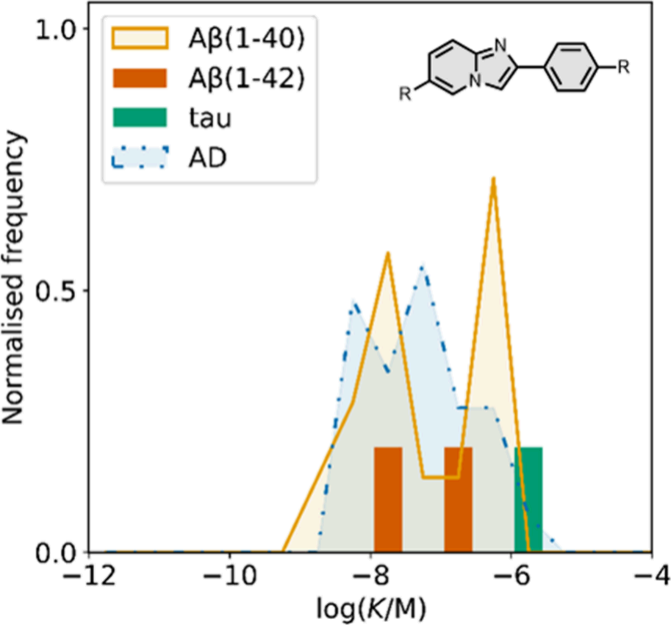
Distribution of binding affinities for the interaction of 38 imidazopyridine ligands with amyloids: Aβ­(1-40) (solid yellow line, 14 ligands), Aβ­(1-42) (orange bars, 2 ligands), tau (green bar, 1 ligands), and AD brain homogenates (dash-dot-dot blue line, 29 ligands). Bars are used to represent three or fewer ligands. For ligands where several binding affinities have been reported, the average value was used.

**Figure 21:**
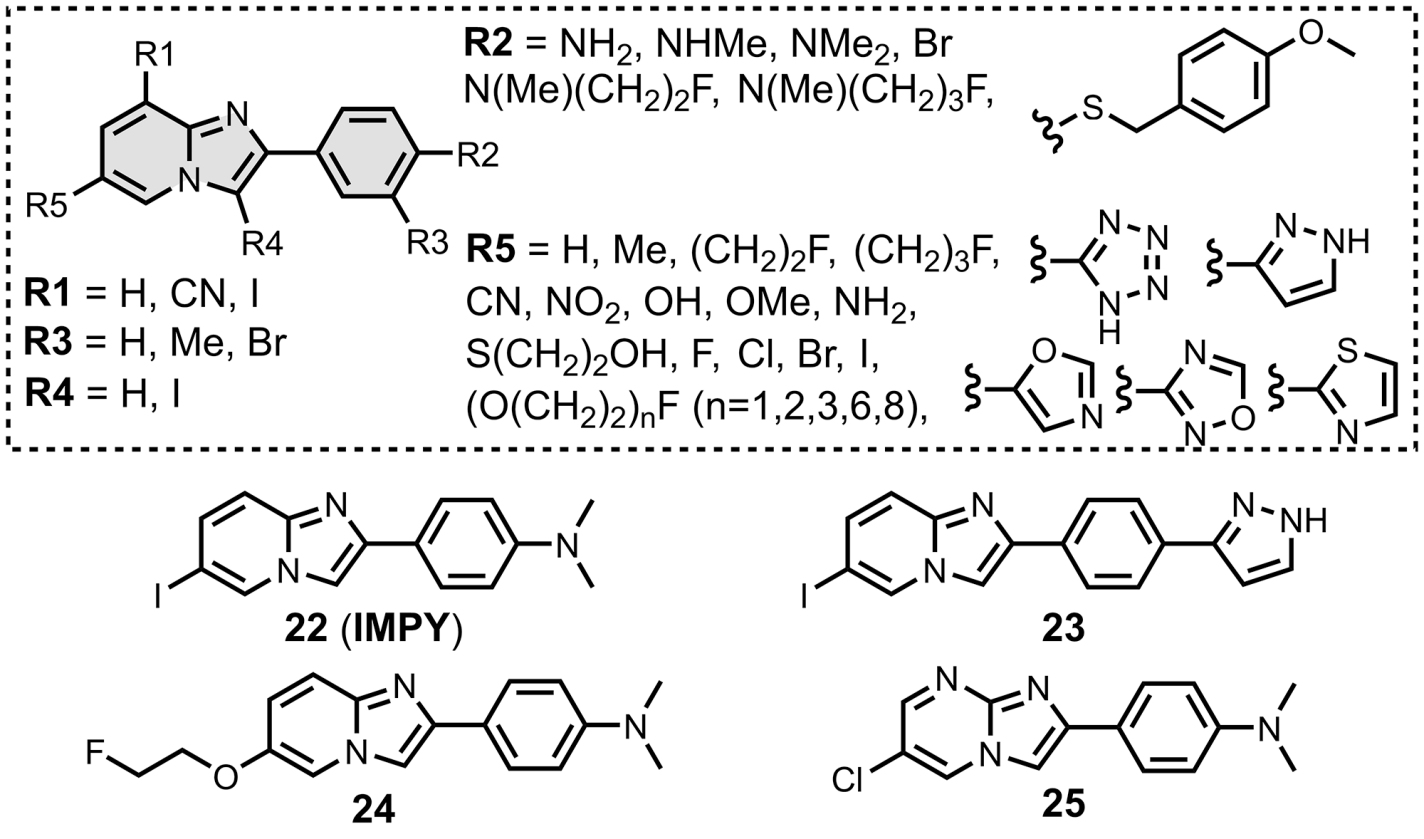
Imidazopyridine ligands.

Amines were usually incorporated at R2 in Figure 21 with dimethylation typically improving binding affinity over the monomethylated or *des*methylated analogue by up to an order of magnitude.^469,537^ Aromatic heterocycles at R2 maintained a high affinity (1–10 nM) for Aβ­(1-40), whereas a bromine reduced affinity (340 nM).^416,537^ One analogue, **23**, was shown to access two sites in AD brain homogenates (*K*_1_ = 4 nM, *K*_2_ = 300 nM).^416^ Amines at R2 derivatized with fluoroethyl and fluoropropyl groups also demonstrated good affinity for AD brain homogenates (*K =* 30–40 nM) and facilitated radiolabelling.^584^

Substituents at R5 in Figure 21 were also commonly explored, affecting affinities for both Aβ­(1-40) and AD brain homogenates. Halide, nitro, cyano, and mercaptoethanol substituents all maintained good affinities for AD brain homogenates (*K =* 5–15 nM) whereas a hydroxyl group reduced affinity by an order of magnitude (*K =* 180 nM).^591^ Fluoro-PEG chains at R5 were also well tolerated, although affinity decreased with the length of the chain from 16 nM for PEG_1_ (**24**) to 390 nM for PEG_8_.^375^

Substituents at other positions in Figure 21, such as R1, R3, and R4, generally lowered affinity.^537,591^ An imidazopyrazine scaffold was also investigated in **25** (*K =* 90 nM), which had a similar binding affinity to the imidazopyridine analogue (*K =* 20 nM).^591^

### 5.5 Benzimidazoles

Benzimidazole derivatives have been reported and show higher affinities for tau than αSyn, with the range of measured affinities summarized in Figure 22. Figure 23 summarizes the structures of reported benzimidazole derivatives. Benzimidazole derivatives were first identified as possible amyloid ligands when screening the FDA approved drugs astemizole (**26**) and lansoprazole (**27**). These ligands exhibited nanomolar dissociation constants for tau (*K =* 2–3 nM) and Aβ­(1-42) (*K =* 2–11 nM) but had weaker affinities for AD brain homogenates (*K >* 4 μM).^524^ *N*-Methylation of **27** to **28** improved the selectivity for tau (*K =* 0.7 nM) over Aβ­(1-42) (*K =* 8.2 nM) *in vitro* and in AD brain sections, but **28** had poor brain uptake.^358,383^

**Figure 22:**
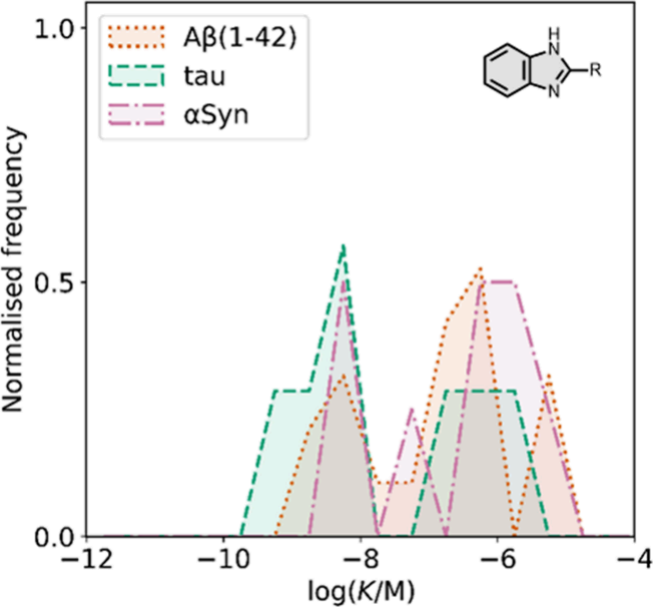
Distribution of binding affinities for the interaction of 21 benzimidazole ligands with amyloids: Aβ­(1-42) (dotted orange line, 19 ligands), tau (dashed green line, 7 ligands), and αSyn (dash-dot purple line, 8 ligands). For ligands where several binding affinities have been reported, the average value was used.

**Figure 23:**
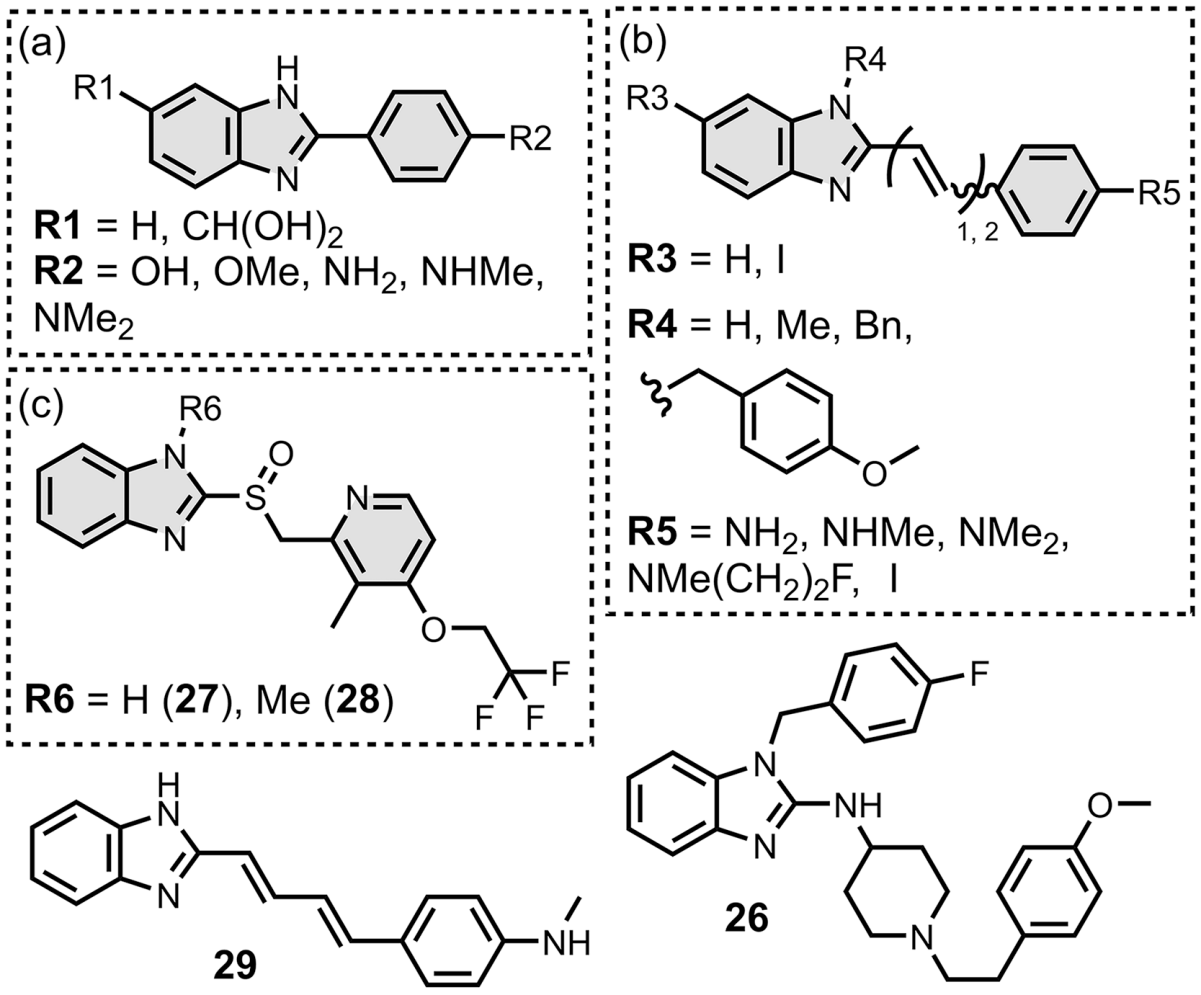
Benzimidazole ligands.

Electron donating groups were studied at R2 in Figure 23a, where a dimethylamino substituent provided the highest affinity for binding to Aβ­(1-42) (*K =* 10 nM), and a hydroxy group the lowest affinity (900 nM).^504^ Both *E*-alkene and *E*,*E*-diene spacers were investigated, as shown in Figure 23b, but these derivatives generally showed lower affinities (100–4,000 nM) than the directly conjugated systems.^554,556,587^ At R5, a dimethylamino substituent was preferred (*K =* 300 nM) over the *des*methyl derivative (*K =* 2,360 nM) for monoalkene benzimidazoles binding to tau.^556^ No significant trend was observed for binding to Aβ­(1-42). Substituents at R4 did not significantly affect the affinity for Aβ­(1-42) or αSyn,^587^ except when the diene spacer was present.^554^ Compounds with *E*- and *Z*-isomers of the alkene spacer had similar affinities.^587^

A sulfonamide linker was also investigated in Figure 23c. The derivative **29** was the only compound that showed a high affinity for tau (*K =* 3.9 nM) and Aβ­(1-42) (*K =* 6.3 nM). The ligand **29** fluorescently labelled SPs and NFTs in AD brain sections, and LBs in DLB brain sections.^630^ However these binding constants were measured by radioligand assays, whereas the other benzimidazoles with alkene spacers were studied using fluorescent assays. As fluorescent assays report only on sites that produce a change in fluorescence, these assay formats may measure binding to different subsets of sites.

### 5.6 Benzofurans

Benzofuran derivatives have primarily been screened with Aβ, with the range of affinities measured summarized in Figure 24. Figure 25 summarizes the benzofuran analogues that have been investigated. A bromo or iodo substituent at R1 (*K =* 0.4–0.6 nM) was preferred over the same substituent at R2 (*K =* 1.6–8 nM).^267^ None of the substituents investigated at R2 significantly affected the measured affinities. On the pendant aryl ring, the substituents explored at R3, R5, X1, and X2 all afforded similar affinities for Aβ­(1-40) (*K =* 2–10 nM) or Aβ­(1-42) (IC_50_ = 19–66 nM).

**Figure 24:**
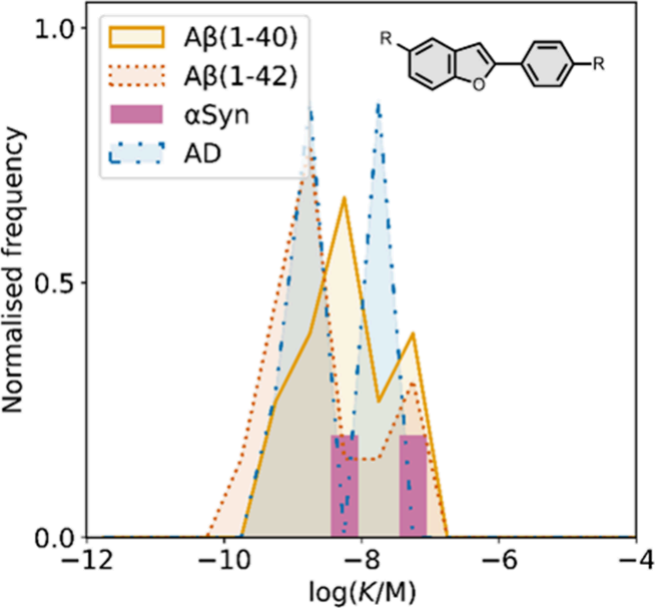
Distribution of binding affinities for the interaction of 34 benzofuran ligands with amyloids: Aβ­(1-40) (solid yellow line, 15 ligands), Aβ­(1-42) (dotted orange line, 13 ligands), αSyn (purple bars, 2 ligands), and AD brain homogenates (dash-dot-dot blue line, 7 ligands). Bars are used to represent three or fewer ligands. For ligands where several binding affinities have been reported, the average value was used.

**Figure 25:**
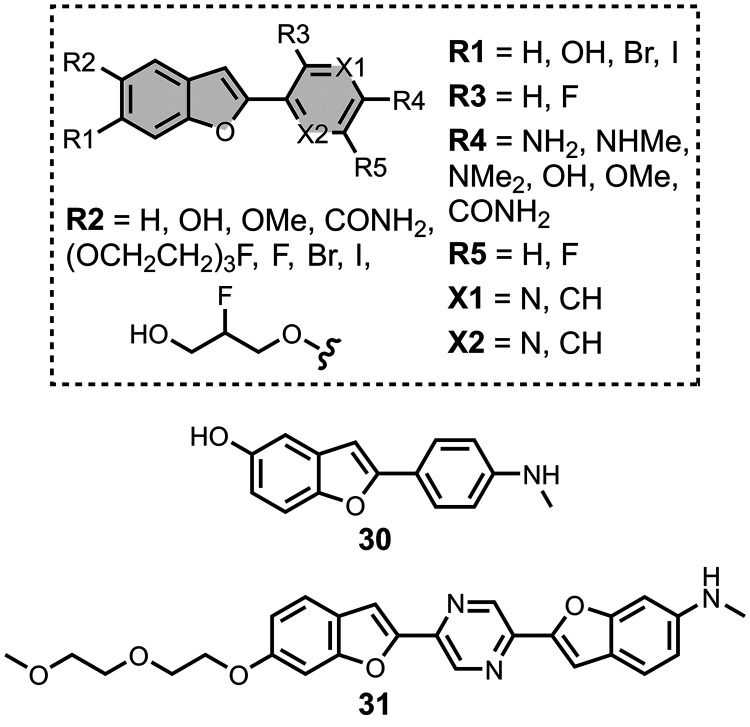
Benzofuran ligands.

For binding to AD brain homogenates, the effects of substituents at R4 in Figure 23 depended on the substituent at R2. When amino or hydroxyl groups were present at both positions, partial methylation of these substituents gave the highest binding affinities (*K =* 0.7–2.8 nM).^462^ **30** exhibited the highest affinity for AD brain homogenates (*K =* 0.7 nM) but bound more weakly to Aβ­(1-40).^462,565^ For binding to Aβ­(1-40) or Aβ­(1-42), substituents at R4 did not significantly affect affinity.^231,267^ A benzofuran dimer, **31**, was reported as a two-photon imaging probe that crossed the blood–brain barrier in mice.^500^ **31** bound Aβ­(1-42) with a modest affinity *in vitro* (*K =* 63.8 nM) and stained NPs in AD brain sections.

### 5.7 Benzoxazoles

Benzoxazole derivatives have been prepared either directly joined to a pendant aryl ring or joined via an alkene linker. Benzoxazoles without an alkene linker will be discussed first, and the reported binding affinities are summarized in Figure 26. Figure 27 summarizes the structures of benzoxazole ligands without an alkene linker. The benzoxazole scaffold was first reported for **32** (commonly known as **IBOX**), which showed good affinity for Aβ­(1-40) (*K =* 0.8 nM) and stained amyloid plaques in AD brain sections.^389^ For the derivatives that were subsequently prepared, substituents at R1 in Figure 27a were generally well tolerated. The malonitrile and acid derivatives exhibited high affinities for Aβ­(1-42) (*K =* 0.5-1.6 nM), with **33** showing the strongest binding (*K =* 0.5 nM). In contrast, a propenoic acid group at R1 bound more weakly (*K =* 15.3 nM).^599^ Fluorinated PEG chains generally afforded low nanomolar affinities (*K =* 2–10 nM).^459^ At R3, dimethylamino derivatives yielded superior binding affinities to Aβ­(1-42) (*K =* 7 nM) over *des*methylamino derivatives (*K =* 140 nM).^550,565^ Fluoroalkylamino derivatives were also prepared and showed worse affinities (IC_50_ = 158-395 nM).^581^

**Figure 26:**
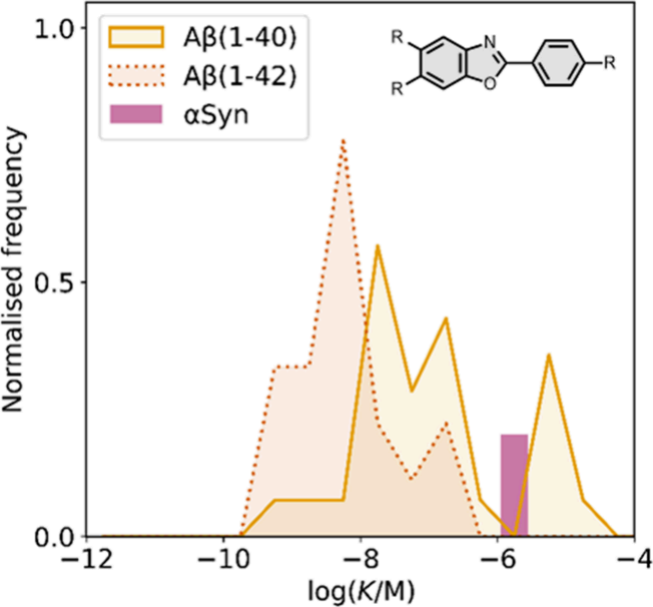
Distribution of binding affinities for the interaction of 46 benzoxazole ligands with amyloids: Aβ­(1-40) (solid yellow line, 28 ligands), Aβ­(1-42) (dotted orange line, 18 ligands), and αSyn (purple bar, 1 ligand). Bars are used to represent three or fewer ligands. For ligands where several binding affinities have been reported, the average value was used.

**Figure 27:**
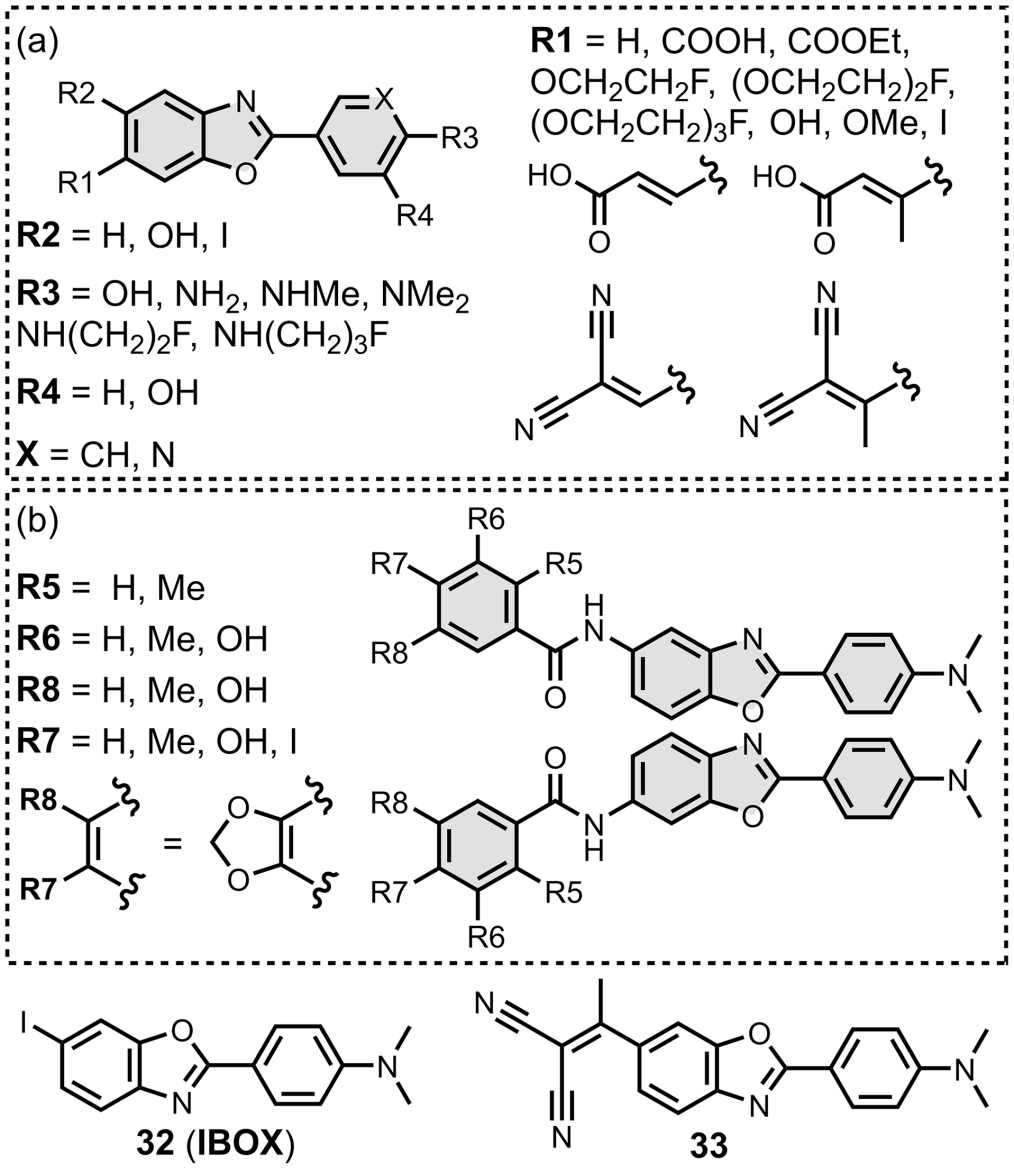
Benzoxazole ligands.

Derivatives with amide-linked aromatic rings, shown in Figure 27b, typically showed affinities for Aβ­(1-40) between 10–20 nM.^594^ An amide-linked aromatic ring was generally preferred at R2 over R1, and at both positions a trimethoxybenzene group exhibited the worst affinity (100–600 nM).

A large number of styryl benzoxazole ligands have also been prepared, with binding affinities summarized in Figure 28.^272,295,404,608^ Derivatives with nanomolar affinities for Aβ and AD brain homogenates have been reported, while comparatively weaker binding to αSyn has been achieved. Figure 29 outlines the structures of the styryl benzoxazole derivatives that have been assayed. The ligand **34** (commonly known as **BF-227**) is the most studied styryl benzoxazole derivative and has a nanomolar affinity for AD brain homogenates (*K =* 33 nM) and αSyn (10–50 nM). The ligand **34** appeared to target two sites on Aβ­(1-42) (*K*_1_ = 1.3–1.7 nM, *K*_2_ = 60–80 nM). While **34** does not bind DLB brain homogenates, both **34** and the hydroxymethene derivative **35** stained amyloid plaques in AD brain sections.^218^

**Figure 28:**
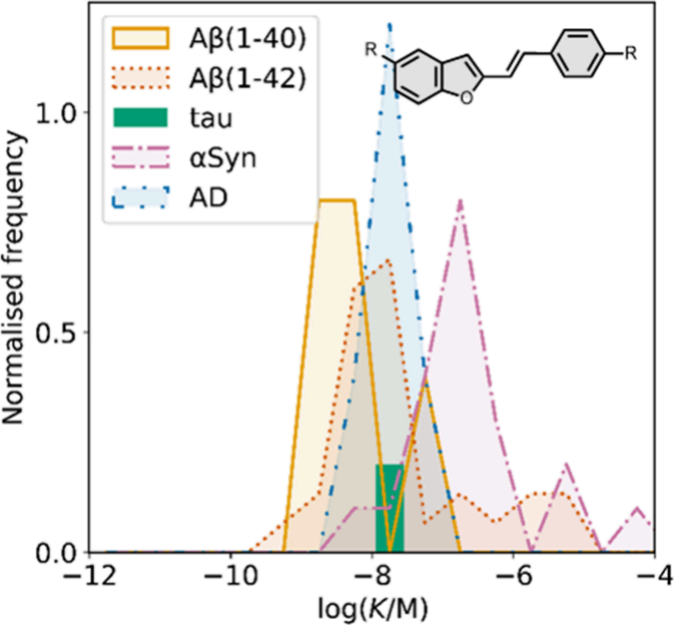
Distribution of binding affinities for the interaction of 36 styryl benzoxazole ligands with amyloids: Aβ­(1-40) (solid yellow line, 5 ligands), Aβ­(1-42) (dotted orange line, 30 ligands), tau (green bar, 1 ligand), αSyn (dash-dot purple line, 20 ligands) and AD brain homogenates (dash-dot-dot blue line, 5 ligands). Bars are used to represent three or fewer ligands. For ligands where several binding affinities have been reported, the average value was used.

**Figure 29:**
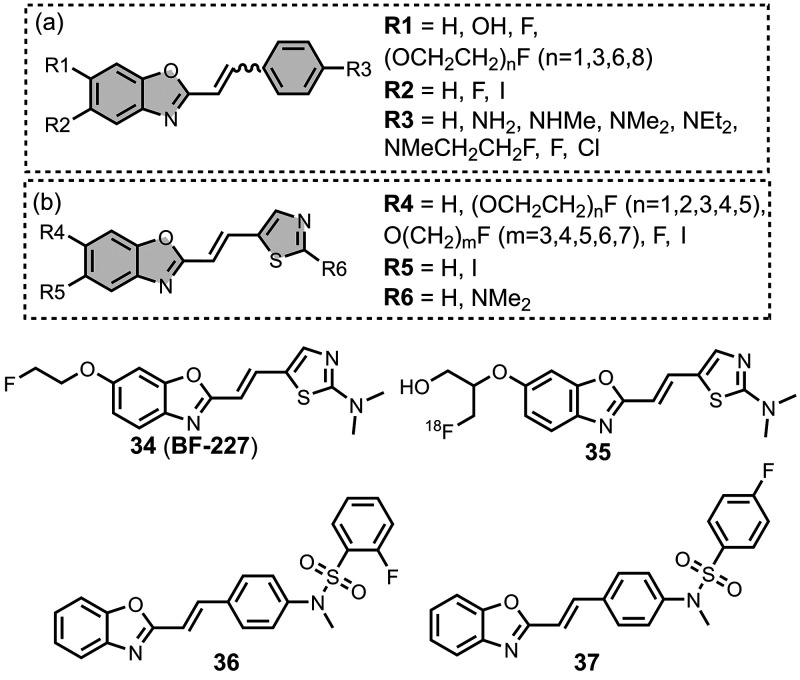
Styryl benzoxazole ligands.

Pendant benzene (Figure 29a) and thiazole rings (Figure 29b) afforded similar affinities for Aβ­(1-42) and αSyn.^608^ For benzene derivatives, FPEG chains at R1 gave nanomolar affinities for AD brain homogenates (6–12 nM) (Figure 29a).^375^ For binding to Aβ isoforms, substituents at R1 or R2 did not significantly affect affinity (<10 nM).^452^ However, the substituent at R3 was critical. Amines at R3 generally yielded nanomolar affinities (<10 nM) while fluoro or iodo groups removed activity (>5 μM).^587^ Notably, both the *E*- and *Z*-isomers had similar dissociation constants.^587^

For thiazole derivatives (Figure 29b), a dimethylamino group at R6 improved affinities. At R4, fluorinated PEG and alkyl chains gave similar affinities for Aβ­(1-42) (*K =* 9–31 nM). Greater variation was seen when assayed with αSyn (*K =* 50–460 nM), where PEG chains (*K =* 50–100 nM) were preferred over alkyl chains (*K =* 160–460 nM).^608^

The sulfonamide-linked fluorobenzene derivatives shown in Figure 29 were also prepared (**36**, **37**).^401^ The derivative **36** showed high affinity binding to αSyn (3 nM) and was selective over Aβ­(1-42) (145 nM). The position of the fluorine substituent significantly impacted both the affinity and selectivity, as **37** was selective for Aβ­(1-42) (8 nM) over αSyn (155 nM).

### 5.8 Oxazolopyridines

The reported oxazolopyridines showed nanomolar binding affinities for AD brain homogenates, summarized in Figure 30. Figure 31 outlines the structures of these derivatives. The first oxazolopyridine reported was **38** (commonly known as **DMAB**), which showed a high affinity (25 nM) for AD brain homogenates.^566^ Most of the analogues subsequently prepared had dissociation constants between 10–50 nM. The highest affinity ligand was **39** (*K =* 5 nM). Incorporating 5-membered heterocycles between R1 and R2 maintained high affinity binding aside from a methylimidazole group (*K* > 1 μM). Installing a nitrogen at X1 prevented nanomolar binding (*K >* 1 μM), while at X2 a nitrogen reduced affinity by an order of magnitude at most.

**Figure 30:**
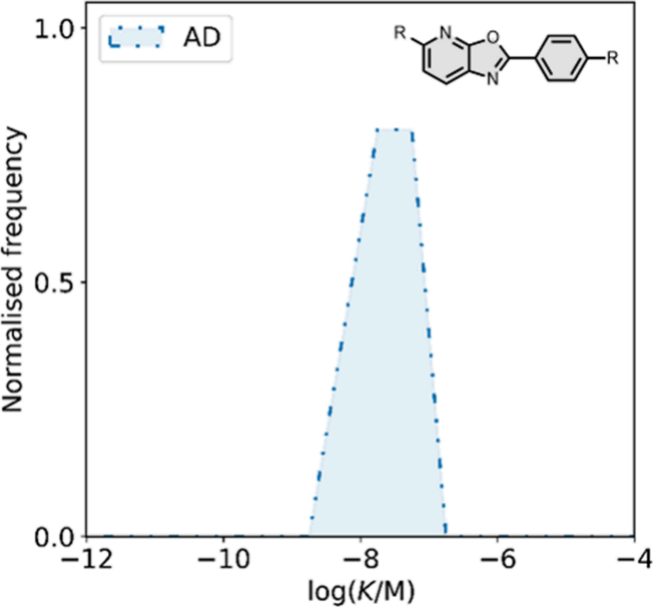
Distribution of binding affinities for the interaction of 15 oxazolopyridine ligands with AD brain homogenates (dash-dot-dot blue line).

**Figure 31:**
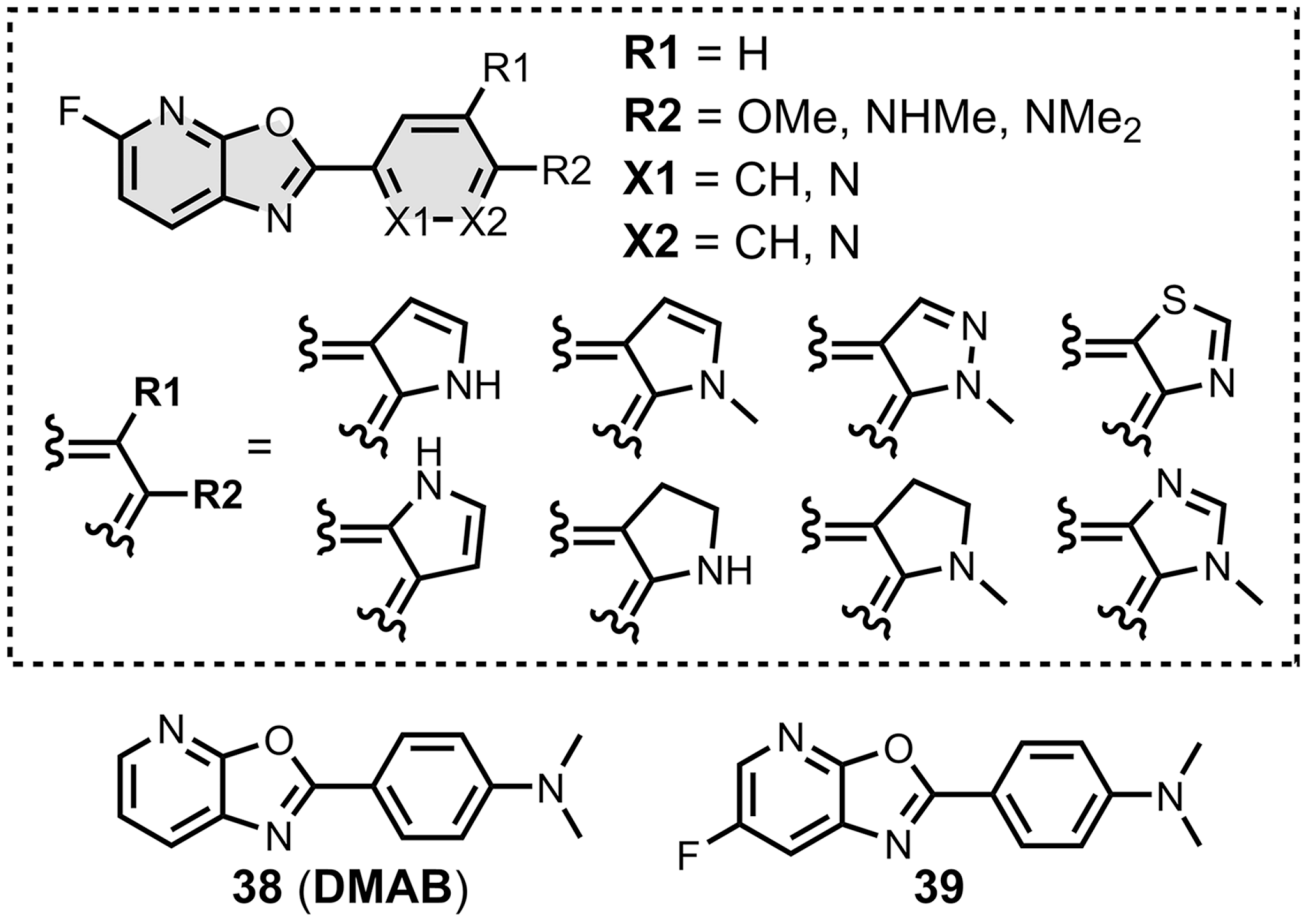
Oxazolopyridine ligands.

### 5.9 Indoles

Indoles have been screened with Aβ­(1-42) with a range of affinities, summarized in Figure 32. Figure 33 shows the indole derivatives that have been prepared. In Figure 33a, at R2, methylation reduced affinity and substitution with a dimethylaniline ring (**40**) prevented binding to Aβ­(1-42) (*K* > 10 μM).^629^ At R3, dialkylated amino substituents led to the highest-affinity derivatives, and monosubstituted amines typically had binding at least one order of magnitude weaker. Several saturated nitrogen heterocycles were also explored at R3 in combination with a pendant pyridine or pyrimidine ring (N at X1 and/or X2). These ligands were screened with various biological brain homogenates, and many had high binding affinities. The ligands **41** and **42** showed strong binding to AD, PSP, CBD, and PiD brain homogenates (*K* = 1–10 nM) and modest binding to PD brain homogenates (*K* = 90–140 nM).^400^

**Figure 32:**
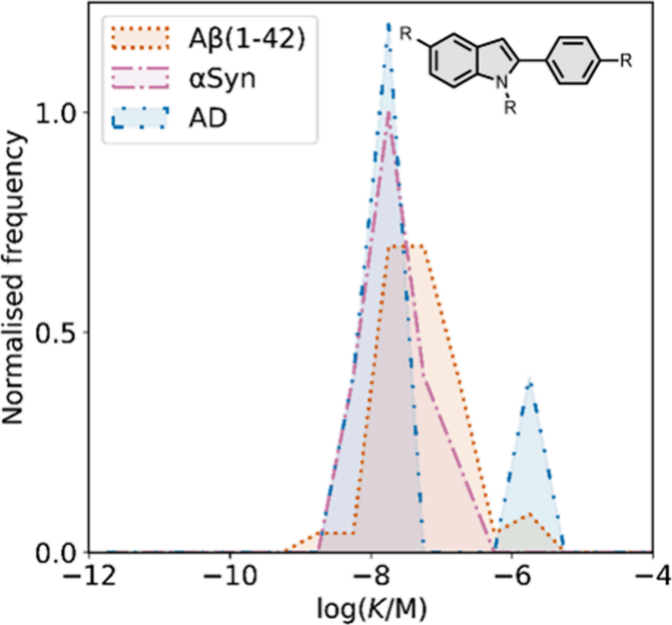
Distribution of binding affinities for the interaction of 52 indole ligands with amyloids: Aβ­(1-42) (dotted orange line, 46 ligands), αSyn (dash-dot purple line, 20 ligands), and AD brain homogenates (dash-dot-dot blue line, 5 ligands). For ligands where several binding affinities have been reported, the average value was used.

**Figure 33:**
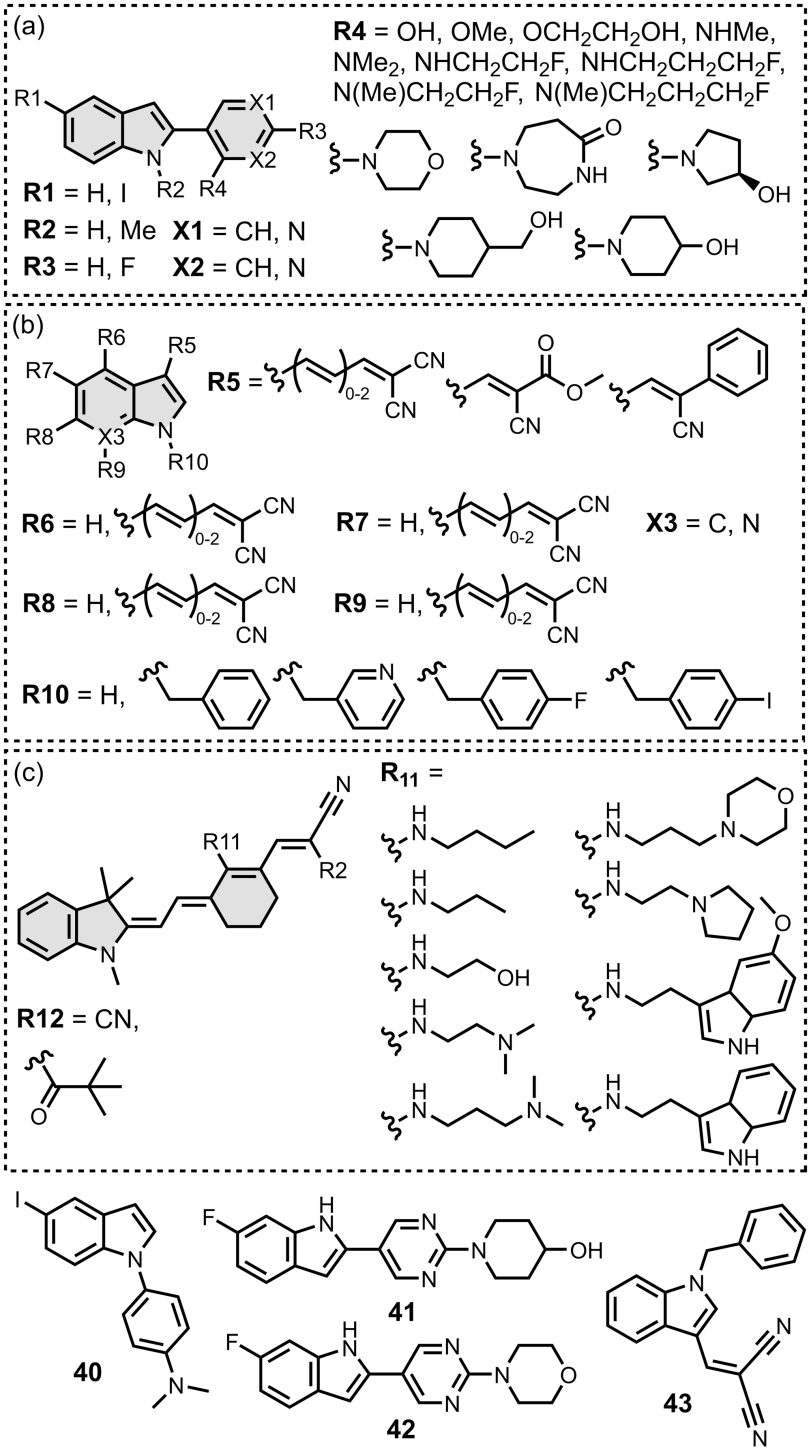
Indole ligands.

Other indole derivatives were explored as αSyn ligands, with benzyl groups at R10 and alkene-linked malonitrile substituents at various positions around the indole core (Figure 33b).^542^ The malonitrile substituent was preferred at R5 for binding to αSyn, and the optimal number of double bonds depended on the substituent’s position. A nitrogen at X3 (i.e. a pyridine) reduced affinity. At R10 a benzyl group produced the strongest binding, leading to the highly selective ligand **43** with a low nanomolar dissociation constant for αSyn (*K* = 3.5 nM) and no measurable binding to Aβ­(1-42).

Finally, several red-emitting indole derivatives with an extended conjugated system have been reported (Figure 33c).^225,229,348,453^ These derivatives all showed similar mid-nanomolar dissociation constants for Aβ­(1-42) (*K* = 30–450 nM).

### 5.10 Isoindolinone

A small number of isoindolinones have been investigated showing affinities for Aβ­(1-42) between 0.3–0.9 nM, as summarized in Figure 34.^212^ The structures of these derivatives are shown in Figure 35.

**Figure 34:**
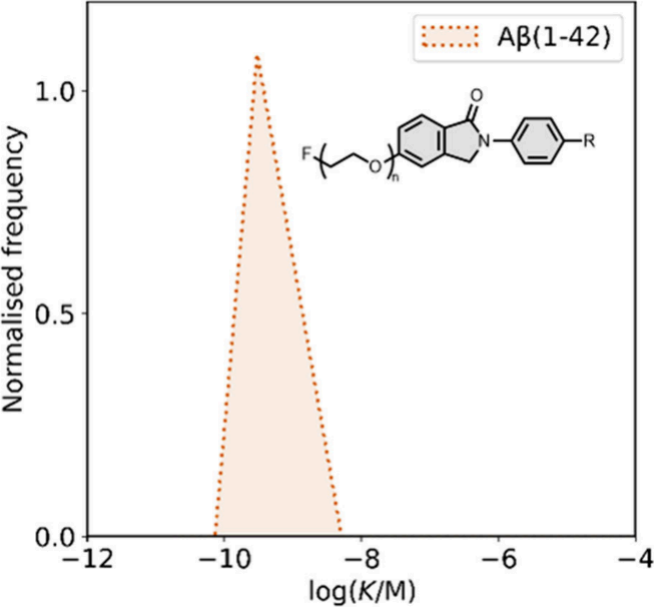
Distribution of binding affinities for the interaction of 6 isoindolinone ligands with Aβ­(1-42) (dotted orange line).

**Figure 35:**
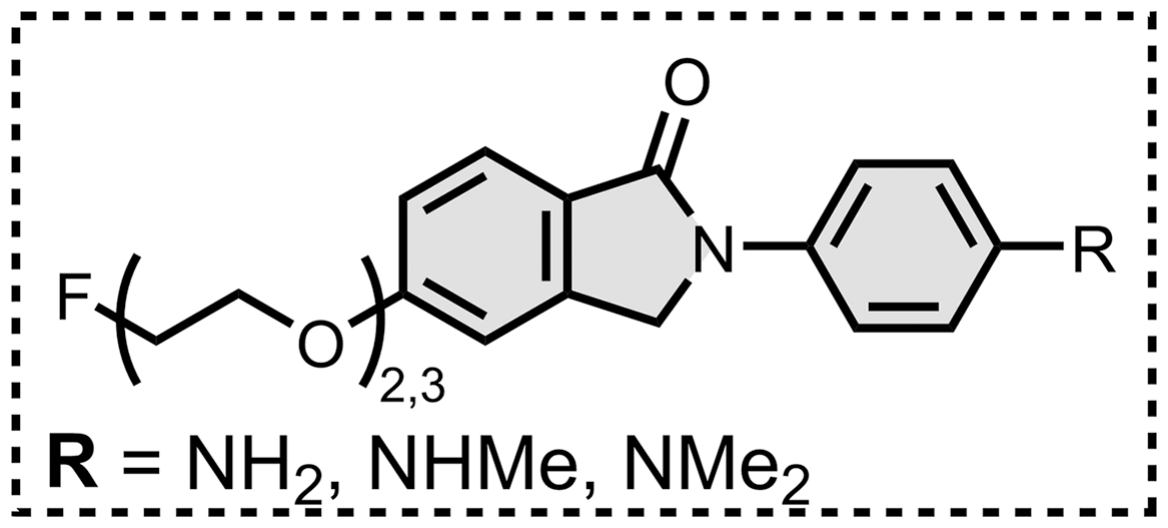
Isoindolinone ligands.

### 5.11 Phthalimides

Phthalamides have been screened with Aβ­(1-42) and AD brain homogenates, showing low- and sub-nanomolar affinities as summarized in Figure 36. The structures of the phthalimide derivatives are shown in Figure 37. For binding to Aβ­(1-42), different FPEG chains at R1 and various methylated aniline rings at R3 all yielded similar binding affinities (*K* = 0.6–0.9 nM). Ligands were screened for binding to AD brain homogenates with different aniline rings substituted at R1. An iodo-substituted ring afforded high affinity binding (*K =* 0.2–0.9 nM), with **44** exhibiting the smallest dissociation constant (*K =* 0.2 nM). Hydroxy-derivatized rings gave the lowest affinity (16–20 nM), and incorporating an aniline ring without any para substituent at R1 or R2 prevented binding (*K >* 1 μM).

**Figure 36:**
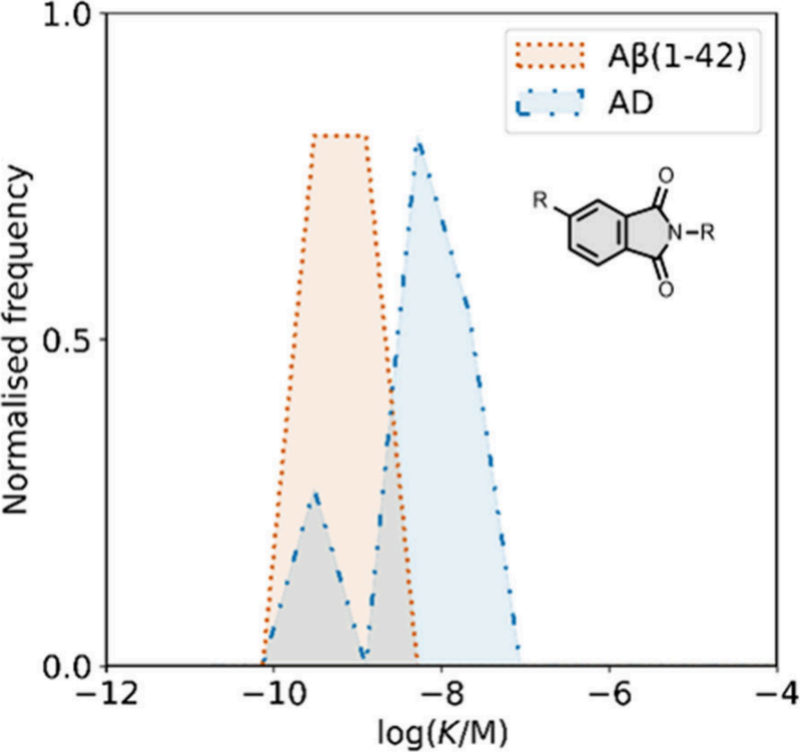
Distribution of binding affinities for the interaction of 12 phthalimide ligands with amyloids: Aβ­(1-42) (dotted orange line, 6 ligands and AD brain homogenates (dash-dot-dot blue line, 6 ligands).

**Figure 37:**
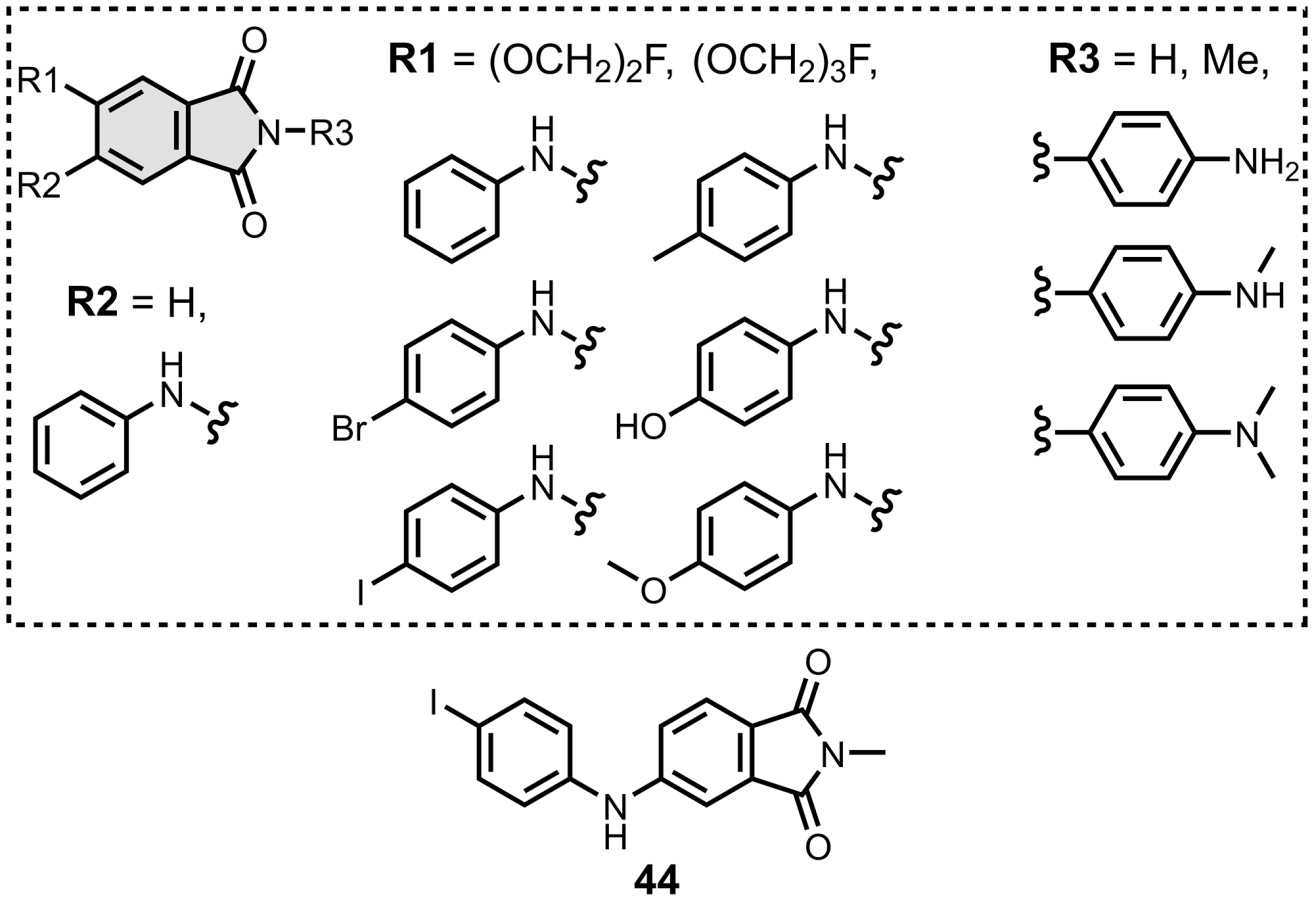
Phthalimide ligands.

### 5.12 Pyrrolopyridines

Pyrrolopyridines selectively bind to tau in AD brain homogenates, with the range of binding affinities summarized in Figure 38.^338,491,629^ These ligands were screened with AD brain homogenates with competition binding assays using one reporting ligand, **45**, that targeted tau and another reporting ligand, **46**, that targeted Aβ. Figure 39 shows the structures of the pyrrolopyridine ligands. The first pyrrolopyridine ligand reported was **47** (commonly known as **MK-6240**), which exhibited high affinity binding to NFT-rich AD brain homogenates (*K =* 0.1–0.4 nM) and bound to phosphorylated tau in AD brain sections.^338,491^ Pyrrolopyridine ligands with an isoquinoline substituent (Figure 39a) generally showed high affinities (< 1 nM) for tau in AD brain homogenates. At R1 or R2, a fluoro group reduced affinity by at least two orders of magnitude. At R3, R4, R5, and R6, installation of alkylated amino or hydroxy groups typically reduced affinity. Analogues that were bifunctionalized at these position with an amino group and a fluoro or iodo group typically showed affinities under 2 nM for tau in AD brain homogenates, although some (**48**) afforded lower affinity binding (208 nM).

**Figure 38:**
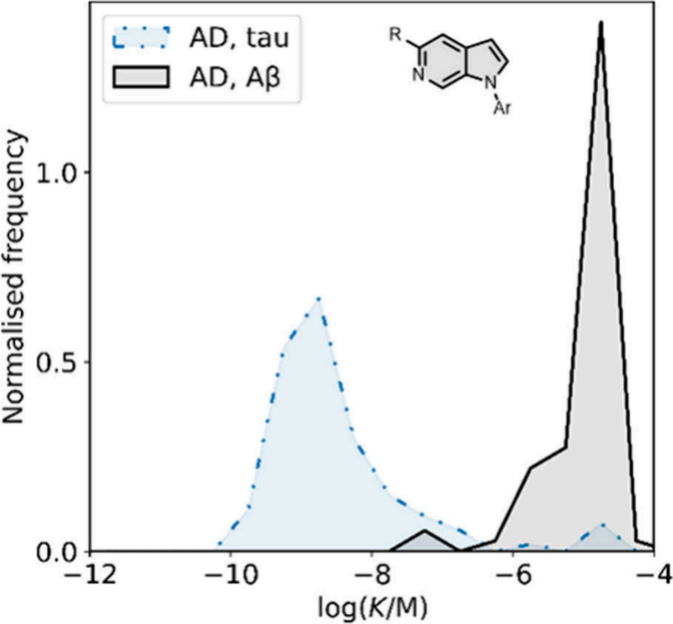
Distribution of binding affinities for the interaction of 108 pyrollopyridine ligands with Aβ in AD brain homogenates (solid black line, 73 ligands), and tau in AD brain homogenates (dash-dot-dot blue line, 108 ligands).

**Figure 39:**
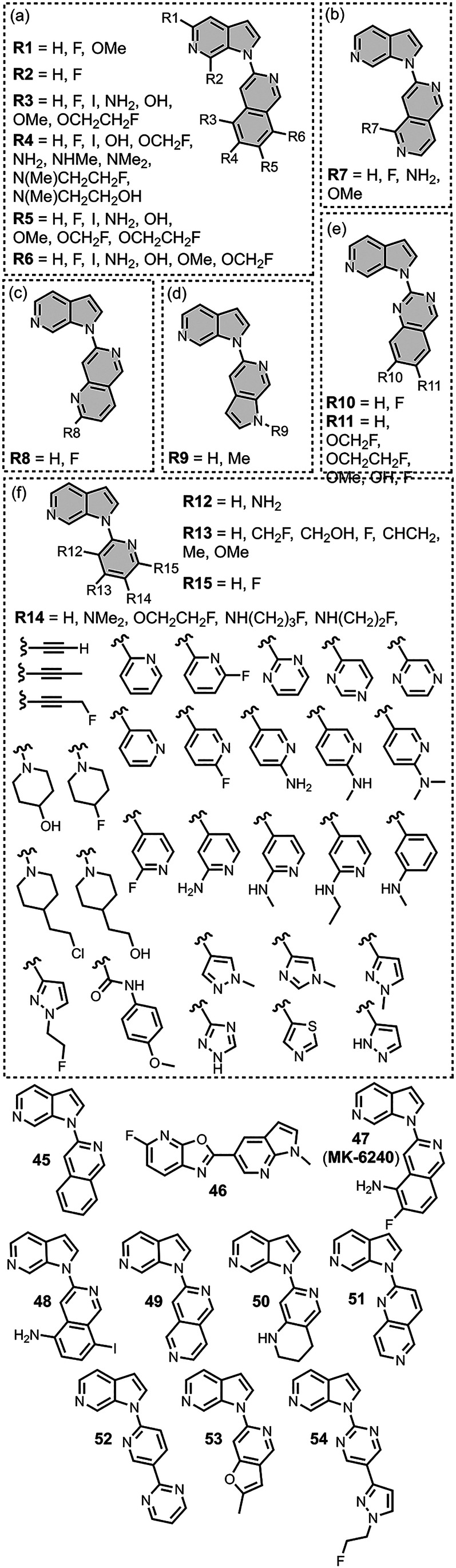
Pyrrolopyridine ligands.

Pyrrolopyridine ligands with a range of other bicyclic substituents were also investigated for binding to tau in AD brain homogenates. Naphthyridine substituents (Figure 39b and Figure 39c) generally afforded low nanomolar dissociation constants (**49**, 2.3 nM). At R7 in Figure 39b, an amino group was tolerated (2.3 nM) but a methoxy group was not (185 nM). Saturation of one of the rings (**50**) reduced affinity (10.8 nM), and a 1,6-naphthyridine substituent (**51**) prevented binding altogether (*K >* 10 μM). In Figure 39e, a fluoro or methoxy group at R11 led to slightly stronger binding (*K =* 1.6–2.6 nM) than a hydroxy or fluoroalkyl group (9–13 nM). Substituents were preferred at R10 over R11 for the ligands in Figure 39e.

In Figure 39f, pyrrolopyridine ligands with a pyridine substituent generally afforded high affinity binding to tau in AD brain homogenates (*K <* 5 nM). Most alkyl, hydroxy, and amino groups at R13 and R14 did not significantly impact affinity (1–4 nM), except for a dimethylamino group at R14 (*K >* 10 μM). Several ring systems were investigated at R14, and 5-membered aromatic heterocycles (*K =* 1.1–8.8 nM) and piperidine groups (*K =* 3–71 nM) all gave nanomolar affinities. Pyridine rings at R14 also afforded high binding affinities (0.5–2.0 nM) except when a fluoro group was substituted at R15 (*K =* 20 nM). Pyrazines and pyrimidines were also explored at R14, and notably, **52** reduced affinity to > 1 μM. Pyrrolopyridine ligands with other aromatic substituents (e.g. **53**, **54**) also gave low nanomolar affinities (*K =* 2.1–2.4 nM).

### 5.13 Comparing Structural Cores of Fused Benzoheterocycles

Several publications directly compared different fused benzoheterocycle structural cores (Figure 40). In Figure 40a, Swahn *et al*. investigated various substituents on benzothiazole, benzoxazole, and benzofuran derivatives, as well as the inclusion of pendant pyridine rings.^565^ Benzothiazole and benzofuran derivatives generally afforded higher binding affinities for Aβ­(1-40) than benzoxazole derivatives. When comparisons were possible, a similar trend was observed for fluorinated derivatives.^581^

**Figure 40:**
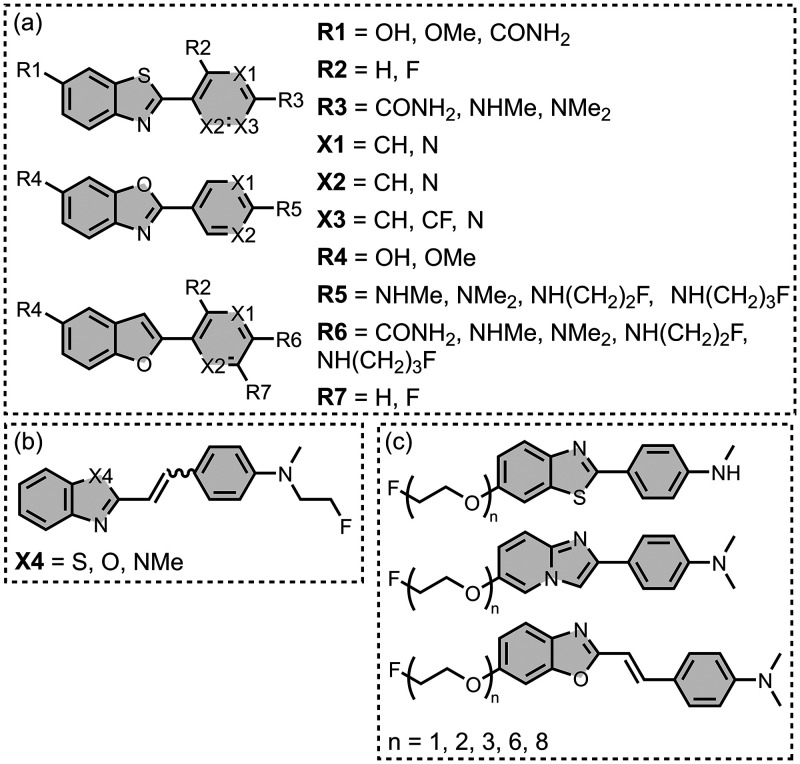
Fused benzoheterocycle ligands with different structural cores.

Figure 40b shows styryl benzothiazoles, benzoxazoles, and benzimidazoles bearing an *N*-fluoroethyl substituent on the pendant aniline ring that were prepared by Morais *et al*.^587^ By fluorescence saturation assays, most ligands had affinities between 1000–10,000 nM to Aβ­(1-42) and αSyn. The *E* and *Z* isomers of these ligands had similar binding affinities.

Stephenson *et al*. compared three structural cores in Figure 40c while investigating different FPEG substituents.^375^ Benzothiazoles (*K =* 2–9 nM) and styrylbenzoxazoles (*K =* 6–15 nM) showed similar affinities for AD brain homogenates, whereas the affinity of imidazopyridines (*K =* 16–387 nM) was lower. The effects of the PEG chain length on affinity also differed between structural cores.^375^ From one to eight PEG repeats, the affinity of benzothiazoles and styrylbenzoxazoles was relatively constant. For imidazopyridines, binding affinity decreased with increasing PEG chain length.

Overall, several broad observations can be made regarding the fused benzoheterocycle binding data. First, the *E* and *Z* isomers of styrene analogues generally exhibited similar binding affinities. Second, the affinity of ligands is commonly modified by changing the degree of methylation on a pendant aniline (or phenol) ring or by changing the length of a PEG chain on the benzoheterocycle core. Third, replacing the pendant benzene ring for a pyridine ring typically reduced the lipophilicity of the ligand without a significant loss of affinity. Fourth, the binding affinity and selectivity were dependent on whether the benzoheterocycle core was substituted at the 5- or 6-position. Many high-affinity fused benzoheterocycle ligands have been reported, but developing ligands that are selective and have promising *in vivo* properties is more challenging.

## 6 Oxindoles and Indolones

Oxindoles and indolones have been investigated as ligands for Aβ, tau, and αSyn with the range of affinities measured shown in Figure 41. Figure 42 shows the oxindole analogues studied. Honson *et al.* first identified oxindoles as amyloid ligands and potential aggregation inhibitors.^333^ A subsequent SAR study by Chu *et al*. delivered several oxindole ligands selective for αSyn.^312^ Pharmacophore modelling was later performed to design oxindoles as aggregation inhibitors *in silico*.^186^

**Figure 41:**
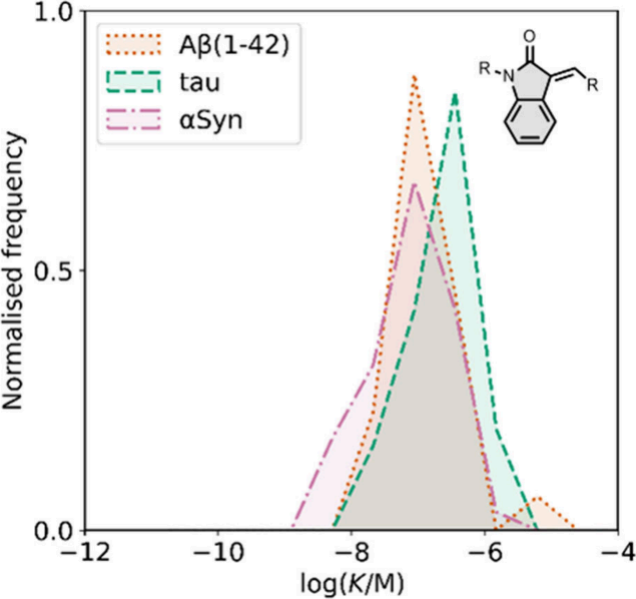
Distribution of binding affinities for the interaction of 50 oxindole ligands with amyloids: Aβ­(1-42) (dotted orange line, 50 ligands), tau (dashed green line, 50 ligands), and αSyn (dash-dot purple line, 46 ligands). For ligands where several binding affinities have been reported, the average value was used.

**Figure 42:**
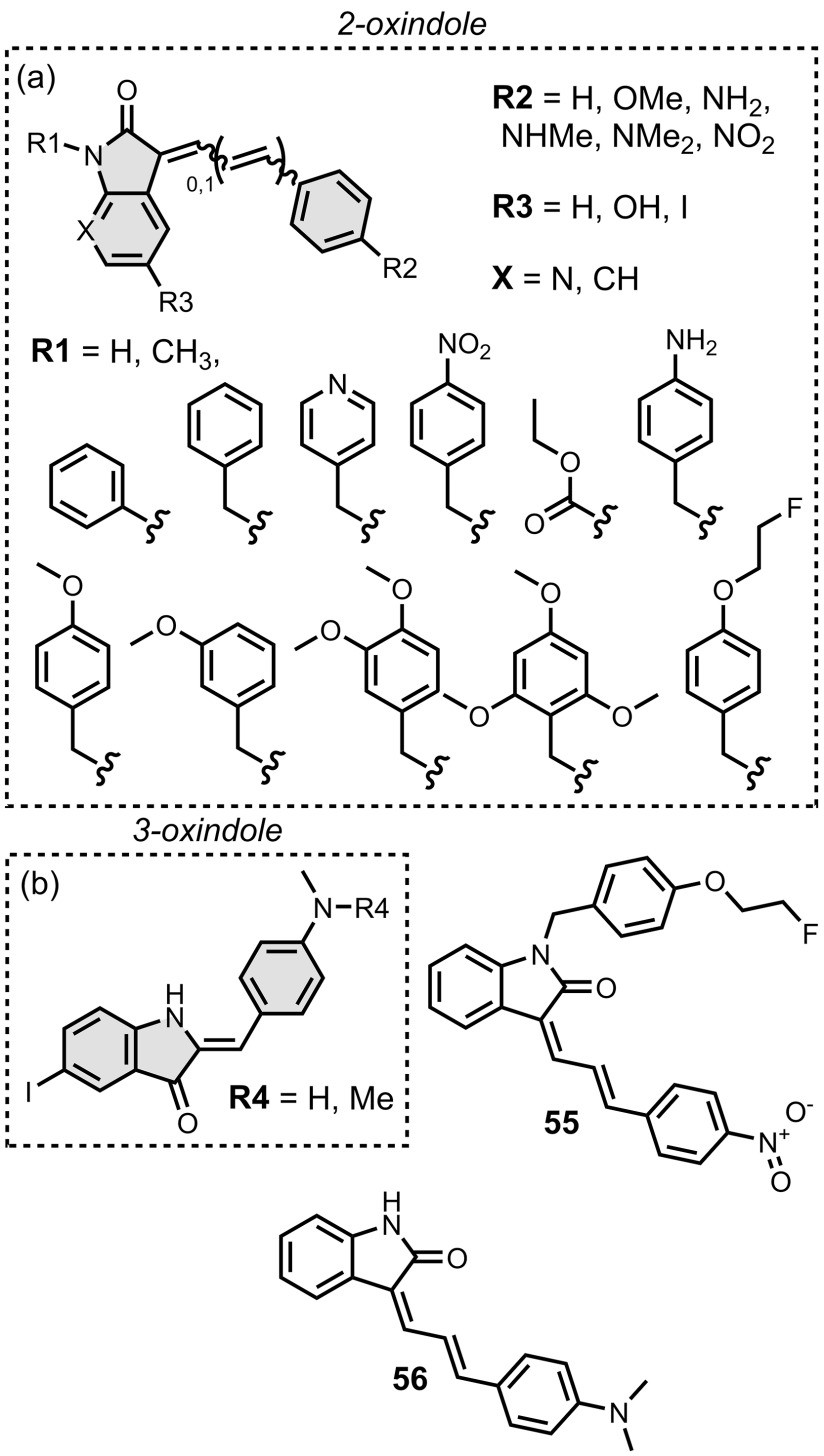
Oxindole ligands.

Ligands were typically prepared and screened as a mixture of the *E*/*Z* isomers, making trends in SAR difficult to identify. 3-Oxindoles (Figure 42b) exhibited stronger binding to tau and Aβ­(1-42) (*K =* 20–120 nM) than the 2-oxindole analogues (Figure 42a) (*K =* 600–5,000 nM).^553^ On 2-oxindoles in Figure 42a, diene linkers to the pendant aromatic ring generally improved binding affinity. At R1, large substituents typically improved both the binding affinity and selectivity for αSyn over tau or Aβ­(1-42). No clear SAR was apparent at X, R2, or R3. The derivative **55** showed the highest affinity binding to αSyn (*K =* 2–9 nM) and was selective over Aβ­(1-42) (*K =* 140–270 nM) and tau (*K =* 50–80 nM). However, **55** was considered a poor *in vivo* candidate due to its hydrophobicity (log *P* = 4.18). Ligand **56** was instead used to label Lewy bodies in PD/DLB brain sections and Aβ plaques in AD brains, and bound to Aβ­(1-42), tau, and αSyn with a similar affinity (*K =* 28–41 nM).

## 7 Indanones

Indanones and several similar scaffolds have been investigated as ligands for a range of targets with the binding affinities shown in Figure 43.

**Figure 43:**
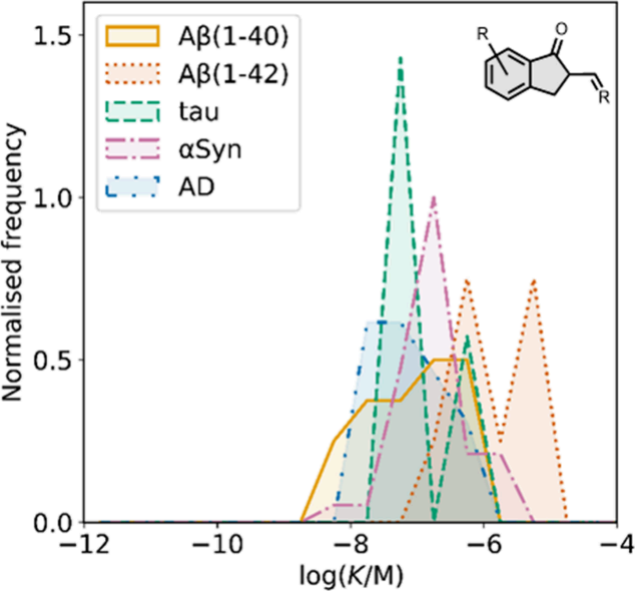
Distribution of binding affinities for the interaction of 71 indanone ligands with amyloids: Aβ­(1-40) (solid yellow line, 16 ligands), Aβ­(1-42) (dotted orange line, 8 ligands), tau (dashed green line, 7 ligands), αSyn (dash-dot purple line, 38 ligands) and AD brain homogenates (dash-dot-dot blue line, 13 ligands). For ligands where several binding affinities have been reported, the average value was used.

Figure 44 shows the structures of the indanone analogues that have been investigated. Indanones were first prepared as structural analogues of donepezil (**57**), an existing medication for Alzheimer’s disease.^467^ On a 1-indanone scaffold (Figure 44a), a dimethylamino group at R7 and methoxy groups at R2 and R3 produced the highest-affinity indanone **58** (*K =* 16 nM). Substituents at R2 generally led to higher affinity binding than the equivalent substituent at R3. Methoxy groups at these positions were favored over bromo or iodo groups. At R7 a methylated amino group, such as in **58** (*K =* 16 nM), was favored over methoxy or bromo substituents (*K =* 111–290 nM), and a fluorine, nitro, *des*methylamino, or hydrogen removed binding altogether (*K >* 1,000 nM). At R6, a bromo substituent also abolished binding.

**Figure 44:**
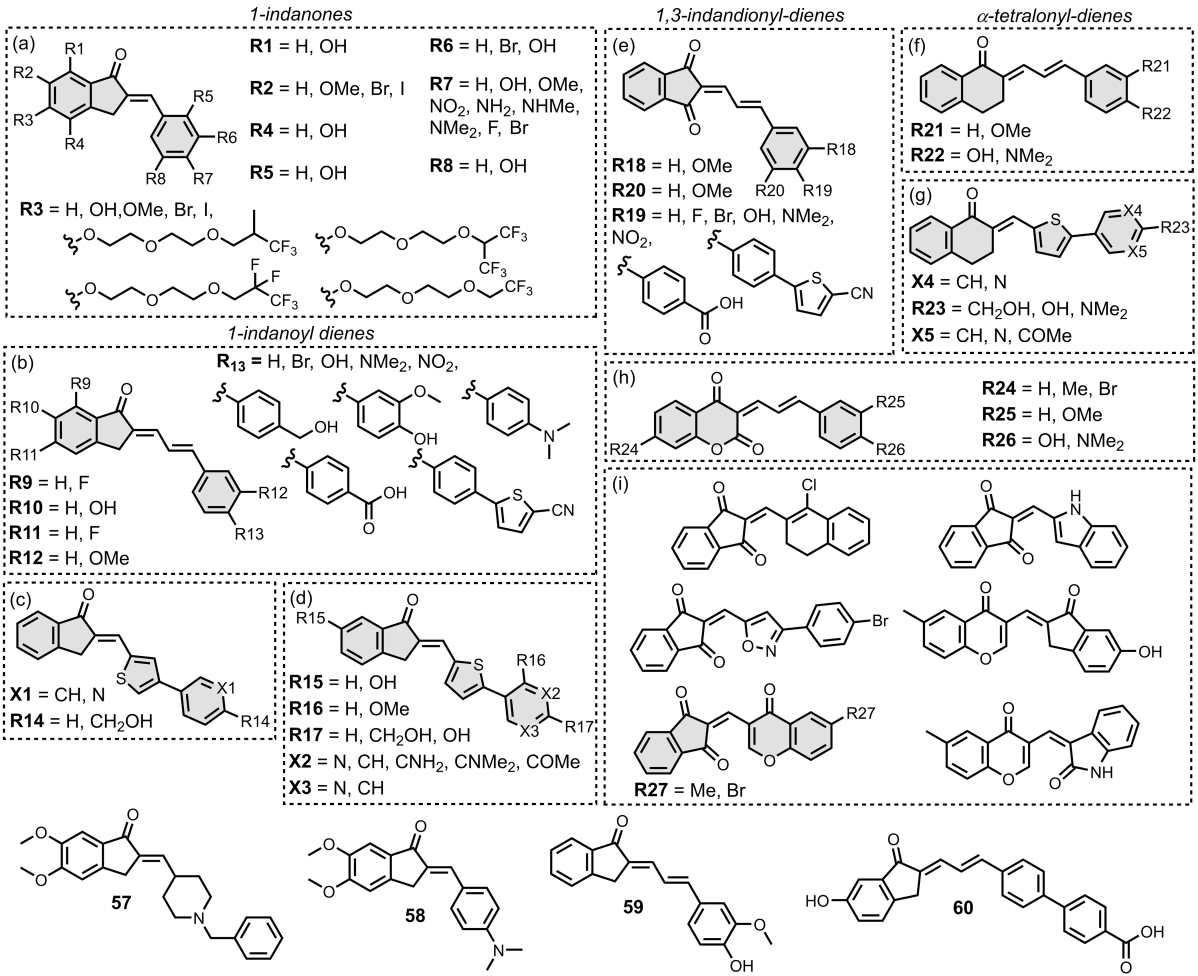
Indanone ligands.

Indanones showed higher-affinity binding to Aβ­(1-40) than AD brain homogenates, mainly due to a greater tolerance of different substituents at R7 in Figure 44a.^197^ Dimethylamino or monomethylamino groups at R7 led to the highest affinity ligands for AD brain homogenates (*K =* 6–27 nM). FPEG chains at R3 led to high nanomolar affinities (*K =* 170–750 nM).^442^ Several polyhydroxylated indanones were also screened with tau and achieved mid nanomolar affinities (42–90 nM).^363^

A large SAR study by Sun *et al*. studied indanones bearing diene linkers as αSyn ligands (Figure 44b).^312^ A hydroxy group at R10, or fluorine groups at R11 and R9, reduced the binding affinity by approximately an order of magnitude. The hydroxy derivative **59** exhibited the highest-affinity binding measured for αSyn (*K =* 9 nM) and showed selectivity over both Aβ (*K =* 140 nM) and tau (*K =* 390 nM). **59** also fluorescently labelled Lewy pathology in PD brain sections. A dimethylamino or nitro group at R13 in Figure 44b led to nanomolar binding affinities (38 nM) whereas a bromo derivative demonstrated no measurable binding. Exploring aromatic substituents at R13 led to **60**, which showed good selectivity for αSyn (*K =* 20 nM) over Aβ (*K =* 490 nM) and tau (*K =* 580 nM). The ligands in Figure 44c and Figure 44d, where the second alkene unit is masked as a thiophene, generally showed poor affinity. Several analogues with 1,3-indandione, tetralon, and coumarin cores were also prepared and generally showed weaker binding than the indanone analogues (Figure 44e–h), as did the fused-ring derivatives in Figure 44i.^190^ The highest affinity indanone ligands (*K <* 100 nM) showed a fluorescence enhancement and a bathochromic shift upon binding to fibrils.

## 8 Aurones and Benzofuranones

Aurones and benzofuranones displayed nanomolar affinities to fibrils, with some selectivity for αSyn as summarized in Figure 45. The structures of the aurone and benzofuranone ligands are summarized in Figure 46. All substituents investigated at R4 showed low nanomolar affinities for Aβ­(1-42) (*K =* 1–7 nM) (Figure 46a).^263,472^ The derivative **61** demonstrated both a high binding affinity (*K =* 4.2 nM) and specifically labelled amyloid plaques in AD brain sections. Polyhydroxylated derivatives were screened with tau and exhibited modest nanomolar affinities (*K =* 22–90 nM).^363^

**Figure 45:**
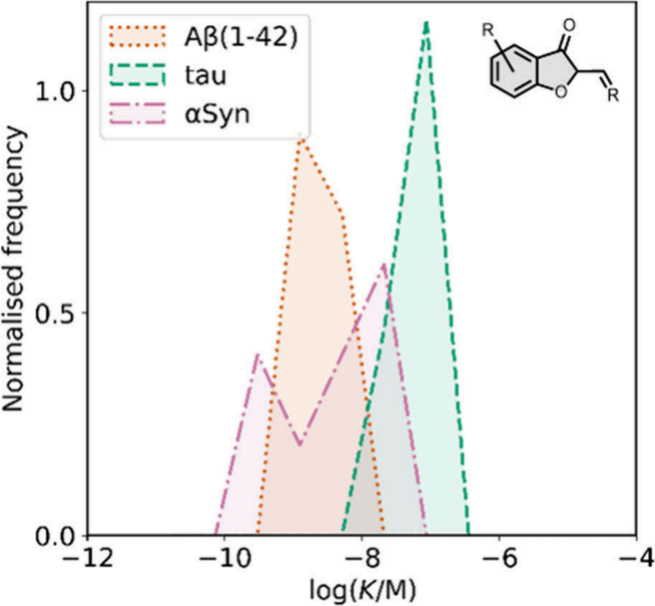
Distribution of binding affinities for the interaction of 24 aurone and benzofuranone ligands with amyloids: Aβ­(1-42) (dotted orange line, 9 ligands), tau (dashed green line, 7 ligands), and αSyn (dash-dot purple line, 8 ligands). For ligands where several binding affinities have been reported, the average value was used.

**Figure 46:**
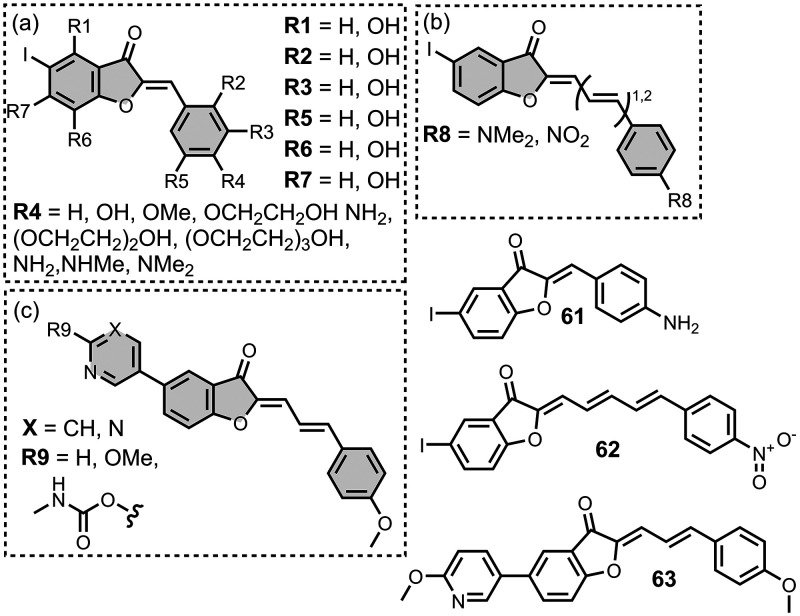
Aurone and benzofuranone ligands.

Benzofuranone-dienes and trienes in Figure 46b were prepared and screened with both αSyn and Aβ.^580^ The triene derivatives exhibited higher binding affinities (*K =* 0.3–1 nM) than the diene derivatives (*K =* 1–4 nM), but with no selectivity between αSyn and Aβ. The ligand **62** demonstrated the highest binding affinity for αSyn (*K =* 0.3 nM) and also bound to Aβ (*K =* 1.2 nM). Dienes with an aryl ring in place of an iodo substituent were prepared and bound to αSyn with nanomolar dissociation constants (*K =* 2–35 nM) (Figure 46c).^559^ The highest-affinity binding to αSyn (*K =* 1–2 nM) was achieved by **63**.

## 9 Rhodanines

Rhodanines display mid-nanomolar affinities for Aβ and tau, as shown in Figure 47. The structures of the rhodanine ligands are summarized in Figure 48. Rhodanines were first observed to inhibit tau aggregation and promote NFT disassembly by Bulic *et al*.^522^ Several rhodanine and thiohydantoine derivatives have since been studied for fibril binding activity and anti-aggregation properties.^328,371,521^ The ligand **64** exhibited the highest affinity for tau (*K =* 10 nM), although it also bound to Aβ­(1-40) with a similar affinity (*K =* 46 nM).

**Figure 47:**
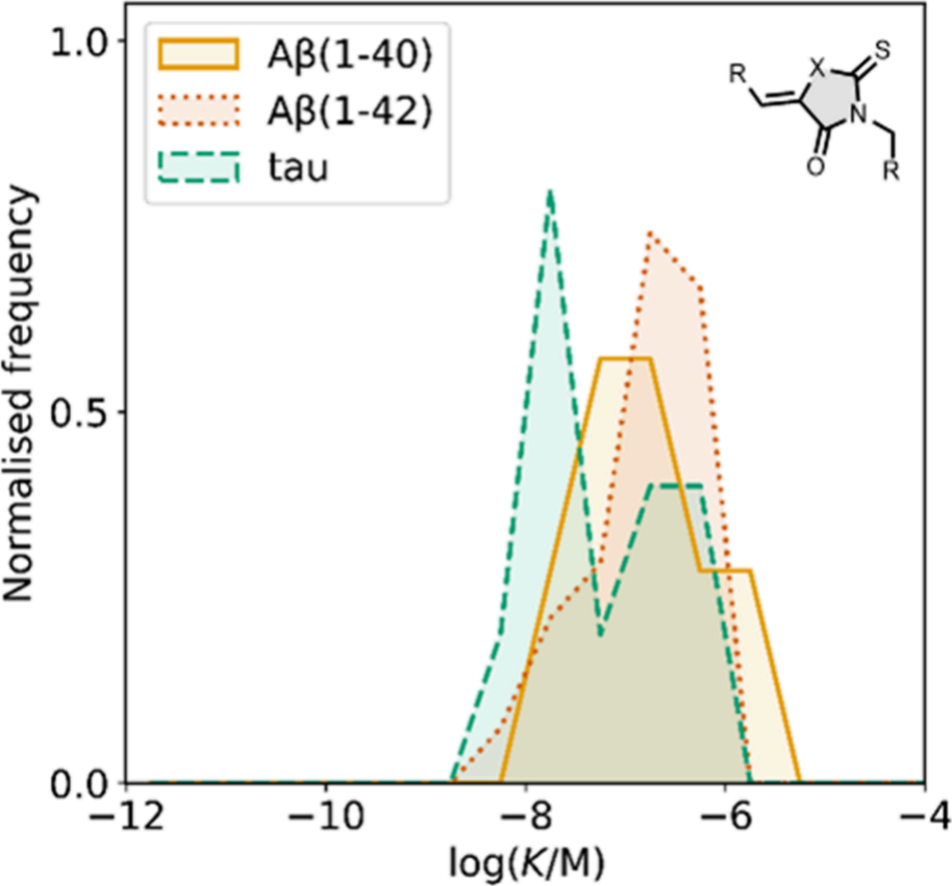
Distribution of binding affinities for the interaction of 33 rhodanine ligands with amyloids: Aβ­(1-40) (solid yellow line, 7 ligands), Aβ­(1-42) (dotted orange line, 27 ligands), and tau (dashed green line, 10 ligands). For ligands where several binding affinities have been reported, the average value was used.

**Figure 48:**
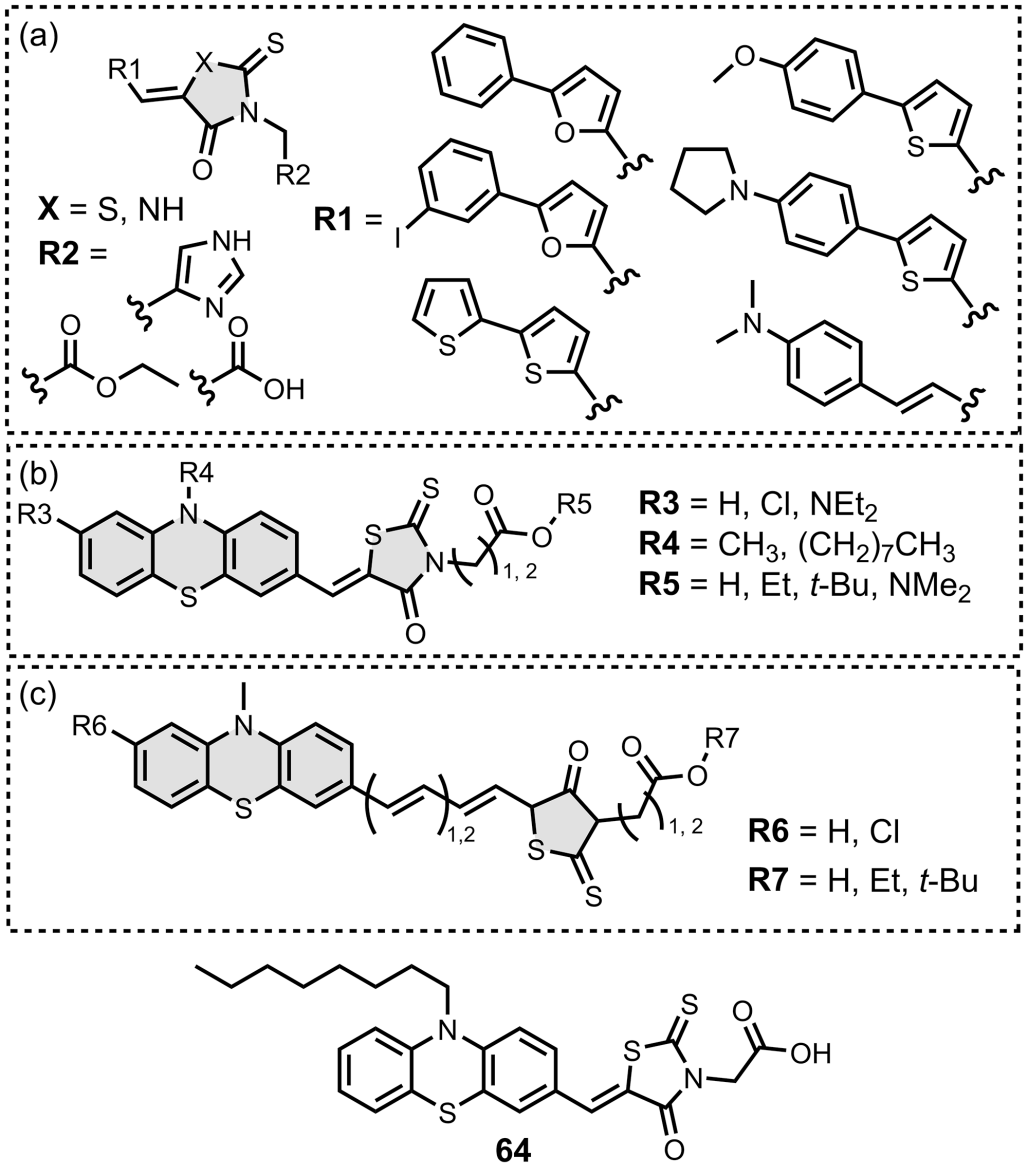
Rhodanine ligands.

Modifications at R1 in Figure 48a gave largely similar dissociation constants for tau (*K =* 14–32 nM) and Aβ­(1-40) (*K =* 28–150 nM), with selectivities for binding to tau of up to an order of magnitude.^371^ Only the phenylfuran derivative showed different binding properties, with a poorer affinity for both tau (*K =* 330 nM) and Aβ­(1-40) (*K =* 2,500 nM). At X in Figure 48a, both an S and NH yielded similar affinities for tau and Aβ­(1-42).^521^ Phenothiazine conjugates were also explored and showed red-shifted emissions above 650 nm (Figure 48b,c).^328,371^ Substituents at R3 in Figure 46b generally reduced affinity, and at R4 a longer alkyl substituent increased the affinities for tau and Aβ­(1-42). At R2 and R5, a free acid generally gave higher binding affinities for Aβ than the ethyl ester.

## 10 Flavones and Chromanones

The flavones reported have been exclusively screened with Aβ and show high nanomolar dissociation constants (Figure 49). The structures of the flavone ligands are summarized in Figure 50. Flavones were first reported as Aβ aggregation inhibitors and were later shown to bind mature amyloid fibrils.^490^ On a flavone scaffold (Figure 50a), electron donating groups are well tolerated at R2 (*K =* 12-77 nM) with dimethylamino substituents yielding the highest affinities for Aβ.^208,506^ At R1, halogen and fluorinated PEG chains led to high-affinity binders.

**Figure 49:**
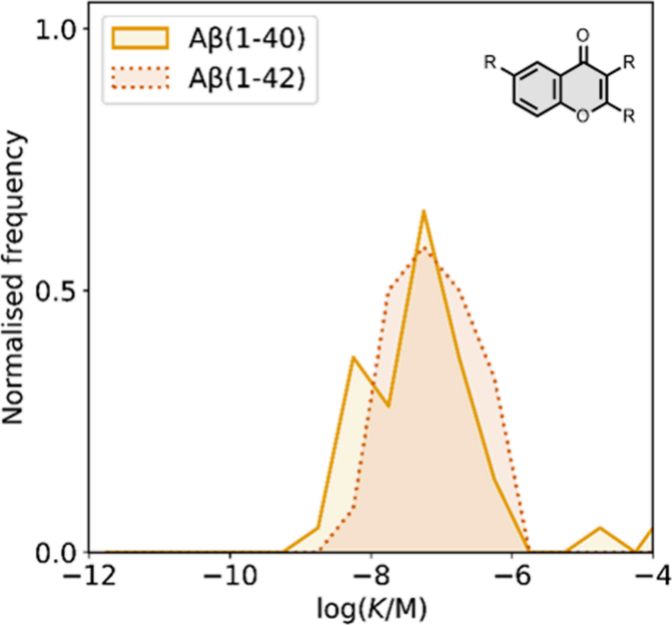
Distribution of binding affinities for the interaction of 62 flavone and chromanone ligands with amyloids: Aβ­(1-40) (solid yellow line, 43 ligands) and Aβ­(1-42) (dotted orange line, 24 ligands). For ligands where several binding affinities have been reported, the average value was used.

**Figure 50:**
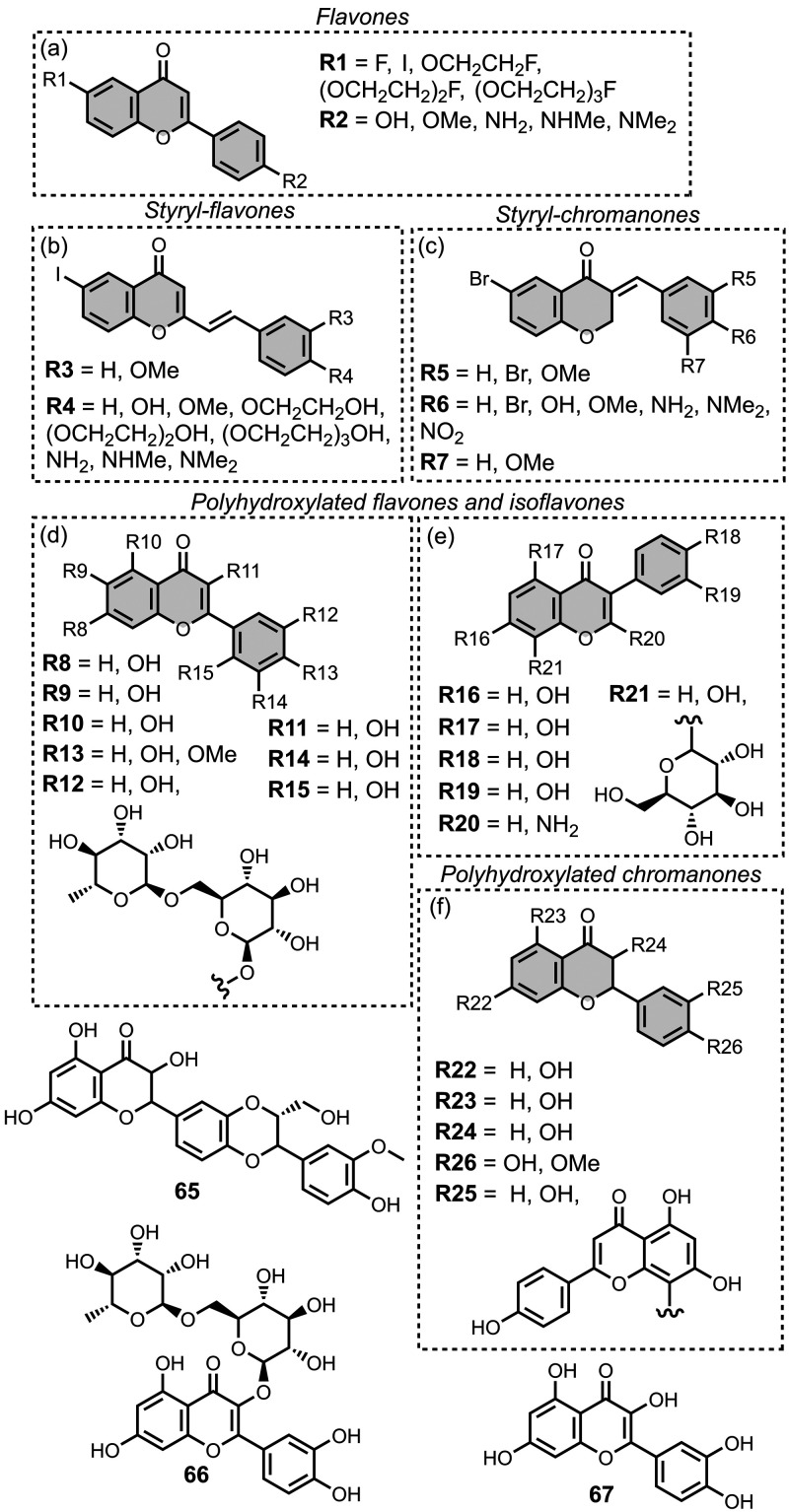
Flavone and chromanone ligands.

Styryl-flavones with an alkene spacer in Figure 50b demonstrated nanomolar affinities with methylated amine and hydroxyl substituents at R4 (*K =* 22 nM).^323,561^ Introducing PEG chains with a terminal hydroxyl group at R4 reduced the binding affinity (*K >* 400 nM). On the pendant aromatic ring, a methoxy group at R3 in addition to a methoxy group at R4 was well tolerated (*K =* 50 nM).

Chromanones with a directly conjugated styrene group were also prepared (Figure 50c).^388^ A methylated, electron-donating group at R6 was essential for high affinity binding (*K =* 9–10 nM), while a substituent at R5 or R7 reduced the binding affinity (*K =* 25–420 nM).

A range of polyhydroxylated flavones and isoflavones were prepared (Figure 50d, e). Most exhibited nanomolar dissociation constants for Aβ (*K =* 2–391 nM), although an amino group at R20 resulted in micromolar dissociation constants (*K* = 14.7–313 μM).^184^ Larger substituents reduced binding affinities (**65**, *K =* 241 nM; **66**, *K =* 391 nM), while the derivative **67** with five hydroxy groups exhibited the highest binding affinity (2 nM). The position of the pendant aryl ring, and the use of a chromanone core, affected the binding affinity of the ligand by up to an order of magnitude.

## 11 Stilbenes

The stilbenes reported have been mainly tested for binding to Aβ, with affinities summarized in Figure 51. Stilbene derivatives have been prepared with various substituents on the main stilbene scaffold, modifications to the pendant aromatic rings and the alkene linker, as well as stilbene dimers. Figure 52 summarizes the chemical structures of stilbenes with different substituents on the main scaffold. The structures resemble the 6,5-fused benzoheterocycles and one side of Congo red (**CR**, **68**, see Congo red derivatives section). Stilbenes were first used as amyloid ligands by Kung *et al.*, who also observed that these ligands compete with 6,5-fused benzoheterocycles on Aβ­(1-40) but not *bis*styryl benzenes.^473^ One of the most successful ligands of this class is **69** (commonly known as **AV-45** or **florbetapir**), which has been approved for use as a PET imaging agent in patients being evaluated for AD. The closely related analogue **212** (commonly known as **AV-1** or **florbetaben**) has also been approved as a PET imaging agent for AD.

**Figure 51:**
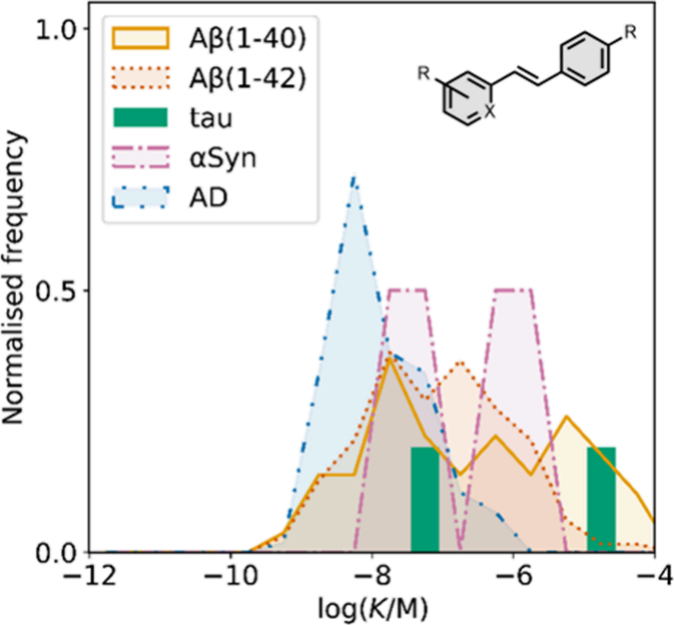
Distribution of binding affinities for the interaction of 287 stilbene ligands with amyloids: Aβ­(1-40) (solid yellow line, 54 ligands), Aβ­(1-42) (dotted orange line, 131 ligands), tau (green bars, 2 ligands), αSyn (dash-dot purple line, 4 ligands) and AD brain homogenates (dash-dot-dot blue line, 105 ligands). Bars are used to represent three or fewer ligands. For ligands where several binding affinities have been reported, the average value was used.

**Figure 52:**
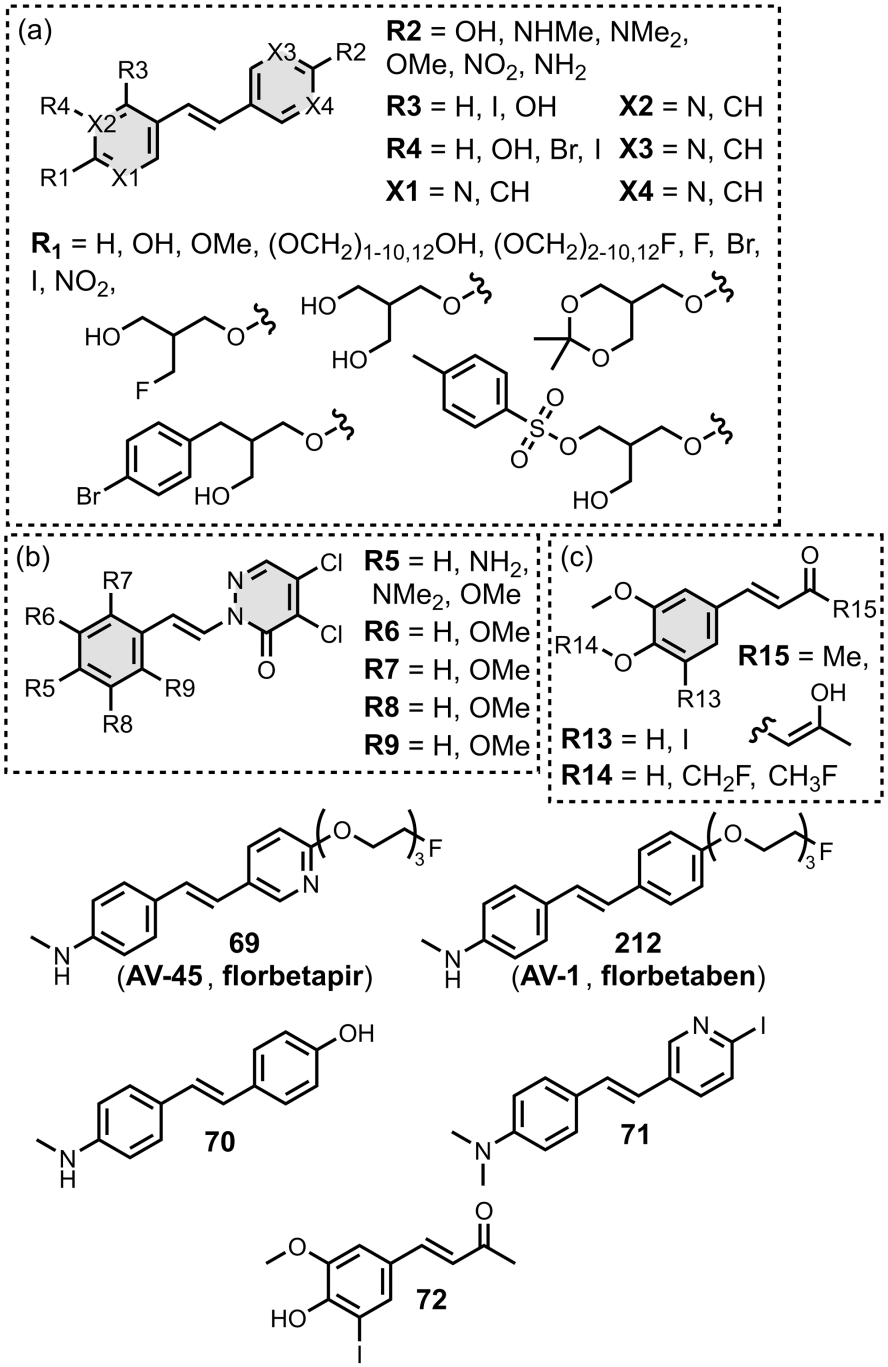
Stilbene ligands with substituents on the aryl rings.

The unsubstituted (*E*)-stilbene core has a high-nanomolar affinity (500 nM) for Aβ­(1-40). Installing electron donating amino or hydroxy substituents on the para position of one of the two aryl rings (R1 or R2 in Figure 52a) improved the binding affinity for Aβ­(1-40) and AD brain homogenates, although methylation of these substituents was essential for high affinities.^199^ Monomethylation of amine groups is preferred over dimethylation to avoid high lipophilicities, and **70** showed nanomolar affinities for AD brain homogenates (1–3 nM).^274,494,597^

If an electron donating substituent is installed at R2, large halides or PEG chains at R1 in Figure 52a afforded low nanomolar affinities for AD brain homogenates (<10 nM). Halides were also tolerated at R3 and R4. Installing a nitro group at R1 (*K =* 150 nM) or branched substituents (*K =* 15–150 nM) generally reduced binding affinity.^364^

Substituting one of the aryl rings in Figure 52a for pyridine reduced lipophilicity while maintaining high-affinity binding to AD brain homogenates (e.g. **71**, *K =* 3–7 nM).^474,503,548^ The FPEG derivative **AV-45** demonstrated good *in vivo* properties and showed a high affinity for AD brain homogenates (*K =* 3–4 nM). The ligand **AV-45** appeared to target Aβ rather than tau in AD.^211,492,494,608,641^ Iodo-substituted analogues were also reported as possible dual PET-SPECT imaging agents.^474^ Pyridazinones showed only micromolar affinities for Aβ­(1-40) (Figure 52b).^367^ Dehydrozingerone derivatives such as **72** were prepared resembling a simple styryl group, and displaced both **IMPY** and the *bis*styryl benzene **73** (commonly referred to as **IMSB**, see *bis*styryl benzenes section) (Figure 52c).^298^

Figure 53 summarizes stilbene derivatives prepared with extended aromatic π-systems and different linkers. The indole and quinoline analogues in Figure 53a yielded a range of nanomolar affinities for Aβ­(1-40) (*K =* 4-288 nM),^350^ with indole derivatives, such as **74**, giving the highest affinities. Pyrene substituents in Figure 53b were investigated and exhibited a large change in fluorescence upon binding, although only the salicylic derivatives **75** had a nanomolar affinity and targeted two sites (*K*_1_ = 48 nM, *K*_2_ = 360 nM) on Aβ­(1-42).^423,621^

**Figure 53:**
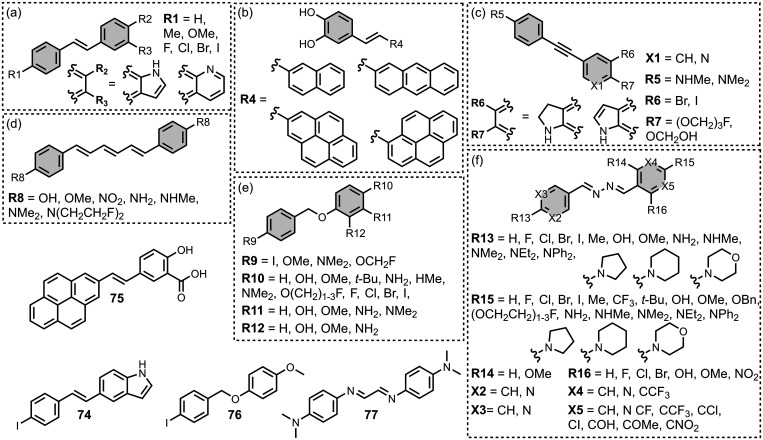
Stilbene ligands with extended aromatic π-systems and different linkers.

Different linkers have been explored in place of the trans-alkene core. The diphenylacetylene derivatives in Figure 53c have high binding affinities (*K =* 2–4 nM) for AD brain homogenates.^454,631^ Pyridine and indole phenylacetylene derivatives also showed high binding affinities (*K <* 20 nM) and promising *in vivo* properties in mice.^503,577^ While stilbenes compete with **IMPY** but not **IMSB**, the biphenyltrienes in Figure 53d displaced both **IMPY** (*K <* 1 nM) and **IMSB** (*K <* 122 nM) in AD brain homogenates,^597^ which shows that extending the linker allows different binding sites to be targeted. For these ligands, the dimethylamine and methylhydroxy derivatives at R8 showed worse affinity (*K >* 7,000 nM) than the monomethylated or *des*methylated counterparts (*K =* 8–9 nM).

Flexible ether linkers were also prepared (Figure 53e), with **76** (commonly known as **BOB-4**) showing nanomolar affinities for Aβ­(1-42) (*K =* 0.6-24 nM).^495,496,505^ The asymmetry imparted by the ether linker did not affect the binding affinity of the different isomers but did affect the rate of metabolism in mice models. A number of diaryl azines were also tested with Aβ­(1-42) in a thorough SAR study (Figure 53f and 77), affording ligands with a range of dissociation constants (*K* = 0.5 to >1,000 nM).^374^

Lastly, a multivalent strategy was employed using the stilbene ligands shown in Figure 54.^203,223,444^ Of the ligands shown in Figure 54a, the dimer **78** showed the highest affinity for AD brain homogenates (*K =* 3 nM) although this affinity is comparable to those of many of the monomeric stilbene ligands. The dimer **78** showed relatively poor penetration across the BBB but did label cerebral amyloid angiopathy in brain sections. In Figure 54b, the affinity of stilbene dimers with a PEG_n_ linker to AD brain homogenates generally increased with linker length, whereas monomeric ligands attached to longer PEG_n_ chains showed no increase in affinity.^203^ A similar trend was observed for divalent stilbazolium ligands (Figure 54c), where the measured dissociation constant for Aβ­(1-42) improved from micromolar binding (*K* = 1,900–4,600 nM) to nanomolar binding (*K* = 130–300 nM) with a longer alkane linker.^223^

**Figure 54:**
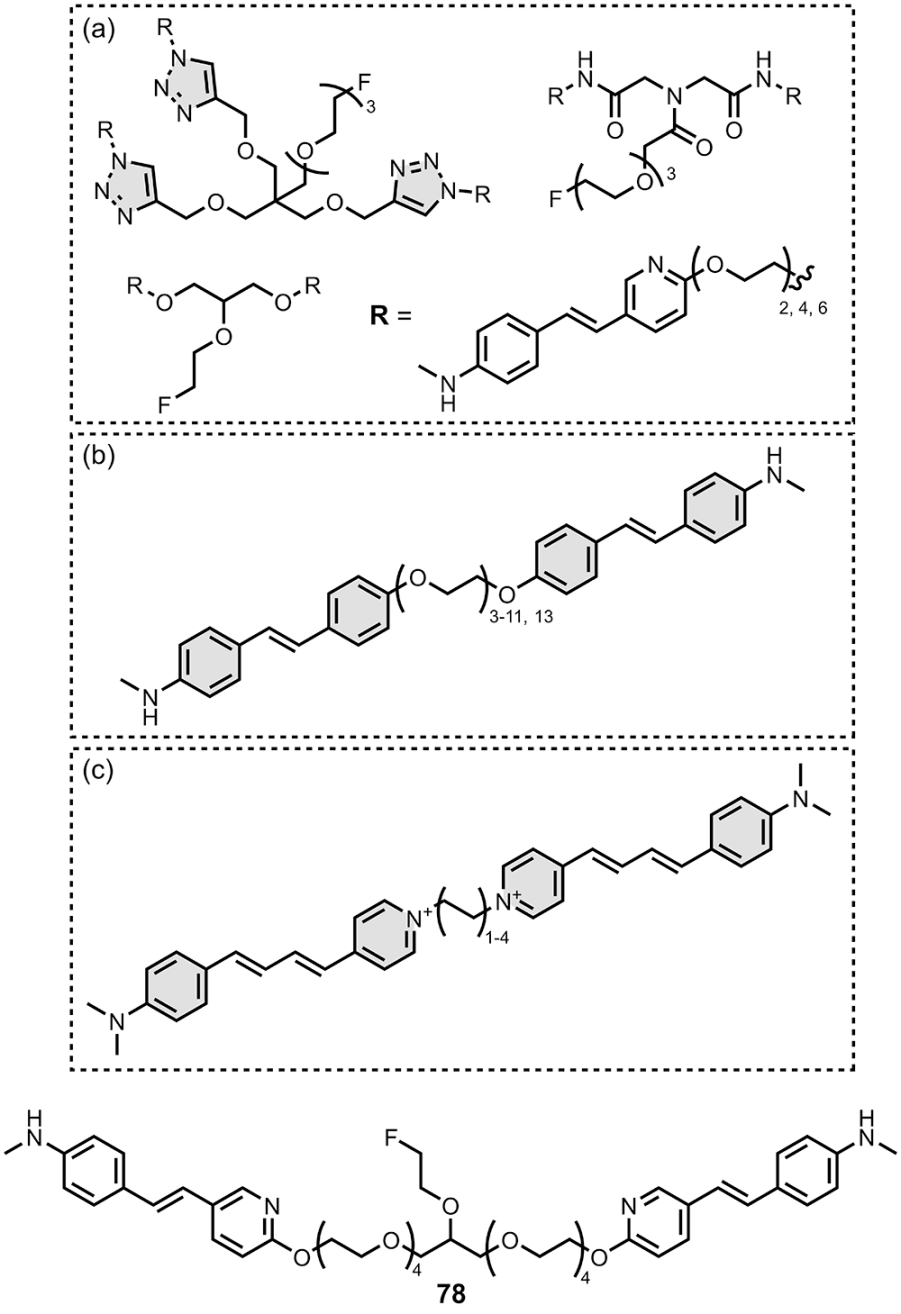
Dimeric stilbene ligands.

In summary, methylated amino and hydroxy substituents were preferred on the aromatic rings of stilbenes, whereas bulky and electron withdrawing groups reduced affinity. Pyridine rings and FPEG substituents are well tolerated and reduce lipophilicity. The different linkers studied all produced nanomolar ligands. Multivalency was explored but did not provide a clear enhancement in affinity.

## 12 Congo Red Derivatives

Congo red (**CR**, **68**) was one of the first ligands used for the detection of amyloid fibrils alongside **ThT**.^296,666^ Several derivatives of **CR** have been prepared showing micromolar to nanomolar dissociation constants for Aβ (Figure 55). Figure 56 summarizes the structures of **CR** derivatives. **CR** yields apple-green birefringence upon amyloid binding, but it’s poor blood–brain barrier (BBB) permeability prohibits *in vivo* applications.^666^ **CR** also targets multiple binding sites on Aβ; direct fluorescence binding assays using **CR** give micromolar affinities (1,100–1,500 nM) whereas competition binding assays give a wide range of affinities (7–560 nM or >1,000 nM), suggesting that the different assay formats report on binding to different sites. Derivatives of **CR** have been synthesized to improve BBB permeability and binding affinity. The salicylic acid derivative **79** (commonly known as **Chrysamine G**, or **CG**) was an early analogue that showed binding in brain tissue from both healthy individuals and AD patients.^290^ Both **CR** and **CG** appear to target at least two sites on amyloid fibrils, not all of which produce a change in fluorescence properties.^187,290,310,546^ Other substituents on the aromatic rings, or the inclusion of an amide linker and different cores (**80**, **81**, **82**), did not improve binding affinities.^195,329,333,604,606^

**Figure 55:**
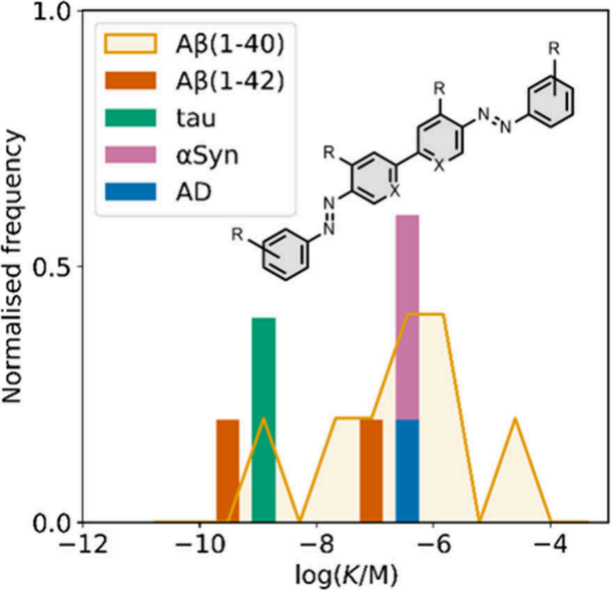
Distribution of binding affinities for the interaction of 8 **CR** derivatives with amyloids: Aβ­(1-40) (solid yellow line, 8 ligands), Aβ­(1-42) (orange bar, 2 ligands), tau (green bars, 2 ligands), αSyn (purple bar, 2 ligands) and AD brain homogenates (blue bar, 1 ligands). Bars are used to represent three or fewer ligands. For ligands where several binding affinities have been reported, the average value was used.

**Figure 56:**
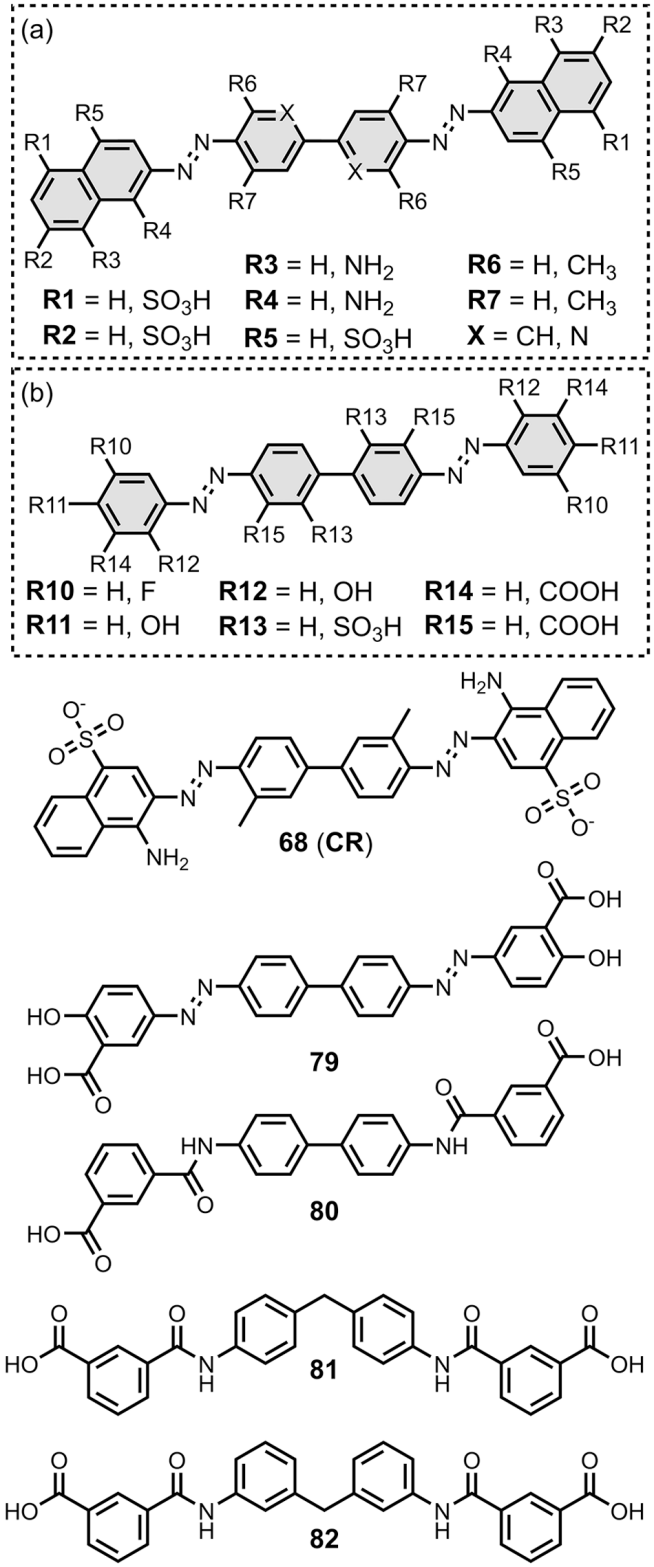
Congo Red derivatives.

## 13 *Bis*styryl Benzenes

Figure 57 shows the measured binding affinities for *bis*styryl benzene derivatives. Some low- and sub-nanomolar ligands have been reported for tau and Aβ, whereas only relatively weaker affinities have been achieved for αSyn. Figure 58 shows the chemical structures of some of the *bis*styryl benzene derivatives explored, including analogues with nitrogen-containing aromatic cores. Figure 59 shows the derivatives prepared with other aromatic heterocyclic cores and spacer groups. *Bis*styryl benzenes were originally designed as Congo red analogues and have been extensively studied. Ligand **83** (commonly known as **X-34**, Figure 58a) was the first ligand reported from this class and stains a range of amyloid pathologies *ex vivo*.^667,668^

**Figure 57:**
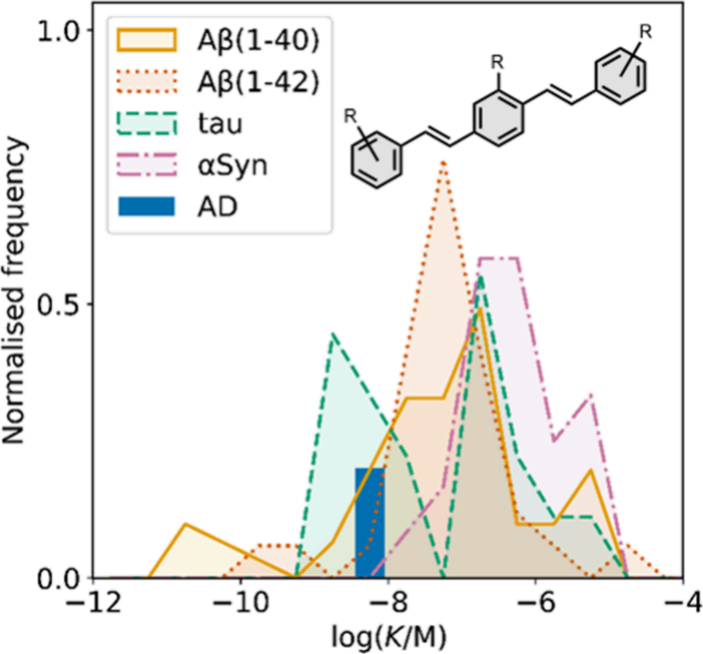
Distribution of binding affinities for the interaction of 101 *bis*styryl benzene ligands with amyloids: Aβ­(1-40) (solid yellow line, 61 ligands), Aβ­(1-42) (dotted orange line, 34 ligands), tau (dashed green line, 18 ligands), αSyn (dash-dot purple line, 24 ligands) and AD brain homogenates (blue bar, 1 ligands). Bars are used to represent three or fewer ligands. For ligands where several binding affinities have been reported, the average value was used.

**Figure 58:**
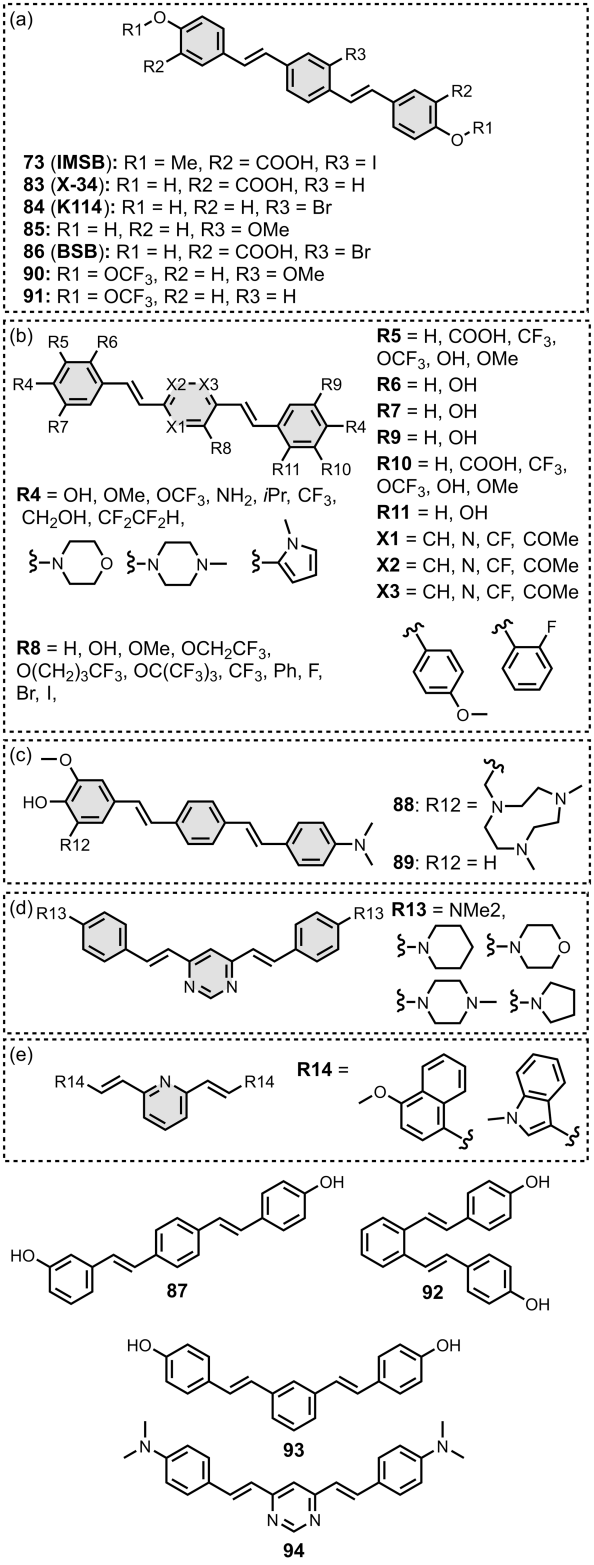
*Bis*styryl benzene ligands with substituents on the pendant aryl rings.

**Figure 59:**
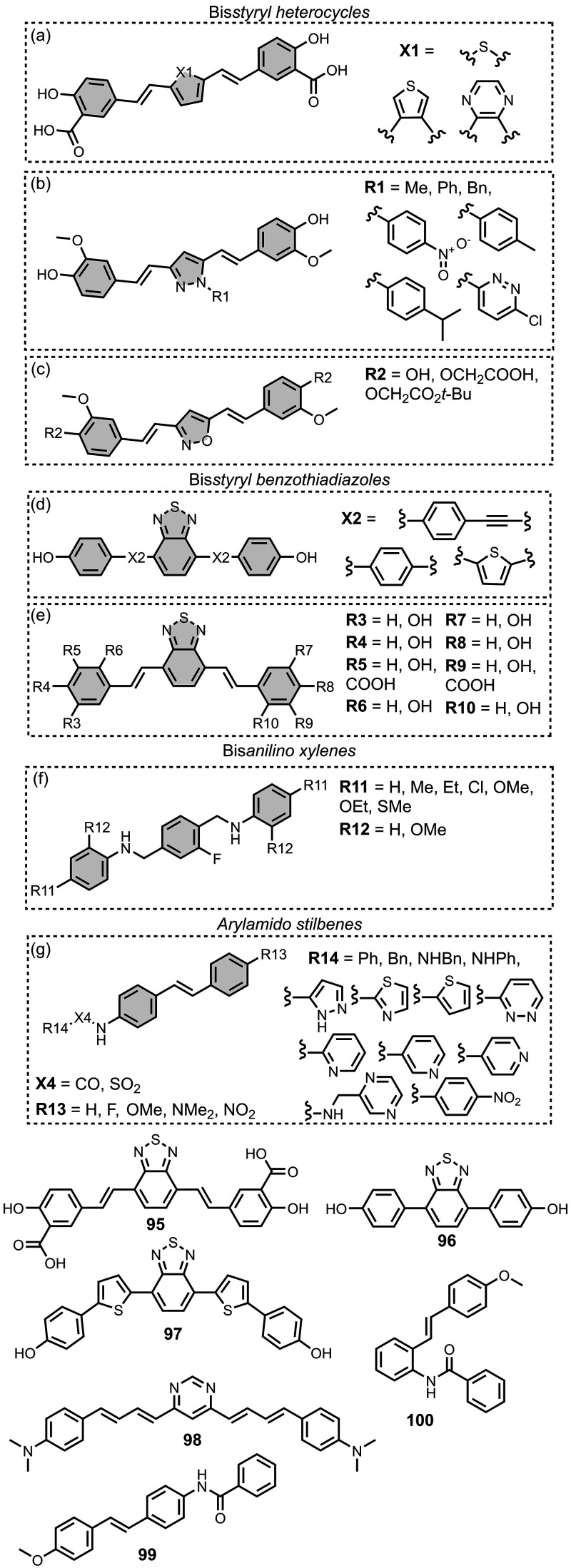
*Bis*styryl benzene ligands with different linkers and aromatic cores.

Several notable ligands have been reported from this structural class. Ligand **84** (commonly known as **K114**) was shown to bind to SPs, NFTs, and LBs and had promising fluorescent properties.^220,507^ Ligand **85** (commonly known as **Methoxy-X04**) produced a weaker fluorescence response but had improved brain penetration in transgenic mice.^394^ The ligand **73** (commonly known as **IMSB**) is commonly used in competition assays and shows a high affinity for Aβ­(1-40) and Aβ­(1-42) (*K =* 0.1–0.7 nM), and to Aβ aggregates in AD brain sections (*K =* 5.1 nM).^187,597^ **IMSB** was also shown to occupy a distinct set of binding sites to other structural classes of ligands, most notably fused benzoheterocycles.^274,461,494,506,536^ However, **86** (commonly known as **BSB**) can displace the charged fused benzoheterocycle **ThT** (*K =* 600 nM), albeit with a lower affinity than self-displacement (*K =* 0.1 nM).^304^ The details of the binding sites for *bis*styryl benzenes on Aβ are therefore not fully understood.^137^

On the pendant aromatic rings of *bis*styrylbenzenes, carboxyl groups at R5 and R10 in Figure 58b were featured on several high affinity Aβ-ligands. Derivatives with identical hydroxy derivatives at R4 also led to high affinity ligands (*K <* 1 nM), with methylation and fluoroalkyl substituents on a hydroxyl group at R4 having little effect on affinity. Asymmetric substituents (i.e. different substituents on each pendant ring at R5/R7 and R9/R10) or multiple hydroxyl groups on the pendant rings significantly reduced affinity (160–2,600 nM), such as **87** (*K =* 3,500 nM).^482,487^ However, incorporation of a pendant azamacrocycle on ligand **88** in Figure 58c produced relatively strong binding (*K* = 58 nM) compared to the unsubstituted derivative **89** (*K* = 10,900 nM).^247^

No consistent preference for substituents at an ortho (R6, R11), meta (R5, R7, R9, R10), or para (R4) position was observed.^482,483^ Additionally, the binding of ligands to fibrils formed *in vitro* did not always correlate with their binding to NFTs and SPs in biological samples.^488^

Methoxy and halide groups at R8 on the benzene core shown in Figure 58b significantly affected binding affinity. The bromo group in **BSB** improved affinities for Aβ­(1-40) and Aβ­(1-42) (*K =* 0.1–39 nM) compared to the unsubstituted analogue **X-34** (*K =* 6-299 nM). However, the methoxy group in **90** abolished binding (*K >* 1,000 nM) compared to the analogue **91** (*K =* 10 nM). Larger aryl substituents at R5 reduced affinity for tau (*K >* 600 nM) and Aβ­(1-40) (*K >* 2,000 nM), as did a second methoxy substituent at X1, X2, or X3, although one of the fluoroalkyl substituents explored was tolerated.^277,488^

Modifying the shape and size of the ligand was also explored. The 1,2- and 1,3-styryl substituted isomers **92** (*K =* 6,300 nM) and **93** (*K =* 2,500 nM) showed reduced binding affinity to Aβ­(1-40),^482^ although the different *E*/*Z* isomers of **BSB** all exhibited sub-nanomolar affinities (*K =* 0.1–0.3 nM).^424^ Different angular isomers were reported by Boländer *et al.*, who incorporated 2,5-substituted pyrazines (Figure 58b, X1 = X3 = N), 4,6-substituted pyrimidine (Figure 58d), and 3,6-substituted pyridazine cores (Figure 58b, X2 = X3 = N).^277,669^ The pyridazines showed the weakest affinities, whereas several pyrimidines and pyrazines were reported with low-nanomolar affinities for tau (*K =* 1-21 nM). The pyrimidine **94** exhibited a low nanomolar affinity for tau (*K =* 2 nM), with some selectivity over Aβ­(1-40) (*K =* 27 nM), and targeted tau in AD brain tissue. Some 2,6-subsituted pyridines (Figure 58e) were also reported but showed no binding (*K* > 10,000 nM).^544^

Figure 59 summarizes the *bis*styrylbenzene derivatives prepared with other aromatic heterocyclic cores and different spacer groups.^318,483^ Thiophene analogues exhibited relatively strong affinities for Aβ­(1-42) and tau (*K =* 13–16 nM) (Figure 59a). Pyrazole and isoxazole cores were reported with poor affinities for Aβ­(1-42) but were screened using an **IMPY** displacement assay which targets a different binding site to most *bis*styryl benzene derivatives (Figure 59b, c).^299^ The benzothiadiazole analogues in Figure 59d and Figure 59e exhibited mid nanomolar affinities (**95**, 93–110 nM) but promising fluorescence emission above 600 nm. Different benzothiadiazole derivatives also appeared to bind to different sites on fibrils.^483^

The spacer between the benzothiadiazole core and the pendant aromatic rings had a substantial effect on binding affinity and selectivity (X2 in Figure 59d). Removal of the alkene spacer in **96** reduced binding affinity for both Aβ­(1-40) (*K =* 250 nM) and Aβ­(1-42) (*K =* 510 nM), while a phenylacetylene spacer improved affinity (*K =* 36–59 nM).^483^ A thiophene spacer (**97**) enhanced binding selectivity between Aβ­(1-40) (*K =* 10 nM) and Aβ­(1-42) (*K >* 400 nM). A longer dialkene linker (**98**) improved affinity to Aβ­(1-40) (*K =* 1 nM) compared to the monoalkene analogue (*K =* 27 nM), but both had a similar affinity to tau (*K =* 1–2 nM).^277^ The flexible benzyldiamine linkers in Figure 59f produced high affinity ligands when screened using an **IMPY** displacement assay on Aβ­(1-42) (*K <* 6.8 nM), indicating this linker allowed these ligands to target fused benzoheterocycle binding sites.^207^

Asymmetric ligands containing one alkene linker and one amide or sulfonamide linker were screened with αSyn (Figure 59g).^339^ A 1,4-substitution of the linkers on the core yielded low affinity ligands (*K >* 10 μM, e.g. **99**) whereas the 1,2- and 1,3-substituted isomers demonstrated superior affinities (*K =* 40–490 nM, e.g. **100**). This result differs from a previous study that observed poor binding affinities for the 1,2- and 1,3-styryl derivatives **92** and **93**.^482^ Different substituents explored at R13 in Figure 59g yielded broadly similar affinities. Of the heterocycles explored at R14, 2-pyridines produced some of the highest-affinity ligands (*K =* 10–100 nM).

In summary, the overall shape and size of *bis*styryl benzene ligands has a pronounced effect on binding affinity. On the pendant aromatic rings, monosubstitution with electron donating *O*- and *N*-groups improved the affinity for Aβ, and the location of the substituent influenced binding selectivity. Polyfluorination of the benzene core, or replacement with an aromatic heterocycle, significantly influenced binding affinity and could produce favorable fluorescence properties. Substituents on the core, and the nature of the linker used, also influenced binding, but no clear SAR has been determined.

## 14 Curcumin Derivatives

Curcumin derivatives have predominantly been screened with Aβ, with some selectivity toward Aβ­(1-42) over Aβ­(1-40) as shown in Figure 60. Figure 61 shows the structures of the curcumin derivatives that have been prepared. **Curcumin** (**101**) has structural similarities to *bis*styryl benzenes derivatives, bearing two styryl groups connected by a conjugated core. **Curcumin** inhibits Aβ aggregation and competes with both **IMSB** (*K =* 4 nM) and **IMPY** (*K =* 110 nM) on Aβ­(1-42).^298,670^ Several difluoroboron-curcumin derivatives have also been prepared and are summarized in the boron-complexed dyes section.

**Figure 60:**
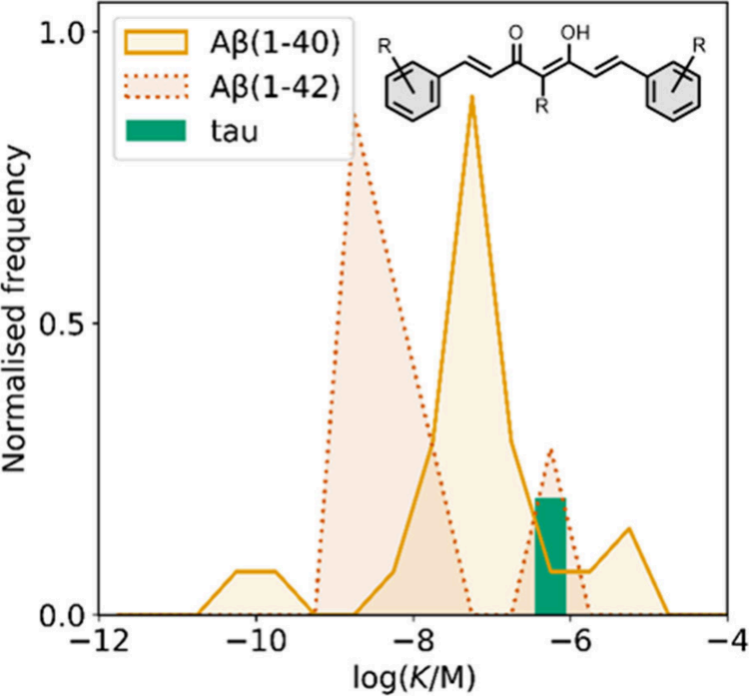
Distribution of binding affinities for the interaction of 34 curcumin ligands with amyloids: Aβ­(1-40) (solid yellow line, 27 ligands), Aβ­(1-42) (dotted orange line, 7 ligands), and tau (green bar, 1 ligand). Bars are used to represent three or fewer ligands. For ligands where several binding affinities have been reported, the average value was used.

**Figure 61:**
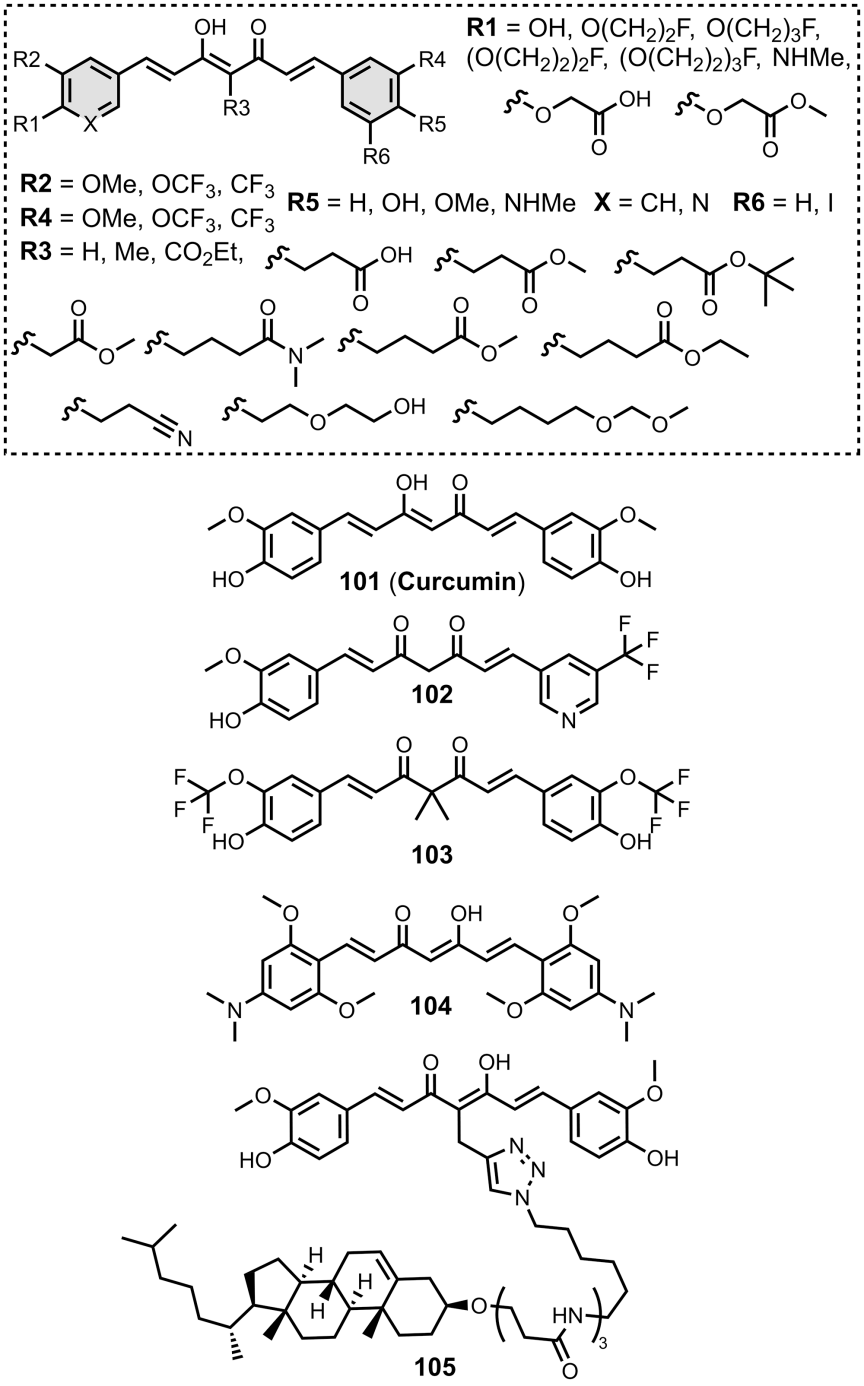
Curcumin ligands.

On a curcumin core, fluoroalkyl or FPEG substituents at R1 and R5 maintained high affinity binding for Aβ (*K =* 0.1–5 nM), whereas an iodo substituent at R6 reduced the affinity (9 nM).^425,562^ Several other curcumin derivatives were prepared, and although no clear SAR was obtained, **102** exhibited nanomolar affinities for Aβ­(1-40) (*K =* 16 nM).^627^ Notably the diketone **103** showed no measurable binding. The derivative **104** showed modest affinities for tau (*K =* 770 nM) and, upon binding, has a red-shifted emission at 620 nm and a 23-fold enhancement in quantum yield.^478^ A cholesterol conjugate of curcumin, **105**, was reported, but only micromolar binding was achieved.^279,291^

## 15 *Bis*styryl Acetones

*Bis*styryl acetones bind Aβ with nanomolar affinities and show some selectivity for synthetic over biological fibrils (Figure 62).

**Figure 62:**
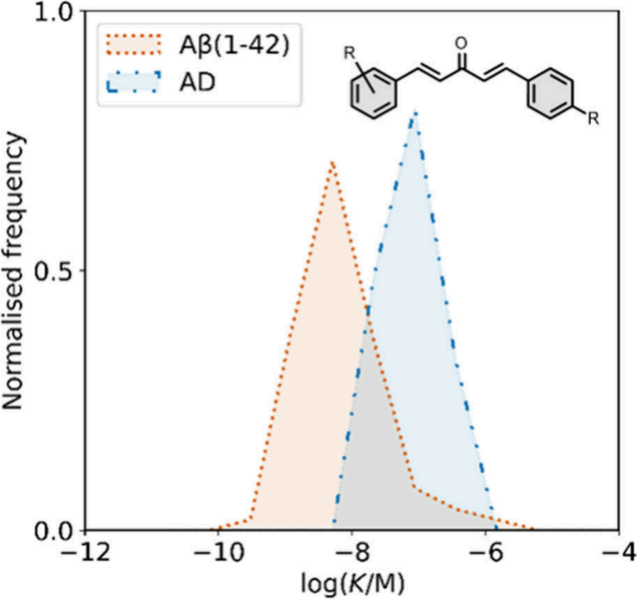
Distribution of binding affinities for the interaction of 88 *bis*styryl acetone ligands with amyloids: Aβ­(1-42) (dotted orange line, 80 ligands), and AD brain homogenates (dash-dot-dot blue line, 10 ligands). For ligands where several binding affinities have been reported, the average value was used.

The structures of the *bis*styryl acetone analogues are summarized in Figure 63. These derivatives resemble curcumin with a structural core that cannot tautomerize. A large SAR study of this class of ligands was performed by Cui *et al*.^592^ The highest affinity ligands for Aβ­(1-42) tended to have substituents at R1 and R6 on the pendant aryl rings in Figure 63a (e.g. **106**). Hydroxyl and amino groups at R1 and R6 most frequently led to high affinity binding, although halogen, alkyl, and nitro groups were well tolerated (*K <* 10 nM). Substituents at R2 and R5 also allowed for nanomolar binding, whereas substituents at R3 and R4 could drastically reduce binding affinity (e.g. **107**, *K =* 2,600 nM). The steric tolerance of substituents at R1 was unclear, as diethylamino or diphenylamino substituents reduced affinity (*K =* 16–130 nM) whereas a benzoxyl or tritylmethoxy substituent retained high-affinity binding (2–3 nM). Replacing one of the pendant aromatic rings with an aromatic heterocycle or naphthalenyl group was generally well tolerated (Figure 63b, c). Incorporating a cyclohexanone core (Figure 63d) tended to reduce binding affinity, although high affinity ligands such as **108** were still achieved (*K =* 7 nM).

**Figure 63:**
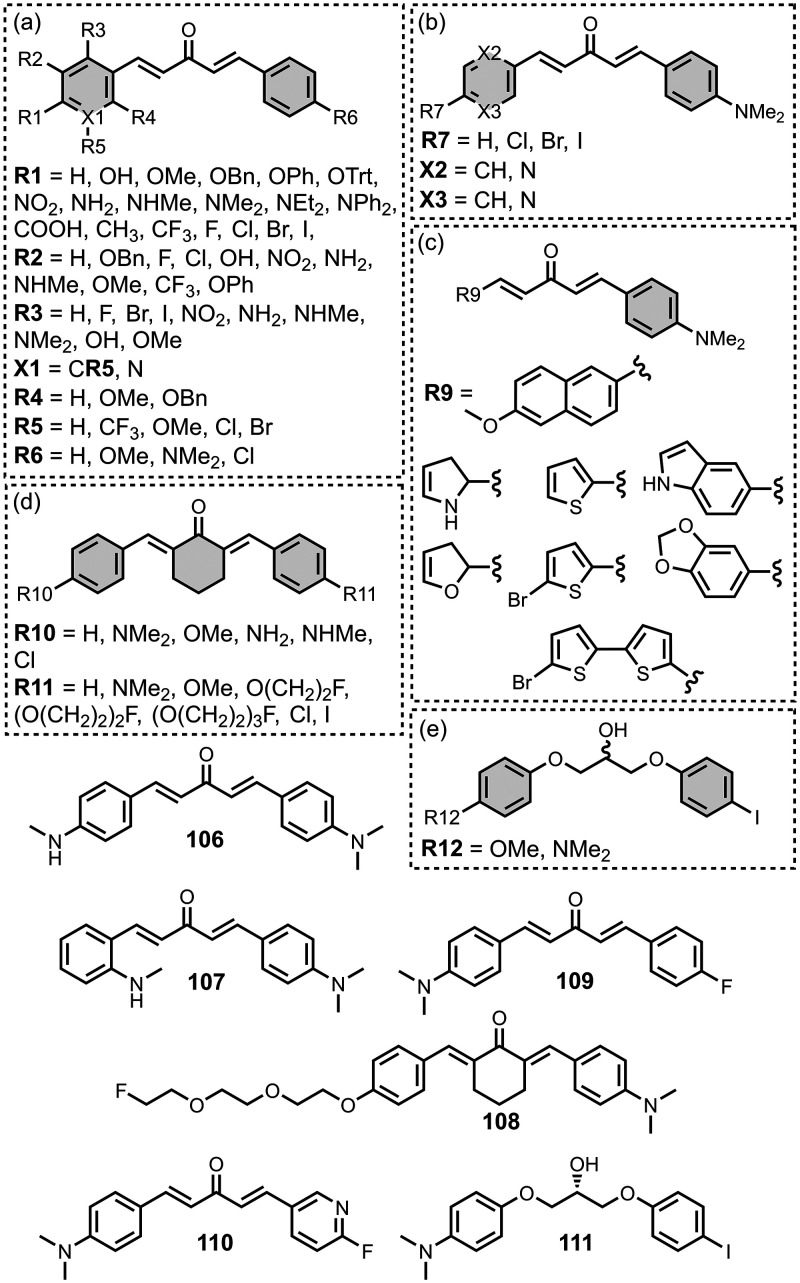
*Bis*styryl acetone ligands.

A later study investigated [^18^F]-labelled dibenzylidene acetone derivatives (Figure 63e).^460^ **109** exhibited the highest affinity for Aβ­(1-42) (*K =* 3 nM). Incorporating pyridine rings, such as in **110**, maintained high binding affinities (*K =* 9 nM). Enantiopure ligands with a glycerol core were studied (eg. **111**), but both enantiomers had similar affinities for Aβ­(1-42) (*K =* 16–45 nM).^477^

## 16 Chalcones

Chalcones were studied as amyloid ligands based on the ability of flavones and chromanones to bind amyloids. Chalcones have been primarily screened with Aβ and αSyn, with the measured binding affinities summarized in Figure 64. Figure 65 summarizes the chemical structures of the chalcone derivatives prepared. For chalcones bearing two phenyl rings (Figure 65a), electron-donating groups without a H-bond donor were favored at R1 and R2.^376,464,536,576^ Methylation of amine or hydroxyl substituents at these positions was essential for strong binding, with the dimethylamino derivative **112** (commonly known as **DMIC**) exhibiting a significantly higher affinity for Aβ­(1-42) (*K =* 3–4 nM) than the *des*methyl analogue (*K =* 100 nM). With an electron donating group at one of R1 or R2, a second electron donating group at the other position reduced the binding affinity (*K >* 20 nM) whereas a hydrophobic substituent was favored.^376^ Polyhydroxylated derivatives were studied as αSyn ligands but showed lower affinities (*K >* 900 nM) (Figure 65b).^628^

**Figure 64:**
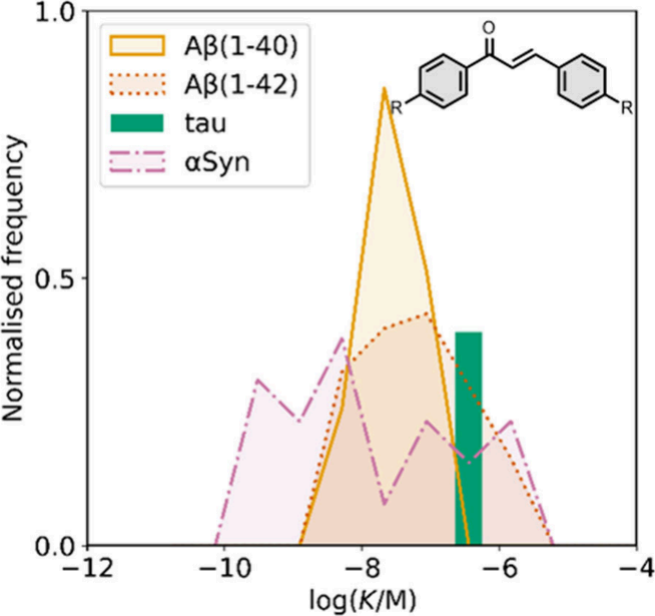
Distribution of binding affinities for the interaction of 94 chalcone ligands with amyloids: Aβ­(1-40) (solid yellow line, 19 ligands), Aβ­(1-42) (dotted orange line, 60 ligands), tau (green bar, 2 ligands), and αSyn (dash-dot purple line, 21 ligands). Bars are used to represent three or fewer ligands. For ligands where several binding affinities have been reported, the average value was used.

**Figure 65:**
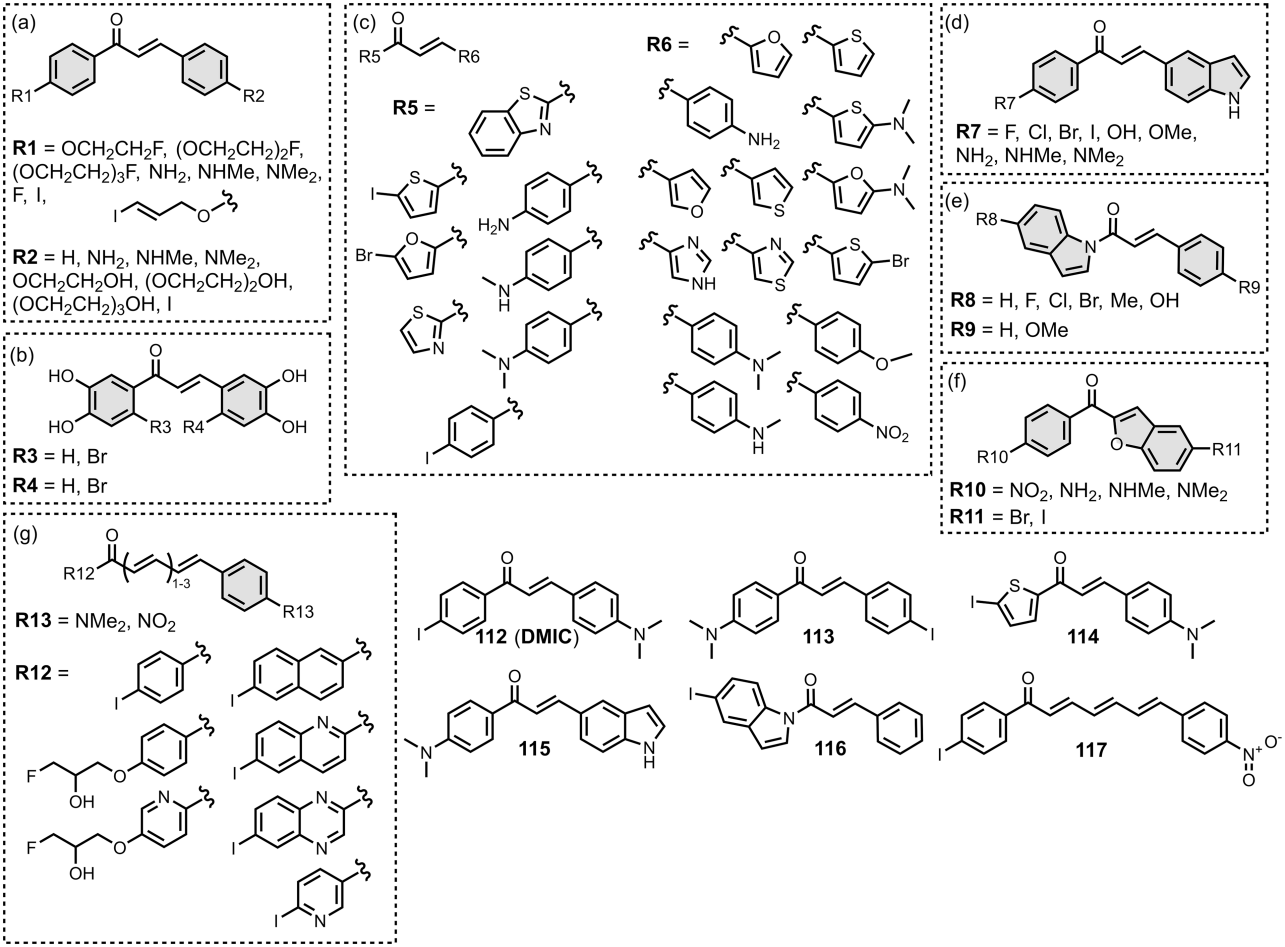
Chalcone ligands.

The arrangement of substituents on R1 and R2 in Figure 65a was important, as the structural isomer of **DMIC**, **113**, exhibited weaker binding (*K =* 13 nM).^536^ This effect was more pronounced for analogues with pendant aromatic heterocycles (Figure 65c–f). Aromatic heterocycles at R5 and R6 in Figure 65c generally led to high nanomolar binding affinities (*K >* 100 nM) with some exceptions, such as a thiophene substituent at R5 as in **114** (*K =* 4 nM), an indole substituent at R5 (Figure 65d), or an indole substituent at R6 (Figure 65e).^284,466,536,552^ For an indole ring at R6 in Figure 65d, methylation of hydroxyl or amine groups at R7 was essential as in **115** (*K =* 5 nM), as the *des*methyl analogues had no measurable binding.^552^ Further evaluation of an indole ring at R5 in Figure 65e revealed that functionalization at R8 with halogen substituents, such as **116**, was most favored (*K =* 8 nM).^466^ Masking the alkene linker as a benzofuran in Figure 65f also led to high-affinity ligands with halogen substituents at R11 and methylated electron-donating groups at R10 favored (*K =* 7–40 nM).^573^

Extended polyene linkers were investigated and assayed with both Aβ­(1-42) and αSyn (Figure 65g).^283,539,571^ Increasing the length of the linker improved selectivity for binding to αSyn up to approximately an order of magnitude. A range of aromatic rings at R12 were well tolerated, and **117** was one of the highest affinity ligands prepared. **117** bound to αSyn (*K =* 0.5–7 nM) selectivity over Aβ­(1-42) (*K =* 100 nM).

## 17 *Bis*aryl Heterocycles

Most binding studies on *bis*aryl heterocycles were performed with Aβ­(1-42) or AD brain homogenates. The measured binding affinities are summarized in Figure 66. Figure 67 provides an overview of the *bis*aryl heterocycle analogues that have been prepared. *Bis*aryl heterocycles resemble chalcones with the central carbonyl and alkene masked as an aromatic heterocycle. For a thiophene core (Figure 67a), electron donating substituents at R1 and R2 on the pendant aromatic rings generally gave good affinities for AD brain homogenates (*K =* 4–30 nM).^314^ In contrast, substituents at R3 led to lower affinity ligands (**118**, *K =* 630 nM). Incorporating a single electron withdrawing nitro group at R2 retained a nanomolar dissociation constant (**119**, *K =* 19 nM), whereas nitro groups at both R1 and R2 significantly reduced binding affinity (190 nM). Other para-substituents without a hydrogen bond donor, such as in **120** (*K =* 500 nM), afforded lower-affinity binding.

**Figure 66:**
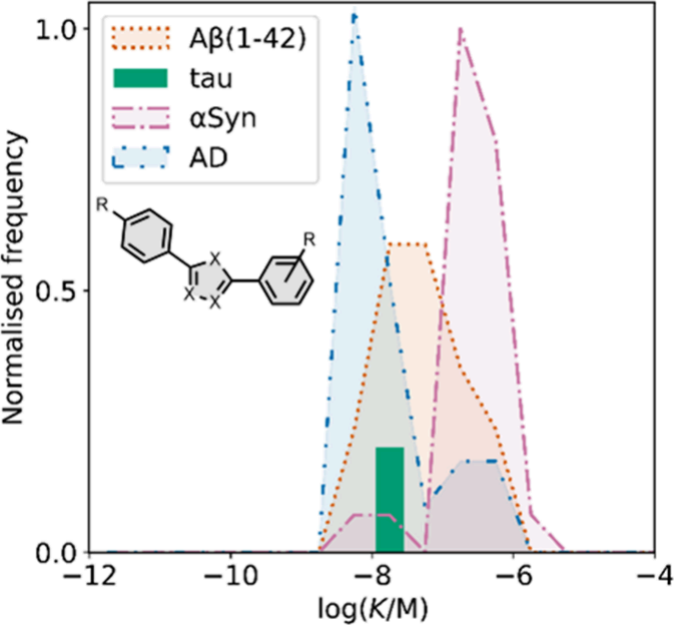
Distribution of binding affinities for the interaction of 65 *bis*aryl heterocycle ligands with amyloids: Aβ­(1-42) (dotted orange line, 17 ligands), tau (green bar, 1 ligand), αSyn (dash-dot purple line, 28 ligands) and AD brain homogenates (dash-dot-dotted blue line, 23 ligands). Bars are used to represent three or fewer ligands. For ligands where several binding affinities have been reported, the average value was used.

**Figure 67:**
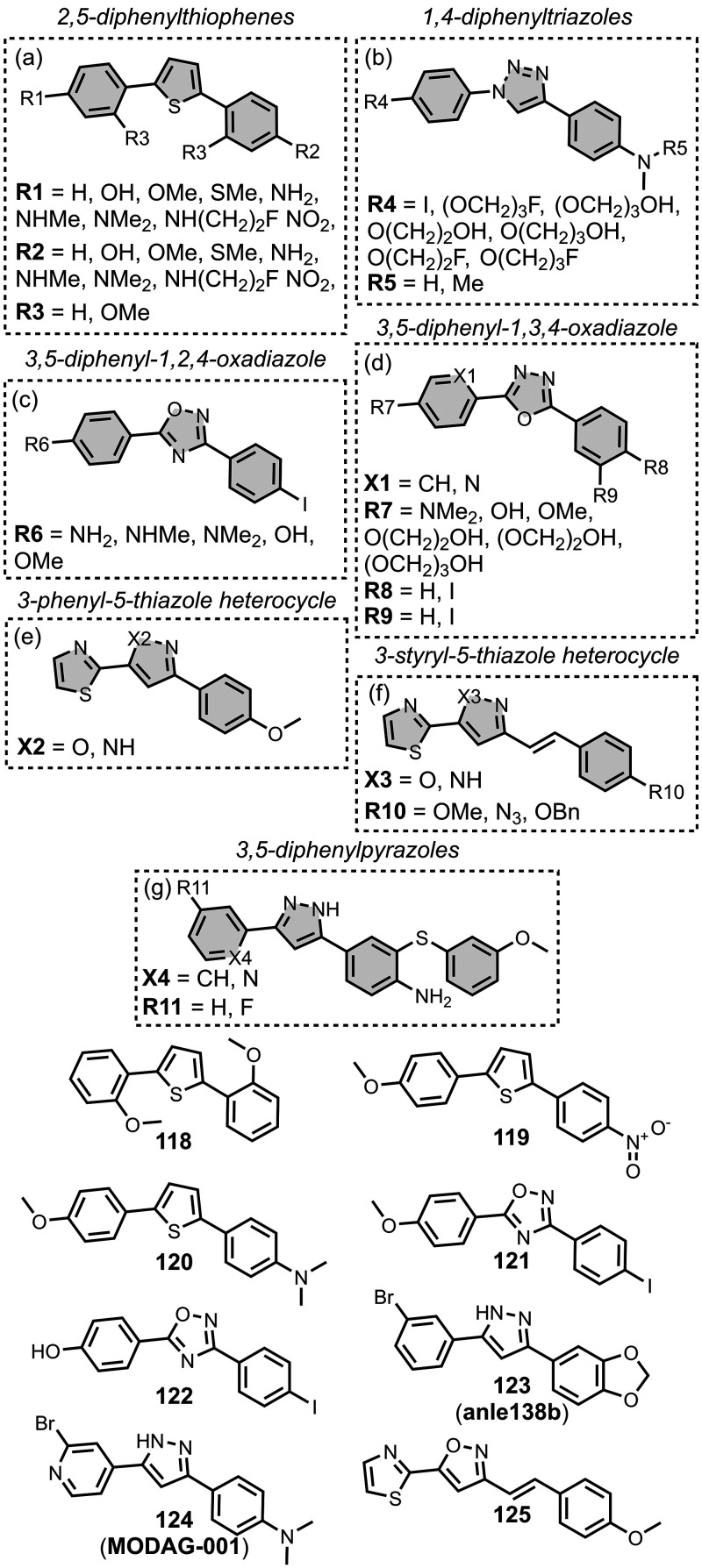
*Bis*aryl heterocycle ligands.

In order to reduce lipophilicity, the 1,4-diphenyltriazoles in Figure 67b were prepared and typically had good affinities for AD brain homogenates (*K =* 4–30 nM).^501^ A series of 3,5-diphenyl-1,2,4-oxadiazole (1,2,4-DPOD) analogues were prepared and showed nanomolar affinities for Aβ­(1-42) (Figure 67c) but significant nonspecific binding in transgenic mice.^327^ Less lipophilic 1,3,4-DPOD analogues were later synthesized to reduce nonspecific binding (Figure 67d) and displayed more promising *in vivo* properties despite poorer *in vitro* binding affinities.^471,557^ For these ligands, methylation of amino substituents did not significantly affect binding affinities (14–15 nM), whereas methylation of hydroxyl substituents was preferred (e.g. **121**, *K =* 4 nM; **122**, *K =* 47 nM).

Many of the derivatives were also screened with αSyn, but no clear SAR was apparent.^628^ The ligands **123** (commonly known as **anle138b**) and **124** (commonly known as **MODAG-001**) have subsequently been reported as promising αSyn ligands, with radiolabelled variants being tested as PET tracers.^198,201,259,671^

More recently, *bis*aryl heterocycles bearing a pendant thiazole were reported with selective binding to αSyn (*K =* 47–488 nM) over Aβ­(1-42) and tau (Figure 67e).^180,284^ Introducing an alkene linker to the pendant phenyl ring improved binding to both αSyn and Aβ­(1-42) (Figure 67f), whereas adding an additional aromatic ring led to no binding to αSyn (Figure 67g). The ligand **125** showed the most promising affinities for αSyn (*K =* 19 nM) and Aβ­(1-42) (*K =* 90 nM), but binding to tau was not investigated.

## 18 Oligothiophenes

The dissociation constants measured for oligothiophene derivatives are summarized in Figure 68. The chemical structures of the oligothiophene ligands are summarized in Figure 69. Oligothiophenes emerged as an early class of fluorescent amyloid probes with the report of **126** (commonly known as **NIAD-4**).^415^ Based on a push–pull architecture, **NIAD-4** demonstrates a large shift in λ_ex_ and an increase in fluorescence intensity upon binding to Aβ­(1-40). The derivative **127** (commonly known as **pFTAA**) stained AD sections in patterns that matched both Aβ and phosphorylated-tau antibodies.^468^ The spacing between anionic groups in oligothiophenes influences Aβ/tau selectivity.^342^ The neutral analogue **128** (commonly known as **pTP-TFE**) demonstrated improved selectivity for tau and appeared to bind only soluble tau aggregates.^640^ For a single thiophene ring, when conjugated to a malonitrile substituent via a polyene linker, the number of double bonds did not have a consistent effect on the binding affinity (Figure 69).^410^ Other oligothiophene ligands have been reported, but the binding affinities were not measured.^433,672^

**Figure 68:**
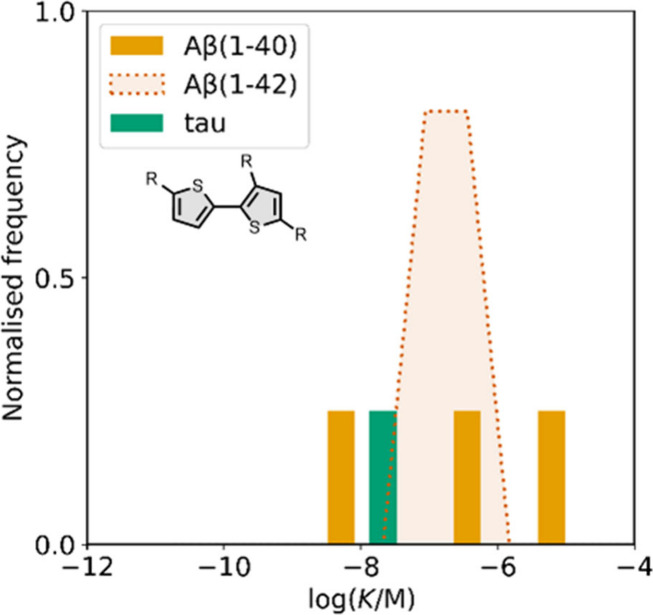
Distribution of binding affinities for the interaction of 7 oligothiophene ligands with amyloids: Aβ­(1-40) (yellow bars, 3 ligands), Aβ­(1-42) (dotted orange line, 4 ligands), and tau (green bar, 1 ligand). Bars are used to represent three or fewer ligands. For ligands where several binding affinities have been reported, the average value was used.

**Figure 69:**
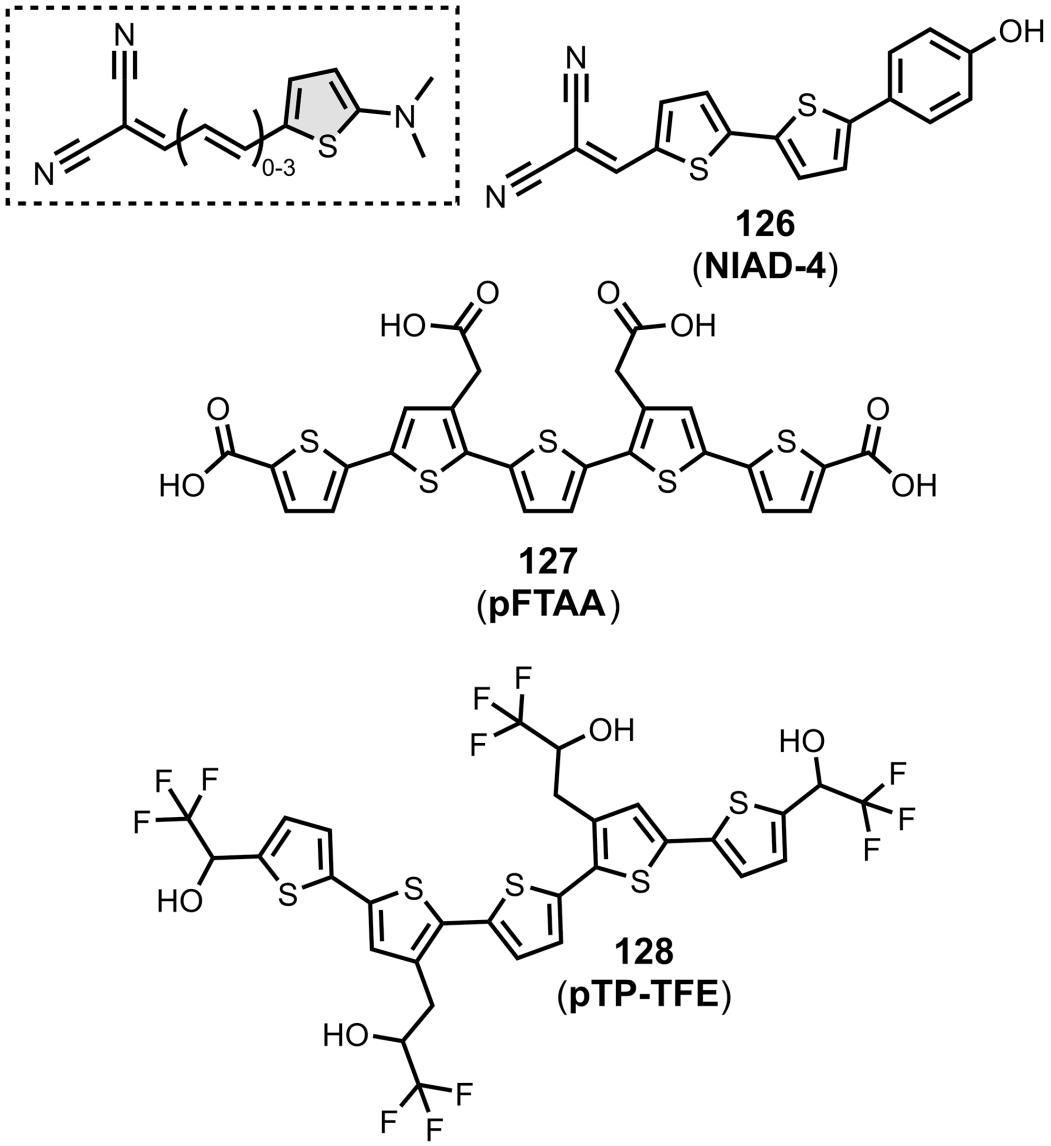
Oligothiophene ligands.

## 19 Aminoaryl

Many aminoaryl derivatives have been reported with a range of binding affinities, as summarized in Figure 70. The structures of the aminoaryl ligands are summarized in Figure 71 and Figure 72. **129** (commonly known as **FDDNP**) was the first amyloid ligand reported with an aminoaryl core.^479,673^ The ligand **FDDNP** binds to tau (*K =* 12–260 nM) and Aβ (*K =* 0.1–75 nM) fibrils formed *in vitro* with good affinity.^194,270,304,411,639^ **FDDNP** may bind to two or more sites on Aβ, as the binding affinity differs when measured by fluorescence and radioligand assays and when measured using different reporting ligands in competition assays.^194,270,285,304,309,332,411,421,597^ **FDDNP** also binds to AD brain homogenates (*K =* 150–290 nM) and labels SPs, LBs, amyloid in CAA, and possibly NFTs. However, **FDDNP** does not label aggregates from PSP, PiD, or MSA.^421,605^ Like **ThT**, **FDDNP** contains an electron-donating aniline motif conjugated with an electron-acceptor motif via a polyene linker. Many analogues have since been prepared by modifying these motifs and the conjugated linker joining them.

**Figure 70:**
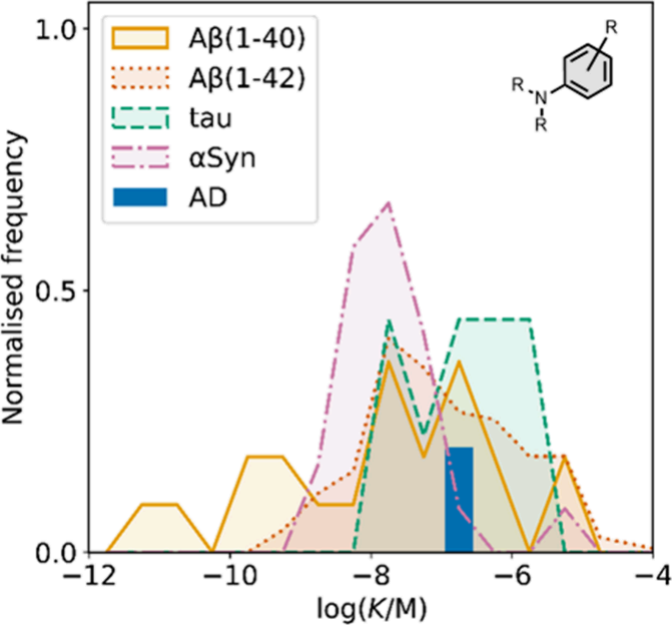
Distribution of binding affinities for the interaction of 159 aminoaryl ligands with amyloids: Aβ­(1-40) (solid yellow line, 22 ligands), Aβ­(1-42) (dotted orange line, 142 ligands), tau (dashed green line, 9 ligands), αSyn (dash-dot purple line, 24 ligands) and AD brain homogenates (blue bar, 1 ligand). Bars are used to represent three or fewer ligands. For ligands where several binding affinities have been reported, the average value was used.

**Figure 71:**
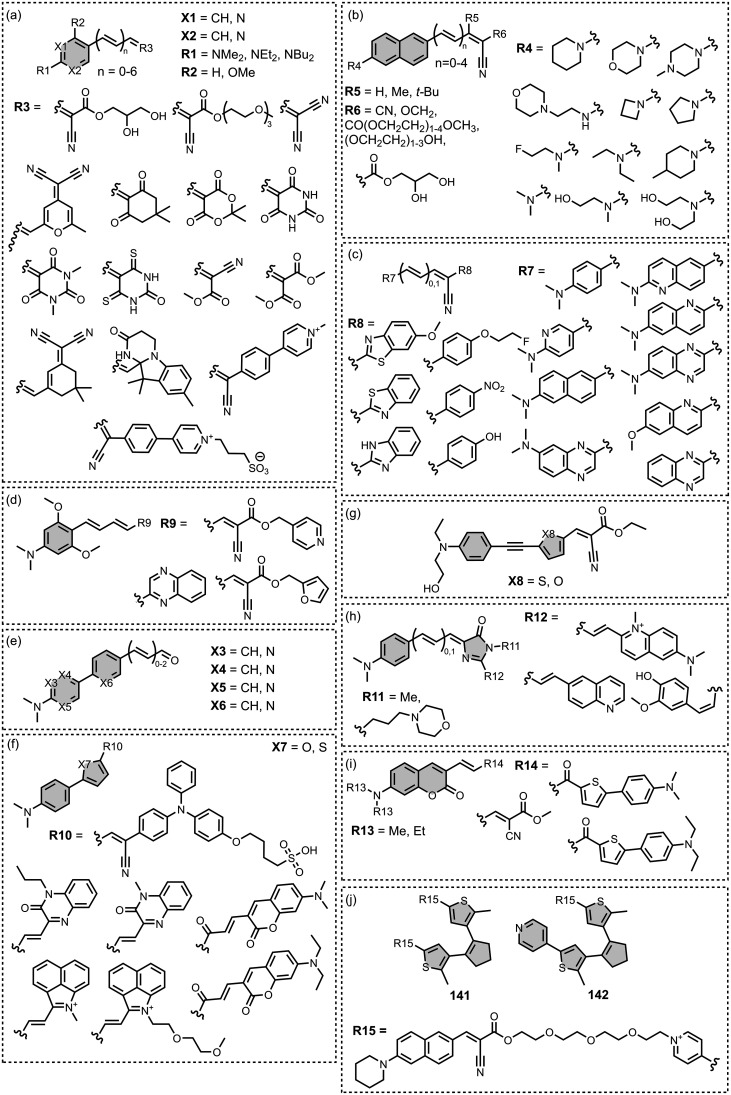
Aminoaryl ligands.

**Figure 72:**
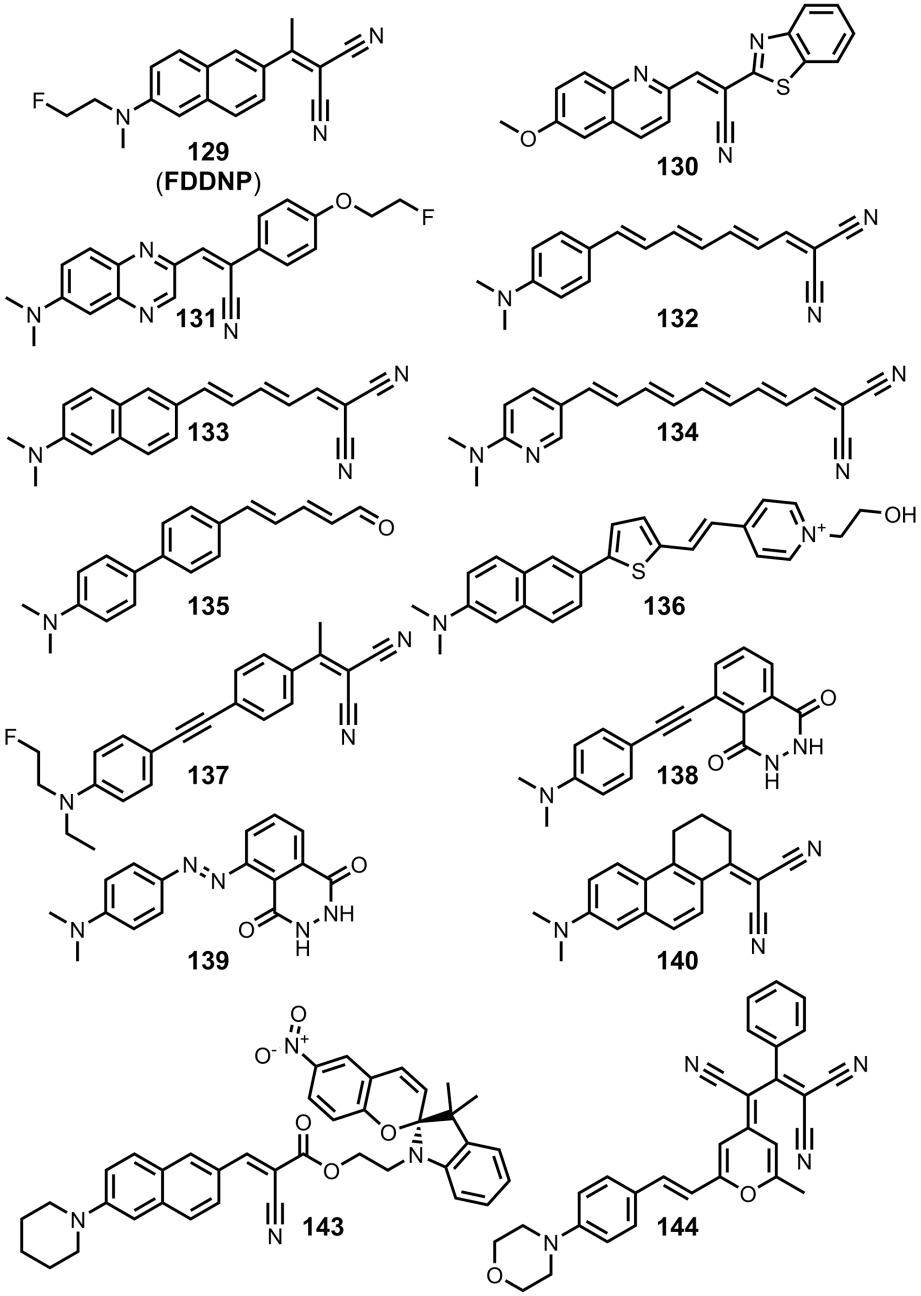
Specific aminoaryl ligands.

Aminoaryl ligands typically contain a phenyl or naphthyl ring bearing a dialkylated amino group (Figure 71), although a range of other aromatic nitrogen heterocycles have been incorporated (Figure 71a–c).^251,258,332,512^ In Figure 71a, a methoxy substituent at R2 did not affect the binding,^239,512^ while dimethoxy derivatives bound weakly to tau and Aβ­(1-42) (*K =* 500–1,500 nM) (Figure 71d).^239^ Other aniline-based donors were also studied at R7 and afforded nanomolar dissociation constants (*K =* 0.4–90 nM). When the dimethylamino group of an aniline was replaced by a methoxy group, such as in **130**, weaker binding affinities were observed for αSyn (*K* not measurable) and Aβ­(1-42) (*K =* 120 nM).^302^ The aminoquinoxaline **131** was one of the most successful derivatives with nanomolar binding to αSyn (*K =* 6 nM) and Aβ­(1-42) (*K =* 2 nM).

The length of the polyene linker in Figure 71a–c affected the binding affinity, with longer polyene linkers generally improving affinity up to a certain length.^251,360,387,529,530,541,612^ A longer polyene linker was favored for phenyl derivatives (Figure 71a) compared to naphthyl derivatives (Figure 71c), as conjugated systems with a similar total length yielded similar affinities (**132**: *K =* 14 nM; **133:** *K =* 2 nM).^360,530^ The optimal length of the polyene linker also increased with the number of nitrogen atoms incorporated into the ring bearing the aniline nitrogen substituent, suggesting there is an optimal hydrophobicity for binding (**132**: *K =* 14 nM; **134:** *K =* 27 nM).^529^ Aminoaryl derivatives are also able to bind to at least two sites, and the selectivity between these sites is tuned by the length of the linker.^251,360,387,529,530,541,612^

Several other types of linker connecting an electron-donating aminoaryl group and an electron-acceptor motif were investigated. Several biaryl systems with a phenyl linker and a conjugated aldehyde were studied (Figure 71e).^513^ Incorporating aromatic nitrogen heterocycles generally decreased affinity, and a single double bond was typically required to produce nanomolar-affinity ligands (*K =* 4–340 nM). The only exception to this was **135**, which also exhibited the highest binding affinity for Aβ­(1-42) (*K =* 2.5 nM). Several ligands with thiophene or furan linkers were also prepared (Figure 71f, **136**). Derivatives with coumarin or triphenylamino electron-acceptor motifs showed micromolar affinities for Aβ­(1-42) and tau (*K =* 1,400–59,000 nM), whereas the tricyclic and quinoxaline derivatives exhibited nanomolar affinities for Aβ (*K =* 17–380 nM) (Figure 71f).^254,260,308,515^ Linkers featuring an alkyne group (Figure 71g, **137**) were reported but bound relatively weakly (*K* = 150–5,500 nM), except for **138**, which selectively targeted αSyn (*K* = 6.6 nM) over Aβ­(1-42) (*K* = 80 nM) and tau (*K* = 322 nM).^292,309,465,513^ Similar selectivity was seen for the diazo derivative **139**.

A range of different electron-acceptor motifs have been studied (Figure 71a–d).^239,360,612,639^ At R3 in Figure 71a malononitrile, methyl cyanoacetate, and methylketone acceptors all produced binding affinities below 100 nM.^270,332,409^ Various PEG polymers were explored at R6 in Figure 71b and generally did not affect the binding affinity.^512^ However, when longer polyene linkers were used in Figure 71b, there was a small preference for longer PEG chains at R6, possibly to maintain an optimum hydrophobicity.^541^ Substituents at R5 did not significantly affect the measured affinity, but the tricyclic ligand **140** showed particularly strong binding (*K <* 1 nM).^194,332^ Using aryl rings as an electron-acceptor motif at R8 in Figure 71c also led to nanomolar affinities for both αSyn and Aβ­(1-42) (*K =* 0.4–120 nM), although no more than one order of magnitude selectivity was achieved.^302^ The imidazolones in Figure 71h have also been studied as the electron-acceptor motif and gave high nanomolar dissociation constants for Aβ­(1-42) (*K* = 190–290 nM).^232,326,381^ Coumarin derivatives in Figure 71i gave a range of affinities, although thiophene substituents at R14 significantly weakened binding for Aβ­(1-42) (*K* = 24,600–59,300 nM) (Figure 71j).^260,644^

Several additional ligands have been prepared. The dimeric ligand **141** was reported, although this ligand had a similar binding affinity for Aβ­(1-42) aggregates (*K =* 1.1 μM) as the monomer **142** (*K =* 1.3 μM).^330^ Spiropyran ligand **143** preferentially interacted with Aβ oligomers (*K =* 1.7 μM) over monomers or fibrils.^240^ The ligand **144** bound to Aβ­(1-42) with nanomolar dissociation constants (*K* = 44–140 nM) and emitted NIR light.^516^

## 20 Quinolines

Quinolines are an important class of tau ligands, although some derivatives also show high binding affinities for αSyn, as shown in Figure 73. A range of quinoline derivatives have been prepared, and the structures are summarized in Figure 74. Quinolines were first identified as tau ligands by Okamura *et al*. and selectively stain tau pathology in AD brain sections.^293,295,365,457,502,535,635^ Quinolines, such as the commonly used **145** (commonly known as **THK-523**) and **146** (commonly known as **THK-5117**), appear to target two separate sites on tau and in AD brain homogenates.^293,295,365,457,635^ The primary high-affinity site that quinolines bind in AD brain homogenates is different from the primary site targeted by carbazoles or fused benzoheterocycles.^281^

**Figure 73:**
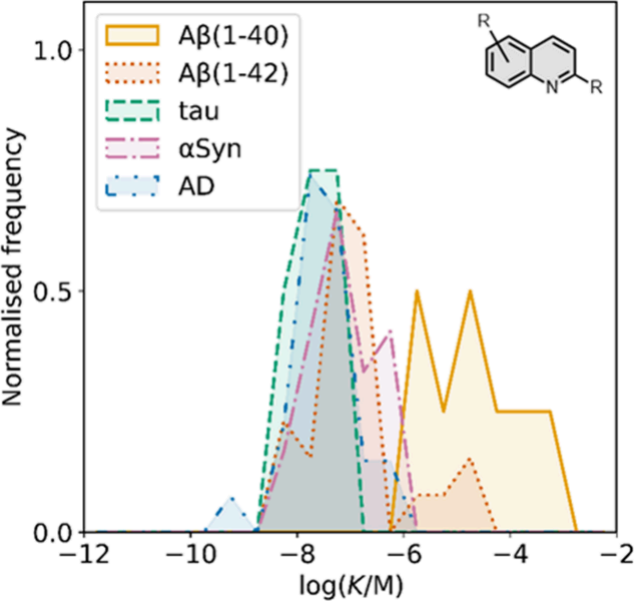
Distribution of binding affinities for the interaction of 69 quinoline ligands with amyloids: Aβ­(1-40) (solid yellow line, 8 ligands), Aβ­(1-42) (dotted orange line, 26 ligands), tau (dashed green line, 8 ligands), αSyn (dash-dot purple line, 24 ligands) and AD brain homogenates (dash-dot-dot blue line, 27 ligands). For ligands where several binding affinities have been reported, the average value was used.

**Figure 74:**
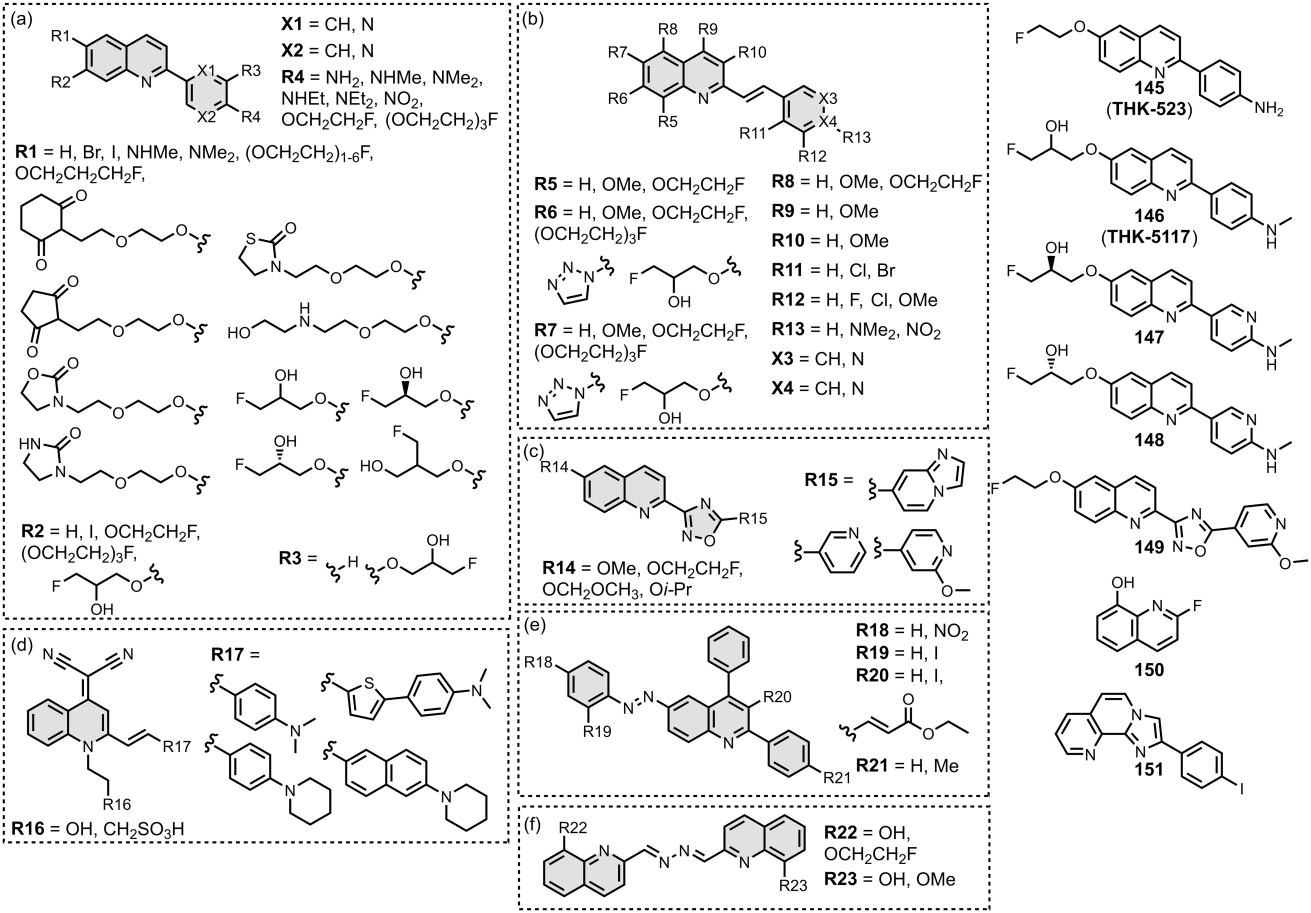
Quinoline ligands.

The length of the PEG chains at R1 in Figure 74a affected the binding affinity and the targeted binding site, and an FPEG_2_ chain provided one of the highest affinities (*K =* 28 nM).^281^ Chirality of the substituent at R1 was also important for binding, as **147** had a higher affinity (2.9 nM) for AD brain homogenates than **148** (*K =* 28 nM).^293,441,535^ Substituents at R1 gave higher affinity binding than the same substituent at R2, and substituents at R3 prevented nanomolar binding altogether.^535^ On the pendant aryl ring, various amine substituents at R4 and a nitrogen atom at X1 or X2 all afforded similar affinities for tau or AD brain homogenates.^457,535,602,635^

Styryl quinolines were investigated as αSyn ligands and showed stronger binding than styryl quinoxaline analogues (Figure 74b).^313,543^ At R7, a fluoroethoxy or FPEG_3_ substituent led to low nanomolar dissociation constants for αSyn (*K =* 4–11 nM) and Aβ­(1-42) (*K =* 8–10 nM), while an imidazole group led to selective binding for Aβ­(1-42) (*K =* 21 nM) over αSyn (*K =* 200 nM). The same substituent at R6 generally produced binding an order of magnitude weaker, although often improved selectivity. Substituents on the quinoline ring at R5, R8, R9, and R10 typically weakened binding significantly.^543^

On the pendant aryl ring, a dimethylamino substituent at R13 afforded high-affinity binding, and substituents at R12 were often tolerated. Some ligands with nanomolar dissociation constants were reported with no substituent at R13 and a nitrogen atom at X4. A nitrogen atom at X3 or substituents at R11 generally significantly reduced the measured binding affinity. Replacing the alkene linker with an oxadiazole typically prevented binding to αSyn (Figure 74c), although **149** demonstrated nanomolar binding to both αSyn (*K =* 18 nM) and AD brain homogenates (*K =* 30 nM).^313^

Quinoline-malonitrile derivatives were reported (Figure 74d) with nanomolar affinities for Aβ (*K* = 35–170 nM) and NIR emissions.^347,455,514^ A small number of aryldiazoquinolines were also prepared (Figure 74e) and had mid-nanomolar affinities for Aβ­(1-42) (*K* = 170–200 nM) with some evidence for inhibition of aggregation.^262^ Several bisquinolines have been reported with nanomolar affinities for αSyn (*K =* 3–17 nM) and Aβ­(1-42) (*K =* 1–9 nM) (Figure 74f).^390^

A small number of other quinoline ligands have been reported. **150** had a nanomolar dissociation constant (*K* = 1.5 nM) for Aβ aggregates.^590^ The tricyclic derivative **151** exhibited high-affinity binding to AD brain homogenates (IC_50_ = 0.2–1.6 nM) and targeted NFTs over Aβ plaques in AD brain sections.^336^

Several charged quinolinium ligands have been reported (Figure 75). Carbazole-quinolinium conjugates generally showed micromolar affinities for Aβ­(1-40), but they inhibited aggregation and had emissions above 600 nm (Figure 75a).^352,378,419^ Quinolinium-aniline conjugates (Figure 75b) also inhibited aggregation and showed micromolar binding to Aβ­(1-40) and Aβ­(1-42) monomers, oligomers, and fibrils.^241^ Quinolinium derivatives with a pendant alkene-linked thiophene had nanomolar dissociation constants for Aβ­(1-42) (*K* = 50–210 nM) and fluorescence emissions above 600 nm (Figure 75c). A coumarin-quinolinium conjugate, **152**, was reported which selectively bound to Aβ­(1-42) (*K =* 86 nM) over tau and αSyn.^265^

**Figure 75:**
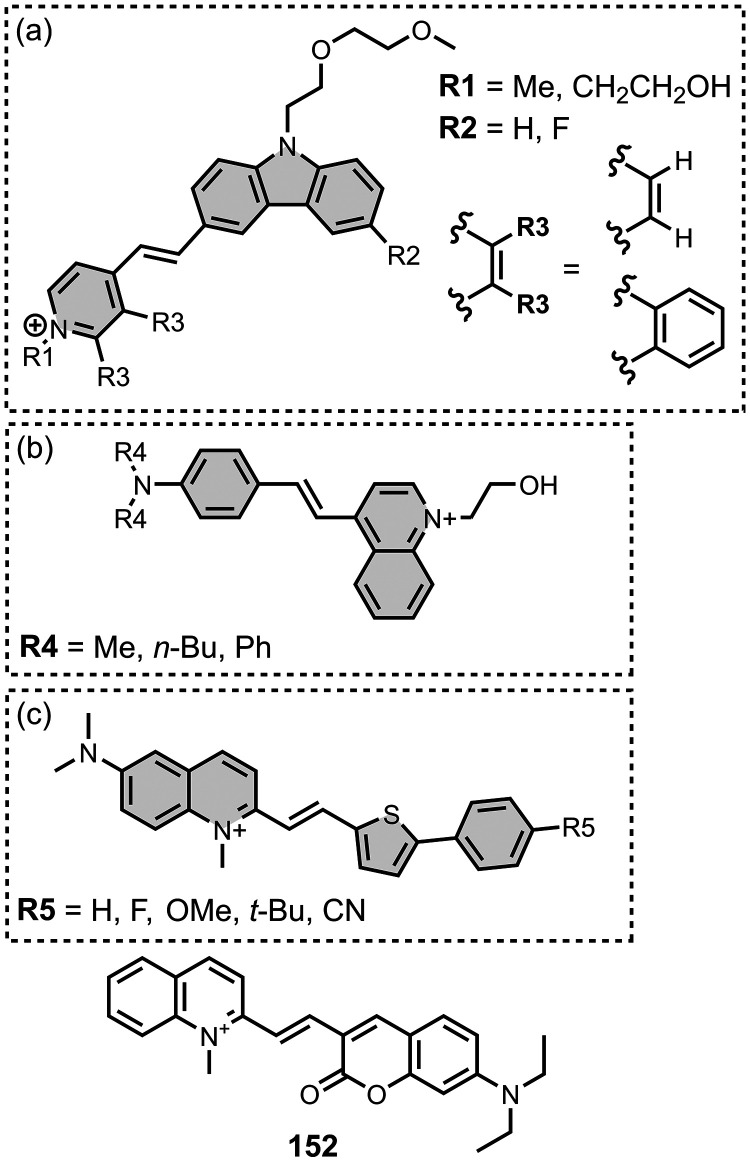
Charged quinolinium ligands.

## 21 Quinoxalines

Quinoxalines generally show weak selectivity for Aβ­(1-42), with the measured binding affinities summarized in Figure 76. Quinoxalines were reported as amyloid ligands from Honson *et al*.’s screening efforts.^333^ Several quinoxaline derivatives have since been reported, and the chemical structures are summarized in Figure 77.

**Figure 76:**
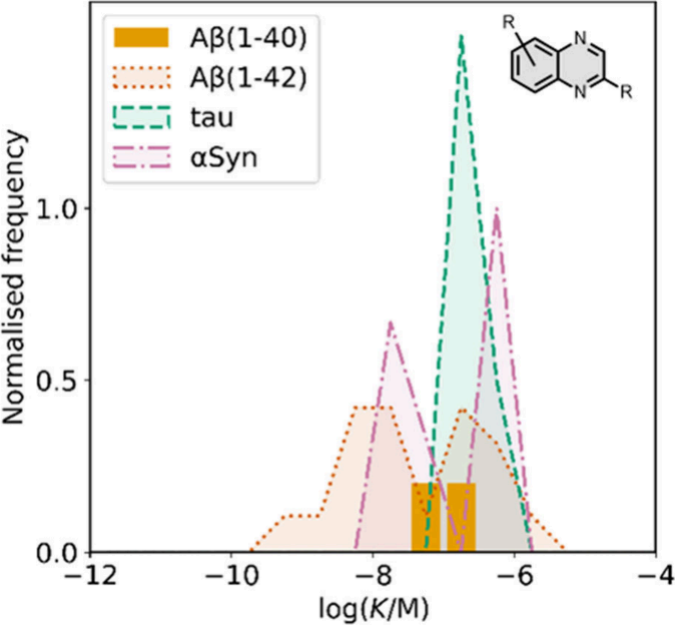
Distribution of binding affinities for the interaction of 21 quinoxaline ligands with amyloids: Aβ­(1-40) (yellow bars, 2 ligands), Aβ­(1-42) (dotted orange line, 19 ligands), tau (dashed green line, 4 ligands), and αSyn (dash-dot purple line, 6 ligands). Bars are used to represent three or fewer ligands. For ligands where several binding affinities have been reported, the average value was used.

**Figure 77:**
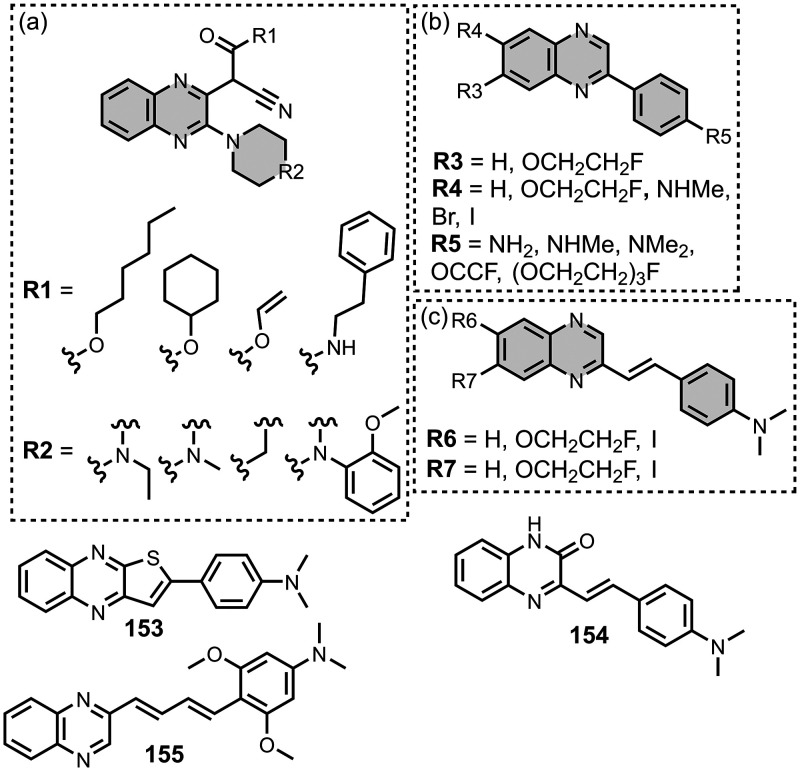
Quinoxaline ligands.

Nitrile quinoxaline derivatives were screened with Aβ­(1-42), tau, and αSyn (Figure 77a).^333^ An amide at R1 led to no binding, whereas esters at R1 and a range of substitutents at R2 resulted in similar nanomolar affinities to all three fibrils (*K =* 150–720 nM). Phenyl-substituted quinoxalines were explored as Aβ­(1-42) ligands (Figure 77b). At R4, amino, iodo, and FPEG substituents afforded nanomolar binding (*K =* 3–11 nM), and at R5, methylation of amine substituents was preferred.^206,470,538^ Introduction of the alkene spacer in Figure 77c led to similar or weaker binding affinities (*K =* 11–80 nM) that were comparable to those of styryl-quinoline analogues (*K =* 4–40 nM).^543^

Several quinoxaline derivatives with unique modifications were prepared (**153**, **154**, **155**), but they exhibited only modest affinities for Aβ (*K =* 80–350 nM).^238,242^ Both **153** and **154** demonstrated significant solvatochromism upon binding.

## 22 Acridines

Acridine derivatives have mainly been screened with Aβ­(1-42), showing mid-nanomolar dissociation constants (Figure 78). Figure 79 summarizes the structures of the acridine ligands reported. Acridines with two amino substituents in Figure 79a had nanomolar dissociation constants for Aβ­(1-42) (*K* = 32–167 nM) and bound SPs in the AD temporal cortex.^235,411^ For the iodoacridines in Figure 79b, methoxy and dimethylamine groups at R4 were well tolerated (*K =* 10–40 nM). At R3, a dimethylamine group reduced the affinity (*K* = 120 nM) and a methoxy group removed measurable binding (*K >* 10,000 nM). A larger amine substituent at R3, as in **156**, reduced the binding affinity when measured by an competition assay (*K =* 350 nM) but maintained good affinity in a direct binding assay when radiolabelled (*K =* 50 nM). Acridines therefore appear to target two sites, one of which is shared with **IMPY**. The modified acridone **157** was also prepared and exhibited anti-aggregation activity but had only a weak affinity for Aβ­(1-42) and tau (*K =* 16,000–34,000 nM).

**Figure 78:**
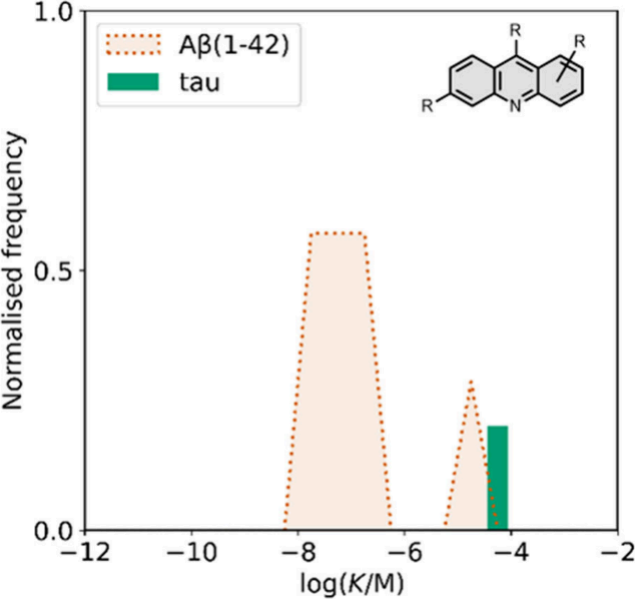
Distribution of binding affinities for the interaction of 7 acridine ligands with amyloids: Aβ­(1-42) (dotted orange line, 7 ligands), and tau (green bar, 1 ligand). Bars are used to represent three or fewer ligands. For ligands where several binding affinities have been reported, the average value was used.

**Figure 79:**
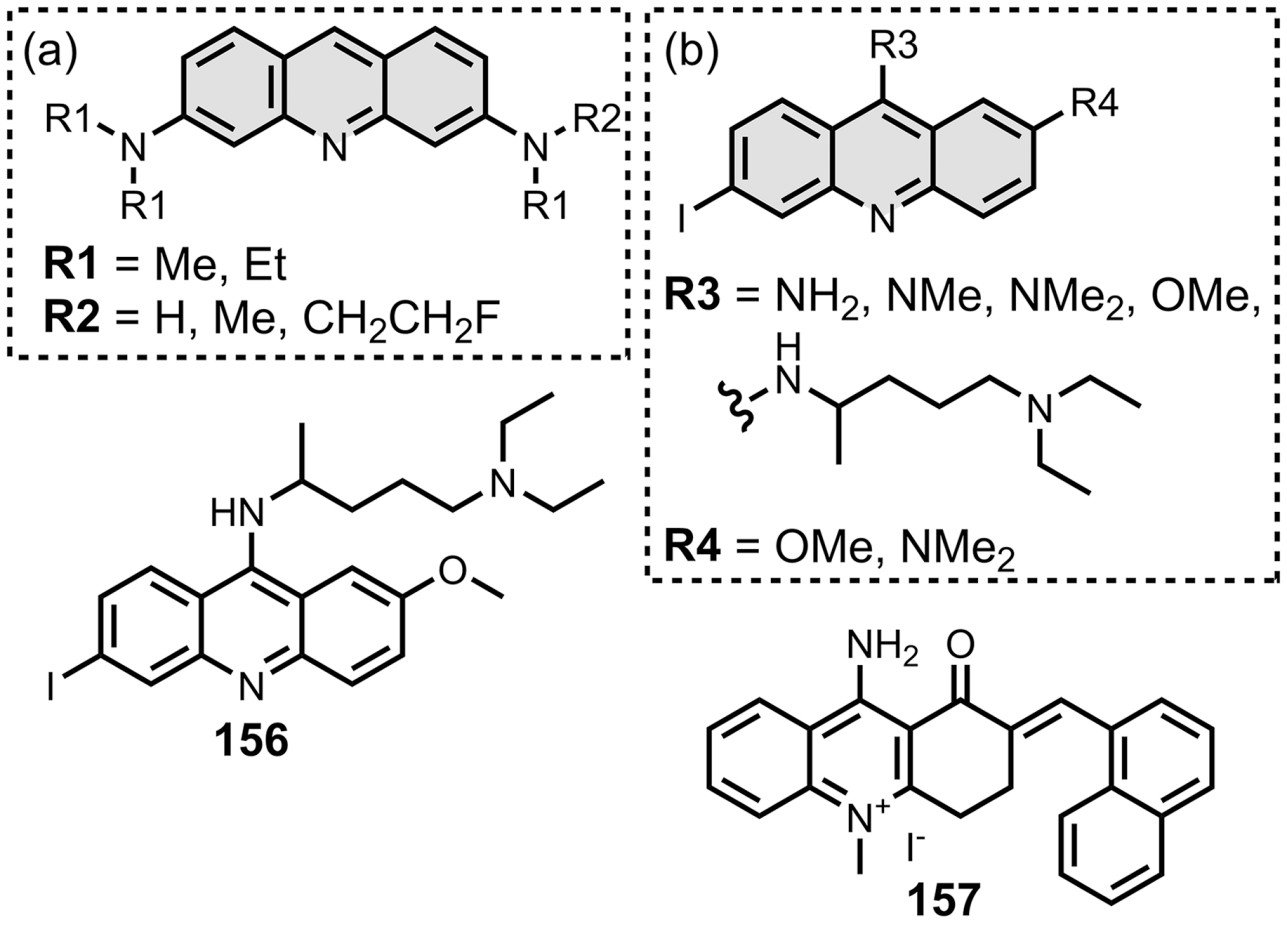
Acridine ligands.

## 23 Phenothiazines

Phenothiazines have emerged as a promising class of αSyn ligands, with the measured binding affinities summarized in Figure 80. The structures of the phenothiazine ligands are summarized in Figure 81.^272,588^ A nitro group at R1 produced high affinity binding, and the fluoroethoxy group at R2 in **158** led to the highest affinity derivative for αSyn (*K =* 16 nM). **158** also has nanomolar affinity binding for PD brain homogenates (34 nM), and some selectivity over Aβ­(1-42) (*K =* 103 nM) and tau (*K =* 125 nM). Aryl-pyrazole substituents at R1 were tested, but none showed selectivity for αSyn over Aβ­(1-42). Substituents at R3 led to dissociation constants greater than 500 nM. Additionally **ThT**, **CG**, **BF-227**, and **PiB** all displaced phenothiazines from αSyn and PD homogenates suggesting a common binding site.^272^ The charged phenothiazine **159** (commonly known as methylene blue) was also investigated for binding to tau but showed low affinity.^563^ Several phenothiazine-rhodanine conjugates have been reported and exhibit a range of affinities for Aβ­(1-42) (*K =* 8–700 nM; see the Rhodanines section).^328,371^

**Figure 80:**
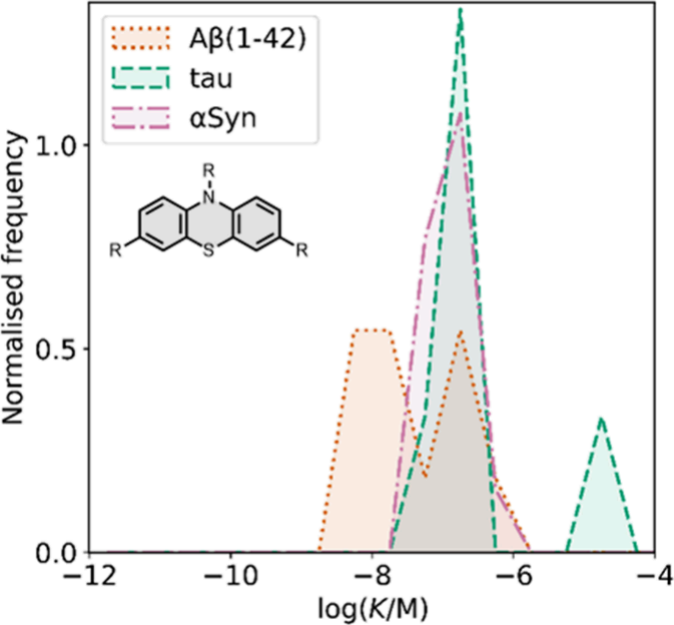
Distribution of binding affinities for the interaction of 15 phenothiazine ligands with amyloids: Aβ­(1-42) (dotted orange line, 11 ligands), tau (dashed green line, 6 ligands), and αSyn (dash-dot purple line, 13 ligands). For ligands where several binding affinities have been reported, the average value was used.

**Figure 81:**
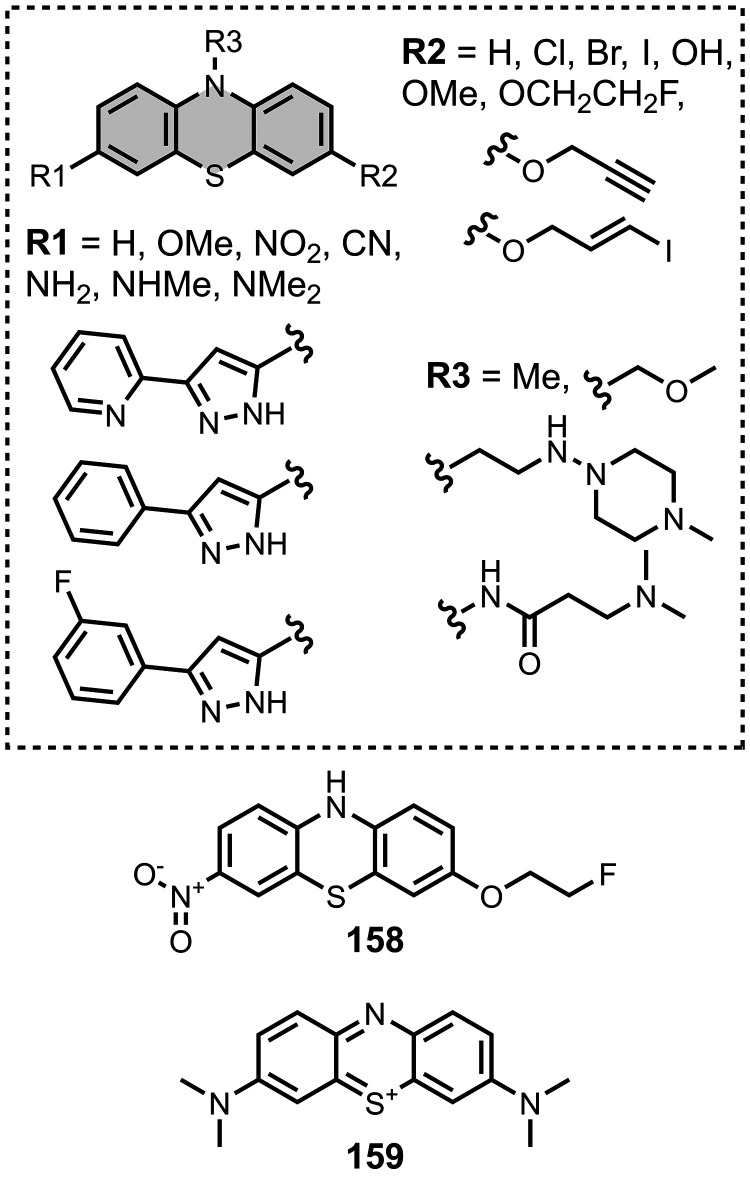
Phenothiazine ligands.

## 24 Carbazoles

Carbazoles appear to predominantly bind tau in AD brain homogenates, with the measured binding affinities summarized in Figure 82. Figure 83 summarizes the structures of the carbazoles synthesized. Honson *et al*. first identified carbazoles as amyloid ligands with the report of **160**.^333^ **161** (commonly known as **T807**, **AV-1451**, and **flortaucipir**) and **162** (commonly known as **T808**, **AV-680**, or **GTP-1** if deuterated) have since become two of the most commonly employed carbazole ligands and exhibit selective binding to tau, targeting both a high-affinity (*K* = 0.4–4 nM) and a low-affinity site (*K* = 80–100 nM).^205,227,351^ Carbazoles appear to target a different high-affinity site than fused benzoheterocycle or quinolines, and they do not compete with **PBB3**.^281,293,337,341^ These carbazoles show promising *in vivo* properties, although **T807** shows off-target binding to monoamine oxidases A and B.^293,294,613^ Some carbazole-quinoline conjugates have also been reported (see quinoline section).

**Figure 82:**
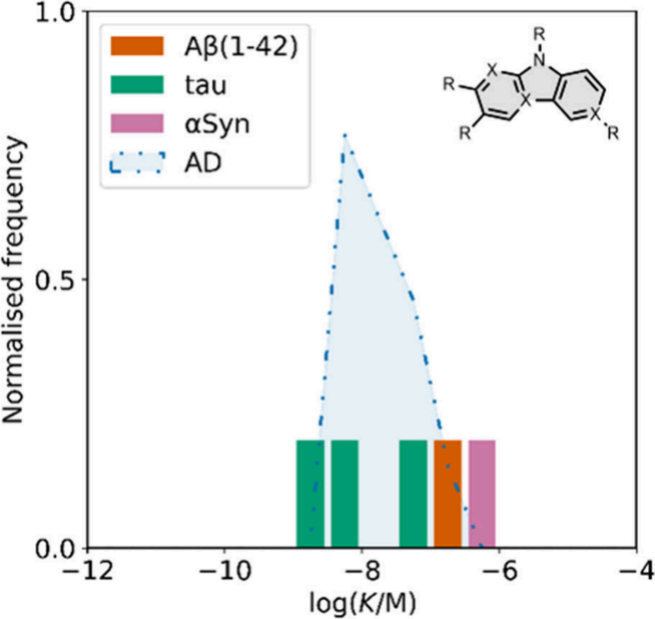
Distribution of binding affinities for the interaction of 14 carbazole ligands with amyloids: Aβ­(1-42) (orange bar, 1 ligand), tau (green bars, 3 ligands), αSyn (purple bar, 1 ligand) and AD brain homogenates (dash-dot-dot blue line, 13 ligands). Bars are used to represent three or fewer ligands. For ligands where several binding affinities have been reported, the average value was used.

**Figure 83:**
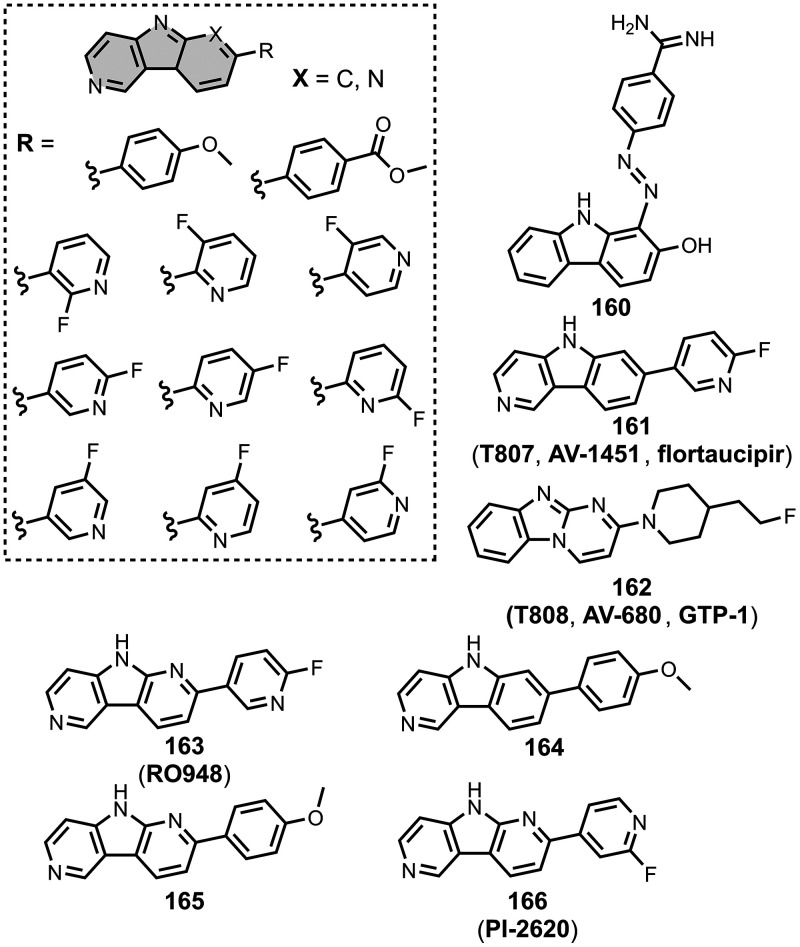
Carbazole ligands.

Introducing an additional nitrogen atom into the carbazole ring system had an unpredictable effect on binding affinity. Both **T807** and **163** (commonly known as **RO948**) have nanomolar dissociation constants for AD brain homogenates (*K =* 1–4 nM), whereas **164** showed nanomolar binding (*K =* 5–14 nM) and **165** showed no binding (*K >* 4,000 nM).^294,337^ No consistent SAR was observed when the R substituent was varied. The ligand **166** (commonly known to as **PI-2620**) was identified as a high-affinity ligand for tau from AD and PiD brain homogenates (IC50 = 2–4 nM) and exhibited negligible binding to monoamine oxidase B, although **PI-2620** bound to tau from PSP brain homogenates with a lower affinity (IC50 = 20 nM).^316^ ^[REFS]^ **PI-2620** has demonstrated promising properties for use as a PET probe for AD, but further research is needed to verify it’s utility as a PET probe for non-AD tauopathies.^674–676^

## 25 Imidazobenzothiazoles

Imidazobenzothiazoles have been reported as Aβ ligands by Yousefi *et al*. and Alagille *et al*.^193,568^ All the ligands prepared exhibited nanomolar binding affinities (*K =* 4–133 nM) as shown in Figure 84. The structures of the ligands are summarized in Figure 85. At R1, a methoxy group was favored, and at R2, a monomethyl amino group led to the high affinity ligand **167** (*K =* 4–6 nM).

**Figure 84:**
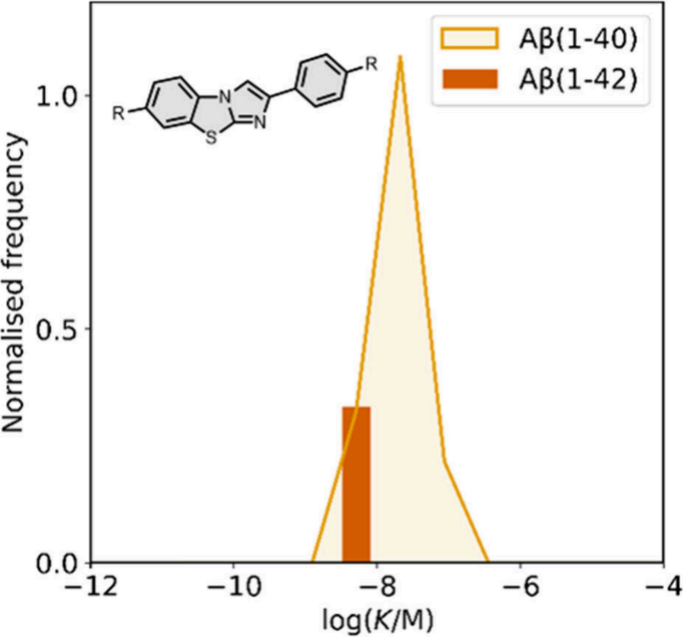
Distribution of binding affinities for the interaction of 15 imidazobenzothiazole ligands with amyloids: Aβ­(1-40) (solid yellow line, 15 ligands), and Aβ­(1-42) (orange bar, 1 ligand). Bars are used to represent three or fewer ligands.

**Figure 85:**
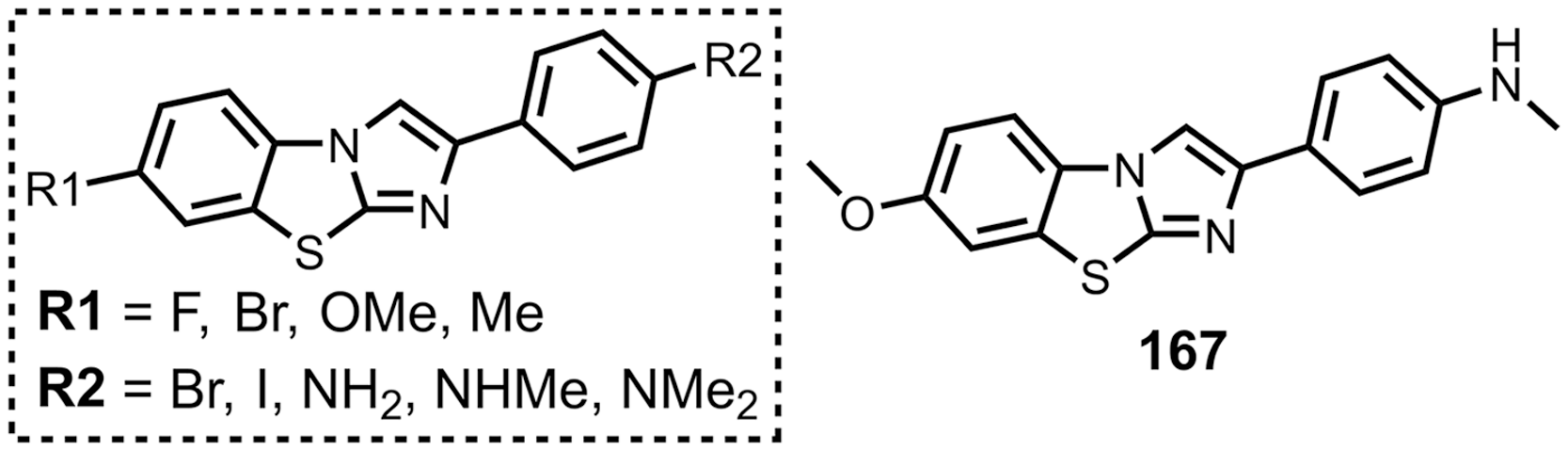
Imidazobenzothiazole ligands.

## 26 Fluorenes

Fluorenes were reported by Lee *et al*. to displace the fused benzoheterocycle ligand **TZDM** from Aβ­(1-40), with measured binding affinities summarized in Figure 86.^335^ Figure 87 shows the chemical structures of the fluorene ligands explored. The ligand **168** had the highest affinity binding measured for Aβ­(1-40) (*K <* 1 nM). Halides or a dimethylamino group at R1 and R4 were essential for binding, and substituents at R2 and R3 lead to lower affinity ligands. Substituents at R5 to form a fluorenone or hydroxyfluorene significantly reduced the measured binding affinities.

**Figure 86:**
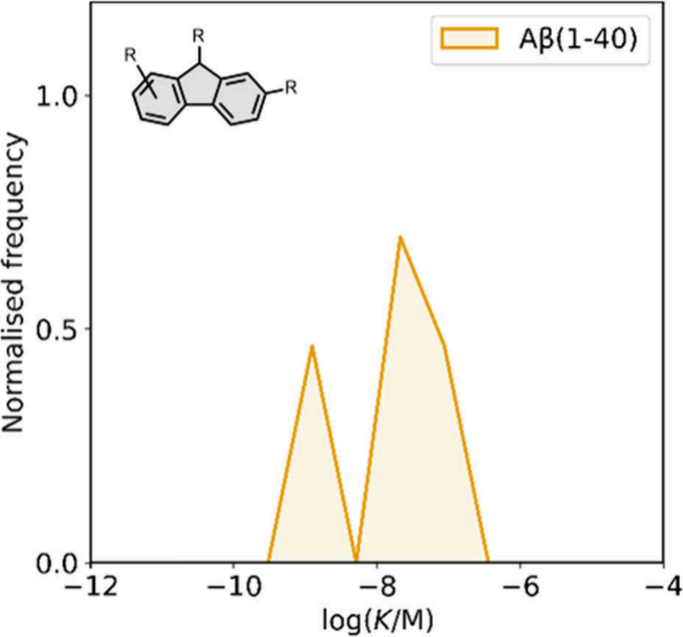
Distribution of binding affinities for the interaction of 7 fluorene ligands with Aβ­(1-40) (solid yellow line, 7 ligands).

**Figure 87:**
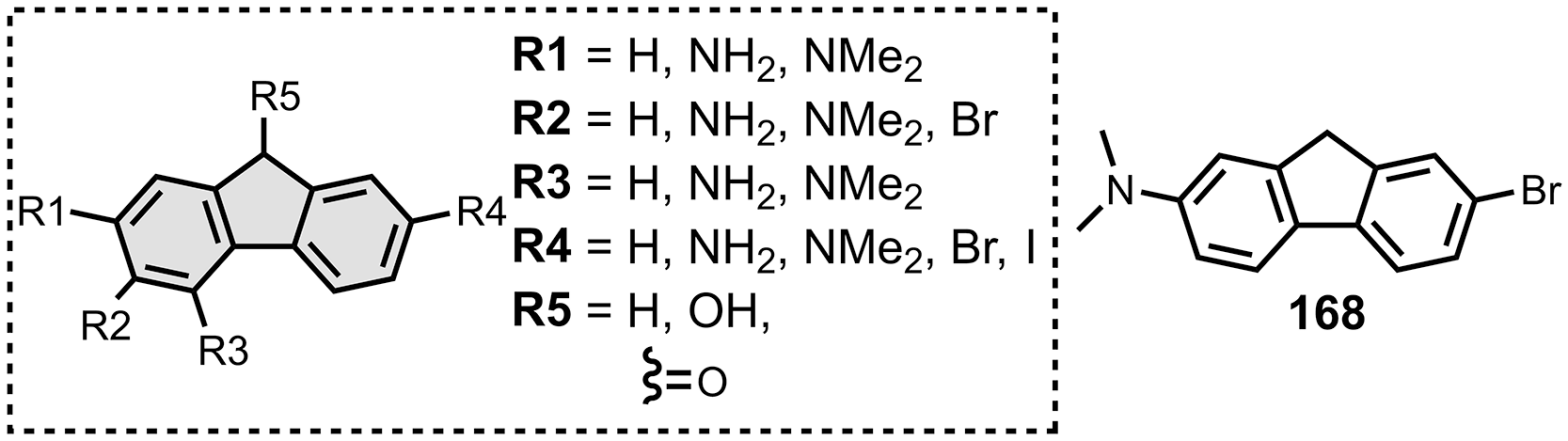
Fluorene ligands.

## 27 Boron Complexed Dyes

Several fluorescent difluoroboron-curcumin and boron-dipyrromethene (BODIPY) dyes have been reported as amyloid ligands and screened for binding to tau and Aβ, with the affinities shown in Figure 88.^315^ These ligands typically display promising fluorescence properties, including fluorescence emissions above 600 nm.

**Figure 88:**
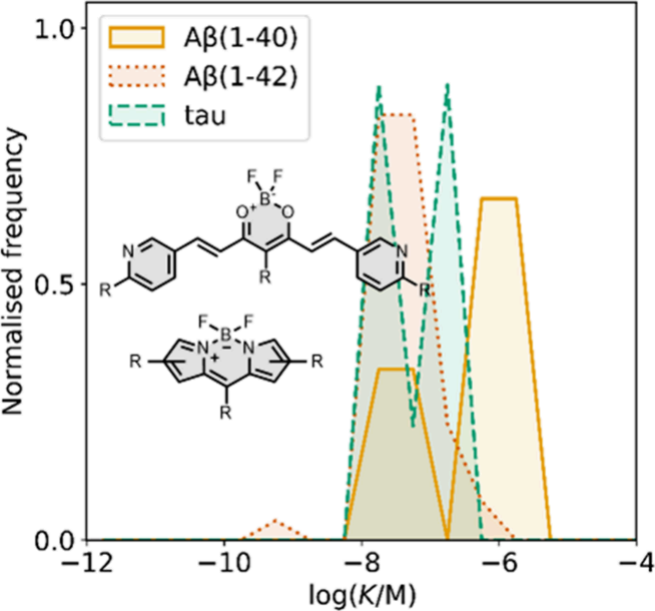
Distribution of binding affinities for the interaction of 64 boron-complexed ligands with amyloids: Aβ­(1-40) (solid yellow line, 6 ligands), Aβ­(1-42) (dotted orange line, 53 ligands), and tau (dashed green line, 9 ligands). Bars are used to represent three or fewer ligands. For ligands where several binding affinities have been reported, the average value was used.

The structures of reported boron-complexed dyes are summarized in Figure 89. Difluoroboron-curcumin derivatives were shown to bind to Aβ fibrils, monomers and oligomers (Figure 89a).^306,315,448,485,624^ The ligand **169** was the first difluoroboron-curcumin derivative prepared and exhibited a nanomolar dissociation constant for Aβ (*K =* 39 nM) and an emission (λ_em_ = 805 nm) that, upon binding, is blue-shifted (λ_em_ = 715 nm) and more intense.^315^ Incorporating pyridine rings by making X1 and X2 nitrogen had little effect on the measured affinity for Aβ (*K =* 40-50 nM) while substitution of the core, as in **170**, reduced the measured binding affinity (500 nM). **171** and **172** exhibited modest nanomolar affinities for Aβ­(1-42) (*K =* 22–52 nM), and **173** exhibited a sub-nanomolar dissociation constant (*K =* 0.6 nM).^485^

**Figure 89:**
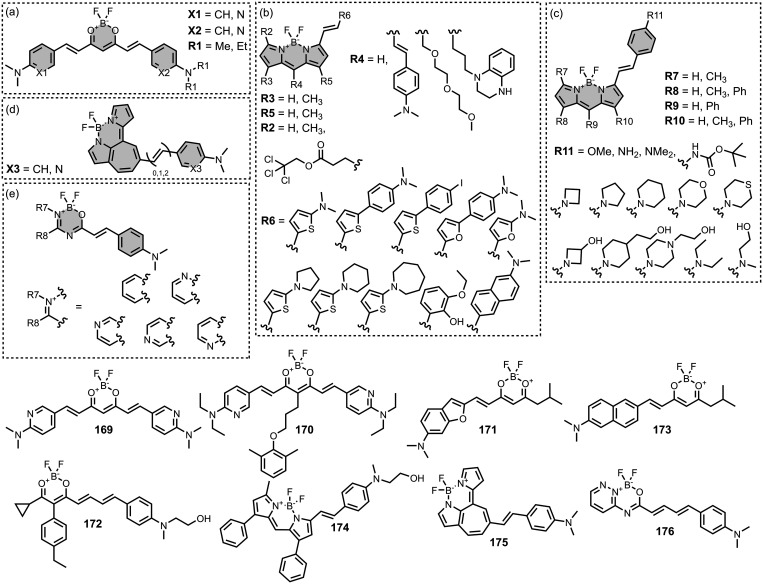
Boron-complexed ligands.

The BODIPY derivatives in Figure 89b and Figure 89c all had nanomolar dissociation constants for Aβ­(1-42) (*K =* 16–300 nM).^280,324,368,440^ Substituents at R3, R4, and R5 in Figure 89b were well tolerated, whereas the choice of substituent at R6 had a large effect on the measured dissociation constant.^440^ A number of phenyl derivatives with nitrogen substituents at R11 (Figure 89c) were explored, and **174** showed the most promising combination of binding (*K* = 16 nM), fluorescence, and *in vivo* properties.^219,316,446,580^ Fused cycloheptatriene derivatives (Figure 89d) were also prepared and screened with tau.^380^ A monoalkene or dialkene spacer improved affinity (*K =* 13–48 nM) over no spacer (*K =* 120–130 nM), while a pyridine ring at X3 did not reliably affect the affinity. **175** had the highest affinity of all the studied ligands for tau (*K =* 13 nM).

*N*,*O*-Benzamide derivatives were also prepared (Figure 89e).^361^ Introducing an additional nitrogen atom at R7 or R8 strongly affected both the binding affinity and selectivity to Aβ­(1-42) and tau. The 4-pyrimidine derivative showed high affinity binding to both Aβ­(1-42) (*K =* 24 nM) and tau (*K =* 26 nM), whereas the pyrazine derivative **176** showed highly selective binding to Aβ­(1-42) (*K =* 20 nM) over tau (no binding detected).

## 28 Other Structures

Additional amyloid ligands have been reported with diverse structural cores. An overview is provided here with the measured affinities shown in Figure 90. Full details of these ligands are available in the Supporting Information.

**Figure 90:**
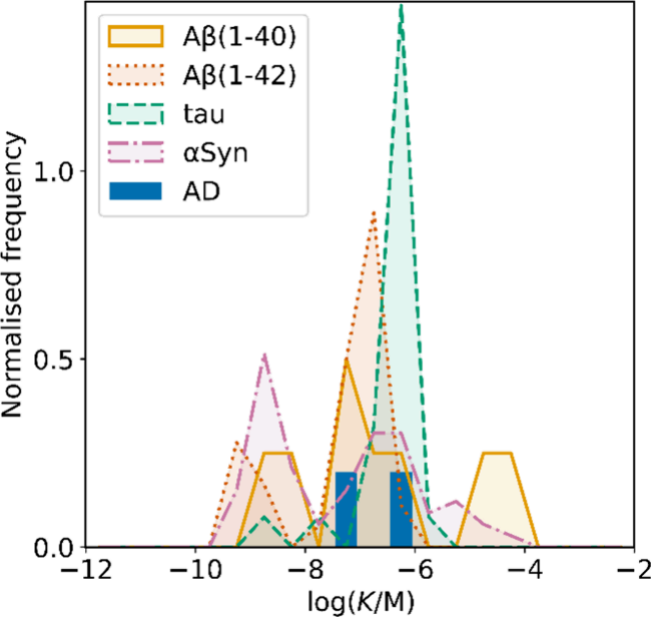
Distribution of binding affinities for the interaction of 77 other ligands with amyloids: Aβ­(1-40) (solid yellow line, 8 ligands), Aβ­(1-42) (dotted orange line, 36 ligands), tau (dashed green line, 25 ligands), αSyn (dash-dot purple line, 66 ligands) and AD brain homogenates (blue bar, 2 ligands). For ligands where several binding affinities have been reported, the average value was used.

A screening effort by Honson *et al*. reported a number of amyloid ligands for Aβ­(1-42), tau, and αSyn.^333^ The ligand **177** (commonly known as crystal violet) bound to all three types of fibril with stronger affinities (*K =* 17–53 nM) than the *des*methyl analogue **178** (*K =* 110–700 nM). The ligand **179** (commonly known as adriamycin) exhibited high nanomolar affinities for all fibrils (*K =* 110–800 nM), whereas the analogue **180** bound only to Aβ­(1-42) and tau (*K =* 300–900 nM) and not αSyn. The analogue **181**, resembling a combination of **177** and **182** (commonly known as **ANS**), did not bind to any fibril. A series of disarylbisthiazole ligands were prepared as potential αSyn ligands (Figure 91).^399,638^ Sub-nanomolar dissociation constants for αSyn were achieved alongside more than 1,000-fold selectivity over Aβ and tau. These ligands also appeared to target at least two binding sites on each fibril.

**Figure 91:**
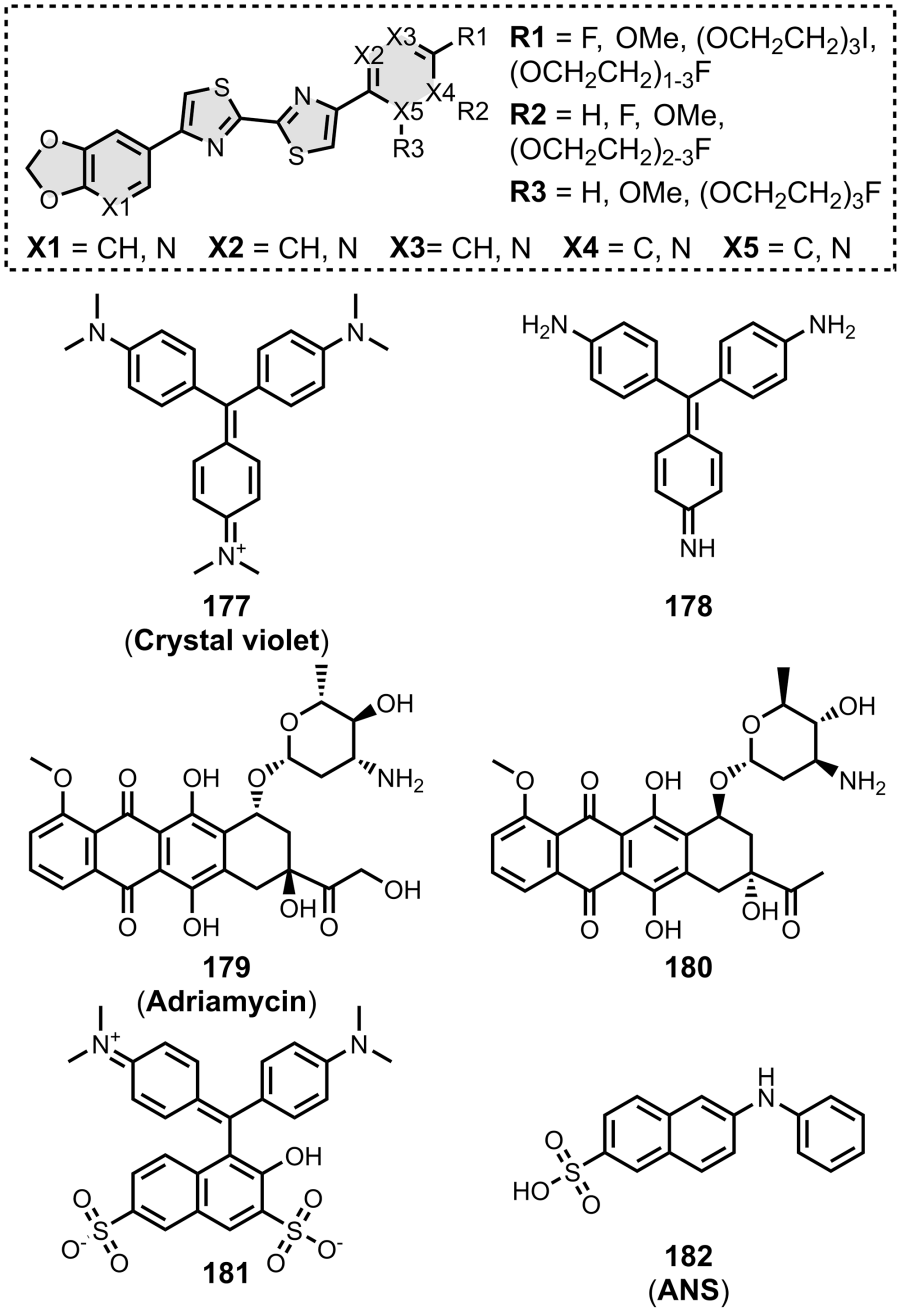
Structures of other ligands screened with Aβ­(1-42), tau, and αSyn.

Multiple Aβ ligands have been reported with structural cores that do not adhere to the classes listed above (Figure 92). The anti-inflammatory drug (*S*)-naproxen ((*S*)-**183**) was shown to bind to Aβ­(1-40) either with nanomolar affinity (*K =* 6 nM) or not at all, according to two contradicting reports.^304,639^ The ligand **184** was reported to bind to Aβ in rodent brains with a sub-nanomolar dissociation constant (*K* = 0.2 nM).^213^ Both (*R*)- and (*S*)-ibuprofen (**185**) were also screened and bound to Aβ­(1-40) with a micromolar affinity (*K =* 11,000–40,000 nM). Ferulic acid (**186**) showed sub-nanomolar affinities for Aβ­(1-42) (*K =* 0.8 nM), whereas the related compound dehydrozingerone (**187**) bound to Aβ­(1-40) with a more modest affinity (54–55 nM).^298,366^ Ferulic acid dimers were also prepared, including the piperazine derivative **188**, which showed nanomolar to sub-nanomolar affinities (*K =* 0.6–1.4 nM).^366^ Two NIR probes, **189** (commonly known as **THK-265**, *K =* 97 nM) and **190** (commonly known as **AOI987**, *K =* 200 nM), also showed nanomolar affinity for Aβ­(1-40) fibrils and were used to image Aβ plaques in mice models.^407,408^ The ligands **191** to **195** were reported from a ligand-based virtual screening effort and showed nanomolar dissociation constants (*K* = 20–600 nM),^181^ as did **196** (*K* = 16 nM) and **197** (*K* = 410 nM).^230,256^

**Figure 92:**
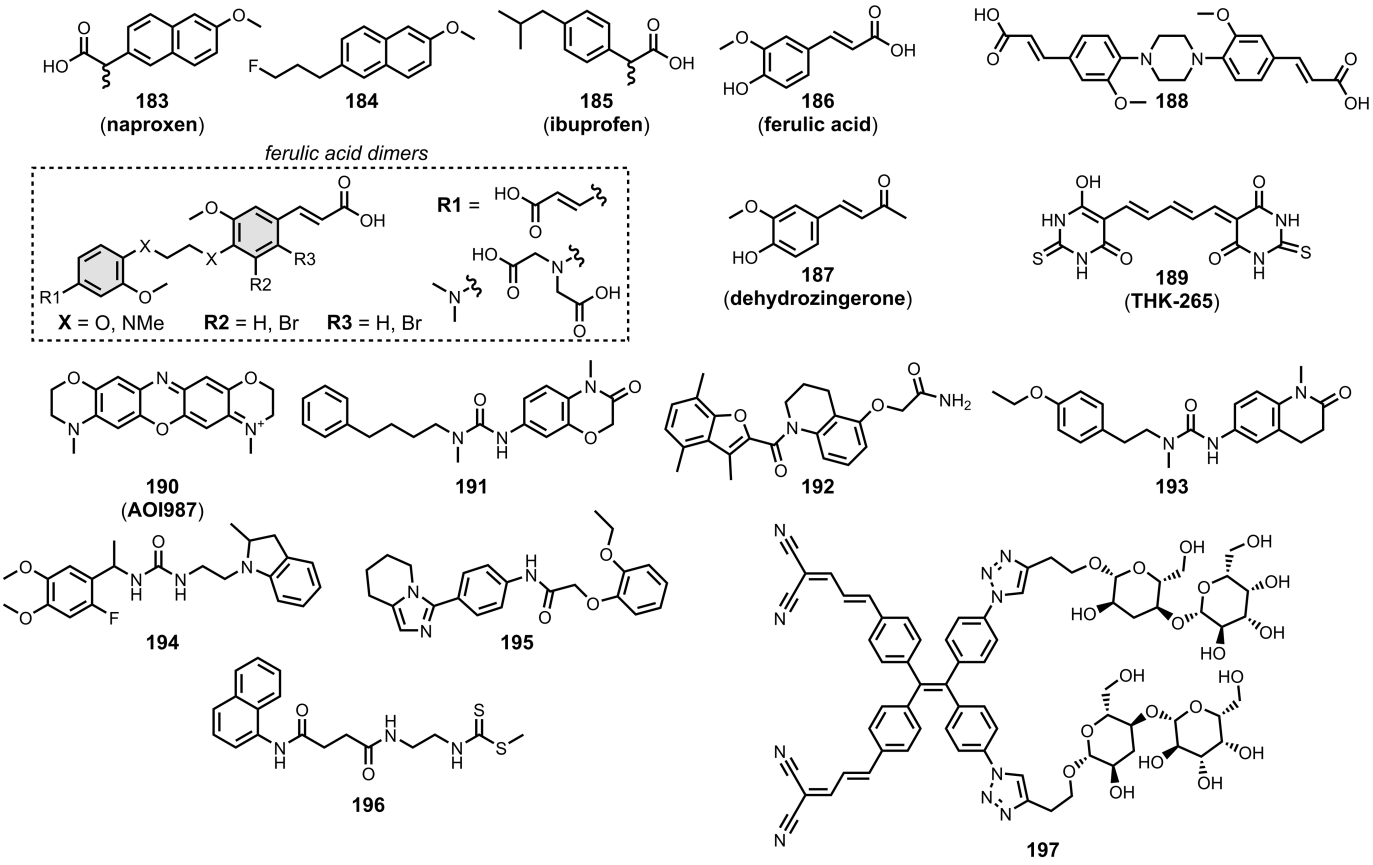
Structures of other ligands screened with Aβ.

Several other tau ligands are shown in Figure 93. The ligand **198** (commonly known as **JNJ311**) had a nanomolar affinity for tau (*K =* 8 nM) and targeted tau-rich regions in AD brain sections, with no apparent affinity for Aβ.^492^ A range of analogues have been prepared (Figure 93a), with **214** (commonly known as **JNJ067**) being investigated as a PET imaging agent for tauopathies.^677,678^ Epigallocatechin gallate (**199**, **EGCG**) also binds tau (*K =* 74 nM) and both inhibits tau aggregation and promotes tau disassembly.^354^ A series of imidazothiazolones bearing pendant aryl and styryl moieties was reported that selectively bind tau over Aβ (Figure 93b).^397^ Substituents on the styryl group at R6 influenced both affinity and selectivity over Aβ­(1-42).

**Figure 93:**
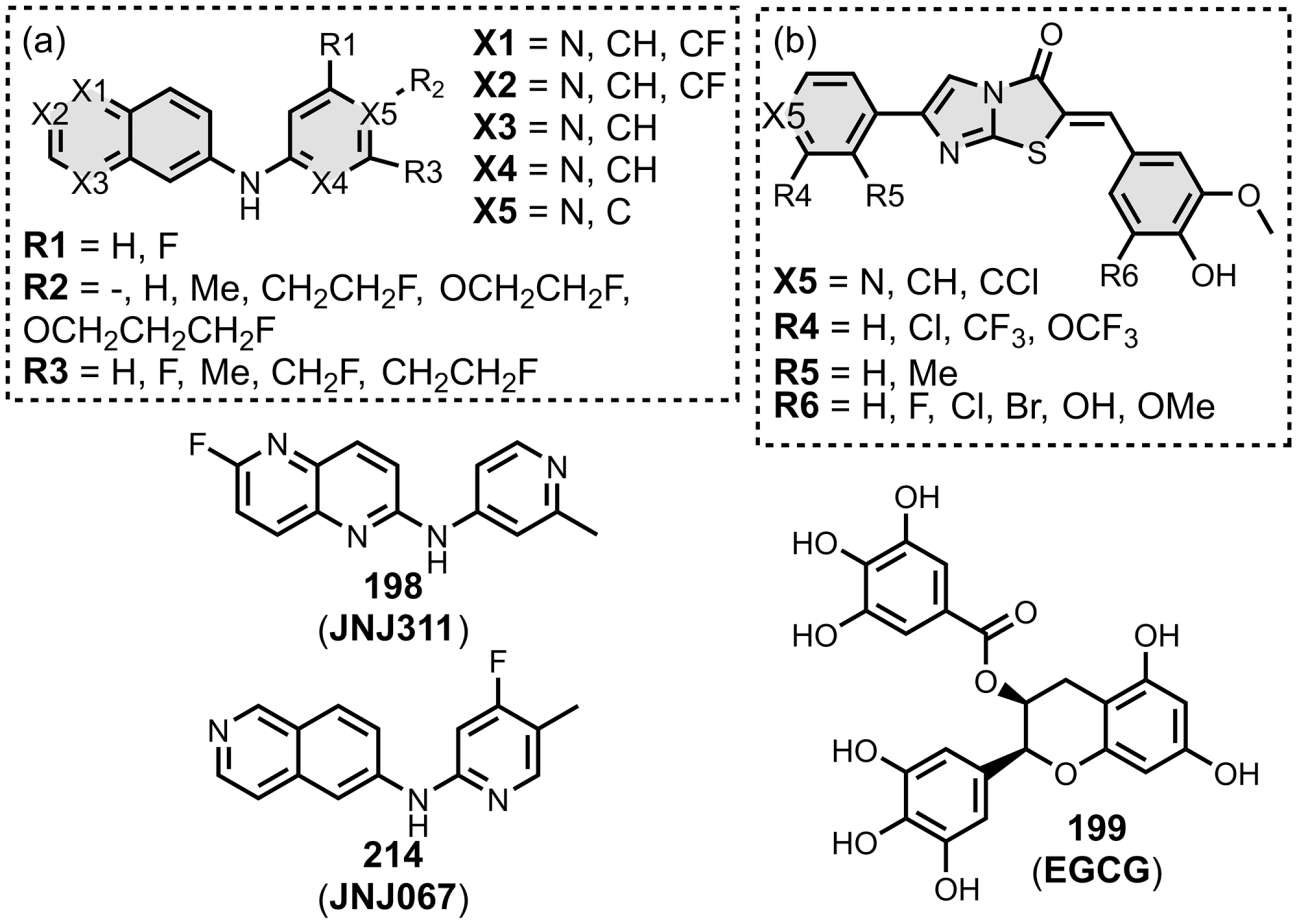
Structures of other ligands screened with tau.

An increasing number of αSyn ligands have been reported in recent years, with additional structures summarized in Figure 94. The ligands **ANS** and **200** showed low micromolar affinities for αSyn (*K =* 9,000–12,000 nM) (Figure 94a), as did the dimers **201** and **202** (*K =* 9,000–12,000 nM) (Figure 94b).^369^ Other micromolar ligands have been reported, including a series of *bis*phenyl derivatives connected by a methylene bridge (IC_50_ = 1,000–2,000 nM) (Figure 94c), the aromatic-rich **203** (*K =* 4,400 nM), the dammarane triterpene **204** (*K =* 50,000 nM), and **205** (*K =* 4,000 nM).^303,319,540,628^ A promising series of ligands were prepared with nitrogen heterocycles connected to a benzamide core (Figure 94d). A piperazine ring at R6 in **206** yielded a low nanomolar binding affinity for αSyn (*K =* 2 nM).^180,559^ Later analogues explored the heterocycle at R6 more thoroughly.^645^ The derivative **207** had nanomolar dissociation constants for PD, MSA, AD, PSP, and CBD brain homogenates (*K* = 3–18 nM), although more clearly labelled αSyn aggregates in MSA brain sections than PD brain sections.^645^ The porphyrin **208** was shown to have a modest affinity for αSyn (*K* = 301 nM).^102^ The ligand **209** showed strong binding to fibrils in PD and MSA (*K* = 17–30 nM) and was able to distinguish MSA from PD and AD when used as a PET tracer.^614^

**Figure 94:**
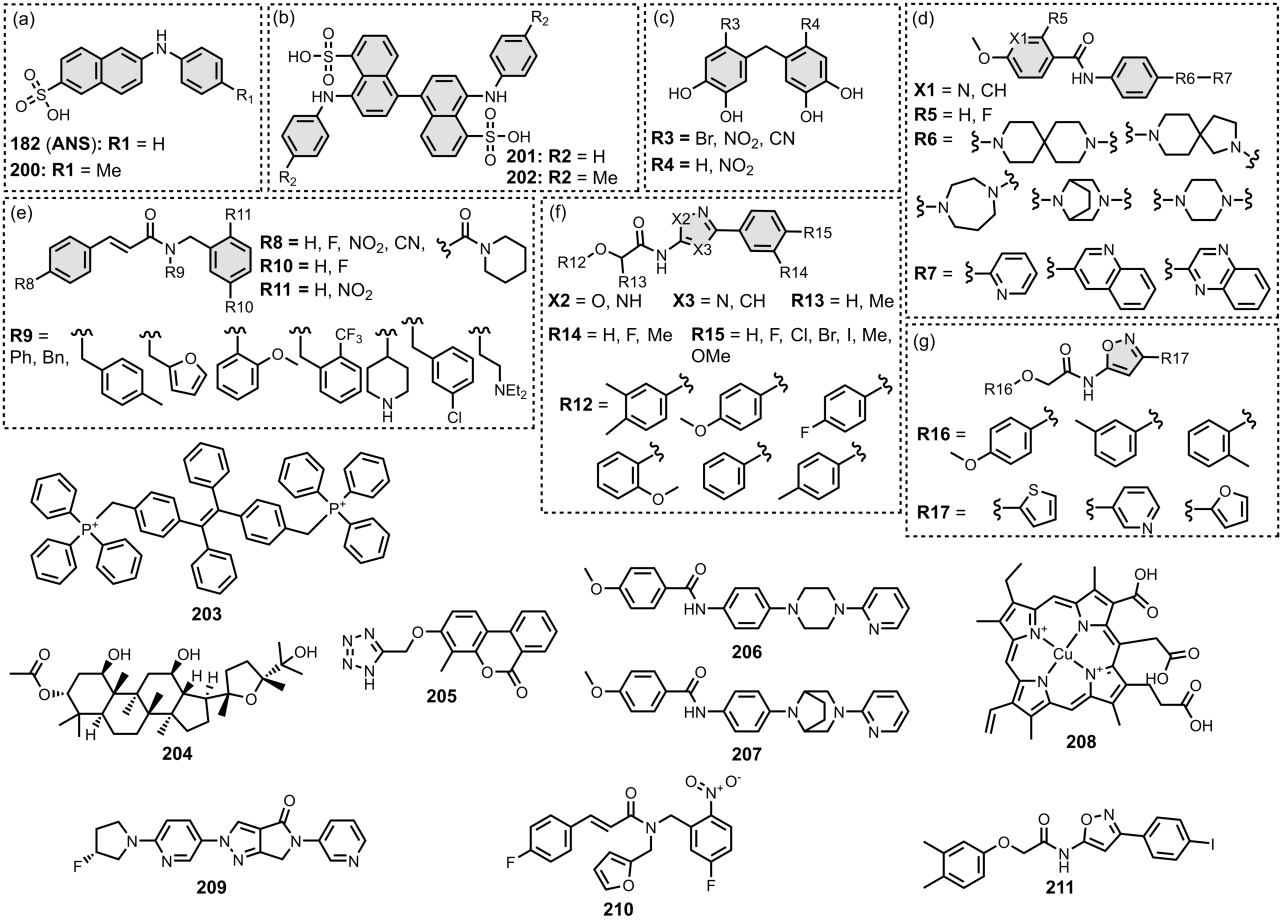
Structures of other ligands screened with αSyn.

A series of dibenzylcinnamide derivatives was prepared showing a range of binding affinities (8–600 nM) (Figure 94e).^311^ A nitro group at R11 generally led to higher affinities for these derivatives. When R8 was a nitrile and R10 hydrogen, a diethylamino substituent was preferred at R9 (*K =* 8 nM) over aromatic or aliphatic rings (*K =* 110–570 nM). For a fluoro group at R8 and R10, a methylfuran group at R7 (**210**, *K =* 1 nM) led to a higher affinity than a benzyl (*K =* 11 nM) or *o*-methoxybenzyl (*K =* 330 nM) group. A series of phenylisoxazole derivatives were prepared based on *in silico* screening and subsequent SAR studies (Figure 94f,g).^392^ Many of these derivatives exhibited nanomolar IC_50_ values for αSyn. Modifications at X2 and X3 to yield a pyrazole or oxadiazole ring led to no binding, and different substituents on the pendant aromatic rings had a significant effect on binding affinity. The derivative **211** was the most promising αSyn ligand, exhibiting a low nanomolar dissociation constant for both αSyn (*K =* 1 nM) and Aβ­(1-42) (*K =* 5 nM).

## 29 Pet Tracers

A key goal in the development of amyloid-binding ligands is to develop PET tracers for the imaging of amyloid deposits *in vivo*. PET tracers should have low nanomolar dissociations constants for their target fibril and should bind selectively over other fibrils or cellular targets with low non-specific binding. Successful PET tracers should also have high blood–brain barrier permeability, show rapid clearance from normal brain tissue, and should not generate radiolabeled metabolites in the brain.^169,603^

To date, only four PET ligands that target the protein fibrils discussed in this review have been approved by the FDA (Figure 95). 18F-florbetaben ([^18^F]**212**, [^18^F]**AV-1**), 18F-florbetapir ([^18^F]**69**, [^18^F]**AV-45**), and 18F-flutemetamol ([^18^F]**213**) have been approved for imaging Aβ fibrils, and 18F-flortaucipir has been approved for imaging tau fibrils.^679,680^ Notably 18F-flortaucipir ([^18^F]**161**, [^18^F]**T807**, [^18^F]**AV-1451**) has limited use for differential diagnosis and imaging in non-AD tauopathies.^681–683^ No PET tracers have been approved for synucleinopathies.

**Figure 95:**
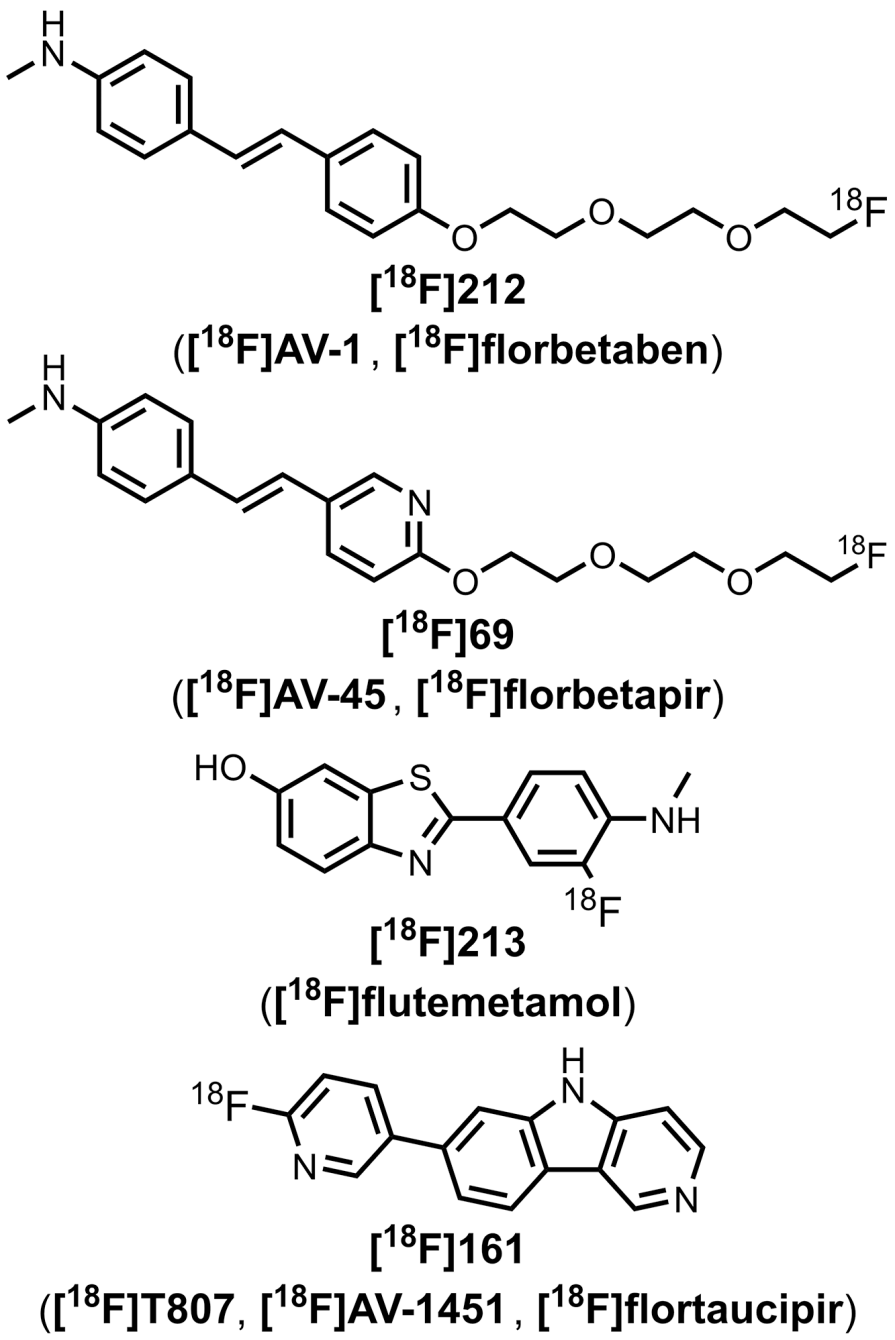
FDA approved PET tracers for amyloid fibrils.

A range of other ligands are being developed as potential PET probes for both tauopathies and synucleinopathies. Fewer PET probes are now being researched for Aβ plaques, although other imaging modalities including NIR imaging are being investigated.^449,534^

The development of tau PET probes has been the focus of significant research efforts.^480,603,609,680,681,684–686^ Early tau radiotracers include THK derivatives, **FDDNP** (**129**), and **PBB3** (**18**) (Figure 96). These ligands encountered several challenges in labelling tau aggregates in AD and other tauopathies, and they suffer from substantial off-target binding. Later PET tracers have since been reported and applied in various research efforts including **PM-PBB3** (**19**), **MK-6240** (**47**), **PI-2620** (**166**), **RO-948** (**163**), **JNJ-067** (**214**), **GTP-1** (**162**, also known as **T808** or **AV-680** if not deuterated), and 18F-flortaucipir ([^18^F]**161**, [^18^F]**T807**, [^18^F]**AV-1451**), which was approved for use by the FDA.

**Figure 96:**
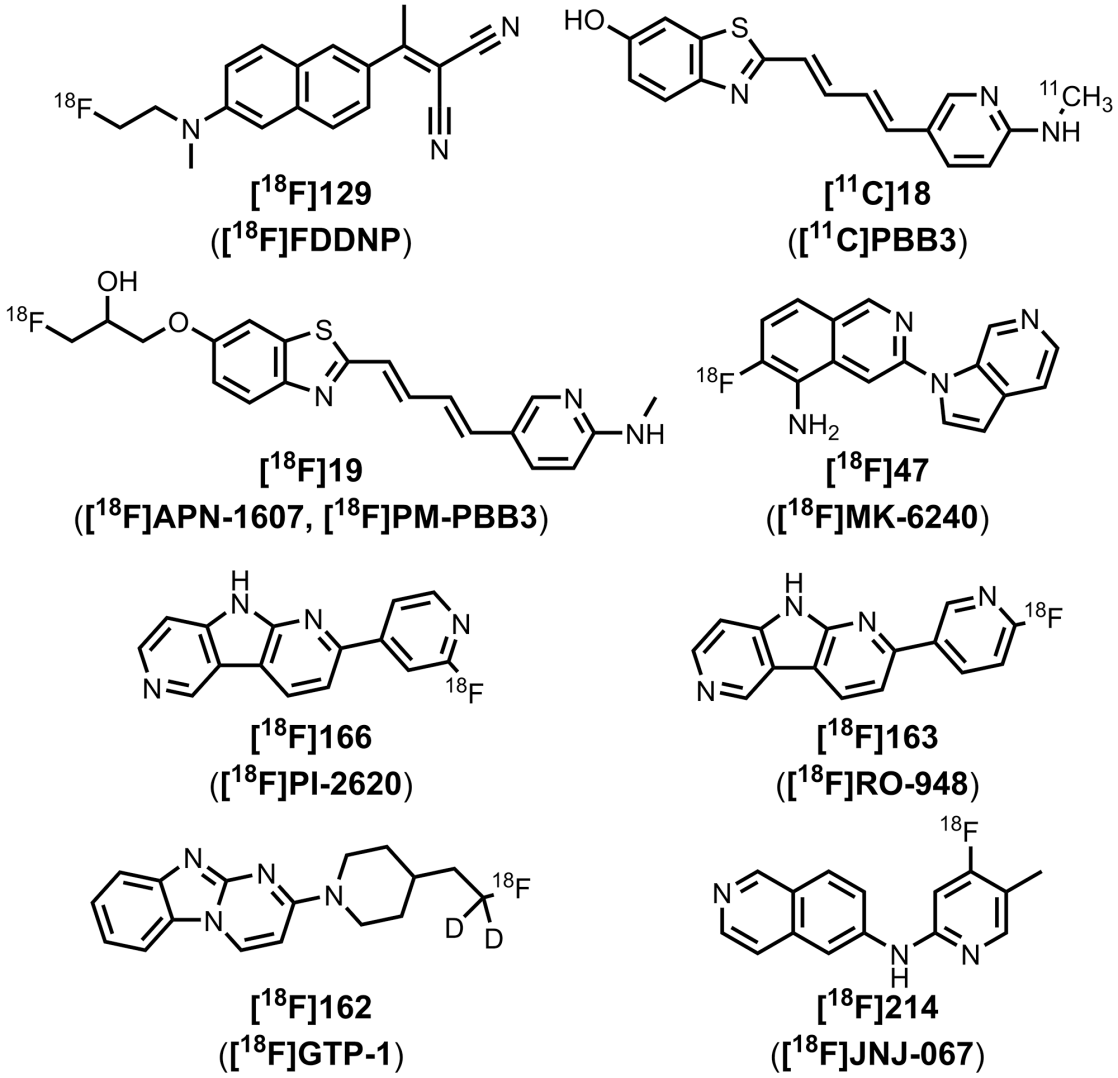
Candidate PET probes developed for imaging tau aggregates.

Many of these early tau ligands were most successful for labelling tau aggregates in AD. Indole and PI-2620 (**166**) derivatives have since been developed with the goal of specifically targeting 4R tauopathies.^676,680,687^ Developing selective tau PET tracers that can be used in non-AD tauopathies remains a challenge and highlights the difficulties in achieving selectivity not just between fibrils composed of different proteins but also between fibril polymorphs.

For synucleinopathies, several reviews have covered the efforts to develop PET tracers.^171,301,688–690^ A smaller number of high-affinity and highly selective ligands have been developed for αSyn fibrils compared to tau and Aβ fibrils. Efforts in developing PET ligands with benzoxazole (**34**), oxindole (**55**), chalcone (e.g. **112**), phenothiazine (e.g. **158**), bisquinolines, and bisaryl heterocycle (**123**, **124**) scaffolds yielded tracers with promising binding to *in vitro* αSyn fibrils, but these ligands generally exhibited either insufficient selectivity or poor labelling of biological fibrils.^688–690^

Recently reported αSyn ligands have exhibited more promise as candidate PET tracers, clearly labelling αSyn pathology *in vivo* (Figure 97). **F0502B** (**16**) labels αSyn aggregates in PD brain sections, and **207** bound αSyn fibrils from both PD and MSA.^645,691^ However, **207** also bound tau fibrils from AD, PSP, and CBD.^645^ **C05-05** (**215**) similarly was found to label αSyn pathology in individuals with PD, DLB, and MSA but also labelled NFTs and amyloid plaques in individuals with AD.^648^ **ACI-12589** (**216**) clearly labels αSyn pathology in the brains of MSA patients but less so in PD, although it has a nanomolar dissociation constant for αSyn aggregates from both.^614,692^ Notably, **ACI-12589** showed promising selectivity against both tau and Aβ aggregates. The ligand **SPAL-T-06** (**217**) also led to clear labelling of αSyn deposits in MSA (K = 2.49 nM), but not PD or DLB pathology.^647,665^ The success of **ACI-12589** and **SPAL-T-06** in MSA cases was ascribed to the relative abundance of αSyn aggregates in this condition, and the rapid clearance of these molecules means sufficient concentrations are not sustained to label the less abundant Lewy bodies and neurites in PD and DLB.^647,648,692^

**Figure 97:**
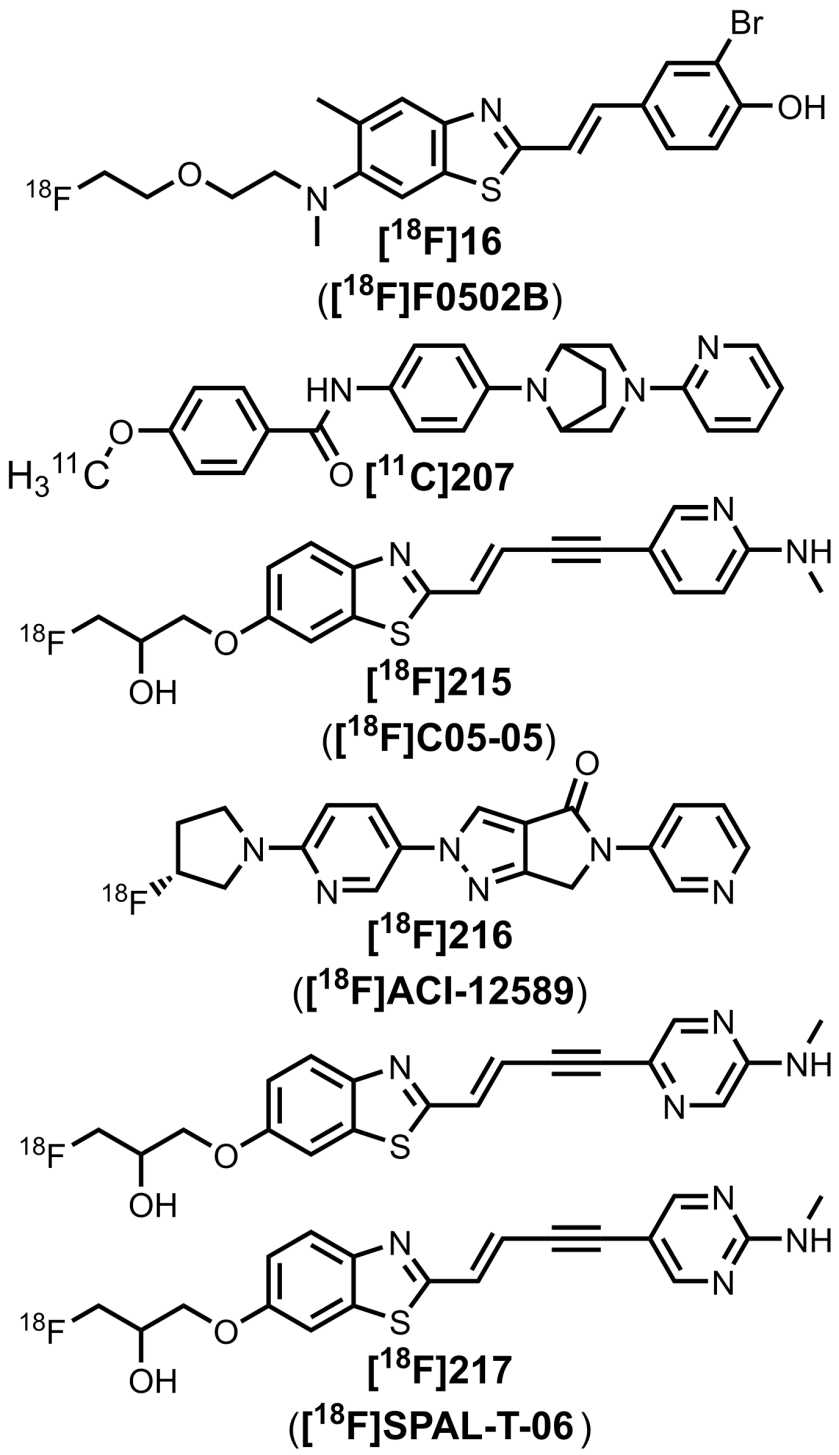
Candidate PET probes developed for imaging αSyn aggregates.

There are therefore a number of potential molecules being tested as PET tracers for non-AD tauopathies and synucleinopathies. For tau, carbazoles (**PI-2620**, **RO-948**, **GTP-1**), isoquinoline derivatives (**MK-6240**, **JNJ-067**), and benzothiazole dienes (**PBB3**, **PM-PBB3**) appear to be the most promising structural classes. There is a particular focus toward developing tau PET probes for non-AD tauopathies. For αSyn, a smaller number of candidate PET ligands have been developed. This slower development is reflective of the greater challenge in developing high-affinity, selective ligands for αSyn.^137^ Nevertheless, several molecules, including the benzothiazole derivatives **C05-05** and **SPAL-T-06**, **ACI-12589**, and **207**, have demonstrated promising early results, particularly for MSA.

## 30 Future Perspectives

A vast array of amyloid-binding ligands has been reported in the literature, with a range of structurally diverse ligands achieving low or sub-nanomolar binding. The overarching aim when developing these ligands is to develop molecules that bind to biological concentrations of protein fibrils and to develop molecules that show selective binding between different protein fibrils. However, relatively few ligands have been developed that are selective between different fibril types. To develop such ligands, there is both a need for methods to accurately assess binding selectivity and a need to more precisely understand binding selectivity in the context of protein fibrils.

Historically, binding selectivity in the field has largely been assessed by measuring the affinities of ligands to fibrils composed of specific proteins, such as αSyn fibrils and Aβ fibrils. Recent research has highlighted that fibrils composed of only a single protein can adopt multiple morphologies and that specific morphologies are associated with different neurodegenerative diseases. Binding selectivity should therefore ideally be assessed by comparing binding to fibrils with specific disease-relevant morphologies, such as αSyn fibrils with a DLB morphology and Aβ fibrils with an AD morphology, or αSyn fibrils with a PD morphology and αSyn fibrils with a MSA morphology.

However, this approach is limited by the practicalities of accessing large amounts of fibrils with disease-specific morphologies. Several avenues of research have the potential to address the difficulty of accessing morphology-specific samples of protein fibrils on useful scales. The discovery of *in vitro* aggregation conditions that replicate *in vivo* morphologies would allow for larger quantities of disease-relevant fibrils to be prepared. Such *in vitro* conditions have already been achieved for the preparation of disease-relevant tau morphologies.^47^ SAAs could also be used to generate disease-relevant morphologies from a small amount of biological samples.^25,693^ However, the morphology of amplified fibrils may differ from those present *in vivo*, as evidenced by the cryo-EM structures reported for amplified αSyn fibrils. Additionally, the SAA protocol may lead to only the most seeding-competent morphology subpopulations being replicated. Accessing disease-relevant fibril morphologies on a scale useful for most assays methods therefore remains an unsolved problem in the field. Alternatively, new screening methods that allow for ligand binding to be assessed using smaller quantities of biological fibrils would also be valuable in addressing this challenge.

The development of selective ligands may also be hampered by the presence of multiple binding sites on a single fibril target. Each binding site has distinct structural features with different SAR requirements for high-affinity binding. Sampling an ensemble of binding sites with binding assays will therefore complicate the development of selective ligands. This challenge can be at least partially mitigated by using competition binding assays to target specific subsets of sites.^137^

Prior work also suggests that ligand-fibril interactions are dominated by the hydrophobic effect.^137^ Ligands that undertake site-specific polar interactions may therefore be attractive leads for developing molecules that selectively bind specific fibril morphologies. Currently, few ligands show selective binding with more than an order of magnitude difference in dissociation constants.^137^

The development of selective ligands would perhaps most significantly benefit from a better understanding of the binding mode of ligands. Recent work has suggested that ligands may bind in a stacked arrangement along the fibril axis.^102^ If these structures represent high-affinity binding modes, then interactions between ligands are an important consideration and may produce cooperative binding.^694^ Such ligand–ligand interactions would be strongly affected by the helical rise and twist angle of the targeted fibril, which varies between different polymorphs and could therefore significantly influence binding selectivity.

The use of multivalent ligands, where two or more ligands are covalently joined with a linker, can also lead to cooperative binding.^434^ If the linker can bridge the distance between binding sites, both ligand headgroups can simultaneously bind, leading to cooperative binding and an enhancement in binding affinity. Designing ligands with cooperative binding that is dependent on the supramolecular structure of fibrils could be a useful strategy to improve the binding selectivity between polymorphs.

Additionally, there remain several aspects related to fibril morphology *in vivo* that are not yet fully understood. What post-translational modifications (PTMs) and bound non-proteinaceous molecules are present *in vivo*, and do these affect ligand binding? What subpopulations of morphologies exist *in vivo*? How do these factors vary within patient populations, and how do they relate to the underlying disease present? Better understanding the answers to these questions will allow for more effective fibril-binding ligands to be prepared for use both as research tools and as potential diagnostic agents.

In summary, the development of selective and high-affinity amyloid-binding ligands is linked closely to our understanding of fibril morphology. Morphology-specific ligands will enable research to answer a range of outstanding questions related to biological protein fibrils, that will in turn facilitate the development of additional morphology-specific ligands. These ligands will help address questions that structural approaches alone cannot and will be useful for both clinical applications and fundamental research.

## Supplementary Material





## Notes

Details of dataset generation and properties; tables of data describing reported fibril structures (PDF)
SMILES and binding assay data (XLSX)
