# Supplementary material for: Ligands for Protein Fibrils of Amyloid-β, α‑Synuclein, and Tau
Source: Chem Rev. 2025 May 6;125(11):5282–348. doi: 10.1021/acs.chemrev.4c00838 (PMC12164286; doi:10.1021/acs.chemrev.4c00838)
Supplement: Supplementary file 1 [file cr4c00838_si_001.pdf]

Ligands for Protein Fibrils of Amyloid-β, α-Synuclein, and Tau

Timothy S. Chisholm[a,*] and Christopher A. Hunter[a]

[a] Yusuf Hamied Department of Chemistry, University of Cambridge, Lensfield Road, Cambridge CB2 1EW, UK.

* Email: tsc42@cam.ac.uk

Supporting Information

Contents

Dataset Generation and Preparation 2

Data Tables 4

References 14

Dataset Generation and Preparation

Protein-ligand binding data were acquired from literature searches using Web of Science and Google Scholar, and the PDBbind and ChEMBL online databases.[1,2] The requirements to be included in this database were 1) a known chemical structure, 2) a structure that did not contain any metal atoms, 3) a K_d, K_i, or IC_{50} measurement, 4) a fibril target related to Aβ, tau, or αSyn, and 5) publication prior to 2024. A total of 4,288 measurements of ligand-amyloid fibril interactions were found for 2,404 unique ligands.[3–472] Details of the ligands, publications, and binding data, including SMILES and DOI, are found in the supporting .csv file.

The attached spreadsheet contains binding measurements for compounds organised in the following columns:

Compound: The name of the reported compound. These names are either taken from the publication, or they are a combination of (a) the publication lead author's name, (b) the year of publication (optional), and (c) the compound number in the publication.
SMILES: The SMILES formula for the reported compound.
DOI: The DOI of the publication containing this binding measurement (if relevant).
Patent number: The number of the patent containing this binding measurement (if relevant).
Kd / nM: The reported dissociation constant from a saturation binding assay. **n.b.** refers to no binding being measured.
Ki / nM: The reported dissociation constant from a competition binding assay. **n.b.** refers to no binding being measured.
K error / nM: The error value provided for the reported K_d or K_i measurement.
IC50 / nM: The reported IC50 from a competition binding assay.
IC50 error / nM: The error value provided for the reported IC50 measurement.
Reporting ligand: If a competition binding assay was performed, this is the ligand displaced in the assay.
Target fibril: The fibril target used in the binding assay. **Ab**: Aβ fibrils (unknown isoform); **Ab(10-43)**: Aβ(10-43) fibrils; **Ab40**: Aβ(1-40) fibrils; **Ab40/Ab42**: fibrils composed of both Aβ(1-40) and Aβ(1-42); **Ab42**: Aβ(1-42) fibrils; **AbZn**: Aβ fibrils (unknown isoform) enriched with Zinc ions; **AD**: fibrils from the brain of patients with Alzheimer's disease (usually in the form of brain homogenates, but sometimes brain sections or isolated fibrils); **AD-Ab**: Aβ fibrils either isolated or enriched from AD brain tissue; **AD-t**: tau fibrils either isolated or enriched from AD brain tissue; **aS**: α-synuclein fibrils; **AD-CAA**: fibrils isolated from cerebral amyloid angiopathy pathology in AD; **DLB**: fibrils from the brain of patients with dementia with Lewy bodies (usually in the form of brain homogenates); **DLB-Ab**: Aβ fibrils either isolated or enriched from DLB brain tissue; **DLB-aS:** α-synuclein fibrils either isolated or enriched from DLB brain tissue; **HC**: brain tissue from healthy patients; **PD**: fibrils from the brain of patients with Parkinson's disease; **PD-aS**: α-synuclein fibrils either isolated or enriched from PD brain tissue; **PiD**: fibrils from the brain of patients with Pick's disease; **PiD-t**: tau fibrils either isolated or enriched from PiD brain tissue; **PSP**: fibrils from the brain of patients with Progressive Supranuclear Palsy; **roAb**: brain

homogenates from a mouse model of AD; **roAb40**: rodent Aβ(1-40) fibrils; **t**: tau fibrils; **Tg mouse-aS**: α-synuclein fibrils from a transgenic mouse.
Target type: The type of target being assayed: f: fibril; b: biological sample; section: brain section.
Target source: Where the fibril target was obtained from (e.g. synthetically, recombinantly, a supplier, a specific brain region).
Sample preparation: A description of where the fibril target was obtained from (e.g. synthetically, recombinantly, or a specific brain region), and how the fibril target was prepared if applicable. This information is taken directly from the publication where available.
Notes: Any notes useful for interpreting the binding measurement.
Assay type: The type of binding assay performed. Typically by measuring fluorescence ("Fluorescence") or using a radiolabelled ligand ("Radioligand") in a saturation or competition assay. Also includes measurements made using autoradiography, ELISA (enzyme-linked immunosorbent assay), ITC (isothermal titration calorimetry), LCMS (liquid chromatography-mass spectrometry), SPR (surface plasmon resonance), UV saturation, and microscale thermophoresis.
Assay conditions: A description of how the binding assay was performed, taken directly from the publication where available.

Structures of fibrils formed from Aβ, tau, or αSyn, were found by searching the RCSB PDB.[3,4,473–565]

Data Tables

Table S1. Details of αSyn fibrils formed *in vitro* that have structures reported on the PDB. Blank entries indicate that no information was given.

Study	Buffer	[αSyn] / μM	Protein Modifications	Additives	pH	T / °C	Agitation	Time	PDB
Chen, S. W. *et al.* **2024**									8RI9
Monistrol, J. *et al.* **2024**	50 mM $NaPO_4$, 100 mM NaCl, 0.05% w/v NaN_3	200			7.3	37	1,000 rpm	7 days	8RQM, 8RRR
Tao, Y. Q. *et al.* **2024**				heparin				1 h	8HZB, 8HZC
Tao, Y. Q. *et al.* **2024**				heparin				3 days	8HZS
Tao, Y. *et al.* **2023**	50 mM Tris, 150 mM NaCl, 0.05% NaN_3	500		0.5 mol.% seeds	7.5	37	900 rpm	5 days	7YNG, 7YNG, 7YNL, 7YNM, 7YNN, 7YNO, 7YNP, 7YNQ, 7YNR, 7YNS, 7YNT, 7WMM
Zhang, S. *et al.* **2023**	50 mM Tris·HCl, 150 mM KCl, 0.05% NaN_3	500		0.5 vol.% seeds	7.5	37	900 rpm	5 days	7YK2
Zhang, S. *et al.* **2023**	50 mM Tris·HCl, 150 mM KCl, 0.05% NaN_3	500		0.5 vol.% seeds	7.5	37	900 rpm	5 days	7YK8
Frieg, B. *et al.* **2022**	50 mM HEPES, 100 mM NaCl	330		POPC/POPA lipids (5:1 lipid:protein ratio)		37	Repeated sonication and incubation	96 h	8ADS, 8ADU, 8ADV, 8ADW, 8AEX, 8A4L
Tao, Y. *et al.* **2022**	50 mM Tris·HCl, 150 mM KCl, 0.05% NaN_3	50		600 μg/mL heparin, 1 mol.% seeds	7.5		900 rpm	5 days	7V4A, 7V4B, 7V4C
Tao, Y. *et al.* **2022**				heparin					7V4D
Hojjatian, A. *et al.* **2020**	10 mM phosphate buffer	70		20 uM tau	7.4	37	250 rpm	1 day	7L7H
Long, H. *et al.* **2021**	50 mM Tris, 150 mM KCl, 0.05% NaN_3	100		0.5 mol.% seeds (E46K mutant αSyn seeds used)	7.5	37	900 rpm	7 days	7C1D
Guerrero-Ferreira, R. *et al.* **2019**	50 mM Tris·HCl, 150 mM KCl	700			7.5	37	600 rpm	7 days	6SST, 6SSX, 6RT0, 6RTB
Li, B. *et al.* **2018**	15 mM tetrabutylphosphonium bromide	300				rt		14-30 days	6CU7, 6CU8
Tuttle, M. D. *et al.* **2016**	50 mM sodium phosphate, 0.1 mM EDTA, 0.02% NaN_3	15 mg/mL			7.4	37	200 rpm	3 weeks	2N0A
Balana, A. T. *et al.* **2024**	50 mM Tris, 150 mM NaCl		Ser87 O-GlcNAc		7.5	37	1,000 rpm	14 days	8GF7
Zhao, Q. *et al.* **2023**	50 mM Tris·HCl, 150 mM KCl, 0.05% w/v NaN_3	100	N-term acetylation	100 uM FeCl3	7.5	37	900 rpm	7 days	7XJX
McGlinchey, R. P. *et al.* **2021**	20 mM NaPi, 140 mM NaCl	180	N-term trunc: αSyn (41-140)		7.4	37	600 rpm	4-5 days	7LC9
Zhao, K. *et al.* **2020**	50 mM Tris, 150 mM KCl, 0.05% NaN3	100	Tyr39 phosphorylation	0.5 vol.% seeds	7.5	37	900 rpm	2 weeks	6L1T, 6L1U
Ni, X. *et al.* **2019**	10 mM NaPi, 140 mM NaCl	100-300	N-term acetylation		7.4	37	600 rpm	4-5 days	6OSJ
Ni, X. *et al.* **2019**	10 mM NaPi, 140 mM NaCl	100-300	N-term acetylation, C-term trunc: αSyn (1-122)		7.4	37	600 rpm	4-5 days	6OSL

Study	Buffer	[αSyn] / μM	Protein Modifications	Additives	pH	T / °C	Agitation	Time	PDB
Ni, X. *et al.* **2019**	10 mM NaPi, 140 mM NaCl	100-300	N-term acetylation, C-term trunc: αSyn (1-103)		7.4	37	600 rpm	4-5 days	6OSM
Guerrero-Ferreira, R. *et al.* **2018**	DPBS	360 uM (5 mg/mL)	C-term trunc: αSyn (1-121)		7.0-7.3	37	1,000 rpm	5 days	6H6B
Li, Y. *et al.* **2018**	50 mM Tris, 150 mM KCl, 0.05% NaN_3	500	N-term acetylation		7.5	37	900 rpm	3 days	6A6B
Sun, C. *et al.* **2023**	15 mM tetrabutylphosphonium bromide	300	A53E			37		2 weeks	7UAK
Yany, Y. *et al.* **2023**	PBS	130	MAAAEKT insertion after residue 22			37	200 rpm	4 days	8CEB, 8CE7
Huang, J. Y.-C. *et al.* **2023**			A53T						7WNZ
Huang, J. Y.-C. *et al.* **2023**			A53T	Calcium ions					7WO0
Sun, Y. *et al.* **2021**	50 mM phosphate buffer, 50 mM NaCl, 0.05% NaN_3	100	G51D	0.5 mol.% seeds	7	37	900 rpm	7 days	7E0F
Zhao, K. *et al.* **2020**	50 mM Tris, 150 mM KCl	100	E46K	0.5 mol.% seeds	7.5	37	900 rpm	7 days	6L4S
Sun, Y. *et al.* **2020**	D-PBS	100	A53T	1 vol.% seeds		37	1,000 rpm	7 days	6LRQ
Boyer, D. R. *et al.* **2020**	15 mM tetrabutylphosphonium bromide	300	E46K			37	shaking	2 weeks	6UFR
Boyer, D. R. *et al.* **2019**	15 mM tetrabutylphosphonium bromide	300	H50Q			37	shaking	2 weeks	6PEO, 6PES

Table S2. Details of αSyn fibrils formed *in vivo* that have structures reported on the PDB.[a]

Study	Pathogenic Origin	PDB
Yang, Y. *et al.* **2023**	JOS (SNCA mutation)	8BQV, 8BQW
Yang, Y. *et al.* **2022**	Lewy fold (PD, PDD, DLB)	8A9L
Schweighauser, M. *et al.* **2020**	MSA	6XYO, 6XYP, 6XYQ

[a] JOS: Juvenile-onset synucleinopathy. SNCA: PD: Parkinson's disease. PDD: Parkinson's disease dementia. MSA: Multiple system atrophy.

Table S3. Details of αSyn fibrils formed using SAAs that have structures reported on the PDB.[a]

Study	Pathogenic Origin of Seeds	Biosample	Seeding method	Monomer	PDB
Dhavale, D. D. *et al.* **2024**	LBD	Brain tissue (caudate)	PMCA	αSyn (1-140)	8FPT
Frieg, B. *et al.* **2022**	PD	Brain tissue (amygdala)	PMCA	N-term acetylated αSyn (1-140)	7OZG
Frieg, B. *et al.* **2022**	MSA	Brain tissue (amygdala)	PMCA	N-term acetylated αSyn (1-140)	7OZH
Lövestam, S. *et al.* **2021**	MSA	Brain tissue (putamen)	RT-QuIC	αSyn (1-140)	7NCA, 7NCG, 7NCH, 7NCI, 7NCJ, 7NCK
Sokratian, A. *et al.* **2022**	HC	CSF	RT-QuIC	αSyn (1-140)	8CYR
Sokratian, A. *et al.* **2022**	DLB	CSF	RT-QuIC	αSyn (1-140)	8CYS, 8CYT, 8CYV, 8CYW, 8CYX, 8CYY, 8CZ0, 8CZ1, 8CZ2, 8CZ3, 8CZ6
Fan, Y *et al.* **2023**	PD (preclinical)	CSF	RT-QuIC	N-term acetylated αSyn (1-140)	8H03, 7V47
Fan, Y *et al.* **2023**	PD (mid stage)	CSF	RT-QuIC	N-term acetylated αSyn (1-140)	7XO0, 7XO1, 7XO2, 7XO3
Fan, Y *et al.* **2023**	PD (late stage)	CSF	RT-QuIC	N-term acetylated αSyn (1-140)	8HO4, 8HO5, 7V48, 7V49

[a] LBD: Lewy body dementia. PMCA: Protein misfolding cyclic amplification. PD: Parkinson's disease. MSA: Multiple system atrophy. RT-QuIC: Real time quaking-induced conversion. HC: Healthy control. CSF: Cerebrospinal fluid. DLB: Dementia with Lewy bodies. PD: Parkinson's disease.

Table S4. Details of tau fibrils formed *in vitro* that have structures reported on the PDB. Blank entries indicate that no information was given.

Study	Protein	Protein Modifications	[tau]	Buffer	Additives	pH	T / °C	Agitation	Time	PDB
Zhang, W. *et al.* **2019**	Tau (1-441)		3.0 mg/mL	30 mM MOPS, 1 mM AEBSF, 4 mM TCEP	heparin (400 μg/mL)	7.2	37		3 days	6QJM, 6QJH, 6QJP
Zhang, W. *et al.* **2019**	Tau 2N3R (1-410)		3.0 mg/mL	30 mM MOPS, 1 mM AEBSF, 4 mM TCEP	heparin (400 μg/mL)	7.2	37		3 days	6QJQ
Louros, N. *et al.* **2024**	Tau (350-362)		200 μM	5 mM HEPES, 10 mM KCl, 5 mM $MgCl_2$, 3 mM TCEP, 0.01% NaN_3		7.2	rt		7 days	8OH2
Louros, N. *et al.* **2024**	Tau (350-362)	N-terminal Fmoc	200 μM	5 mM HEPES, 10 mM KCl, 5 mM $MgCl_2$, 3 mM TCEP, 0.01% NaN_3		7.2	rt		7 days	8OHI, 8OHP, 8OI0
Limorenko, G. *et al.* **2023**	Tau (1-441)		100 μM	10 mM phosphate, 50 mM NaF, 0.5 mM DTT			37	100 rpm	24 h	8R3T
Duan, P. *et al.* **2023**	Tau (297–391)		100 μM	10 mM phosphate buffer, 200 mM $MgCl_2$, 10 mM DTT, 0.02 wt% NaN_3		5	37	150 rpm	3 days	8G58
Lövestam, S. *et al.* **2024**	Tau (297–391)		592 μM (6 mg/mL)	10 mM phosphate buffer, 100 mM $MgCl_2$, 10 mM DTT		7.2	37	200 rpm	12 h	8Q2L, 8Q8E, 8Q8F, 8Q8L, 8Q8M, 8Q8R, 8Q8S, 8Q8C, 8QCP, 8Q27, 8Q2J, 8Q2K, 8Q7F, 8Q7L, 8Q7M, 8Q7T, 8Q88, 8Q8D, 8PPO
Lövestam, S. *et al.* **2024**	Tau (297–391)		592 μM (6 mg/mL)	50 mM phosphate buffer, 150 mM NaCl, 10 mM DTT		7.2	37	200 rpm	12 h	8Q8U, 8Q8V, 8Q8W, 8Q8X, 8Q8Y, 8Q8Z, 8Q97, 8Q98, 8Q99, 8Q9A, 8Q9B, 8Q9C, 8Q9D, 8Q9E, 8Q9F, 8Q9G, 8Q9H, 8Q9I, 8Q9J, 8Q9K, 8Q9L, 8Q9M, 8Q9O, 8Q9R
Li, L. *et al.* **2023**	Tau (263-280)	2nd and 3rd lysine residues acetylated	500 μM	1xPBS	25 μM ThT	7			7 days	8FNZ
El Mammeri, N. *et al.* **2023**	Tau (198 to 399)		4.7 μM	1xPBS, 2 mM DTT	0.125 mg/mL heparin		24	50 rpm	3 days	8G54
El Mammeri, N. *et al.* **2023**	Tau (198 to 399)		4.7 μM	1xPBS, 2 mM DTT	0.125 mg/mL heparin		12	55 rpm	3 days	8G55
Li, X. *et al.* **2022**	Tau (266–391 (3R)) and Tau (297–391)		2 mg/mL	10 mM phosphate buffer, 20 mM DTT, 200 mM $MgCl_2$		7.4	37	orbital shaking	48 h	7YMN
Li, X. *et al.* **2022**	Tau (297–391)		2 mg/mL	10 mM phosphate buffer, 20 mM DTT, 200 mM $MgCl_2$		7.4	37	orbital shaking	48 h	7YPG
Abskharon, R. *et al.* **2022**	Tau (1-441)		50 μM	20 mM ammonium acetate	400 μg/mL RNA	7	37	shaking	3 days	7SP1
Lövestam, S. *et al.* **2021**	Tau (297-394)		4 mg/mL	10 mM PB, 10 mM DTT		7.4		700 rpm	48 h	7QJX

Study	Protein	Protein Modifications	[tau]	Buffer	Additives	pH	T / °C	Agitation	Time	PDB
Lövestam, S. *et al.* **2021**	Tau (266/297–391)		4 mg/mL	10 mM PB, 10 mM DTT, 20 mM $MgCl_2$, 100 mM NaCl		7.4		200 rpm	48 h	7QKJ
Lövestam, S. *et al.* **2021**	Tau (258-391)		4 mg/mL	10 mM PB, 10 mM DTT, 0.1% NaN_3		7.4		700 rpm	48 h	7QKH
Lövestam, S. *et al.* **2021**	Tau (266/297–391)		4 mg/mL	10 mM PB, 10 mM DTT, 10 mM $NaHCO_3$, 100 mM NaCl		7.4		200 rpm	48 h	7R4T
Lövestam, S. *et al.* **2021**	Tau (266/297–391)		4 mg/mL	10 mM PB, 10 mM DTT, 200 mM KCl		7.4		200 rpm	48 h	7R5H
Lövestam, S. *et al.* **2021**	Tau (297–391)		4 mg/mL	10 mM PB, 10 mM DTT		7.4		200 rpm	48 h	7QJV
Lövestam, S. *et al.* **2021**	Tau (266/297–391)		4 mg/mL	10 mM PB, 10 mM DTT, 200 mM LiCl		7.4		200 rpm	48 h	7QJY
Lövestam, S. *et al.* **2021**	Tau (266/297–391)		4 mg/mL	10 mM PB, 10 mM DTT, 200 mM LiCl		7.4		200 rpm	48 h	7QJZ
Lövestam, S. *et al.* **2021**	Tau (297–394)		4 mg/mL	PBS, 10 mM DTT		7.4		700 rpm	48 h	7QK1
Lövestam, S. *et al.* **2021**	Tau (300-391)		4 mg/mL	PBS, 10 mM DTT		7.4		700 rpm	48 h	7QK2
Lövestam, S. *et al.* **2021**	Tau (258-391)		4 mg/mL	10 mM PB, 10 mM DTT		7.4		700 rpm	48 h	7QK3
Lövestam, S. *et al.* **2021**	Tau (266/297–391)		4 mg/mL	10 mM PB, 10 mM DTT, 200 mM KCl		7.4		200 rpm	48 h	7QK5
Lövestam, S. *et al.* **2021**	Tau (258-391)		4 mg/mL	10 mM PB, 10 mM DTT	300 µg/µL heparan sµLphate	7.4		700 rpm	48 h	7QK6
Lövestam, S. *et al.* **2021**	Tau (266/297–391)		4 mg/mL	10 mM PB, 10 mM DTT, 200 µM $CuCl_2$		7.4		200 rpm	48 h	7QKF
Lövestam, S. *et al.* **2021**	Tau (258-391)		4 mg/mL	10 mM PB, 10 mM DTT	5 mM phospho-glycerate	7.4		700 rpm	48 h	7QKG
Lövestam, S. *et al.* **2021**	Tau (297-408)	S396D, S400D, T403D, S404D	4 mg/mL	10 mM PB, 10 mM DTT, 200 mM $MgCl_2$		7.4		200 rpm	48 h	7QKI
Lövestam, S. *et al.* **2021**	Tau (258-391)		4 mg/mL	10 mM PB, 10 mM DTT	5 mM phospho-glycerate	7.4		700 rpm	48 h	7QKK
Lövestam, S. *et al.* **2021**	Tau (266/297–391)		4 mg/mL	10 mM PB, 10 mM DTT, 200 µM $ZnCl_2$		7.4		200 rpm	48 h	7QKL
Lövestam, S. *et al.* **2021**	Tau (266–391)	S356D	4 mg/mL	10 mM PB, 10 mM DTT, 200 mM NaCl		7.4		200 rpm	48 h	7QKM
Lövestam, S. *et al.* **2021**	Tau (266/297–391)		4 mg/mL	10 mM PB, 10 mM DTT, 20 mM $MgCl_2$, 100 mM NaCl		7.4		200 rpm	48 h	7QKU
Lövestam, S. *et al.* **2021**	Tau (266/297–391)		4 mg/mL	10 mM PB, 10 mM DTT, 10 mM $MgSO_4$, 100 mM NaCl		7.4		200 rpm	48 h	7QKV
Lövestam, S. *et al.* **2021**	Tau (266–391)	S356D	4 mg/mL	10 mM PB, 10 mM DTT, 200 mM KCl		7.4		200 rpm	48 h	7QKW
Lövestam, S. *et al.* **2021**	Tau (266/297–391)		4 mg/mL	10 mM PB, 10 mM DTT, 10 mM $MgSO_4$, 100 mM NaCl		7.4		200 rpm	48 h	7QKX
Lövestam, S. *et al.* **2021**	Tau (0N4R)		4 mg/mL	PBS, 5 mM TCEP	5 mM L-phos-phoserine	7.4		200 rpm	96 h	7QKY
Lövestam, S. *et al.* **2021**	Tau (305–379)		4 mg/mL	10 mM PB, 10 mM DTT, 200 mM $MgCl_2$		7.4		200 rpm	48 h	7QKZ
Lövestam, S. *et al.* **2021**	Tau (266/297–391)		4 mg/mL	10 mM PB, 10 mM DTT, 10 mM $MgSO_4$, 100 mM NaCl		7.4		200 rpm	48 h	7QL0

Study	Protein	Protein Modifications	[tau]	Buffer	Additives	pH	T / °C	Agitation	Time	PDB
Lövestam, S. *et al.* **2021**	Tau (266-391)		4 mg/mL	PBS, 10 mM DTT		7.4		200 rpm	48 h	7QL1
Lövestam, S. *et al.* **2021**	Tau (244-391)		4 mg/mL	10 mM PB, 10 mM DTT, 5 mM $Na_4P_2O_7$				200 rpm	76 h	7QL2
Lövestam, S. *et al.* **2021**	Tau (266/297–391)		4 mg/mL	10 mM PB, 10 mM DTT, 200 mM NaCl		7.4		200 rpm	48 h	7QL3
Lövestam, S. *et al.* **2021**	not specified		4 mg/mL							7QL4
Lövestam, S. *et al.* **2021**	Tau (266/297–391)		4 mg/mL	10 mM PB, 10 mM DTT, 200 mM NaCl		7.4		200 rpm	48 h	7QJW
Seidler, P. M. *et al.* **2018.**	KVQIINKKLD			Crystallisation						5V5B
Seidler, P. M. *et al.* **2018.**	VQIINK			Vapour diffusion						5V5C
Wiltzius, J. J.W. *et al.* **2009**	VQIVYK			Vapour diffusion						4NP8

Table S5. Details of tau fibrils formed *in vivo* that have structures reported on the PDB.[a]

Study	Pathogenic Origin	Dominant tau isoform	PDB
Stern, A. M. *et al.* **2023**	AD (PHF)	3R + 4R	8AZU
Fowler, S. L. *et al.* **2023** (bioRxiv)	AD (PHF)	3R + 4R	8BGV
Seidler, P. M. *et al.* **2022**	AD (PHF)	3R + 4R	7UPE
Shi, Y. *et al.* **2021**	AD (PHF)	3R + 4R	7NRQ
Arakhamia, T. *et al.* **2020**	AD (PHF)	3R + 4R	6VHL
Falcon, B. *et al.* **2018**	AD (PHF)	3R + 4R	6HRE
Fitzpatrick, A. W.P. *et al.* **2017**	AD (PHF)	3R + 4R	5O3L
Fitzpatrick, A. W.P. *et al.* **2017**	AD (PHF)	3R + 4R	5O3O[b]
Fowler, S. L. *et al.* **2023** (bioRxiv)	AD (PHF, from EV)	3R + 4R	8BGS
Falcon, B. *et al.* **2018**	AD (SF)	3R + 4R	6HRF
Shi, Y. *et al.* **2021**	AD (SF)	3R + 4R	7NRS, 7NRT
Arakhamia, T. *et al.* **2020**	AD (SF)	3R + 4R	6VI3
Fitzpatrick, A. W.P. *et al.* **2017**	AD (SF)	3R + 4R	5O3T
Hallinan, G. I. *et al.* **2021**	PrP-CAA	3R + 4R	7MKF, 7MKG
Hallinan, G. I. *et al.* **2021**	GSS	3R + 4R	7MKH
Lövestam, S. *et al.* **2024**	CTE	3R + 4R	8QJJ
Falcon, B. *et al.* **2019**	CTE	3R + 4R	6NWP, 6NWQ
Qi, C. *et al.* **2023**	SSPE	3R + 4R	8CAQ, 8CAX
Schweighauser, M. *et al.* **2023**	PiD	3R	8P34[c]
Falcon, B. *et al.* **2018**	PiD	3R	6GX5
Chang, A. *et al.* **2022**	PSP	4R	7U0Z
Shi, Y. *et al.* **2021**	PSP	4R	7P65
Shi, Y. *et al.* **2021**	GGT	4R	7P66, 7P67, 7P68
Shi, Y. *et al.* **2021**	LNT (GPT fold)	4R	7P6A, 7P6B, 7P6C
Shi, Y. *et al.* **2021**	AGD	4R	7P6D, 7P6E
Arakhamia, T. *et al.* **2020**	CBD	4R	6VH7
Arakhamia, T. *et al.* **2020**	CBD	4R	6VHA
Zhang, W. *et al.* **2020**	CBD	4R	6TJO, 6TJX

[a] AD: Alzheimer's disease. PHF: Paired helical filament. SF: Straight filament. EV: Extracellular vesicle. PrP-CAA: Prion protein cerebral amyloid angiopathy. GSS: Gerstmann–Sträussler–Scheinker. CTE: Chronic traumatic encephalopathy. SSPE: Subacute sclerosing panencephalitis. CBD: Corticobasal degeneration. PiD: Pick's disease. PSP: Progressive supranuclear palsy. GGT: Globular glial tauopathy. LNT: Limbic-predominant neuronal inclusion body 4R tauopathy. GPT: GGT-PSP-Tau. AGD: Argyrophilic grain disease. [b] Pronase-treated. [c] ΔK281.

Table S6. Details of tau fibrils formed in mice or cells that have structures reported on the PDB.[a]

Study	Pathogenic Origin	Dominant tau isoform	Notes	PDB
Schweighauser, M. *et al.* **2023**	Transgenic mouse	4R	P301S	8Q92, 8Q96
Tarutani, A. *et al.* **2023**	AD	3R + 4R	Seeded in SH-SY5Y cells	8ORE
Tarutani, A. *et al.* **2023**	CBD	4R	Seeded in SH-SY5Y cells	8ORF, 8ORG

[a] AD: Alzheimer's disease. CBD: Corticobasal degeneration.

Table S7. Details of tau fibrils formed *in vivo* with bound small molecule ligands that have structures reported on the PDB.[a]

Study	Pathogenic Origin	Dominant tau isoform	Modification	PDB
Merz, G. E. *et al.* **2023**	AD (PHF)	3R + 4R	Bound GTP-1	8FμG
Shi, Y. *et al.* **2023**	CTE	3R + 4R	Bound Flortaucipir	8BYN
Seidler, P. M. *et al.* **2022**	AD (PHF)	3R + 4R	Bound EGCG	7UPF, 7UPG
Shi, Y. *et al.* **2021**	AD (PHF)	3R + 4R	Bound APN-1607	7NRV
Shi, Y. *et al.* **2021**	AD (SF)	3R + 4R	Bound APN-1607	7NRX

[a] AD: Alzheimer's disease. PHF: Paired helical filament. SF: Straight filament. CTE: Chronic traumatic encephalopathy.

Table S8. Details of Aβ fibrils formed *in vitro* that have structures reported on the PDB. Blank entries indicate that no information was given.

Study	Protein	Protein Modifications	[Aβ] / μM	Buffer	Additives	pH	T / °C	Agitation	Time	PDB
Frieg, B. *et al.* **2024**	Aβ(1-40)		20	NaPi	600 uM DMPG vesicles	6.5	37	Quiescent	1-2 days	8OVK, 8OVM, 8OWD, 8OWE, 8OWJ, 8OWK
Pfeiffer, P. B. *et al.* **2024**	Aβ(1-40)		23 (0.1 mg/mL)	100 mM phosphate buffer		7.3	37		72	8OT1, 8OT3
Pfeiffer, P. B. *et al.* **2024**	Aβ(1-40)		23 (0.1 mg/mL)	100 mM phosphate buffer	5% (w/w) Aβ fibrils extracted from AD meningeal tissue	7.3	37		72	8OT4
Yagi-Utsumi, M. *et al.* **2023**	Aβ(1-40)		500	5 mM potassium phosphate buffer	GM1/DMPC vesicles	7.4				7Y8Q
Ghosh, U. *et al.* **2021**	Aβ(1-40)		100	10 mM sodium phosphate buffer, 0.01% w/v NaN_3	5-9% seeds from AD cortical tissue	7.4	24		2 days	6W0O
Cerofolini, L. *et al.* **2020**	Aβ(1-40)		100	50 mM ammonium acetate		8.5	37	950 rpm	5 weeks	6TI5
Hu, Z. W. *et al.* **2019**	Aβ(1-40)	Ser8-phosphorylated	50	20 mM phosphate buffer, 0.05% NaN_3	10 mol.% seeds	7.4	37	quiescent	72 h	6OC9
Sgourakis, N. G. *et al.* **2015**	Aβ(1-40)	D23N	100	10 mM phosphate buffer, 0.01% NaN_3	10% seeds	7.4	4		4 hours	2MPZ
Schütz, A. K. *et al.* **2014**	Aβ(1-40)	E22Δ	60	10 mM phosphate buffer, 100 mM NaCl		7.4	37	700 rpm	80 min	2MVX
Cerofolini, L. *et al.* **2020**	Aβ(1-40) + Aβ(1-42)		100	50 mM ammonium acetate		8.5	37	950 rpm	5 weeks	6TI6, 6TI7
Liu, D. *et al.* **2021**	Aβ(1-42)	Tyr10 O-Glycosylation (Galβ1-3GalNAcα)	200	1xPBS			37		3 days	7F29
Gremer, L. *et al.* **2017**	Aβ(1-42)			30 vol.% MeCN in H2O	0.1 vol.% TFA		rt	quiescent	8 weeks	5OQV
Xiao, Y. *et al.* **2015**	Aβ(1-42)		50	10 mM phosphate buffer	5% (w/w) seeds	7.4	rt	slow rotation	1 day - 1 week	2MXU
Griner S. L. *et al.* **2019**	Aβ(16-26)	D23N		Crystallisation: 0.1 M Tris, 0.2 M magnesium formate, 20% DMSO, 15% isopropanol		8	rt	quiescent	4 days - 2 weeks	6O4J
Warmack, R. A. *et al.* **2019**	Aβ(20-34)	L-isoaspartate 23	1,600	Crystallisation: 50 mM Tris-HCl, 150 mM NaCl (TBS), 1% DMSO	2% seeds	7.6	37	shaken	2 days	6NB9
Warmack, R. A. *et al.* **2019**	Aβ(20-34)		3,200	Crystallisation: 50 mM Tris-HCl, 150 mM NaCl (TBS), 1% DMSO	2% seeds	7.5	37	shaken	2 days	6OIZ
Krotee, P. *et al.* **2017**	Aβ(24-34)		1,700 (10 mg/mL)	Crystallisation: 25 mM citric acid, 5% DMSO		4	37	shaking	2 days - 1 week	5VOS
Do, T. D. *et al.* **2018**	Aβ(19-24)		2,300 (10 mg/mL)	Hanging-drop crystallisation: 0.2 M Ammonium nitrate, 20% w/v Polyethylene glycol 3,350					2-3 days	5TXD
Do, T. D. *et al.* **2018**	Aβ(27-32)		2,300 (10 mg/mL)	Hanging-drop crystallisation: .2 M Potassium phosphate dibasic, 20% w/v Polyethylene glycol 3,350					2-3 days	5TXJ

Table S9. Details of Aβ fibrils formed *in vivo* that have structures reported on the PDB.[a]

Study	Protein	Protein Modifications	Pathological origin	PDB
Fu, Z. *et al.* **2024**	Aβ(1-40)		CAA (vessels in brain cortices)	8FF2
Fu, Z. *et al.* **2024**	Aβ(1-40)	E22Q	CAA (familial Dutch-type) (vessels in brain cortices)	8FF3
Yang, Y. *et al.* **2023**	Aβ(1-40)		AD and CAA (leptomeninges)	8QN6, 8QN7
Yang, Y. *et al.* **2023**	Aβ(1-42)	E22G	AD (frontal cortex)	8BFZ
Yang, Y. *et al.* **2023**	Aβ(1-40)	E22G	AD (frontal cortex)	8BG0
Stern, A. M. *et al.* **2023**	Aβ(1-42)		AD (cortex)	8AZS, 8AZT
Yang, Y. *et al.* **2022**	Aβ(1-42)		AD (frontal cortex)	7Q4B
Yang, Y. *et al.* **2022**	Aβ(1-42)		Pathological aging (PA) (frontal cortex)	7Q4M
Kollmer, M. *et al.* **2019**	Aβ(1-40)		AD (meningeal)	6SHS
Lu, J.-X. *et al.* **2013**	Aβ(1-40)		AD (grey matter)	2M4J

[a] AD: Alzheimer's disease. CAA: Cerebral amyloid angiopathy. PA: Pathological aging.

Table S10. Details of Aβ fibrils formed *in vivo* in transgenic mouse models that have structures reported on the PDB.

Study	Protein Modifications	Pathological origin	PDB
Zielinksi, M. *et al.* **2023**		Transgenic mouse (APP23)	8OL2
Zielinksi, M. *et al.* **2023**		Transgenic mouse (APP/PS1)	8OL3
Zielinksi, M. *et al.* **2023**		Transgenic mouse (ARTE10)	8OL5, 8OLO
Zielinksi, M. *et al.* **2023**		Transgenic mouse (tgAPPSwe)	8OL6
Zielinksi, M. *et al.* **2023**	E22G	Transgenic mouse (tg-APPArcSwe)	8OL7
Zielinksi, M. *et al.* **2023**	E22Q, D23N	Transgenic mouse (tg-SwDI)	8OLG, 8OLN, 8OLQ
Leistner, C. *et al.* **2023**	E22G	Transgenic mouse (App NL-G-F)	8BFA, 8BFB
Yang, Y. *et al.* **2023**	E22G	Transgenic mouse (App NL-G-F)	8BG9

References

(1) Wang, R.; Fang, X.; Lu, Y.; Wang, S. The PDBbind Database: Collection of Binding Affinities for Protein−Ligand Complexes with Known Three-Dimensional Structures. *J. Med. Chem.* **2004**, *47* (12), 2977–2980. DOI: 10.1021/jm030580l.

(2) Gaulton, A.; Hersey, A.; Nowotka, M.; Bento, A. P.; Chambers, J.; Mendez, D.; Mutowo, P.; Atkinson, F.; Bellis, L. J.; Cibrián-Uhalte, E.; Davies, M.; Dedman, N.; Karlsson, A.; Magariños, M. P.; Overington, J. P.; Papadatos, G.; Smit, I.; Leach, A. R. The ChEMBL Database in 2017. *Nucleic Acids Res.* **2017**, *45* (D1), D945–D954. DOI: 10.1093/nar/gkw1074.

(3) Seidler, P. M.; Boyer, D. R.; Sawaya, M. R.; Ge, P.; Shin, W. S.; DeTure, M. A.; Dickson, D. W.; Jiang, L.; Eisenberg, D. S. CryoEM Reveals How the Small Molecule EGCG Binds to Alzheimer's Brain-Derived Tau Fibrils and Initiates Fibril Disaggregation. *Nat. Commun.* **2022**, *13*, 5451. DOI: 10.1101/2020.05.29.124537.

(4) Tao, Y.; Xia, W.; Zhao, Q.; Xiang, H.; Han, C.; Zhang, S.; Gu, W.; Tang, W.; Li, Y.; Tan, L.; Li, D.; Liu, C. Structural Mechanism for Specific Binding of Chemical Compounds to Amyloid Fibrils. *Nat. Chem. Biol.* **2023**, 1–11. DOI: 10.1038/s41589-023-01370-x.

(5) Alzghool, O. M.; van Dongen, G.; van de Giessen, E.; Schoonmade, L.; Beaino, W. α-Synuclein Radiotracer Development and In Vivo Imaging: Recent Advancements and New Perspectives. *Mov. Disord.* **2022**, *37* (5), 936–948. DOI: 10.1002/mds.28984.

(6) Aliyan, A.; Cook, N. P.; Martí, A. A. Interrogating Amyloid Aggregates Using Fluorescent Probes. *Chem. Rev.* **2019**, *119* (23), 11819–11856. DOI: 10.1021/acs.chemrev.9b00404.

(7) Hsieh, C. J.; Ferrie, J. J.; Xu, K.; Lee, I.; Graham, T. J. A.; Tu, Z.; Yu, J.; Dhavale, D.; Kotzbauer, P.; Petersson, E. J.; Mach, R. H. Alpha Synuclein Fibrils Contain Multiple Binding Sites for Small Molecules. *ACS Chem. Neurosci.* **2018**, *9* (11), 2521–2527. DOI: 10.1021/acschemneuro.8b00177.

(8) Zhuang, Z. P.; Kung, M. P.; Hou, C.; Skovronsky, D. M.; Gur, T. L.; Plössl, K.; Trojanowski, J. Q.; Lee, V. M. Y.; Kung, H. F. Radioiodinated Styrylbenzenes and Thioflavins as Probes for Amyloid Aggregates. *J. Med. Chem.* **2001**, *44* (12), 1905–1914. DOI: 10.1021/jm010045q.

(9) Chisholm, T. S.; Hunter, C. A. Ligand Profiling to Characterize Different Polymorphic Forms of α-Synuclein Aggregates. *J. Am. Chem. Soc.* **2023**, *145* (49), 27030–27037. DOI: 10.1021/jacs.3c10521.

(10) Chisholm, T. S.; Melki, R.; Hunter, C. Ligand Profiling as a Diagnostic Tool to Differentiate Patient-Derived α-Synuclein Polymorphs. *ACS Chem Neurosci* **2024**, *15* (10), 2080–2088. DOI: 10.1021/acschemneuro.4c00178.

(11) Kung, H. F.; Choi, S. R.; Qu, W.; Zhang, W.; Skovronsky, D. 18 F Stilbenes and Styrylpyridines for PET Imaging of Aβ Plaques in Alzheimer's Disease: A Miniperspective. *J. Med. Chem.* **2010**, *53* (3), 933–941. DOI: 10.1021/jm901039z.

(12) Sun, X.; Admane, P.; Starosolski, Z. A.; Eriksen, J. L.; Annapragada, A. V.; Tanifum, E. A. 1-Indanone and 1,3-Indandione Derivatives as Ligands for Misfolded α-Synuclein Aggregates. *ChemMedChem* **2022**, *17* (2), e202100611. DOI: 10.1002/cmdc.202100611.

(13) Kudo, Y.; Okamura, N.; Furumoto, S.; Tashiro, M.; Furukawa, K.; Maruyama, M.; Itoh, M.; Iwata, R.; Yanai, K.; Arai, H. 2-(2-[2-Dimethylaminothiazol-5-Yl]Ethenyl)-6-(2-[Fluoro]Ethoxy)Benzoxazole: A Novel PET Agent for in Vivo Detection of Dense Amyloid Plaques in Alzheimer's Disease Patients. *J. Nucl. Med.* **2007**, *48* (4), 553–561. DOI: 10.2967/jnumed.106.037556.

(14) Wang, Y.; Huynh, T. T.; Bandara, N.; Cho, H.-J.; Rogers, B. E.; Mirica, L. M. 2-(4-Hydroxyphenyl)Benzothiazole Dicarboxylate Ester TACN Chelators for 64Cu PET Imaging in Alzheimer's Disease. *Dalton Trans.* **2022**, *51* (3), 1216–1224. DOI: 10.1039/D1DT02767K.

(15) Alagille, D.; Dacosta, H.; Baldwin, R. M.; Tamagnan, G. D. 2-Arylimidazo[2,1-b]Benzothiazoles: A New Family of Amyloid Binding Agents with Potential for PET and SPECT Imaging of Alzheimer's Brain. *Bioorg. Med. Chem. Lett.* **2011**, *21* (10), 2966–2968. DOI: 10.1016/j.bmcl.2011.03.052.

(16) Agdeppa, E. D.; Kepe, V.; Liu, J.; Small, G. W.; Huang, S. C.; Petrič, A.; Satyamurthy, N.; Barrio, J. R. 2-Dialkylamino-6-Acylmalononitrile Substituted Naphthalenes (DDNP Analogs): Novel Diagnostic and Therapeutic Tools in Alzheimer's Disease. *Mol. Imaging Biol.* **2003**, *5* (6), 404–417. DOI: 10.1016/j.mibio.2003.09.010.
(17) Gu, J.; Anumala, U. R.; Lo Monte, F.; Kramer, T.; Heyny Von Haußen, R.; Hölzer, J.; Goetschy-Meyer, V.; Mall, G.; Hilger, I.; Czech, C.; Schmidt, B. 2-Styrylindolium Based Fluorescent Probes Visualize Neurofibrillary Tangles in Alzheimer's Disease. *Bioorg. Med. Chem. Lett.* **2012**, *22* (24), 7667–7671. DOI: 10.1016/j.bmcl.2012.09.109.
(18) Telpoukhovskaia, M. A.; Rodríguez-Rodríguez, C.; Cawthray, J. F.; Scott, L. E.; Page, B. D. G.; Alí-Torres, J.; Sodupe, M.; Bailey, G. A.; Patrick, B. O.; Orvig, C. 3-Hydroxy-4-Pyridinone Derivatives as Metal Ion and Amyloid Binding Agents†. *Metallomics* **2014**, *6* (2), 249–262. DOI: 10.1039/c3mt00135k.
(19) Nan, D. D.; Gan, C. S.; Wang, C. W.; Qiao, J. P.; Wang, X. M.; Zhou, J. N. 6-Methoxy-Indanone Derivatives as Potential Probes for β-Amyloid Plaques in Alzheimer's Disease. *Eur. J. Med. Chem.* **2016**, *124*, 117–128. DOI: 10.1016/j.ejmech.2016.07.069.
(20) Maurer, A.; Leonov, A.; Ryazanov, S.; Herfert, K.; Kuebler, L.; Buss, S.; Schmidt, F.; Weckbecker, D.; Linder, R.; Bender, D.; Giese, A.; Pichler, B. J.; Griesinger, C. 11C Radiolabeling of Anle253b: A Putative PET Tracer for Parkinson's Disease That Binds to α-Synuclein Fibrils in Vitro and Crosses the Blood-Brain Barrier. *ChemMedChem* **2020**, *15* (5), 411–415. DOI: 10.1002/cmdc.201900689.
(21) Ono, M.; Wilson, A.; Nobrega, J.; Westaway, D.; Verhoeff, P.; Zhuang, Z. P.; Kung, M. P.; Kung, H. F. 11C-Labeled Stilbene Derivatives as Aβ-Aggregate-Specific PET Imaging Agents for Alzheimer's Disease. *Nucl. Med. Biol.* **2003**, *30* (6), 565–571. DOI: 10.1016/S0969-8051(03)00049-0.
(22) Serdons, K.; Verduyckt, T.; Vanderghinste, D.; Borghgraef, P.; Cleynhens, J.; Van Leuven, F.; Kung, H.; Bormans, G.; Verbruggen, A. 11C-Labelled PIB Analogues as Potential Tracer Agents for in Vivo Imaging of Amyloid β in Alzheimer's Disease. *Eur. J. Med. Chem.* **2009**, *44* (4), 1415–1426. DOI: 10.1016/j.ejmech.2008.09.038.
(23) Kuebler, L.; Buss, S.; Leonov, A.; Ryazanov, S.; Schmidt, F.; Maurer, A.; Weckbecker, D.; Landau, A. M.; Lillethorup, T. P.; Bleher, D.; Saw, R. S.; Pichler, B. J.; Griesinger, C.; Giese, A.; Herfert, K. [11C]MODAG-001—towards a PET Tracer Targeting α-Synuclein Aggregates. *Eur. J. Nucl. Med. Mol. Imaging* **2021**, *48* (6), 1759–1772. DOI: 10.1007/s00259-020-05133-x.
(24) Klunk, W. E.; Jr, C. A. M.; Wang, Y. Thioflavin Derivatives for Use in Antemortem Diagnosis of Alzheimer's Disease and in Vivo Imaging and Prevention of Amyloid Deposition. US10137210B2, 2018.
(25) Kung, H. F.; Choi, S. R.; Qu, W.; Zhang, W.; Skovronsky, D. 18 F Stilbenes and Styrylpyridines for PET Imaging of Aβ Plaques in Alzheimer's Disease: A Miniperspective. *J. Med. Chem.* **2010**, *53* (3), 933–941. DOI: 10.1021/jm901039z.
(26) Sanabria Bohórquez, S.; Marik, J.; Ogasawara, A.; Tinianow, J. N.; Gill, H. S.; Barret, O.; Tamagnan, G.; Alagille, D.; Ayalon, G.; Manser, P.; Bengtsson, T.; Ward, M.; Williams, S.-P.; Kerchner, G. A.; Seibyl, J. P.; Marek, K.; Weimer, R. M. [8F]GTP1 (Genentech Tau Probe 1), a Radioligand for Detecting Neurofibrillary Tangle Tau Pathology in Alzheimer's Disease. *Eur. J. Nucl. Med. Mol. Imaging* **2019**, *46*, 2077–2089. DOI: 10.1007/s00259-019-04399-0.
(27) Xia, C.-F.; Arteaga, J.; Chen, G.; Gangadharmath, U.; Gomez, L. F.; Kasi, D.; Lam, C.; Liang, Q.; Liu, C.; Mocharla, V. P.; Mu, F.; Sinha, A.; Su, H.; Szardenings, A. K.; Walsh, J. C.; Wang, E.; Yu, C.; Zhang, W.; Zhao, T.; Kolb, H. C. [^{18}F]T807, a Novel Tau Positron Emission Tomography Imaging Agent for Alzheimer's Disease. *Alzheimers Dement.* **2013**, *9* (6), 666–676. DOI: 10.1016/j.jalz.2012.11.008.
(28) Yu, P.; Cui, M.; Wang, X.; Zhang, X.; Li, Z.; Yang, Y.; Jia, J.; Zhang, J.; Ono, M.; Saji, H.; Jia, H.; Liu, B. 18F-Labeled 2-Phenylquinoxaline Derivatives as Potential Positron Emission Tomography Probes for in Vivo Imaging of β-Amyloid Plaques. *Eur. J. Med. Chem.* **2012**, *57*, 51–58. DOI: 10.1016/j.ejmech.2012.08.031.
(29) Li, Z.; Zhang, X.; Zhang, X.; Cui, M.; Lu, J.; Pan, X.; Zhang, X. 18F-Labeled Benzyldiamine Derivatives as Novel Flexible Probes for Positron Emission Tomography of Cerebral β-Amyloid Plaques. *J. Med. Chem.* **2016**, *59*, 10577–10585. DOI: 10.1021/acs.jmedchem.6b01063.

(30) Ono, M.; Watanabe, R.; Kawashima, H.; Kawai, T.; Watanabe, H.; Haratake, M.; Saji, H.; Nakayama, M. 18F-Labeled Flavones for in Vivo Imaging of β-Amyloid Plaques in Alzheimer's Brains. *Bioorg. Med. Chem.* **2009**, *17* (5), 2069–2076. DOI: 10.1016/j.bmc.2009.01.025.

(31) Matsumura, K.; Ono, M.; Kimura, H.; Ueda, M.; Nakamoto, Y.; Togashi, K.; Okamoto, Y.; Ihara, M.; Takahashi, R.; Saji, H. 18F-Labeled Phenyldiazenyl Benzothiazole for in Vivo Imaging of Neurofibrillary Tangles in Alzheimer's Disease Brains. *ACS Med. Chem. Lett.* **2012**, *3* (1), 58–62. DOI: 10.1021/ml200230e.

(32) Filippi, L.; Chiaravalloti, A.; Bagni, O.; Schillaci, O. 18F-Labeled Radiopharmaceuticals for the Molecular Neuroimaging of Amyloid Plaques in Alzheimer's Disease. *Am. J. Nucl. Med. Mol. Imaging* **2018**, *8* (4), 268–281.

(33) Zhang, W.; Kung, M. P.; Oya, S.; Hou, C.; Kung, H. F. 18F-Labeled Styrylpyridines as PET Agents for Amyloid Plaque Imaging. *Nucl. Med. Biol.* **2007**, *34* (1), 89–97. DOI: 10.1016/j.nucmedbio.2006.10.003.

(34) Lee, J. H.; Byeon, S. R.; Kim, Y. S.; Lim, S. J.; Oh, S. J.; Moon, D. H.; Yoo, K. H.; Chung, B. Y.; Kim, D. J. [18F]-Labeled Isoindol-1-One and Isoindol-1,3-Dione Derivatives as Potential PET Imaging Agents for Detection of β-Amyloid Fibrils. *Bioorg. Med. Chem. Lett.* **2008**, *18*, 5701–5704. DOI: 10.1016/j.bmcl.2008.05.019.

(35) Rivera-Marrero, S.; Fernández-Maza, L.; León-Chaviano, S.; Sablón-Carrazana, M.; Bencomo-Martínez, A.; Perera-Pintado, A.; Prats-Capote, A.; Zoppolo, F.; Kreimerman, I.; Pardo, T.; Reyes, L.; Balcerzyk, M.; Dubed-Bandomo, G.; Mercerón-Martínez, D.; Espinosa-Rodríguez, L. A.; Engler, H.; Savio, E.; Rodríguez-Tanty, C. [18F]Amylovis as a Potential PET Probe for β-Amyloid Plaque: Synthesis, In Silico, In Vitro and In Vivo Evaluations. *Curr. Radiopharm.* **2019**, *12* (1), 58–71. DOI: 10.2174/1874471012666190102165053.

(36) Higuchi, M.; Iwata, N.; Matsuba, Y.; Sato, K.; Sasamoto, K.; Saido, T. C. 19F and 1H MRI Detection of Amyloid β Plaques in Vivo. *Nat. Neurosci.* **2005**, *8* (4), 527–533. DOI: 10.1038/nn1422.

(37) Dezutter, N. A.; Dom, R. J.; De Groot, T. J.; Bormans, G. M.; Verbruggen, A. M. 99mTC-MAMA-Chrysamine G, a Probe for Beta-Amyloid Protein of Alzheimer's Disease. *Eur. J. Nucl. Med.* **1999**, *26* (11), 1392–1399. DOI: 10.1007/s002590050470.

(38) Nguyen, G. A. H.; Liang, C.; Mukherjee, J. [124I]IBETA: A New Aβ Plaque Positron Emission Tomography Imaging Agent for Alzheimer's Disease. *Molecules* **2022**, *27* (14), 4552. DOI: 10.3390/molecules27144552.

(39) Limpengco, R. R.; Liang, C.; Sandhu, Y. K.; Mukherjee, J. [125I]INFT: Synthesis and Evaluation of a New Imaging Agent for Tau Protein in Post-Mortem Human Alzheimer's Disease Brain. *Molecules* **2023**, *28* (15), 5769. DOI: 10.3390/molecules28155769.

(40) Furumoto, S.; Okamura, N.; Furukawa, K.; Tashiro, M.; Ishikawa, Y.; Sugi, K.; Tomita, N.; Waragai, M.; Harada, R.; Tago, T.; Iwata, R.; Yanai, K.; Arai, H.; Kudo, Y. A 18 F-Labeled BF-227 Derivative as a Potential Radioligand for Imaging Dense Amyloid Plaques by Positron Emission Tomography. *Mol Imaging Biol* **2013**, *15*, 497–506. DOI: 10.1007/s11307-012-0608-5.

(41) Zhu, M.; Zhang, G.; Hu, Z.; Zhu, C.; Chen, Y.; James, T. D.; Ma, L.; Wang, Z. A BODIPY-Based Probe for Amyloid-β Imaging in Vivo. *Org. Chem. Front.* **2023**, *10* (8), 1903–1909. DOI: 10.1039/D2QO02032G.

(42) Crystal, A. S.; Giasson, B. I.; Crowe, A.; Kung, M. P.; Zhuang, Z. P.; Trojanowski, J. Q.; Lee, V. M. Y. A Comparison of Amyloid Fibrillogenesis Using the Novel Fluorescent Compound K114. *J. Neurochem.* **2003**, *86* (6), 1359–1368. DOI: 10.1046/j.1471-4159.2003.01949.x.

(43) Lim, S.; Paterson, B. M.; Fodero-Tavoletti, M. T.; O'Keefe, G. J.; Cappai, R.; Barnham, K. J.; Villemagne, V. L.; Donnelly, P. S. A Copper Radiopharmaceutical for Diagnostic Imaging of Alzheimer's Disease: A Bis(Thiosemicarbazonato)Copper(Ii) Complex That Binds to Amyloid-β Plaques. *Chem. Commun.* **2010**, *46* (30), 5437–5439. DOI: 10.1039/c0cc01175d.

(44) Watanabe, H.; Ono, M.; Kimura, H.; Kagawa, S.; Nishii, R.; Fuchigami, T.; Haratake, M.; Nakayama, M.; Saji, H. A Dual Fluorinated and Iodinated Radiotracer for PET and SPECT Imaging of β-Amyloid Plaques in the Brain. *Bioorg. Med. Chem. Lett.* **2011**, *21* (21), 6519–6522. DOI: 10.1016/j.bmcl.2011.08.063.

(45) Zhuang, Z.-M.; Zhou, Z.; Chen, X.; Xu, X.-R.; Wang, H.-X.; Pan, J. A Flexible Bivalent Approach to Comprehensively Improve the Performances of Stilbazolium Dyes as Amyloid-β Fluorescent Probes. *ACS Appl. Mater. Interfaces* **2023**, *15* (38), 44742–44751. DOI: 10.1021/acsami.3c09034.

(46) Di Nanni, A.; Saw, R. S.; Battisti, U. M.; Bowden, G. D.; Boeckermann, A.; Bjerregaard-Andersen, K.; Pichler, B. J.; Herfert, K.; Herth, M. M.; Maurer, A. A Fluorescent Probe as a Lead Compound for a Selective α-Synuclein PET Tracer: Development of a Library of 2-Styrylbenzothiazoles and Biological Evaluation of [18F]PFSB and [18F]MFSB. *ACS Omega* **2023**, *8* (34), 31450–31467. DOI: 10.1021/acsomega.3c04292.

(47) Yang, H.-L.; Fang, S.-Q.; Tang, Y.-W.; Wang, C.; Luo, H.; Qu, L.-L.; Zhao, J.-H.; Shi, C.-J.; Yin, F.-C.; Wang, X.-B.; Kong, L.-Y. A Hemicyanine Derivative for Near-Infrared Imaging of β-Amyloid Plaques in Alzheimer's Disease. *Eur. J. Med. Chem.* **2019**, *179*, 736–743. DOI: 10.1016/j.ejmech.2019.07.005.

(48) Rajasekhar, K.; Narayanaswamy, N.; Murugan, N. A.; Kuang, G.; Ågren, H.; Govindaraju, & T. A High Affinity Red Fluorescence and Colorimetric Probe for Amyloid β Aggregates OPEN. *Sci. Rep.* **2016**, *6*, 23668. DOI: 10.1038/srep23668.

(49) Zhang, W.; Arteaga, J.; Cashion, D. K.; Chen, G.; Gangadharmath, U.; Gomez, L. F.; Kasi, D.; Lam, C.; Liang, Q.; Liu, C.; Mocharla, V. P.; Mu, F.; Sinha, A.; Katrin Szardenings, A.; Wang, E.; Walsh, J. C.; Xia, C.; Yu, C.; Zhao, T.; Kolb, H. C. A Highly Selective and Specific PET Tracer for Imaging of Tau Pathologies. *J. Alzheimers Dis.* **2012**, *31* (3), 601–612. DOI: 10.3233/JAD-2012-120712.

(50) Mathis, C. A.; Bacskai, B. J.; Kajdasz, S. T.; McLellan, M. E.; Frosch, M. P.; Hyman, B. T.; Holt, D. P.; Wang, Y.; Huang, G. F.; Debnath, M. L.; Klunk, W. E. A Lipophilic Thioflavin-T Derivative for Positron Emission Tomography (PET) Imaging of Amyloid in Brain. *Bioorg. Med. Chem. Lett.* **2002**, *12* (3), 295–298. DOI: 10.1016/S0960-894X(01)00734-X.

(51) Tan, H.; Qiu, Y.; Sun, H.; Yan, J.; Zhang, L. A Lysosome-Targeting Dual-Functional Fluorescent Probe for Imaging Intracellular Viscosity and Beta-Amyloid. *Chem. Commun.* **2019**, *55* (18), 2688–2691. DOI: 10.1039/C9CC00113A.

(52) Rivera-Marrero, S. A New Naphthalene Derivative with Anti-Amyloidogenic Activity as Potential Therapeutic Agent for Alzheimer's Disease. *Bioorg. Med. Chem.* **2020**, *28* (20), 115700. DOI: 10.1016/j.bmc.2020.115700.

(53) Cheng, Y.; Ono, M.; Kimura, H.; Kagawa, S.; Nishii, R.; Saji, H. A Novel 18F-Labeled Pyridyl Benzofuran Derivative for Imaging of β-Amyloid Plaques in Alzheimer's Brains. *Bioorg. Med. Chem. Lett.* **2010**, *20* (20), 6141–6144. DOI: 10.1016/j.bmcl.2010.08.016.

(54) Sun, H.; Leng, H.; Liu, J.; Roy, G.; Yan, J.; Zhang, L. A Novel Fluorescent Protein Chromophore Analogue to Simultaneously Probe Lysosome Viscosity and β-Amyloid Fibrils. *Sens. Actuators B Chem.* **2020**, *305*, 127509. DOI: 10.1016/j.snb.2019.127509.

(55) Okamura, N.; Suemoto, T.; Shiomitsu, T.; Suzuki, M.; Shimadzu, H.; Akatsu, H.; Yamamoto, T.; Arai, H.; Sasaki, H.; Yanai, K.; Staufenbiel, M.; Kudo, Y.; Sawada, T. A Novel Imaging Probe for In Vivo Detection of Neuritic and Diffuse Amyloid Plaques in the Brain. *J. Mol. Neurosci.* **2004**, *24*, 247–255.

(56) Naganuma, F.; Murata, D.; Inoue, M.; Maehori, Y.; Harada, R.; Furumoto, S.; Kudo, Y.; Nakamura, T.; Okamura, N. A Novel Near-Infrared Fluorescence Probe THK-565 Enables In Vivo Detection of Amyloid Deposits in Alzheimer's Disease Mouse Model. *Mol. Imaging Biol.* **2023**, *25*, 1115–1124. DOI: 10.1007/s11307-023-01843-4.

(57) Shimadzu, H.; Suemoto, T.; Suzuki, M.; Shiomitsu, T.; Okamura, N.; Kudo, Y.; Sawada, T. A Novel Probe for Imaging Amyloid-β: Synthesis of F-18 Labelled BF-108, an Acridine Orange Analog. *J. Label. Compd. Radiopharm.* **2003**, *46* (8), 765–772. DOI: 10.1002/jlcr.716.

(58) Cheng, Y.; Zhu, B. Y.; Li, X.; Li, G. B.; Yang, S. Y.; Zhang, Z. R. A Pyrane Based Fluorescence Probe for Noninvasive Prediction of Cerebral β-Amyloid Fibrils. *Bioorg. Med. Chem. Lett.* **2015**, *25* (20), 4472–4476. DOI: 10.1016/j.bmcl.2015.08.081.

(59) Dae Park, Y.; Kim, J.-J.; Lee, S.; Park, C.-H.; Bai, H.-W.; Lee, S. S. A Pyridazine-Based Fluorescent Probe Targeting Aβ Plaques in Alzheimer's Disease. *J. Anal. Methods Chem.* **2018**, *2018*, 1651989. DOI: 10.1155/2018/1651989.
(60) Tu, Y.; Chai, K.; Wu, J.; Hu, Y.; Shi, S.; Yang, D.; Yao, T. A Rational Design to Improve Selective Imaging of Tau Aggregates by Constructing Side Substitution on N,N-Dimethylaniline/Quinoxaline D-π-A Fluorescent Probe. *Sens. Actuators B Chem.* **2023**, *380*, 133406. DOI: 10.1016/j.snb.2023.133406.
(61) Seo, Y.; Park, K. S.; Ha, T.; Kim, M. K.; Hwang, Y. J.; Lee, J.; Ryu, H.; Choo, H.; Chong, Y. A Smart Near-Infrared Fluorescence Probe for Selective Detection of Tau Fibrils in Alzheimer's Disease. *ACS Chem. Neurosci.* **2016**, *7* (11), 1474–1481. DOI: 10.1021/acschemneuro.6b00174.
(62) Lv, G.; Sun, A.; Wei, P.; Zhang, N.; Lan, H.; Yi, T. A Spiropyran-Based Fluorescent Probe for the Specific Detection of β-Amyloid Peptide Oligomers in Alzheimer's Disease. *Chem. Commun.* **2016**, *52* (57), 8865–8868. DOI: 10.1039/c6cc02741e.
(63) Li, Y.; Xu, D.; Ho, S.-L.; Li, H.-W.; Yang, R.; Wong, M. S. A Theranostic Agent for in Vivo Near-Infrared Imaging of b-Amyloid Species and Inhibition of b-Amyloid Aggregation. *Biomaterials* **2016**, *94*, 84–92. DOI: 10.1016/j.biomaterials.2016.03.047.
(64) Benzeid, H.; Mothes, E.; Essassi, E. M.; Faller, P.; Pratviel, G. A Thienoquinoxaline and a Styryl-Quinoxaline as New Fluorescent Probes for Amyloid-β Fibrils. *Comptes Rendus Chim.* **2012**, *15* (1), 79–85. DOI: 10.1016/j.crci.2011.10.009.
(65) Xu, M.-M.; Ren, W.-M.; Tang, X.-C.; Hu, Y.-H.; Zhang, H.-Y. Advances in Development of Fluorescent Probes for Detecting Amyloid-β Aggregates. *Acta Pharmacol Sin* **2016**, *37*, 719–730. DOI: 10.1038/aps.2015.155.
(66) Sathish, V.; Babu, E.; Ramdass, A.; Lu, Z. Z.; Velayudham, M.; Thanasekaran, P.; Lu, K. L.; Rajagopal, S. Alkoxy Bridged Binuclear Rhenium (I) Complexes as a Potential Sensor for β-Amyloid Aggregation. *Talanta* **2014**, *130*, 274–279. DOI: 10.1016/j.talanta.2014.06.070.
(67) Sun, L.; Sharma, A. K.; Han, B.-H.; Mirica, L. M. Amentoflavone: A Bifunctional Metal Chelator That Controls the Formation of Neurotoxic Soluble Aβ42 Oligomers. *ACS Chem. Neurosci.* **2020**, *11* (17), 2741–2752. DOI: 10.1021/acschemneuro.0c00376.
(68) Cao, K.; Farahi, M.; Dakanali, M.; Chang, W. M.; Sigurdson, C. J.; Theodorakis, E. A.; Yang, J. Aminonaphthalene 2-Cyanoacrylate (ANCA) Probes Fluorescently Discriminate between Amyloid-β and Prion Plaques in Brain. *J. Am. Chem. Soc.* **2012**, *134* (42), 17338–17341. DOI: 10.1021/ja3063698.
(69) Sun, L.; Cho, H.-J.; Sen, S.; Arango, A. S.; Huynh, T. T.; Huang, Y.; Bandara, N.; Rogers, B. E.; Tajkhorshid, E.; Mirica, L. M. Amphiphilic Distyrylbenzene Derivatives as Potential Therapeutic and Imaging Agents for Soluble and Insoluble Amyloid β Aggregates in Alzheimer's Disease. *J. Am. Chem. Soc.* **2021**, *143* (27), 10462–10476. DOI: 10.1021/jacs.1c05470.
(70) Yu, Z.; Guo, W.; Patel, S.; Cho, H.-J.; Sun, L.; Mirica, L. M. Amphiphilic Stilbene Derivatives Attenuate the Neurotoxicity of Soluble Aβ42 Oligomers by Controlling Their Interactions with Cell Membranes. *Chem. Sci.* **2022**, *13* (43), 12818–12830. DOI: 10.1039/D2SC02654F.
(71) Ashburn, T. T.; Han, H.; McGuinness, B. F.; Lansbury, P. T. Amyloid Probes Based on Congo Red Distinguish between Fibrils Comprising Different Peptides. *Chem. Biol.* **1996**, *3* (5), 351–358. DOI: 10.1016/S1074-5521(96)90118-0.
(72) Kim, H.; Parida, R.; Youn, K.; Shin, S. C.; Keum, G.; Lee, J. Y.; Bang, E.-K.; Yoo, S. Amyloid β Aggregate-Sensitive Red Fluorescent Dipolar Probes. *ACS Appl. Opt. Mater.* **2023**, *1* (9), 1513–1526. DOI: 10.1021/acsaom.3c00159.
(73) Fu, H.; Tu, P.; Zhao, L.; Dai, J.; Liu, B.; Cui, M. Amyloid-β Deposits Target Efficient Near-Infrared Fluorescent Probes: Synthesis, in Vitro Evaluation, and in Vivo Imaging. *Anal. Chem.* **2016**, *88* (3), 1944–1950. DOI: 10.1021/acs.analchem.5b04441.
(74) Kepe, V.; Moghbel, M. C.; Långströmd, B.; Zaidi, H.; Vinters, H. V.; Huang, S. C.; Satyamurthy, N.; Doudet, D.; Mishani, E.; Cohen, R. M.; Høilund-Carlsen, P. F.; Alavi, A.; Barrio, J. R. Amyloid-β Positron

Emission Tomography Imaging Probes: A Critical Review. *J. Alzheimers Dis.* **2013**, *36* (4), 613–631. DOI: 10.3233/JAD-130485.
(75) Eckroat, T. J.; Mayhoub, A. S.; Garneau-Tsodikova, S. Amyloid-β Probes: Review of Structure-Activity and Brain-Kinetics Relationships. *Beilstein J Org Chem* **2013**, *9*, 1012–1044. DOI: 10.3762/bjoc.9.116.
(76) Miao, J.; Miao, M.; Jiang, Y.; Zhao, M.; Li, Q.; Zhang, Y.; An, Y.; Pu, K.; Miao, Q. An Activatable NIR-II Fluorescent Reporter for In Vivo Imaging of Amyloid-β Plaques. *Angew. Chem. Int. Ed.* **2023**, *62* (7), e202216351. DOI: 10.1002/anie.202216351.
(77) Zhao, M.; Zhang, G.; Huang, S.; Zhang, J.; Zhu, Y.; Zhu, X.; Zhang, R.; Li, F. An Activatable Small-Molecule Fluorogenic Probe for Detection and Quantification of Beta-Amyloid Aggregates. *Spectrochim. Acta. A. Mol. Biomol. Spectrosc.* **2023**, *303*, 123145. DOI: 10.1016/j.saa.2023.123145.
(78) Ji, Y.-M.; Hou, M.; Zhou, W.; Ning, Z.-W.; Zhang, Y.; Xing, G.-W. An AIE-Active NIR Fluorescent Probe with Good Water Solubility for the Detection of Aβ1–42 Aggregates in Alzheimer's Disease. *Molecules* **2023**, *28* (13), 5110. DOI: 10.3390/molecules28135110.
(79) Marzano, N. R.; Wray, K. M.; Johnston, C. L.; Paudel, B. P.; Hong, Y.; van Oijen, A.; Ecroyd, H. An α-Cyanostilbene Derivative for the Enhanced Detection and Imaging of Amyloid Fibril Aggregates. *ACS Chem. Neurosci.* **2020**, *11* (24), 4191–4202. DOI: 10.1021/acschemneuro.0c00478.
(80) Chang, W. M.; Dakanali, M.; Capule, C. C.; Sigurdson, C. J.; Yang, J.; Theodorakis, E. A. ANCA: A Family of Fluorescent Probes That Bind and Stain Amyloid Plaques in Human Tissue. *ACS Chem. Neurosci.* **2011**, *2* (5), 249–255. DOI: 10.1021/cn200018v.
(81) Deeg, A. A.; Reiner, A. M.; Schmidt, F.; Schueder, F.; Ryazanov, S.; Ruf, V. C.; Giller, K.; Becker, S.; Leonov, A.; Griesinger, C.; Giese, A.; Zinth, W. Anle138b and Related Compounds Are Aggregation Specific Fluorescence Markers and Reveal High Affinity Binding to α-Synuclein Aggregates. *Biochim. Biophys. Acta - Gen. Subj.* **2015**, *1850* (9), 1884–1890. DOI: 10.1016/j.bbagen.2015.05.021.
(82) Chen, X.; Li, Y.; Kang, J.; Ye, T.; Yang, Z.; Liu, Z.; Liu, Q.; Zhao, Y.; Liu, G.; Pan, J. Application of a Novel Coumarin-Derivative near-Infrared Fluorescence Probe to Amyloid-β Imaging and Inhibition in Alzheimer's Disease. *J. Lumin.* **2023**, *256*, 119661. DOI: 10.1016/j.jlumin.2022.119661.
(83) Lee, B. C.; Kim, J. S.; Kim, B. S.; Son, J. Y.; Hong, S. K.; Park, H. S.; Moon, B. S.; Jung, J. H.; Jeong, J. M.; Kim, S. E. Aromatic Radiofluorination and Biological Evaluation of 2-Aryl-6-[18F]Fluorobenzothiazoles as a Potential Positron Emission Tomography Imaging Probe for β-Amyloid Plaques. *Bioorg. Med. Chem.* **2011**, *19* (9), 2980–2990. DOI: 10.1016/j.bmc.2011.03.029.
(84) Rana, M.; Pareek, A.; Bhardwaj, S.; Arya, G.; Nimesh, S.; Arya, H.; Bhatt, T. K.; Yaragorla, S.; Sharma, A. K. Aryldiazoquinoline Based Multifunctional Small Molecules for Modulating Aβ42 Aggregation and Cholinesterase Activity Related to Alzheimer's Disease. *RSC Adv.* **2020**, *10* (48), 28827–28837. DOI: 10.1039/d0ra05172a.
(85) Ono, M.; Maya, Y.; Haratake, M.; Ito, K.; Mori, H.; Nakayama, M. Aurones Serve as Probes of β-Amyloid Plaques in Alzheimer's Disease. *Biochem. Biophys. Res. Commun.* **2007**, *361* (1), 116–121. DOI: 10.1016/j.bbrc.2007.06.162.
(86) Johnson, A. E.; Jeppsson, F.; Sandell, J.; Wensbo, D.; Neelissen, J. A. M.; Juréus, A.; Ström, P.; Norman, H.; Farde, L.; Svensson, S. P. S. AZD2184: A Radioligand for Sensitive Detection of β-Amyloid Deposits. *J. Neurochem.* **2009**, *108* (5), 1177–1186. DOI: 10.1111/j.1471-4159.2008.05861.x.
(87) Rajasekhar, K.; Narayanaswamy, N.; Murugan, N. A.; Viccaro, K.; Lee, H. G.; Shah, K.; Govindaraju, T. Aβ Plaque-Selective NIR Fluorescence Probe to Differentiate Alzheimer's Disease from Tauopathies. *Biosens. Bioelectron.* **2017**, *98*, 54–61. DOI: 10.1016/j.bios.2017.06.030.
(88) Pravin, N.; Kumar, R.; Tripathi, S.; Kumar, P.; Mohite, G. M.; Navalkar, A.; Panigrahi, R.; Singh, N.; Gadhe, L. G.; Manchanda, S.; Shimozawa, M.; Nilsson, P.; Johansson, J.; Kumar, A.; Maji, S. K.; Shanmugam, M. Benzimidazole-Based Fluorophores for the Detection of Amyloid Fibrils with Higher Sensitivity than Thioflavin-T. *J. Neurochem.* **2021**, *156* (6), 1003–1019. DOI: 10.1111/jnc.15138.

(89) Ono, M.; Kung, M.-P.; Hou, C.; Kung, H. F. Benzofuran Derivatives as A-Aggregate-Specific Imaging Agents for Alzheimer's Disease. *Nucl. Med. Biol.* **2002**, *29*, 633–642. DOI: 10.1016/S0969-8051(02)00326-8.

(90) Gan, C.; Zhou, L.; Zhao, Z.; Wang, H. Benzothiazole Schiff-Bases as Potential Imaging Agents for β-Amyloid Plaques in Alzheimer's Disease. *Med. Chem. Res.* **2013**, *22* (9), 4069–4074. DOI: 10.1007/s00044-012-0416-0.

(91) Ma, L.; Yang, S.; Ma, Y.; Chen, Y.; Wang, Z.; James, T. D.; Wang, X.; Wang, Z. Benzothiazolium Derivative-Capped Silica Nanocomposites for β-Amyloid Imaging In Vivo. *Anal. Chem.* **2021**, *93* (37), 12617–12627. DOI: 10.1021/acs.analchem.1c02289.

(92) Agdeppa, E. D.; Kepe, V.; Liu, J.; Flores-Torres, S.; Satyamurthy, N.; Petric, A.; Cole, G. M.; Small, G. W.; Huang, S. C.; Barrio, J. R. Binding Characteristics of Radiofluorinated 6-Dialkylamino-2-Naphthylethylidene Derivatives as Positron Emission Tomography Imaging Probes for Beta-Amyloid Plaques in Alzheimer's Disease. *J. Neurosci. Off. J. Soc. Neurosci.* **2001**, *21* (24), RC189–RC189. DOI: 10.1523/jneurosci.21-24-j0004.2001.

(93) Groenning, M. Binding Mode of Thioflavin T and Other Molecular Probes in the Context of Amyloid Fibrils-Current Status. *J. Chem. Biol.* **2010**, *3* (1), 1–18. DOI: 10.1007/s12154-009-0027-5.

(94) Bagchi, D. P.; Yu, L.; Perlmutter, J. S.; Xu, J.; Mach, R. H.; Tu, Z.; Kotzbauer, P. T. Binding of the Radioligand SIL23 to α-Synuclein Fibrils in Parkinson Disease Brain Tissue Establishes Feasibility and Screening Approaches for Developing a Parkinson Disease Imaging Agent. *PLoS ONE* **2013**, *8* (2), e55031. DOI: 10.1371/journal.pone.0055031.

(95) Rovnyagina, N. R.; Sluchanko, N. N.; Tikhonova, T. N.; Fadeev, V. V.; Litskevich, A. Y.; Maskevich, A. A.; Shirshin, E. A. Binding of Thioflavin T by Albumins: An Underestimated Role of Protein Oligomeric Heterogeneity. *Int. J. Biol. Macromol.* **2018**, *108*, 284–290. DOI: 10.1016/j.ijbiomac.2017.12.002.

(96) Kung, M. P.; Hou, C.; Zhuang, Z. P.; Skovronsky, D.; Kung, H. F. Binding of Two Potential Imaging Agents Targeting Amyloid Plaques in Postmortem Brain Tissues of Patients with Alzheimer's Disease. *Brain Res.* **2004**, *1025* (1–2), 98–105. DOI: 10.1016/j.brainres.2004.08.004.

(97) Yang, Y.; Zhu, L.; Chen, X.; Zhang, H. Binding Research on Flavones as Ligands of β-Amyloid Aggregates by Fluorescence and Their 3D-QSAR, Docking Studies. *J. Mol. Graph. Model.* **2010**, *29* (4), 538–545. DOI: 10.1016/j.jmgm.2010.10.006.

(98) Hanczyc, P. Binuclear Ruthenium(II) Complexes for Amyloid Fibrils Recognition. *Chem. Phys.* **2014**, *445*, 1–4. DOI: 10.1016/j.chemphys.2014.10.015.

(99) Zhuang, Z. P.; Kung, M. P.; Hou, C.; Ploessl, K.; Kung, H. F. Biphenyls Labeled with Technetium 99m for Imaging β-Amyloid Plaques in the Brain. *Nucl. Med. Biol.* **2005**, *32* (2), 171–184. DOI: 10.1016/j.nucmedbio.2004.10.002.

(100) Boländer, A.; Kieser, D.; Voss, C.; Bauer, S.; Schön, C.; Burgold, S.; Bittner, T.; Hölzer, J.; Heyny-Von Haußen, R.; Mall, G.; Goetschy, V.; Czech, C.; Knust, H.; Berger, R.; Herms, J.; Hilger, I.; Schmidt, B. Bis(Arylvinyl)Pyrazines, -Pyrimidines, and -Pyridazines As Imaging Agents for Tau Fibrils and β-Amyloid Plaques in Alzheimers Disease Models. *J. Med. Chem.* **2012**, *55* (21), 9170–9180. DOI: 10.1021/jm300653b.

(101) Banka, V. K.; Sehlin, D.; Hultqvist, G.; Sigurdsson, E.; Boutajangout, A.; Wisniewski, T.; Syvanen, S.; Ding, Y.-S. Bispecific Antibody Radioligands for in Vivo PET Imaging of Both Amyloid-β and Tau. *J. Nucl. Med.* **2022**, *63* (supplement 2), 2853–2853.

(102) Liu, K.; Guo, T. L.; Chojnacki, J.; Lee, H. G.; Wang, X.; Siedlak, S. L.; Rao, W.; Zhu, X.; Zhang, S. Bivalent Ligand Containing Curcumin and Cholesterol as a Fluorescence Probe for Aβ Plaques in Alzheimer's Disease. *ACS Chem. Neurosci.* **2012**, *3* (2), 141–146. DOI: 10.1021/cn200122j.

(103) Ono, M.; Watanabe, H.; Kimura, H.; Saji, H. BODIPY-Based Molecular Probe for Imaging of Cerebral β-Amyloid Plaques. *ACS Chem Neurosci* **2012**, *3*, 319–324. DOI: 10.1021/cn3000058.

(104) Cai, L.; Qu, B.; Hurtle, B. T.; Dadiboyena, S.; Diaz-Arrastia, R.; Pike, V. W. Candidate PET Radioligand Development for Neurofibrillary Tangles: Two Distinct Radioligand Binding Sites Identified in

Postmortem Alzheimer's Disease Brain Graphical Abstract HHS Public Access. *ACS Chem Neurosci* **2016**, *7* (7), 897–911. DOI: 10.1021/acschemneuro.6b00051.
(105) Ono, K.; Noguchi, M.; Matsumoto, Y.; Yanase, D.; Iwasa, K.; Naiki, H.; Yamada, M. Cerebrospinal Fluid of Alzheimer Patients Promotes β-Amyloid Fibril Formation in Vitro. *Neurobiol Dis* **2005**, *20* (2), 233–240. DOI: 10.1016/j.nbd.2005.03.005.
(106) Kaide, S.; Watanabe, H.; Iikuni, S.; Hasegawa, M.; Itoh, K.; Ono, M. Chalcone Analogue as New Candidate for Selective Detection of α-Synuclein Pathology. *ACS Chem. Neurosci.* **2021**, *13* (1), 16–26. DOI: 10.1021/acschemneuro.1c00441.
(107) Hsieh, C. J.; Xu, K.; Lee, I.; Graham, T. J. A.; Tu, Z.; Dhavale, D.; Kotzbauer, P.; Mach, R. H. Chalcones and Five-Membered Heterocyclic Isosteres Bind to Alpha Synuclein Fibrils in Vitro. *ACS Omega* **2018**, *3* (4), 4486–4493. DOI: 10.1021/acsomega.7b01897.
(108) Ye, L.; Morgenstern, J. L.; Lamb, J. R.; Lockhart, A. Characterisation of the Binding of Amyloid Imaging Tracers to Rodent Aβ Fibrils and Rodent-Human Aβ Co-Polymers. *Biochem. Biophys. Res. Commun.* **2006**, *347* (3), 669–677. DOI: 10.1016/j.bbrc.2006.06.126.
(109) Sundaram, G. S. M.; Garai, K.; Rath, N. P.; Yan, P.; Cirrito, J. R.; Cairns, N. J.; Lee, J.-M.; Sharma, V. Characterization of a Brain Permeant Fluorescent Molecule and Visualization of Aβ Parenchymal Plaques, Using Real-Time Multiphoton Imaging in Transgenic Mice. *Org. Lett.* **2014**, *16* (14), 3640–3643. DOI: 10.1021/ol501264q.
(110) Juréus, A.; Swahn, B. M.; Sandell, J.; Jeppsson, F.; Johnson, A. E.; Johnström, P.; Neelissen, J. A. M.; Sunnemark, D.; Farde, L.; Svensson, S. P. S. Characterization of AZD4694, a Novel Fluorinated Aβ Plaque Neuroimaging PET Radioligand. *J. Neurochem.* **2010**, *114* (3), 784–794. DOI: 10.1111/j.1471-4159.2010.06812.x.
(111) Kung, M. P.; Hou, C.; Zhuang, Z. P.; Cross, A. J.; Maier, D. L.; Kung, H. F. Characterization of IMPY as a Potential Imaging Agent for β-Amyloid Plaques in Double Transgenic PSAPP Mice. *Eur. J. Nucl. Med. Mol. Imaging* **2004**, *31* (8), 1136–1145. DOI: 10.1007/s00259-004-1487-z.
(112) Kung, M. P.; Zhuang, Z. P.; Hou, C.; Jin, L. W.; Kung, H. F. Characterization of Radioiodinated Ligand Binding to Amyloid β Plaques. *J. Mol. Neurosci.* **2003**, *20* (3), 249–253. DOI: 10.1385/JMN:20:3:249.
(113) Klunk, W. E.; Debnath, M. L.; Pettegrew, J. W. Chrysamine-G Binding to Alzheimer and Control Brain: Autopsy Study of a New Amyloid Probe. *Neurobiol. Aging* **1995**, *16* (4), 541–548. DOI: 10.1016/0197-4580(95)00058-M.
(114) Lenhart, J. A.; Ling, X.; Gandhi, R.; Guo, T. L.; Gerk, P. M.; Brunzell, D. H.; Zhang, S. "Clicked" Bivalent Ligands Containing Curcumin and Cholesterol as Multifunctional Aβ Oligomerization Inhibitors: Design, Synthesis, and Biological Characterization. *J. Med. Chem.* **2010**, *53* (16), 6198–6209. DOI: 10.1021/jm100601q.
(115) Wang, Q.; Zhong, J.; Li, K.; Wu, J.; Wang, X.; Jiang, S.; Dai, J.; Cheng, Y. Compact Luminol Chemiluminophores for In Vivo Detection and Imaging of β-Sheet Protein Aggregates. *Anal. Chem.* **2023**, *95* (2), 1065–1073. DOI: 10.1021/acs.analchem.2c03776.
(116) Lemoine, L.; Gillberg, P.-G.; Svedberg, M.; Stepanov, V.; Jia, Z.; Huang, J.; Nag, S.; Tian, H.; Ghetti, B.; Okamura, N.; Higuchi, M.; Halldin, C.; Nordberg, A. Comparative Binding Properties of the Tau PET Tracers THK5117, THK5351, PBB3, and T807 in Postmortem Alzheimer Brains. *Alzheimer's Res. Ther.* **2017**, *9*, 96. DOI: 10.1186/s13195-017-0325-z.
(117) Declercq, L.; Celen, S.; Lecina, J.; Ahamed, M.; Tousseyn, T.; Moechars, D.; Alcazar, J.; Ariza, M.; Fierens, K.; Bottelbergs, A.; Mariën, J.; Vandenberghe, R.; Andres, I. J.; Van Laere, K.; Verbruggen, A.; Bormans, G. Comparison of New Tau PET-Tracer Candidates with [18F]T808 and [18F]T807. *Mol. Imaging* **2016**, *15*, 1–15. DOI: 10.1177/1536012115624920.
(118) Harada, R.; Okamura, N.; Furumoto, S.; Tago, T.; Maruyama, M.; Higuchi, M.; Yoshikawa, T.; Arai, H.; Iwata, R.; Kudo, Y.; Yanai, K. Comparison of the Binding Characteristics of [18F]THK-523 and Other Amyloid Imaging Tracers to Alzheimer's Disease Pathology. *Eur. J. Nucl. Med. Mol. Imaging* **2013**, *40* (1), 125–132. DOI: 10.1007/s00259-012-2261-2.

(119) Yakupova, E. I.; Bobyleva, L. G.; Vikhlyantsev, I. M.; Bobylev, A. G. Congo Red and Amyloids: History and Relationship. *Biosci. Rep.* **2019**, *39* (1), BSR20181415. DOI: 10.1042/BSR20181415.
(120) Kaide, S.; Ono, M.; Watanabe, H.; Shimizu, Y.; Nakamoto, Y.; Togashi, K.; Yamaguchi, A.; Hanaoka, H.; Saji, H. Conversion of Iodine to Fluorine-18 Based on Iodinated Chalcone and Evaluation for β-Amyloid PET Imaging. *Bioorg. Med. Chem.* **2018**, *26* (12), 3352–3358. DOI: 10.1016/j.bmc.2018.05.001.
(121) Ryu, E. K.; Choe, Y. S.; Lee, K. H.; Choi, Y.; Kim, B. T. Curcumin and Dehydrozingerone Derivatives: Synthesis, Radiolabeling, and Evaluation for β-Amyloid Plaque Imaging. *J. Med. Chem.* **2006**, *49* (20), 6111–6119. DOI: 10.1021/jm0607193.
(122) Narlawar, R.; Pickhardt, M.; Leuchtenberger, S.; Baumann, K.; Krause, S.; Dyrks, T.; Weggen, S.; Mandelkow, E.; Schmidt, B. Curcumin-Derived Pyrazoles and Isoxazoles: Swiss Army Knives or Blunt Tools for Alzheimer's Disease? *ChemMedChem* **2008**, *3* (1), 165–172. DOI: 10.1002/cmdc.200700218.
(123) Zeng, Q.; Cui, M. Current Progress in the Development of Probes for Targeting α-Synuclein Aggregates. *ACS Chem. Neurosci.* **2022**, *13* (5), 552–571. DOI: 10.1021/acschemneuro.1c00877.
(124) Kotzbauer, P. T.; Tu, Z.; Mach, R. H. Current Status of the Development of PET Radiotracers for Imaging Alpha Synuclein Aggregates in Lewy Bodies and Lewy Neurites. *Clin. Transl. Imaging* **2017**, *5*, 3–14. DOI: 10.1007/s40336-016-0217-4.
(125) Zeng, Q.; Chen, Y.; Yan, Y.; Wan, R.; Li, Y.; Fu, H.; Liu, Y.; Liu, S.; Yan, X.-X.; Cui, M. D-π-A-Based Trisubstituted Alkenes as Environmentally Sensitive Fluorescent Probes to Detect Lewy Pathologies. *Anal. Chem.* **2022**, *94* (44), 15261–15269. DOI: 10.1021/acs.analchem.2c02532.
(126) Cornejo, A.; Caballero, J.; Simirgiotis, M.; Torres, V.; Sánchez, L.; Díaz, N.; Guimaraes, M.; Hernández, M.; Areche, C.; Alfaro, S.; Caballero, L.; Melo, F. Dammarane Triterpenes Targeting α-Synuclein: Biological Activity and Evaluation of Binding Sites by Molecular Docking. *J. Enzyme Inhib. Med. Chem.* **2021**, *36* (1), 154–162. DOI: 10.1080/14756366.2020.1851216.
(127) Ye, L.; Morgenstern, J. L.; Gee, A. D.; Hong, G.; Brown, J.; Lockhart, A. Delineation of Positron Emission Tomography Imaging Agent Binding Sites on β-Amyloid Peptide Fibrils. *J. Biol. Chem.* **2005**, *280* (25), 23599–23604. DOI: 10.1074/jbc.M501285200.
(128) Mallesh, R.; Khan, J.; Pradhan, K.; Roy, R.; Jana, N. R.; Jaisankar, P.; Ghosh, S. Design and Development of Benzothiazole-Based Fluorescent Probes for Selective Detection of Aβ Aggregates in Alzheimer's Disease. *ACS Chem. Neurosci.* **2022**, *13* (16), 2503–2516. DOI: 10.1021/acschemneuro.2c00361.
(129) Zhang, X.; Tian, Y.; Li, Z.; Tian, X.; Sun, H.; Liu, H.; Moore, A.; Ran, C. Design and Synthesis of Curcumin Analogues for in Vivo Fluorescence Imaging and Inhibiting Copper-Induced Cross-Linking of Amyloid Beta Species in Alzheimer's Disease. *J. Am. Chem. Soc.* **2013**, *135* (44), 16397–16409. DOI: 10.1021/ja405239v.
(130) Dao, P.; Ye, F.; Du, Z. Y.; Chen, Q.; Zhang, K.; Dong, C. Z.; Meunier, B.; Chen, H. Design and Synthesis of New Theranostic Agents for Near-Infrared Imaging of β-Amyloid Plaques and Inhibition of β-Amyloid Aggregation in Alzheimer's Disease. *Dyes Pigments* **2017**, *147*, 130–140. DOI: 10.1016/j.dyepig.2017.07.071.
(131) Ji, L.; Zhou, X.; Liu, R.; Wang, L.; Hu, Y.; Gu, J.; Li, Z.; Li, C.; Huang, T.; Yu, Y. Design of a Selective and Water-Soluble Fluorescent Probe Targeting Tau Fibrils for Intracellular and in Vivo Imaging. *Sens. Actuators B Chem.* **2023**, *380*, 133415. DOI: 10.1016/j.snb.2023.133415.
(132) Rejc, L.; Šmid, L.; Kepe, V.; Podlipnik, Č.; Golobič, A.; Bresjanac, M.; Barrio, J. R.; Petrič, A.; Košmrlj, J. Design, Syntheses, and in Vitro Evaluation of New Fluorine-18 Radiolabeled Tau-Labeling Molecular Probes. *J. Med. Chem.* **2017**, *60* (21), 8741–8757. DOI: 10.1021/acs.jmedchem.7b00764.
(133) Gu, J.; Anumala, U. R.; Heyny-von Haußen, R.; Hölzer, J.; Goetschy-Meyer, V.; Mall, G.; Hilger, I.; Czech, C.; Schmidt, B. Design, Synthesis and Biological Evaluation of Trimethine Cyanine Dyes as Fluorescent Probes for the Detection of Tau Fibrils in Alzheimer's Disease Brain and Olfactory Epithelium. *ChemMedChem* **2013**, *8* (6), 891–897. DOI: 10.1002/cmdc.201300090.
(134) Chen, Y. F.; Bian, J.; Zhang, P.; Bu, L. L.; Shen, Y.; Yu, W. B.; Lu, X. H.; Lin, X.; Ye, D. Y.; Wang, J.; Chu, Y. Design, Synthesis and Identification of N, N-Dibenzylcinnamamide (DBC) Derivatives as Novel Ligands

for α-Synuclein Fibrils by SPR Evaluation System. *Bioorg. Med. Chem.* **2020**, *28* (7), 115358. DOI: 10.1016/j.bmc.2020.115358.
(135) Chu, W.; Zhou, D.; Gaba, V.; Liu, J.; Li, S.; Peng, X.; Xu, J.; Dhavale, D.; Bagchi, D. P.; D'Avignon, A.; Shakerdge, N. B.; Bacskai, B. J.; Tu, Z.; Kotzbauer, P. T.; Mach, R. H. Design, Synthesis, and Characterization of 3-(Benzylidene)Indolin-2-One Derivatives as Ligands for α-Synuclein Fibrils. *J. Med. Chem.* **2015**, *58* (15), 6002–6017. DOI: 10.1021/acs.jmedchem.5b00571.
(136) Yue, X.; Dhavale, D. D.; Li, J.; Luo, Z.; Liu, J.; Yang, H.; Mach, R. H.; Kotzbauer, P. T.; Tu, Z. Design, Synthesis, and in Vitro Evaluation of Quinolinyl Analogues for α-Synuclein Aggregation. *Bioorg. Med. Chem. Lett.* **2018**, *28* (6), 1011–1019. DOI: 10.1016/j.bmcl.2018.02.031.
(137) Chandra, R.; Kung, M. P.; Kung, H. F. Design, Synthesis, and Structure-Activity Relationship of Novel Thiophene Derivatives for β-Amyloid Plaque Imaging. *Bioorg. Med. Chem. Lett.* **2006**, *16* (5), 1350–1352. DOI: 10.1016/j.bmcl.2005.11.055.
(138) Chongzhao, R.; Xiaoyin, X.; Raymond, S. B.; Ferrara, B. J.; Neal, K.; Bacskai, B. J.; Medarova, Z.; Moore, A. Design, Synthesis, and Testing of Difluoroboron-Derivatized Curcumins as near-Infrared Probes for in Vivo Detection of Amyloid-β Deposits. *J. Am. Chem. Soc.* **2009**, *131* (42), 15257–15261. DOI: 10.1021/ja9047043.
(139) Sozmen, F.; Kolemen, S.; Kumada, H.-O.; Ono, M.; Saji, H.; Akkaya, E. U. Designing BODIPY-Based Probes for Fluorescence Imaging of β-Amyloid Plaques. *RSC Adv.* **2014**, *4* (92), 51032–51037. DOI: 10.1039/C4RA07754G.
(140) Fisher, E.; Zhao, Y.; Richardson, R.; Janik, M.; Buell, A. K.; Aigbirhio, F. I.; Tóth, G. Detection and Characterization of Small Molecule Interactions with Fibrillar Protein Aggregates Using Microscale Thermophoresis. *ACS Chem. Neurosci.* **2017**, *8* (9), 2088–2095. DOI: 10.1021/acschemneuro.7b00228.
(141) Zhang, J.; Sandberg, A.; Konsmo, A.; Wu, X.; Nyström, S.; Nilsson, K. P. R.; Konradsson, P.; LeVine, H.; Lindgren, M.; Hammarström, P. Detection and Imaging of Aβ1-42 and Tau Fibrils by Redesigned Fluorescent X-34 Analogues. *Chem. - Eur. J.* **2018**, *24* (28), 7210–7216. DOI: 10.1002/chem.201800501.
(142) Wai, C.; Leung, T.; Guo, F.; Hong, Y.; Zhao, E.; Tsz, R.; Kwok, K.; Lik, N.; Leung, C.; Chen, S.; Vaikath, N. N.; Mukhtar El-Agnaf, O.; Tang, Y.; Gai, W.-P.; Tang, B. Z. Detection of Oligomers and Fibrils of A-Synuclein by AIEgen with Strong Fluorescence. *Chem. Commun.* **2015**, *51*, 1869. DOI: 10.1039/c4cc07911f.
(143) Kaur, H.; Felix, M. R.; Liang, C.; Mukherjee, J. Development and Evaluation of [18F]Flotaza for Aβ Plaque Imaging in Postmortem Human Alzheimer's Disease Brain. *Bioorg. Med. Chem. Lett.* **2021**, *46*, 128164. DOI: 10.1016/j.bmcl.2021.128164.
(144) Reddy, T. T.; Iguban, M. H.; Melkonyan, L. L.; Shergill, J.; Liang, C.; Mukherjee, J. Development of [124/125I]IAZA as a New Proteinopathy Imaging Agent for Alzheimer's Disease. *Molecules* **2023**, *28* (2), 865. DOI: 10.3390/molecules28020865.
(145) Wang, Y.; Klunk, W. E.; Debnath, M. L.; Huang, G. F.; Holt, D. P.; Shao, L.; Mathis, C. A. Development of a PET/SPECT Agent for Amyloid Imaging in Alzheimer's Disease. *J. Mol. Neurosci.* **2004**, *24* (1), 55–62. DOI: 10.1385/jmn:24:1:055.
(146) Fuchigami, T.; Ogawa, A.; Yamashita, Y.; Haratake, M.; Watanabe, H.; Ono, M.; Kawasaki, M.; Yoshida, S.; Nakayama, M. Development of Alkoxy Styrylchromone Derivatives for Imaging of Cerebral Amyloid-β Plaques with SPECT. *Bioorg. Med. Chem. Lett.* **2015**, *25*, 3363–3367. DOI: 10.1016/j.bmcl.2015.05.048.
(147) Ono, M.; Ishikawa, M.; Kimura, H.; Hayashi, S.; Matsumura, K.; Watanabe, H.; Shimizu, Y.; Cheng, Y.; Cui, M.; Kawashima, H.; Saji, H. Development of Dual Functional SPECT/Fluorescent Probes for Imaging Cerebral β-Amyloid Plaques. *Bioorg. Med. Chem. Lett.* **2010**, *20* (13), 3885–3888. DOI: 10.1016/j.bmcl.2010.05.027.
(148) Ono, M.; Cheng, Y.; Kimura, H.; Watanabe, H.; Matsumura, K.; Yoshimura, M.; Iikuni, S.; Okamoto, Y.; Ihara, M.; Takahashi, R.; Saji, H. Development of Novel 123I-Labeled Pyridyl Benzofuran Derivatives for SPECT Imaging of β-Amyloid Plaques in Alzheimer's Disease. *PLoS ONE* **2013**, *8* (9), e74104. DOI: 10.1371/journal.pone.0074104.

(149) Guo, Y.; Leng, H.; Chen, Q.; Su, J.; Shi, W.; Xia, C.; Zhang, L.; Yan, J. Development of Novel Near-Infrared GFP Chromophore-Based Fluorescent Probes for Imaging of Amyloid-β Plaque and Viscosity. *Sens. Actuators B Chem.* **2022**, *372*, 132648. DOI: 10.1016/j.snb.2022.132648.
(150) Ono, M.; Haratake, M.; Saji, H.; Nakayama, M. Development of Novel β-Amyloid Probes Based on 3,5-Diphenyl-1,2,4-Oxadiazole. *Bioorg. Med. Chem.* **2008**, *16* (14), 6867–6872. DOI: 10.1016/j.bmc.2008.05.054.
(151) Dao, P.; Ye, F.; Liu, Y.; Du, Z. Y.; Zhang, K.; Dong, C. Z.; Meunier, B.; Chen, H. Development of Phenothiazine-Based Theranostic Compounds That Act Both as Inhibitors of β-Amyloid Aggregation and as Imaging Probes for Amyloid Plaques in Alzheimer's Disease. *ACS Chem. Neurosci.* **2017**, *8* (4), 798–806. DOI: 10.1021/acschemneuro.6b00380.
(152) Klunk, W. E.; Debnath, M. L.; Pettegrew, J. W. Development of Small Molecule Probes for the Beta-Amyloid Protein of Alzheimer's Disease. *Neurobiol. Aging* **1994**, *15* (6), 691–698. DOI: 10.1016/0197-4580(94)90050-7.
(153) Lv, G.; Cui, B.; Lan, H.; Wen, Y.; Sun, A.; Yi, T. Diarylethene Based Fluorescent Switchable Probes for the Detection of Amyloid-β Pathology in Alzheimer's Disease. *Chem. Commun.* **2015**, *51* (1), 125–128. DOI: 10.1039/c4cc07656g.
(154) Wu, C.; Wei, J.; Gao, K.; Wang, Y. Dibenzothiazoles as Novel Amyloid-Imaging Agents. *Bioorg. Med. Chem.* **2007**, *15* (7), 2789–2796. DOI: 10.1016/j.bmc.2006.11.022.
(155) Petrič, A.; Johnson, S. A.; Pham, H. V.; Li, Y.; Cěh, S.; Golobič, A.; Agdeppa, E. D.; Timbol, G.; Liu, J.; Keum, G.; Satyamurthy, N.; Kepe, V.; Houk, K. N.; Barrio, J. R. Dicyanovinylnaphthalenes for Neuroimaging of Amyloids and Relationships of Electronic Structures and Geometries to Binding Affinities. *Proc. Natl. Acad. Sci. U. S. A.* **2012**, *109* (41), 16492–16497. DOI: 10.1073/pnas.1214134109.
(156) Honson, N. S.; Johnson, R. L.; Huang, W.; Inglese, J.; Austin, C. P.; Kuret, J. Differentiating Alzheimer Disease-Associated Aggregates with Small Molecules. *Neurobiol. Dis.* **2007**, *28* (3), 251–260. DOI: 10.1016/j.nbd.2007.07.018.
(157) Yang, J.; Zhu, B.; Yin, W.; Han, Z.; Zheng, C.; Wang, P.; Ran, C. Differentiating Aβ40 and Aβ42 in Amyloid Plaques with a Small Molecule Fluorescence Probe. *Chem. Sci.* **2020**, *11* (20), 5238–5245. DOI: 10.1039/D0SC02060E.
(158) Lee, C. W.; Kung, M. P.; Hou, C.; Kung, H. F. Dimethylamino-Fluorenes: Ligands for Detecting β-Amyloid Plaques in the Brain. *Nucl. Med. Biol.* **2003**, *30* (6), 573–580. DOI: 10.1016/S0969-8051(03)00050-7.
(159) Liu, T.; Li, Y.; Wang, Y.; Yan, X.-X.; Dai, J.; Cui, M. Discovery and Evaluation of Aza-Fused Tricyclic Derivatives for Detection of Tau Pathology in Alzheimer's Disease. *Eur. J. Med. Chem.* **2023**, *246*, 114991. DOI: 10.1016/j.ejmech.2022.114991.
(160) Kroth, H.; Oden, F.; Molette, J.; Schieferstein, H.; Capotosti, F.; Mueller, A.; Berndt, M.; Schmitt-Willich, H.; Darmency, V.; Gabellieri, E.; Boudou, C.; Juergens, T.; Varisco, Y.; Vokali, E.; Hickman, D. T.; Tamagnan, G.; Pfeifer, A.; Dinkelborg, L.; Muhs, A.; Stephens, A. Discovery and Preclinical Characterization of [18 F]PI-2620, a next-Generation Tau PET Tracer for the Assessment of Tau Pathology in Alzheimer's Disease and Other Tauopathies. *Eur. J. Nucl. Med. Mol. Imaging* **2019**, *46*, 2178–2189. DOI: 10.1007/s00259-019-04397-2.
(161) Walji, A. M.; Hostetler, E. D.; Selnick, H.; Zeng, Z.; Miller, P.; Bennacef, I.; Salinas, C.; Connolly, B.; Gantert, L.; Holahan, M.; O'Malley, S.; Purcell, M.; Riffel, K.; Li, J.; Balsells, J.; Obrien, J. A.; Melquist, S.; Soriano, A.; Zhang, X.; Ogawa, A.; Xu, S.; Joshi, E.; Della Rocca, J.; Hess, F. J.; Schachter, J.; Hesk, D.; Schenk, D.; Struyk, A.; Babaoglu, K.; Lohith, T. G.; Wang, Y.; Yang, K.; Fu, J.; Evelhoch, J. L.; Coleman, P. J. Discovery of 6-(Fluoro-18F)-3-(1H-Pyrrolo[2,3-c]Pyridin-1-Yl)Isoquinolin-5-Amine ([18F]-MK-6240): A Positron Emission Tomography (PET) Imaging Agent for Quantification of Neurofibrillary Tangles (NFTs). *J. Med. Chem.* **2016**, *59* (10), 4778–4789. DOI: 10.1021/acs.jmedchem.6b00166.

(162) Chisholm, T. S.; Mackey, M.; Hunter, C. A. Discovery of High-Affinity Amyloid Ligands Using a Ligand-Based Virtual Screening Pipeline. *J. Am. Chem. Soc.* **2023**, *145*, 15936–15950. DOI: 10.1021/jacs.3c03749.

(163) Bian, J.; Liu, Y.-Q.; He, J.; Lin, X.; Qiu, C.-Y.; Yu, W.-B.; Shen, Y.; Zhu, Z.-Y.; Ye, D.-Y.; Wang, J.; Chu, Y. Discovery of Styrylaniline Derivatives as Novel Alpha-Synuclein Aggregates Ligands. *Eur. J. Med. Chem.* **2021**, *226*, 113887. DOI: 10.1016/j.ejmech.2021.113887.

(164) Lunven, L.; Bonnet, H.; Yahiaoui, S.; Yi, W.; Da Costa, L.; Peuchmaur, M.; Boumendjel, A.; Chierici, S. Disruption of Fibers from the Tau Model AcPHF6 by Naturally Occurring Aurones and Synthetic Analogues. *ACS Chem. Neurosci.* **2016**, *7* (7), 995–1003. DOI: 10.1021/acschemneuro.6b00102.

(165) Ono, M.; Sahara, N.; Kumata, K.; Ji, B.; Ni, R.; Koga, S.; Dickson, D. W.; Trojanowski, J. Q.; Lee, V. M.-Y.; Yoshida, M.; Hozumi, I.; Yoshiyama, Y.; Van Swieten, J. C.; Nordberg, A.; Suhara, T.; Zhang, M.-R.; Higuchi, M. Distinct Binding of PET Ligands PBB3 and AV-1451 to Tau Fibril Strains in Neurodegenerative Tauopathies Maiko. *Brain J. Neurol.* **2017**, *140* (3), 764–780. DOI: 10.1093/brain/aww339.

(166) Klingstedt, T.; Shirani, H.; Mahler, J.; Wegenast-Braun, B. M.; Nyström, S.; Goedert, M.; Jucker, M.; Nilsson, K. P. R. Distinct Spacing between Anionic Groups: An Essential Chemical Determinant for Achieving Thiophene-Based Ligands to Distinguish β-Amyloid or Tau Polymorphic Aggregates. *Chem. - Eur. J.* **2015**, *21* (25), 9072–9082. DOI: 10.1002/chem.201500556.

(167) Terpstra, K.; Wang, Y.; Huynh, T. T.; Bandara, N.; Cho, H.-J.; Rogers, B. E.; Mirica, L. M. Divalent 2-(4-Hydroxyphenyl)Benzothiazole Bifunctional Chelators for 64Cu Positron Emission Tomography Imaging in Alzheimer's Disease. *Inorg. Chem.* **2022**, *61* (50), 20326–20336. DOI: 10.1021/acs.inorgchem.2c02740.

(168) Zhang, X.; Ran, C. Dual Functional Small Molecule Probes as Fluorophore and Ligand for Misfolding Proteins. *Curr. Org. Chem.* **2013**, *17* (6), 580–593. DOI: 10.2174/1385272811317060004.

(169) Wong, C.-Y.; Chung, L.-H.; Lu, L.; Wang, M.; He, B.; Liu, L.-J.; Leung, C.-H.; Ma, D.-L. Dual Inhibition and Monitoring of Beta-Amyloid Fibrillation by a Luminescent Iridium(III) Complex. *Curr. Alzheimer Res.* **2015**, *12*, 439–444. DOI: 10.2174/1567205012666150504144558.

(170) Leng, H.; Wang, Y.; Wang, J.; Sun, H.; Sun, A.; Pistolozzi, M.; Zhang, L.; Yan, J. Dual-Emission GFP Chromophore-Based Derivative for Imaging and Discriminating Aβ Oligomers and Aggregates. *Anal. Chem.* **2022**, *94* (4), 1999–2006. DOI: 10.1021/acs.analchem.1c03452.

(171) Wang, Y.; Qiu, Y.; Sun, A.; Xiong, Y.; Tan, H.; Shi, Y.; Yu, P.; Roy, G.; Zhang, L.; Yan, J. Dual-Functional AIE Fluorescent Probes for Imaging β-Amyloid Plaques and Lipid Droplets. *Anal. Chim. Acta* **2020**, *1133*, 109–118. DOI: 10.1016/j.aca.2020.07.073.

(172) Tan, H.; Zhou, K.; Yan, J.; Sun, H.; Pistolozzi, M.; Cui, M.; Zhang, L. Dual-Functional Red-Emitting Fluorescent Probes for Imaging Beta-Amyloid Plaques and Viscosity. *Sens. Actuators B Chem.* **2019**, *298*, 126903. DOI: 10.1016/j.snb.2019.126903.

(173) Stepanchuk, A. A.; Morgan, M. L.; Joseph, J. T.; Stys, P. K. Dual-Probe Fluorescence Spectroscopy for Sensitive Quantitation of Alzheimer's Amyloid Pathology. *Acta Neuropathol. Commun.* **2022**, *10* (1), 153. DOI: 10.1186/s40478-022-01456-y.

(174) Yang, Y.; Jia, H.-M.; Liu, B.-L. (E)-5-Styryl-1H-Indole and (E)-6-Styrylquinoline Derivatives Serve as Probes for β-Amyloid Plaques. *Molecules* **2012**, *17* (4), 4252–4265. DOI: 10.3390/molecules17044252.

(175) Chien, D. T.; Bahri, S.; Szardenings, A. K.; Walsh, J. C.; Mu, F.; Su, M.-Y.; Shankle, W. R.; Elizarov, A.; Kolb, H. C. Early Clinical PET Imaging Results with the Novel PHF-Tau Radioligand [F-18]-T807. *J. Alzheimers Dis.* **2013**, *34*, 457–468. DOI: 10.3233/JAD-122059.

(176) Li, Y.; Chen, C.; Xu, D.; Poon, C. Y.; Ho, S. L.; Zheng, R.; Liu, Q.; Song, G.; Li, H. W.; Wong, M. S. Effective Theranostic Cyanine for Imaging of Amyloid Species in Vivo and Cognitive Improvements in Mouse Model. *ACS Omega* **2018**, *3* (6), 6812–6819. DOI: 10.1021/acsomega.8b00475.

(177) Wang, Y.; Mathis, C. A.; Huang, G.-F.; Debnath, M. L.; Holt, D. P.; Shao, L.; Klunk, W. E. Effects of Lipophilicity on the Affinity and Nonspecific Binding of Iodinated Benzothiazole Derivatives. *J. Mol. Neurosci.* **2003**, *20*, 255–260. DOI: 10.1385/JMN:20:3:255.

(178) Sonawane, S. K.; Chidambaram, H.; Boral, D.; Gorantla, N. V.; Balmik, A. A.; Dangi, A.; Ramasamy, S.; Marelli, U. K.; Chinnathambi, S. EGCG Impedes Human Tau Aggregation and Interacts with Tau. *Sci. Rep.* **2020**, *10* (1), 12579. DOI: 10.1038/s41598-020-69429-6.
(179) Ilanchelian, M.; Ramaraj, R. Emission of Thioflavin T and Its Control in the Presence of DNA. *J. Photochem. Photobiol. Chem.* **2004**, *162* (1), 129–137. DOI: 10.1016/S1010-6030(03)00320-4.
(180) Fang, D.; Wen, X.; Wang, Y.; Sun, Y.; An, R.; Zhou, Y.; Ye, D.; Liu, H. Engineering of Donor-Acceptor-Donor Curcumin Analogues as near-Infrared Fluorescent Probes for in Vivo Imaging of Amyloid-β Species. *Theranostics* **2022**, *12* (7), 3178–3195. DOI: 10.7150/thno.68679.
(181) Pan, M.-L.; Mukherjee, M. T.; Patel, H. H.; Patel, B.; Constantinescu, C. C.; Mirbolooki, M. R.; Liang, C.; Mukherjee, J. Evaluation of [11C]TAZA for Amyloid β Plaque Imaging in Postmortem Human Alzheimer's Disease Brain Region and Whole Body Distribution in Rodent PET/CT. *SYNAPSE* **2016**, *70* (4), 163–176. DOI: 10.1002/syn.21893.
(182) Shao, X.; Carpenter, G. M.; Desmond, T. J.; Sherman, P.; Quesada, C. A.; Fawaz, M.; Brooks, A. F.; Kilbourn, M. R.; Albin, R. L.; Frey, K. A.; Scott, P. J. H. Evaluation of [11C] N-Methyl Lansoprazole as a Radiopharmaceutical for PET Imaging of Tau Neurofibrillary Tangles. *ACS Med. Chem. Lett.* **2012**, *3* (11), 936–941. DOI: 10.1021/ml300216t.
(183) Bandara, N.; Sharma, A. K.; Krieger, S.; Schultz, J. W.; Han, B. H.; Rogers, B. E.; Mirica, L. M. Evaluation of 64Cu-Based Radiopharmaceuticals That Target Aβ Peptide Aggregates as Diagnostic Tools for Alzheimer's Disease. *J. Am. Chem. Soc.* **2017**, *139* (36), 12550–12558. DOI: 10.1021/jacs.7b05937.
(184) Fu, H.; Cui, M.; Tu, P.; Pan, Z.; Liu, B. Evaluation of Molecules Based on the Electron Donor—Acceptor Architecture as near-Infrared β-Amyloidal-Targeting Probes. *Chem. Commun.* **2014**, *50* (80), 11875–11878. DOI: 10.1039/c4cc04907a.
(185) Chen, Y.; Ouyang, Q.; Li, Y.; Zeng, Q.; Dai, B.; Liang, Y.; Chen, B.; Tan, H.; Cui, M. Evaluation of N, O-Benzamide Difluoroboron Derivatives as near-Infrared Fluorescent Probes to Detect β-Amyloid and Tau Tangles. *Eur. J. Med. Chem.* **2022**, *227*, 113968. DOI: 10.1016/j.ejmech.2021.113968.
(186) Lockhart, A.; Ye, L.; Judd, D. B.; Merrittu, A. T.; Lowe, P. N.; Morgenstern, J. L.; Hong, G.; Gee, A. D.; Brown, J. Evidence for the Presence of Three Distinct Binding Sites for the Thioflavin T Class of Alzheimer's Disease PET Imaging Agents on β-Amyloid Peptide Fibrils. *J. Biol. Chem.* **2005**, *280* (9), 7677–7684. DOI: 10.1074/jbc.M412056200.
(187) Boukherrouba, E.; Larosa, C.; Nguyen, K.-A.; Caburet, J.; Lunven, L.; Bonnet, H.; Fortuné, A.; Boumendjel, A.; Boucherle, B.; Chierici, S.; Peuchmaur, M. Exploring the Structure-Activity Relationship of Benzylidene-2,3-Dihydro-1H-Inden-1-One Compared to Benzofuran-3(2H)-One Derivatives as Inhibitors of Tau Amyloid Fibers. *Eur. J. Med. Chem.* **2022**, *231*, 114139. DOI: 10.1016/j.ejmech.2022.114139.
(188) Zhang, W.; Oya, S.; Kung, M. P.; Hou, C.; Maier, D. L.; Kung, H. F. F-18 Stilbenes as PET Imaging Agents for Detecting β-Amyloid Plaques in the Brain. *J. Med. Chem.* **2005**, *48* (19), 5980–5988. DOI: 10.1021/jm050166g.
(189) Fodero-Tavoletti, M. T.; Okamura, N.; Furumoto, S.; Mulligan, R. S.; Connor, A. R.; Mclean, C. A.; Cao, D.; Rigopoulos, A.; Cartwright, G. A.; O'keefe, G.; Gong, S.; Adlard, P. A.; Barnham, K. J.; Rowe, C. C.; Masters, C. L.; Kudo, Y.; Cappai, R.; Yanai, K.; Villemagne, V. L. F-THK523: A Novel in Vivo Tau Imaging Ligand for Alzheimer's Disease. *Brain* **2011**, *134*, 1089–1100. DOI: 10.1093/brain/awr038.
(190) Byeon, S. R.; Jin, Y. J.; Lim, S. J.; Lee, J. H.; Yoo, K. H.; Shin, K. J.; Oh, S. J.; Kim, D. J. Ferulic Acid and Benzothiazole Dimer Derivatives with High Binding Affinity to β-Amyloid Fibrils. *Bioorg. Med. Chem. Lett.* **2007**, *17*, 4022–4025. DOI: 10.1016/j.bmcl.2007.04.079.
(191) Park, Y. D.; Park, J. H.; Hur, M. G.; Kim, S. W.; Min, J. J.; Park, S. H.; Yoo, Y. J.; Yoon, Y. J.; Yang, S. D. Fluorescent 2-Styrylpyridazin-3(2H)-One Derivatives as Probes Targeting Amyloid-Beta Plaques in Alzheimer's Disease. *Bioorg. Med. Chem. Lett.* **2012**, *22* (12), 4106–4110. DOI: 10.1016/j.bmcl.2012.04.068.

(192) Ren, W.; Zhang, J.; Peng, C.; Xiang, H.; Chen, J.; Peng, C.; Zhu, W.; Huang, R.; Zhang, H.; Hu, Y. Fluorescent Imaging of β-Amyloid Using BODIPY Based Near-Infrared Off-On Fluorescent Probe. *Bioconjug. Chem.* **2018**, *29* (10), 3459–3466. DOI: 10.1021/acs.bioconjchem.8b00623.
(193) Celej, M. S.; Jares-Erijman, E. A.; Jovin, T. M. Fluorescent N-Arylaminonaphthalene Sulfonate Probes for Amyloid Aggregation of α-Synuclein. *Biophys. J.* **2008**, *94* (12), 4867–4879. DOI: 10.1529/biophysj.107.125211.
(194) Gaur, P.; Galkin, M.; Kurochka, A.; Ghosh, S.; Yushchenko, D. A.; Shvadchak, V. V. Fluorescent Probe for Selective Imaging of α-Synuclein Fibrils in Living Cells. *ACS Chem. Neurosci.* **2021**, *12* (8), 1293–1298. DOI: 10.1021/acschemneuro.1c00090.
(195) Anumala, U. R.; Gu, J.; Lo Monte, F.; Kramer, T.; Heyny-Von Haußen, R.; Hölzer, J.; Goetschy-Meyer, V.; Schön, C.; Mall, G.; Hilger, I.; Czech, C.; Herms, J.; Schmidt, B. Fluorescent Rhodanine-3-Acetic Acids Visualize Neurofibrillary Tangles in Alzheimer's Disease Brains. *Bioorg. Med. Chem.* **2013**, *21* (17), 5139–5144. DOI: 10.1016/j.bmc.2013.06.039.
(196) Vassar, P. S.; Culling, C. F. Fluorescent Stains, with Special Reference to Amyloid and Connective Tissues. *Arch. Pathol.* **1959**, *68*, 487–498.
(197) Cheng, Y.; Ono, M.; Kimura, H.; Kagawa, S.; Nishii, R.; Kawashima, H.; Saji, H. Fluorinated Benzofuran Derivatives for PET Imaging of β-Amyloid Plaques in Alzheimer's Disease Brains. *ACS Med. Chem. Lett.* **2010**, *1* (7), 321–325. DOI: 10.1021/ml100082x.
(198) Li, Y.; Zhou, K.; Zhang, X.; Zhao, H.; Wang, X.; Dong, R.; Wang, Y.; Chen, B.; Yan, X.; Dai, J.; Sui, Y.; Zhang, J.; Cui, M. Fluorine-18-Labeled Diaryl-Azines as Improved β-Amyloid Imaging Tracers: From Bench to First-in-Human Studies. *J. Med. Chem.* **2023**, *66* (7), 4603–4616. DOI: 10.1021/acs.jmedchem.2c01503.
(199) Stephenson, K. A.; Chandra, R.; Zhuang, Z.-P.; Hou, C.; Oya, S.; Kung, M.-P.; Kung, H. F. Fluoro-Pegylated (FPEG) Imaging Agents Targeting Aβ Aggregates. *Bioconjug. Chem.* **2007**, *18* (1), 238–246. DOI: 10.1021/bc060239q.
(200) Ono, M.; Watanabe, R.; Kawashima, H.; Cheng, Y.; Kimura, H.; Watanabe, H.; Haratake, M.; Saji, H.; Nakayama, M. Fluoro-Pegylated Chalcones as Positron Emission Tomography Probes for in Vivo Imaging of β-Amyloid Plaques in Alzheimer's Disease. *J Med Chem* **2009**, *52*, 6394–6401. DOI: 10.1021/jm901057p.
(201) Sato, K.; Higuchi, M.; Iwata, N.; Saido, T. C.; Sasamoto, K. Fluoro-Substituted and 13C-Labeled Styrylbenzene Derivatives for Detecting Brain Amyloid Plaques. *Eur. J. Med. Chem.* **2004**, *39* (7), 573–578. DOI: 10.1016/j.ejmech.2004.02.013.
(202) Li, Y.; Xu, D.; Sun, A.; Ho, S. L.; Poon, C. Y.; Chan, H. N.; Ng, O. T. W.; Yung, K. K. L.; Yan, H.; Li, H. W.; Wong, M. S. Fluoro-Substituted Cyanine for Reliable: In Vivo Labelling of Amyloid-β Oligomers and Neuroprotection against Amyloid-β Induced Toxicity. *Chem. Sci.* **2017**, *8* (12), 8279–8284. DOI: 10.1039/c7sc03974c.
(203) Sundaram, G. S. M.; Dhavale, D. D.; Prior, J. L.; Yan, P.; Cirrito, J.; Rath, N. P.; Laforest, R.; Cairns, N. J.; Lee, J. M.; Kotzbauer, P. T.; Sharma, V. Fluselenamyl: A Novel Benzoselenazole Derivative for Pet Detection of Amyloid Plaques (Aβ) in Alzheimer's Disease. *Sci. Rep.* **2016**, *6*, 35636. DOI: 10.1038/srep35636.
(204) Xie, T.; Li, Y.; Tian, C.; Yuan, C.; Dai, B.; Wang, S.; Zhou, K.; Liu, J.; Tan, H.; Liang, Y.; Dai, J.; Chen, B.; Cui, M. Fused Cycloheptatriene–BODIPY Is a High-Performance Near-Infrared Probe to Image Tau Tangles. *J. Med. Chem.* **2022**, *65* (21), 14527–14538. DOI: 10.1021/acs.jmedchem.2c00859.
(205) Leng, H.; Yang, J.; Long, L.; Yan, Y.; Shi, W.-J.; Zhang, L.; Yan, J. GFP-Based Red-Emissive Fluorescent Probes for Dual Imaging of β-Amyloid Plaques and Mitochondrial Viscosity. *Bioorganic Chem.* **2023**, *136*, 106540. DOI: 10.1016/j.bioorg.2023.106540.
(206) Man, B. Y.-W.; Chan, H.-M.; Leung, C.-H.; Chan, D. S.-H.; Bai, L.-P.; Jiang, Z.-H.; Li, H.-W.; Ma, D.-L. Group 9 Metal-Based Inhibitors of β-Amyloid (1–40) Fibrillation as Potential Therapeutic Agents for Alzheimer's Disease. *Chem. Sci.* **2011**, *2* (5), 917. DOI: 10.1039/c0sc00636j.

(207) Fawaz, M. V.; Brooks, A. F.; Rodnick, M. E.; Carpenter, G. M.; Shao, X.; Desmond, T. J.; Sherman, P.; Quesada, C. A.; Hockley, B. G.; Kilbourn, M. R.; Albin, R. L.; Frey, K. A.; Scott, P. J. H. High Affinity Radiopharmaceuticals Based upon Lansoprazole for PET Imaging of Aggregated Tau in Alzheimer"s Disease and Progressive Supranuclear Palsy: Synthesis, Preclinical Evaluation, and Lead Selection. *ACS Chem. Neurosci.* **2014**, *5* (8), 718–730. DOI: 10.1021/cn500103u.

(208) Mallesh, R.; Khan, J.; Gharai, P. K.; Ghosh, S.; Garg, S.; Arshi, M. U.; Ghosh, S. High-Affinity Fluorescent Probes for the Detection of Soluble and Insoluble Aβ Deposits in Alzheimer's Disease. *ACS Chem. Neurosci.* **2023**, *14* (8), 1459–1473. DOI: 10.1021/acschemneuro.2c00787.

(209) Tao, R.; Wang, N.; Shen, T.; Tan, Y.; Ren, Y.; Wei, W.; Liao, M.; Tan, D.; Tang, C.; Xu, N.; Wang, H.; Liu, X.; Li, X. High-Fidelity Imaging of Amyloid-Beta Deposits with an Ultrasensitive Fluorescent Probe Facilitates the Early Diagnosis and Treatment of Alzheimer's Disease. *Theranostics* **2022**, *12* (6), 2549–2559. DOI: 10.7150/thno.68743.

(210) Qin, L.; Vastl, J.; Gao, J. Highly Sensitive Amyloid Detection Enabled by Thioflavin T Dimers. *Mol. Biosyst.* **2010**, *6* (10), 1791–1795. DOI: 10.1039/c005255h.

(211) Fu, H.; Cui, M.; Zhao, L.; Tu, P.; Zhou, K.; Dai, J.; Liu, B. Highly Sensitive Near-Infrared Fluorophores for in Vivo Detection of Amyloid-β Plaques in Alzheimer's Disease. *J. Med. Chem.* **2015**, *58* (17), 6972–6983. DOI: 10.1021/acs.jmedchem.5b00861.

(212) Gan, C.; Zhao, Z.; Nan, D. D.; Yin, B.; Hu, J. Homoisoflavonoids as Potential Imaging Agents for β-Amyloid Plaques in Alzheimer's Disease. *Eur. J. Med. Chem.* **2014**, *76*, 125–131. DOI: 10.1016/j.ejmech.2014.02.020.

(213) Zhuang, Z. P.; Kung, M. P.; Hou, C.; Plössl, K.; Skovronsky, D.; Gur, T. L.; Trojanowski, J. Q.; Lee, V. M. Y.; Kung, H. F. IBOX(2-(4′-Dimethylaminophenyl)-6-Iodobenzoxazole): A Ligand for Imaging Amyloid Plaques in the Brain. *Nucl. Med. Biol.* **2001**, *28* (8), 887–894. DOI: 10.1016/S0969-8051(01)00264-5.

(214) Kaide, S.; Watanabe, H.; Shimizu, Y.; Iikuni, S.; Nakamoto, Y.; Hasegawa, M.; Itoh, K.; Ono, M. Identification and Evaluation of Bisquinoline Scaffold as a New Candidate for α-Synuclein-PET Imaging. *ACS Chem. Neurosci.* **2020**, *11* (24), 4254–4261. DOI: 10.1021/acschemneuro.0c00523.

(215) Miranda-Azpiazu, P.; Svedberg, M.; Higuchi, M.; Ono, M.; Jia, Z.; Sunnemark, D.; Elmore, C. S.; Schou, M.; Varrone, A. Identification and in Vitro Characterization of C05-01, a PPB3 Derivative with Improved Affinity for Alpha-Synuclein. *Brain Res.* **2020**, *1749*, 147131. DOI: 10.1016/j.brainres.2020.147131.

(216) Ferrie, J. J.; Lengyel-Zhand, Z.; Janssen, B.; Lougee, M. G.; Giannakoulias, S.; Hsieh, C.-J.; Pagar, V. V.; Weng, C.-C.; Xu, H.; Graham, T. J. A.; Lee, V. M.-Y.; Mach, R. H.; Petersson, E. J. Identification of a Nanomolar Affinity α-Synuclein Fibril Imaging Probe by Ultra-High Throughput in Silico Screening. *Chem. Sci.* **2020**, *11* (7), 12746–12754. DOI: 10.1039/d0sc02159h.

(217) Roy, R.; Paul, S. Illustrating the Effect of Small Molecules Derived from Natural Resources on Amyloid Peptides. *J. Phys. Chem. B* **2023**, *127* (3), 600–615. DOI: 10.1021/acs.jpcb.2c07607.

(218) Klunk, W. E.; Bacskai, B. J.; Mathis, C. A.; Kajdasz, S. T.; McLellan, M. E.; Frosch, M. P.; Debnath, M. L.; Holt, D. P.; Wang, Y.; Hyman, B. T. Imaging Aβ Plaques in Living Transgenic Mice with Multiphoton Microscopy and Methoxy-X04, a Systemically Administered Congo Red Derivative. *J. Neuropathol. Exp. Neurol.* **2002**, *61* (9), 797–805. DOI: 10.1093/jnen/61.9.797.

(219) Maruyama, M.; Shimada, H.; Suhara, T.; Shinotoh, H.; Ji, B.; Maeda, J.; Zhang, M. R.; Trojanowski, J. Q.; Lee, V. M. Y.; Ono, M.; Masamoto, K.; Takano, H.; Sahara, N.; Iwata, N.; Okamura, N.; Furumoto, S.; Kudo, Y.; Chang, Q.; Saido, T. C.; Takashima, A.; Lewis, J.; Jang, M. K.; Aoki, I.; Ito, H.; Higuchi, M. Imaging of Tau Pathology in a Tauopathy Mouse Model and in Alzheimer Patients Compared to Normal Controls. *Neuron* **2013**, *79* (6), 1094–1108. DOI: 10.1016/j.neuron.2013.07.037.

(220) Rowe, C. C.; Ng, S.; Ackermann, U.; Gong, S. J.; Pike, K.; Savage, G.; Cowie, T. F.; Dickinson, K. L.; Maruff, P.; Darby, D.; Smith, C.; Woodward, M.; Merory, J.; Tochon-Danguy, H.; O'Keefe, G.; Klunk, W. E.; Mathis, C. A.; Price, J. C.; Masters, C. L.; Villemagne, V. L. Imaging β-Amyloid Burden in Aging and Dementia. *Neurology* **2007**, *68* (20), 1718–1725. DOI: 10.1212/01.wnl.0000261919.22630.ea.

(221) Blass, B. Imidazo[2,1]Thiazol-3-One Derivatives Useful as Diagnostic Agents for Alzheimer's Disease. *ACS Med. Chem. Lett.* **2014**, *5* (6), 619–620. DOI: 10.1021/ml500147n.

(222) Kung, M. P.; Hou, C.; Zhuang, Z. P.; Zhang, B.; Skovronsky, D.; Trojanowski, J. Q.; Lee, V. M. Y.; Kung, H. F. IMPY: An Improved Thioflavin-T Derivative for in Vivo Labeling of β-Amyloid Plaques. *Brain Res.* **2002**, *956* (2), 202–210. DOI: 10.1016/S0006-8993(02)03436-4.

(223) Uzuegbunam, B. C.; Li, J.; Paslawski, W.; Weber, W.; Svenningsson, P.; Ågren, H.; Hooshyar Yousefi, B. In Silico and In Vitro Study towards the Rational Design of 4,4′-Disarylbisthiazoles as a Selective α-Synucleinopathy Biomarker. *Int. J. Mol. Sci.* **2023**, *24* (22), 16445. DOI: 10.3390/ijms242216445.

(224) Graham, T. J. A.; Lindberg, A.; Tong, J.; Stehouwer, J. S.; Vasdev, N.; Mach, R. H.; Mathis, C. A. In Silico Discovery and Subsequent Characterization of Potent 4R-Tauopathy Positron Emission Tomography Radiotracers. *J. Med. Chem.* **2023**, *66* (15), 10628–10638. DOI: 10.1021/acs.jmedchem.3c00775.

(225) Verdurand, M.; Levigoureux, E.; Zeinyeh, W.; Berthier, L.; Mendjel-Herda, M.; Cadarossanesaib, F.; Bouillot, C.; Iecker, T.; Terreux, R.; Lancelot, S.; Chauveau, F.; Billard, T.; Zimmer, L. In Silico, in Vitro, and in Vivo Evaluation of New Candidates for α-Synuclein PET Imaging. *Mol. Pharm.* **2018**, *15* (8), 3153–3166. DOI: 10.1021/acs.molpharmaceut.8b00229.

(226) Li, M.; Zhao, C.; Yang, X.; Ren, J.; Xu, C.; Qu, X. In Situ Monitoring Alzheimer's Disease β-Amyloid Aggregation and Screening of Aβ Inhibitors Using a Perylene Probe. *Small* **2013**, *9* (1), 52–55. DOI: 10.1002/smll.201201543.

(227) Hellström-Lindahl, E.; Westermark, P.; Antoni, G.; Estrada, S. In Vitro Binding of [3H]PIB to Human Amyloid Deposits of Different Types. *Amyloid* **2014**, *21* (1), 21–27. DOI: 10.3109/13506129.2013.860895.

(228) Fodero-Tavoletti, M. T.; Mulligan, R. S.; Okamura, N.; Furumoto, S.; Rowe, C. C.; Kudo, Y.; Masters, C. L.; Cappai, R.; Yanai, K.; Villemagne, V. L. In Vitro Characterisation of BF227 Binding to α-Synuclein/Lewy Bodies. *Eur. J. Pharmacol.* **2009**, *617*, 54–58. DOI: 10.1016/j.ejphar.2009.06.042.

(229) Ye, L.; Velasco, A.; Fraser, G.; Beach, T. G.; Sue, L.; Osredkar, T.; Libri, V.; Spillantini, M. G.; Goedert, M.; Lockhart, A. In Vitro High Affinity A-Synuclein Binding Sites for the Amyloid Imaging Agent PIB Are Not Matched by Binding to Lewy Bodies in Postmortem Human Brain 1. *J Neurochem* **2008**, *105*, 1428–1437. DOI: 10.1111/j.1471-4159.2008.05245.x.

(230) Wu, J.; Shao, C.; Ye, X.; Di, X.; Li, D.; Zhao, H.; Zhang, B.; Chen, G.; Liu, H.-K.; Qian, Y. In Vivo Brain Imaging of Amyloid-β Aggregates in Alzheimer's Disease with a Near-Infrared Fluorescent Probe. *ACS Sens.* **2021**, *6* (3), 863–870. DOI: 10.1021/acssensors.0c01914.

(231) Okamura, N.; Mori, M.; Furumoto, S.; Yoshikawa, T.; Harada, R.; Ito, S.; Fujikawa, Y.; Arai, H.; Yanai, K.; Kudo, Y. In Vivo Detection of Amyloid Plaques in the Mouse Brain Using the Near-Infrared Fluorescence Probe THK-265. *J. Alzheimers Dis.* **2011**, *23* (1), 37–48. DOI: 10.3233/JAD-2010-100270.

(232) Hintersteiner, M.; Enz, A.; Frey, P.; Jaton, A. L.; Kinzy, W.; Kneuer, R.; Neumann, U.; Rudin, M.; Staufenbiel, M.; Stoeckli, M.; Wiederhold, K. H.; Gremlich, H. U. In Vivo Detection of Amyloid-β Deposits by near-Infrared Imaging Using an Oxazine-Derivative Probe. *Nat. Biotechnol.* **2005**, *49*, 2725–2730. DOI: 10.1038/nbt1085.

(233) Cheng, Y.; Zhu, B.; Deng, Y.; Zhang, Z. In Vivo Detection of Cerebral Amyloid Fibrils with Smart Dicynomethylene-4h-Pyran-Based Fluorescence Probe. *Anal. Chem.* **2015**, *87* (9), 4781–4787. DOI: 10.1021/acs.analchem.5b00017.

(234) Watanabe, H.; Ono, M.; Saji, H. In Vivo Fluorescence Imaging of β-Amyloid Plaques with Push-Pull Dimethylaminothiophene Derivatives. *Chem. Commun.* **2015**, *51* (96), 17124–17127. DOI: 10.1039/c5cc06628j.

(235) Suemoto, T.; Okamura, N.; Shiomitsu, T.; Suzuki, M.; Shimadzu, H.; Akatsu, H.; Yamamoto, T.; Kudo, Y.; Sawada, T. In Vivo Labeling of Amyloid with BF-108. *Neurosci. Res.* **2004**, *48* (1), 65–74. DOI: 10.1016/j.neures.2003.09.005.

(236) Fu, H.; Peng, C.; Liang, Z.; Dai, J.; Liu, B.; Cui, M. In Vivo Near-Infrared and Cerenkov Luminescence Imaging of Amyloid-β Deposits in the Brain: A Fluorinated Small Molecule Used for Dual-Modality Imaging. *Chem. Commun.* **2016**, *52* (86), 12745–12748. DOI: 10.1039/C6CC06995A.

(237) Zhu, J.; Zhou, L.; Li, Y.; Chen, S.; Yan, J.; Zhang, L. In Vivo Near-Infrared Fluorescence Imaging of Amyloid-β Plaques with a Dicyanoisophorone-Based Probe. *Anal. Chim. Acta* **2017**, *961*, 112–118. DOI: 10.1016/j.aca.2017.01.017.
(238) Akasaka, T.; Watanabe, H.; Ono, M. In Vivo Near-Infrared Fluorescence Imaging Selective for Soluble Amyloid β Aggregates Using y-Shaped BODIPY Derivative. *J. Med. Chem.* **2023**, *66* (20), 14029–14046. DOI: 10.1021/acs.jmedchem.3c01057.
(239) Nesterov, E. E.; Skoch, J.; Hyman, B. T.; Klunk, W. E.; Bacskai, B. J.; Swager, T. M. In Vivo Optical Imaging of Amyloid Aggregates in Brain: Design of Fluorescent Markers. *Angew. Chem. - Int. Ed.* **2005**, *44* (34), 5452–5456. DOI: 10.1002/anie.200500845.
(240) Chen, C. J.; Bando, K.; Ashino, H.; Taguchi, K.; Shiraishi, H.; Shima, K.; Fujimoto, O.; Kitamura, C.; Matsushima, S.; Uchida, K.; Nakahara, Y.; Kasahara, H.; Minamizawa, T.; Jiang, C.; Zhang, M. R.; Ono, M.; Tokunaga, M.; Suhara, T.; Higuchi, M.; Yamada, K.; Ji, B. In Vivo SPECT Imaging of Amyloid-β Deposition with Radioiodinated Imidazo[1, 2-α]Pyridine Derivative DRM106 in a Mouse Model of Alzheimer's Disease. *J. Nucl. Med.* **2015**, *56* (1), 120–126. DOI: 10.2967/jnumed.114.146944.
(241) Kumar, A.; Moody, L.; Olaivar, J. F.; Lewis, N. A.; Khade, R. L.; Holder, A. A.; Zhang, Y.; Rangachari, V. Inhibition of AB42 Peptide Aggregation by a Binuclear Ruthenium(II)-Platinum(II) Complex: Potential for Multimetal Organometallics as Anti-Amyloid Agents. *ACS Chem. Neurosci.* **2010**, *1* (10), 691–701. DOI: 10.1021/cn100046m.
(242) Lu, L.; Zhong, H. J.; Wang, M.; Ho, S. L.; Li, H. W.; Leung, C. H.; Ma, D. L. Inhibition of Beta-Amyloid Fibrillation by Luminescent Iridium(III) Complex Probes. *Sci. Rep.* **2015**, *5* (1), 1–9. DOI: 10.1038/srep14619.
(243) Yang, W.; Wong, Y.; Ng, O. T. W.; Bai, L.-P.; Kwong, D. W. J.; Ke, Y.; Jiang, Z.-H.; Li, H.-W.; Yung, K. K. L.; Wong, M. S. Inhibition of Beta-Amyloid Peptide Aggregation by Multifunctional Carbazole-Based Fluorophores. *Angew. Chem. Int. Ed.* **2012**, *51* (8), 1804–1810. DOI: 10.1002/anie.201104150.
(244) DeToma, A. S.; Krishnamoorthy, J.; Nam, Y.; Lee, H. J.; Brender, J. R.; Kochi, A.; Lee, D.; Onnis, V.; Congiu, C.; Manfredini, S.; Vertuani, S.; Balboni, G.; Ramamoorthy, A.; Lim, M. H. Interaction and Reactivity of Synthetic Aminoisoflavones with Metal-Free and Metal-Associated Amyloid-β. *Chem. Sci.* **2014**, *5* (12), 4851–4862. DOI: 10.1039/C4SC01531B.
(245) Thompson, P. W.; Ye, L.; Morgenstern, J. L.; Sue, L.; Beach, T. G.; Judd, D. J.; Shipley, N. J.; Libri, V.; Lockhart, A. Interaction of the Amyloid Imaging Tracer FDDNP with Hallmark Alzheimer's Disease Pathologies. *J. Neurochem.* **2009**, *109* (2), 623–630. DOI: 10.1111/j.1471-4159.2009.05996.x.
(246) Zhou, Y.; Hua, J.; Ding, D.; Tang, Y. Interrogating Amyloid Aggregation with Aggregation-Induced Emission Fluorescence Probes. *Biomaterials* **2022**, *286*, 121605. DOI: 10.1016/j.biomaterials.2022.121605.
(247) Zhang, J.; Wang, J.; Sandberg, A.; Wu, X.; Nyström, S.; LeVine, H.; Konradsson, P.; Hammarström, P.; Durbeej, B.; Lindgren, M. Intramolecular Proton and Charge Transfer of Pyrene-Based *Trans*-Stilbene Salicylic Acids Applied to Detection of Aggregated Proteins. *ChemPhysChem* **2018**, *19* (22), 3001–3009. DOI: 10.1002/cphc.201800823.
(248) Lee, C. W.; Zhuang, Z. P.; Kung, M. P.; Plössl, K.; Skovronsky, D.; Gur, T.; Hou, C.; Trojanowski, J. Q.; Lee, V. M. Y.; Kung, H. F. Isomerization of (Z,Z) to (E,E)1-Bromo-2,5-Bis-(3- Hydroxycarbonyl-4-Hydroxy)-Styrylbenzene in Strong Base: Probes for Amyloid Plaques in the Brain. *J. Med. Chem.* **2001**, *44* (14), 2270–2275. DOI: 10.1021/jm010161t.
(249) Price, L. C.; Buescher, R. W. Kinetics of Alkaline Degradation of the Food Pigments Curcumin and Curcuminoids. *J. Food Sci.* **1997**, *62* (2), 267–269. DOI: 10.1111/j.1365-2621.1997.tb03982.x.
(250) Wren, M. C.; Lashley, T.; Årstad, E.; Sander, K. Large Inter- and Intra-Case Variability of First Generation Tau PET Ligand Binding in Neurodegenerative Dementias. *Acta Neuropathol. Commun.* **2018**, *6* (1), 34. DOI: 10.1186/s40478-018-0535-z.
(251) Cisek, K.; Jensen, J. R.; Honson, N. S.; Schafer, K. N.; Cooper, G. L.; Kuret, J. Ligand Electronic Properties Modulate Tau Filament Binding Site Density. *Biophys. Chem.* **2012**, *170*, 25–33. DOI: 10.1016/j.bpc.2012.09.001.

(252) Jensen, J. R.; Cisek, K.; Honson, N. S.; Kuret, J. Ligand Polarizability Contributes to Tau Fibril Binding Affinity. *Bioorg. Med. Chem.* **2011**, *19* (17), 5147–5154. DOI: 10.1016/j.bmc.2011.07.016.

(253) Gu, Y.; Ding, Z.; Zheng, C.; Xu, Y.; Liu, T.; Mao, C.; Ran, C.; Yang, J.; Wang, P. Light-Controlled Fluorescent Probes for Precisely Monitoring Brain Amyloid-β Aggregates in Alzheimer's Disease. *Chem. Eng. J.* **2022**, *446*, 137385. DOI: 10.1016/j.cej.2022.137385.

(254) Mathis, C. A.; Holt, D. P.; Wang, Y.; Huang, G.-F.; Debnath, M. L.; Klunk, W. E. Lipophilic 11C-Labelled Thioflavin-T Analogues for Imaging Amyloid Plaques in Alzheimer's Disease. *J. Label. Compd. Radiopharm.* **2001**, *44* (S1), S26–S28. DOI: 10.1002/jlcr.2580440110.

(255) Kocsis, I.; Sanna, E.; Hunter, C. A. Liposome Enhanced Detection of Amyloid Protein Aggregates. *Org. Lett.* **2021**, *23* (3), 647–650. DOI: 10.1021/acs.orglett.0c03597.

(256) Seneca, N.; Cai, L.; Liow, J.-S.; Zoghbi, S.; Gladding, R.; Little, J.; Aisen, P.; Hong, J.; Pike, V.; Innis, R. Low Retention of [S-Methyl-11C]MeS-IMPY to β-Amyloid Plaques in Patients with Alzheimers Disease. *Curr. Radiopharm.* **2010**, *2* (2), 129–136. DOI: 10.2174/1874471010902020129.

(257) Klingstedt, T.; Nilsson, K. P. R. Luminescent Conjugated Poly- and Oligo-Thiophenes: Optical Ligands for Spectral Assignment of a Plethora of Protein Aggregates. *Biochem. Soc. Trans.* **2012**, *40* (4), 704–710. DOI: 10.1042/BST20120009.

(258) Sanna, E.; Rodrigues, M.; Fagan, S. G.; Chisholm, T. S.; Kulenkampff, K.; Klenerman, D.; Spillantini, M. G.; Aigbirhio, F. I.; Hunter, C. A. Mapping the Binding Site Topology of Amyloid Protein Aggregates Using Multivalent Ligands. *Chem. Sci.* **2021**, *12* (25), 8892–8899. DOI: 10.1039/d1sc01263k.

(259) Hayne, D. J.; Lim, S.; Donnelly, P. S. Metal Complexes Designed to Bind to Amyloid-β for the Diagnosis and Treatment of Alzheimer's Disease. *Chem. Soc. Rev.* **2014**, *43* (19), 6701–6715. DOI: 10.1039/c4cs00026a.

(260) Huang, Y.; Cho, H.-J.; Bandara, N.; Sun, L.; Tran, D.; Rogers, B. E.; Mirica, L. M. Metal-Chelating Benzothiazole Multifunctional Compounds for the Modulation and 64Cu PET Imaging of Aβ Aggregation. *Chem. Sci.* **2020**, *11* (30), 7789–7799. DOI: 10.1039/D0SC02641G.

(261) Caprathe, B. W.; Gilmore, J. L.; Hays, S. J.; Jaen, J. C.; III, H. L. Method of Imaging Amyloid Deposits. US6001331A, 1999.

(262) Muthuraj, B.; Chowdhury, S. R.; Iyer, P. K. Modulation of Amyloid-β Fibrils into Mature Microrod-Shaped Structure by Histidine Functionalized Water-Soluble Perylene Diimide. *ACS Appl. Mater. Interfaces* **2015**, *7* (38), 21233–21234. DOI: 10.1021/acsami.5b07260.

(263) Gao, X.; Wang, L.; Huang, H. L.; Wang, L. L.; Yao, J. L.; Shi, S.; Yao, T. M. Molecular "Light Switch" [Ru(Phen)2dppzidzo]2+ Monitoring the Aggregation of Tau. *Analyst* **2015**, *140* (22), 7513–7517. DOI: 10.1039/c5an01624j.

(264) Watanabe, H.; Ono, M.; Matsumura, K.; Yoshimura, M.; Kimura, H.; Saji, H. Molecular Imaging of β-Amyloid Plaques with near-Infrared Boron Dipyrromethane (BODIPY)-Based Fluorescent Probes. *Mol. Imaging* **2013**, *12* (5), 338–347. DOI: 10.2310/7290.2013.00049.

(265) Ng, K. P.; Pascoal, T. A.; Mathotaarachchi, S.; Therriault, J.; Kang, M. S.; Shin, M.; Guiot, M. C.; Guo, Q.; Harada, R.; Comley, R. A.; Massarweh, G.; Soucy, J. P.; Okamura, N.; Gauthier, S.; Rosa-Neto, P. Monoamine Oxidase B Inhibitor, Selegiline, Reduces 18F-THK5351 Uptake in the Human Brain. *Alzheimers Res. Ther.* **2017**, *9* (1), 25. DOI: 10.1186/s13195-017-0253-y.

(266) Dai, Y.; Fang, T.; Xu, Y.; Jiang, T.; Qiao, J. Multi-Fluorine Labeled Indanone Derivatives as Potential MRI Imaging Probes for β-Amyloid Plaques. *Chem. Biol. Drug Des.* **2022**, *0*, 1–12. DOI: 10.1111/cbdd.14162.

(267) Kumar, B.; Thakur, A.; Dwivedi, A. R.; Kumar, R.; Kumar, V. Multi-Target-Directed Ligands as an Effective Strategy for the Treatment of Alzheimer's Disease. *Curr. Med. Chem.* **2022**, *29* (10), 1757–1803. DOI: 10.2174/0929867328666210512005508.

(268) Zha, Z.; Choi, S. R.; Ploessl, K.; Lieberman, B. P.; Qu, W.; Hefti, F.; Mintun, M.; Skovronsky, D.; Kung, H. F. Multidentate 18F-Polypegylated Styrylpyridines As Imaging Agents for Aβ Plaques in Cerebral Amyloid Angiopathy (CAA). *J. Med. Chem.* **2011**, *54* (23), 8085–8098. DOI: 10.1021/jm2009106.

(269) Zhang, T.; Chen, X.; Yuan, C.; Pang, X.; Shangguan, P.; Liu, Y.; Han, L.; Sun, J.; Lam, J. W. Y.; Liu, Y.; Wang, J.; Shi, B.; Zhong Tang, B. Near-Infrared Aggregation-Induced Emission Luminogens for In Vivo Theranostics of Alzheimer's Disease. *Angew. Chem. Int. Ed.* **2023**, *62* (2), e202211550. DOI: 10.1002/anie.202211550.
(270) Ma, L.; Geng, Y.; Zhang, G.; Hu, Z.; James, T. D.; Wang, X.; Wang, Z. Near-Infrared Bodipy-Based Molecular Rotors for β-Amyloid Imaging In Vivo. *Adv. Healthc. Mater.* **2023**, *12* (25), 2300733. DOI: 10.1002/adhm.202300733.
(271) Hou, S. S.; Yang, J.; Lee, J. H.; Kwon, Y.; Calvo-Rodriguez, M.; Bao, K.; Ahn, S.; Kashiwagi, S.; Kumar, A. T. N.; Bacskai, B. J.; Choi, H. S. Near-Infrared Fluorescence Lifetime Imaging of Amyloid-β Aggregates and Tau Fibrils through the Intact Skull of Mice. *Nat. Biomed. Eng.* **2023**, *7*, 270–280. DOI: 10.1038/s41551-023-01003-7.
(272) Zhang, X.; Tian, Y.; Zhang, C.; Tian, X.; Ross, A. W.; Moir, R. D.; Sun, H.; Tanzi, R. E.; Moore, A.; Ran, C. Near-Infrared Fluorescence Molecular Imaging of Amyloid Beta Species and Monitoring Therapy in Animal Models of Alzheimer's Disease. *Proc. Natl. Acad. Sci. U. S. A.* **2015**, *112* (31), 9734–9739. DOI: 10.1073/pnas.1505420112.
(273) Rai, H.; Gupta, S.; Kumar, S.; Yang, J.; Singh, S. K.; Ran, C.; Modi, G. Near-Infrared Fluorescent Probes as Imaging and Theranostic Modalities for Amyloid-Beta and Tau Aggregates in Alzheimer's Disease. *J. Med. Chem.* **2022**, *65* (13), 8550–8595. DOI: 10.1021/acs.jmedchem.1c01619.
(274) Tong, H.; Lou, K.; Wang, W. Near-Infrared Fluorescent Probes for Imaging of Amyloid Plaques in Alzheimer's Disease. *Acta Pharm. Sin. B* **2015**, *5* (1), 25–33. DOI: 10.1016/j.apsb.2014.12.006.
(275) Fodero-Tavoletti, M. T.; Smith, D. P.; Mclean, C. A.; Adlard, P. A.; Barnham, K. J.; Foster, L. E.; Leone, L.; Perez, K.; Cortés, M.; Culvenor, J. G.; Li, Q.-X.; Laughton, K. M.; Rowe, C. C.; Masters, C. L.; Cappai, R.; Villemagne, V. L. Neurobiology of Disease In Vitro Characterization of Pittsburgh Compound-B Binding to Lewy Bodies. *J. Neurosci.* **2007**, *27* (39), 10365–10371. DOI: 10.1523/JNEUROSCI.0630-07.2007.
(276) Okamura, N.; Suemoto, T.; Shimadzu, H.; Suzuki, M.; Shiomitsu, T.; Akatsu, H.; Yamamoto, T.; Staufenbiel, M.; Yanai, K.; Arai, H.; Sasaki, H.; Kudo, Y.; Sawada, T. Neurobiology of Disease Styrylbenzoxazole Derivatives for In Vivo Imaging of Amyloid Plaques in the Brain. *J Neurosci* **2004**, *24* (10), 2535–2541. DOI: 10.1523/JNEUROSCI.4456-03.2004.
(277) Yan, J. W.; Zhu, J. Y.; Zhou, K. X.; Wang, J. S.; Tan, H. Y.; Xu, Z. Y.; Chen, S. B.; Lu, Y. T.; Cui, M. C.; Zhang, L. Neutral Merocyanine Dyes: For: In Vivo NIR Fluorescence Imaging of Amyloid-β Plaques. *Chem. Commun.* **2017**, *53* (71), 9910–9913. DOI: 10.1039/c7cc05056a.
(278) Chandra, R.; Oya, S.; Kung, M. P.; Hou, C.; Jin, L. W.; Kung, H. F. New Diphenylacetylenes as Probes for Positron Emission Tomographic Imaging of Amyloid Plaques. *J. Med. Chem.* **2007**, *50* (10), 2415–2423. DOI: 10.1021/jm070090j.
(279) Xu, M.; Li, R.; Li, X.; Lv, G.; Li, S.; Sun, A.; Zhou, Y.; Yi, T. NIR Fluorescent Probes with Good Water-Solubility for Detection of Amyloid Beta Aggregates in Alzheimer's Disease. *J. Mater. Chem. B* **2019**, *7* (36), 5535–5540. DOI: 10.1039/C9TB01012B.
(280) McKinney, B.; Grubb, C. Non-Specificity of Thioflavine-T as an Amyloid Stain. *Nature* **1965**, *205* (4975), 1023–1024. DOI: 10.1038/2051023b0.
(281) Okamura, N.; Furumoto, S.; Harada, R.; Tago, T.; Yoshikawa, T.; Fodero-Tavoletti, M.; Mulligan, R. S.; Villemagne, V. L.; Akatsu, H.; Yamamoto, T.; Arai, H.; Iwata, R.; Yanai, K.; Kudo, Y. Novel 18F-Labeled Arylquinoline Derivatives for Noninvasive Imaging of Tau Pathology in Alzheimer Disease. *J. Nucl. Med.* **2013**, *54* (8), 1420–1427. DOI: 10.2967/jnumed.112.117341.
(282) Ono, M.; Cheng, Y.; Kimura, H.; Cui, M.; Kagawa, S.; Nishii, R.; Saji, H. Novel 18F-Labeled Benzofuran Derivatives with Improved Properties for Positron Emission Tomography (PET) Imaging of β-Amyloid Plaques in Alzheimer's Brains. *J. Med. Chem.* **2011**, *54* (8), 2971–2979. DOI: 10.1021/jm200057u.
(283) Cui, M.; Ono, M.; Kimura, H.; Ueda, M.; Nakamoto, Y.; Togashi, K.; Okamoto, Y.; Ihara, M.; Takahashi, R.; Liu, B.; Saji, H. Novel 18F-Labeled Benzoxazole Derivatives as Potential Positron Emission

Tomography Probes for Imaging of Cerebral β-Amyloid Plaques in Alzheimers Disease. *J. Med. Chem.* **2012**, *55* (21), 9136–9145. DOI: 10.1021/jm300251n.
(284) Li, Z.; Cui, M.; Zhang, J.; Dai, J.; Zhang, X.; Chen, P.; Jia, H.; Liu, B. Novel 18F-Labeled Dibenzylideneacetone Derivatives as Potential Positron Emission Tomography Probes for in Vivo Imaging of β-Amyloid Plaques. *Eur. J. Med. Chem.* **2014**, *84*, 628–638. DOI: 10.1016/j.ejmech.2014.07.070.
(285) Duan, X. H.; Qiao, J. P.; Yang, Y.; Cui, M. C.; Zhou, J. N.; Liu, B. L. Novel Anilinophthalimide Derivatives as Potential Probes for β-Amyloid Plaque in the Brain. *Bioorg. Med. Chem.* **2010**, *18* (3), 1337–1343. DOI: 10.1016/j.bmc.2009.12.023.
(286) Ono, M.; Kawashima, H.; Nonaka, A.; Kawai, T.; Haratake, M.; Mori, H.; Kung, M. P.; Kung, H. F.; Saji, H.; Nakayama, M. Novel Benzofuran Derivatives for PET Imaging of β-Amyloid Plaques in Alzheimer's Disease Brains. *J. Med. Chem.* **2006**, *49* (9), 2725–2730. DOI: 10.1021/jm051176k.
(287) Watanabe, H.; Ono, M.; Ariyoshi, T.; Katayanagi, R.; Saji, H. Novel Benzothiazole Derivatives as Fluorescent Probes for Detection of β-Amyloid and α-Synuclein Aggregates. *ACS Chem. Neurosci.* **2017**, *8* (8), 1656–1662. DOI: 10.1021/acschemneuro.6b00450.
(288) Ono, M.; Haratake, M.; Mori, H.; Nakayama, M. Novel Chalcones as Probes for in Vivo Imaging of β-Amyloid Plaques in Alzheimer's Brains. *Bioorg. Med. Chem.* **2007**, *15* (21), 6802–6809. DOI: 10.1016/j.bmc.2007.07.052.
(289) Lv, G.; Xu, Y.; Yang, J.; Li, W.; Li, C.; Sun, A. Novel D–π-A Type near-Infrared Fluorescent Probes for the Detection of Aβ40 Aggregates. *Analyst* **2020**, *145* (20), 6579–6585. DOI: 10.1039/D0AN01180K.
(290) Yang, Y.; Duan, X. H.; Deng, J. Y.; Jin, B.; Jia, H. M.; Liu, B. L. Novel Imaging Agents for β-Amyloid Plaque Based on the N-Benzoylindole Core. *Bioorg. Med. Chem. Lett.* **2011**, *21* (18), 5594–5597. DOI: 10.1016/j.bmcl.2011.06.077.
(291) Qiao, J. P.; Gan, C. S.; Wang, C. W.; Ge, J. F.; Nan, D. D.; Pan, J.; Zhou, J. N. Novel Indanone Derivatives as Potential Imaging Probes for β-Amyloid Plaques in the Brain. *ChemBioChem* **2012**, *13* (11), 1652–1662. DOI: 10.1002/cbic.201200223.
(292) Åslund, A.; Sigurdson, C. J.; Klingstedt, T.; Grathwohl, S.; Bolmont, T.; Dickstein, D. L.; Glimsdal, E.; Prokop, S.; Lindgren, M.; Konradsson, P.; Holtzman, D. M.; Hof, P. R.; Heppner, F. L.; Gandy, S.; Jucker, M.; Aguzzi, A.; Hammarström, P.; Nilsson, K. P. R. Novel Pentameric Thiophene Derivatives for in Vitro and in Vivo Optical Imaging of a Plethora of Protein Aggregates in Cerebral Amyloidoses. *ACS Chem. Biol.* **2009**, *4* (8), 673–684. DOI: 10.1021/cb900112v.
(293) Zhuang, Z.-P.; Kung, M.-P.; Hou, C.; Lee, C.-W.; Trojanowski, J. Q.; Lee, V. M.-Y.; Kung, H. F. Novel Probes for Imaging Amyloid: Iodinated Benzothiazole Derivatives. *J. Label. Compd. Radiopharm.* **2001**, *44* (S1), S29–S32. DOI: 10.1002/jlcr.2580440111.
(294) Cui, M.; Ono, M.; Kimura, H.; Liu, B.; Saji, H. Novel Quinoxaline Derivatives for in Vivo Imaging of β-Amyloid Plaques in the Brain. *Bioorg. Med. Chem. Lett.* **2011**, *21* (14), 4193–4196. DOI: 10.1016/j.bmcl.2011.05.079.
(295) Watanabe, H.; Ono, M.; Kimura, H.; Matsumura, K.; Yoshimura, M.; Iikuni, S.; Okamoto, Y.; Ihara, M.; Takahashi, R.; Saji, H. Novel Radioiodinated 1,3,4-Oxadiazole Derivatives with Improved in Vivo Properties for SPECT Imaging of β-Amyloid Plaques. *MedChemComm* **2014**, *5* (1), 82–85. DOI: 10.1039/c3md00189j.
(296) Maya, Y.; Ono, M.; Watanabe, H.; Haratake, M.; Saji, H.; Nakayama, M. Novel Radioiodinated Aurones as Probes for SPECT Imaging Of-Amyloid Plaques in the Brain. *Bioconjug. Chem.* **2009**, *20*, 95–101. DOI: 10.1021/bc8003292.
(297) Kung, H. F.; Lee, C. W.; Zhuang, Z. P.; Kung, M. P.; Hou, C.; Plössl, K. Novel Stilbenes as Probes for Amyloid Plaques. *J. Am. Chem. Soc.* **2001**, *123* (50), 12740–12741. DOI: 10.1021/ja0167147.
(298) Qu, W.; Kung, M.-P.; Hou, erine; Benedum, T. E.; Kung, H. F. Novel Styrylpyridines as Probes for SPECT Imaging of Amyloid Plaques. *J Med Chem* **2007**, *50* (9), 2157–2165. DOI: 10.1021/JM070025+.
(299) Kelényi, G. On the Histochemistry of Azo Group-Free Thiazole Dyes. *J. Histochem. Cytochem. Off. J. Histochem. Soc.* **1967**, *15* (3), 172–180. DOI: 10.1177/15.3.172.

(300) Wu, C.; Bowers, M. T.; Shea, J. E. On the Origin of the Stronger Binding of PIB over Thioflavin T to Protofibrils of the Alzheimer Amyloid-β Peptide: A Molecular Dynamics Study. *Biophys. J.* **2011**, *100* (5), 1316–1324. DOI: 10.1016/j.bpj.2011.01.058.
(301) Jia, J.; Song, J.; Dai, J.; Liu, B.; Cui, M. Optically Pure Diphenoxy Derivatives as More Flexible Probes for β-Amyloid Plaques. *ACS Chem. Neurosci.* **2016**, *7* (9), 1275–1282. DOI: 10.1021/acschemneuro.6b00155.
(302) Park, K.-S.; Seo, Y.; Kim, M. K.; Kim, K.; Kim, Y. K.; Choo, H.; Chong, Y. Organic & Biomolecular Chemistry A Curcumin-Based Molecular Probe for near-Infrared Fluorescence Imaging of Tau Fibrils in Alzheimer's Disease. *Org Biomol Chem* **2015**, *13*, 11194. DOI: 10.1039/c5ob01847a.
(303) Barrio, j. R.; Huang, S. C.; Cole, G. >: Satyamurthy, N. M.; Petric, A.; Phelps, M. E.; Small, G. W. PET Imaging of Tangles and Plaques in Alzheimer Disease with a Highly Hydrophobic Probe. *J. Label. Compd. Radiopharm.* **1999**, *42* (Supplement 1), 194–195.
(304) Lois, C.; Gonzalez, I.; Johnson, K. A.; Price, J. C. PET Imaging of Tau Protein Targets: A Methodology Perspective. *Brain Imaging Behav.* **2019**, *13* (2), 333–344. DOI: 10.1007/s11682-018-9847-7.
(305) Curtis, C.; Gamez, J. E.; Singh, U.; Sadowsky, C. H.; Villena, T.; Sabbagh, M. N.; Beach, T. G.; Duara, R.; Fleisher, A. S.; Frey, K. A.; Walker, Z.; Hunjan, A.; Holmes, C.; Escovar, Y. M.; Vera, C. X.; Agronin, M. E.; Ross, J.; Bozoki, A.; Akinola, M.; Shi, J.; Vandenberghe, R.; Ikonomovic, M. D.; Sherwin, P. F.; Grachev, I. D.; Farrar, G.; Smith, A. P. L.; Buckley, C. J.; McLain, R.; Salloway, S. Phase 3 Trial of Flutemetamol Labeled with Radioactive Fluorine 18 Imaging and Neuritic Plaque Density. *JAMA Neurol.* **2015**, *72* (3), 287–294. DOI: 10.1001/jamaneurol.2014.4144.
(306) Flaherty, D. P.; Kiyota, T.; Dong, Y.; Ikezu, T.; Vennerstrom, J. L. Phenolic Bis-Styrylbenzenes as β-Amyloid Binding Ligands and Free Radical Scavengers. *J. Med. Chem.* **2010**, *53* (22), 7992–7999. DOI: 10.1021/jm1006929.
(307) Zhang, J.; Konsmo, A.; Sandberg, A.; Wu, X.; Nyströ, S.; Obermü, U.; Wegenast-Braun, B. M.; Konradsson, P.; Lindgren, M.; Hammarströ, P. Phenolic Bis-Styrylbenzo[c]-1,2,5-Thiadiazoles as Probes for Fluorescence Microscopy Mapping of Aβ Plaque Heterogeneity. *J Med Chem* **2019**, *62*, 2038–2048. DOI: 10.1021/acs.jmedchem.8b01681.
(308) Matsumura, K.; Ono, M.; Hayashi, S.; Kimura, H.; Okamoto, Y.; Ihara, M.; Takahashi, R.; Mori, H.; Saji, H. Phenyldiazenyl Benzothiazole Derivatives as Probes for in Vivo Imaging of Neurofibrillary Tangles in Alzheimer's Disease Brains. *MedChemComm* **2011**, *2* (7), 596–600. DOI: 10.1039/c1md00034a.
(309) An, J.; Verwilst, P.; Aziz, H.; Shin, J.; Lim, S.; Kim, I.; Kim, Y. K.; Kim, J. S. Picomolar-Sensitive β-Amyloid Fibril Fluorophores by Tailoring the Hydrophobicity of Biannulated π-Elongated Dioxaborine-Dyes. *Bioact. Mater.* **2022**, *13*, 239–248. DOI: 10.1016/j.bioactmat.2021.10.047.
(310) Barnham, K. J.; Kenche, V. B.; Ciccotosto, G. D.; Smith, D. P.; Tew, D. J.; Liu, X.; Perez, K.; Cranston, G. A.; Johanssen, T. J.; Volitakis, I.; Bush, A. I.; Masters, C. L.; White, A. R.; Smith, J. P.; Cherny, R. A.; Cappai, R. Platinum-Based Inhibitors of Amyloid-β as Therapeutic Agents for Alzheimer's Disease. *Proc. Natl. Acad. Sci. U. S. A.* **2008**, *105* (19), 6813–6818. DOI: 10.1073/pnas.0800712105.
(311) Flaherty, D. P.; Walsh, S. M.; Kiyota, T.; Dong, Y.; Ikezu, T.; Vennerstrom, J. L. Polyfluorinated Bis-Styrylbenzene β-Amyloid Plaque Binding Ligands. *J. Med. Chem.* **2007**, *50* (20), 4986–4992. DOI: 10.1021/jm070085f.
(312) Nabuurs, R. J. A.; Kapoerchan, V. V.; Metaxas, A.; De Jongh, S.; De Backer, M.; Welling, M. M.; Jiskoot, W.; Windhorst, A. D.; Overkleeft, H. S.; Van Buchem, M. A.; Overhand, M.; Van Der Weerd, L. Polyfluorinated Bis-Styrylbenzenes as Amyloid-β Plaque Binding Ligands. *Bioorg. Med. Chem.* **2014**, *22* (8), 2469–2481. DOI: 10.1016/j.bmc.2014.02.054.
(313) Mason, N. S.; Mathis, C. A.; Klunk, W. E. Positron Emission Tomography Radioligands for *in Vivo* Imaging of A*β* Plaques. *J. Label. Compd. Radiopharm.* **2013**, *56* (3–4), 89–95. DOI: 10.1002/jlcr.2989.
(314) Ono, K.; Yoshiike, Y.; Takashima, A.; Hasegawa, K.; Naiki, H.; Yamada, M. Potent Anti-Amyloidogenic and Fibril-Destabilizing Effects of Polyphenols in Vitro: Implications for the Prevention and Therapeutics of Alzheimer's Disease. *J. Neurochem.* **2003**, *87* (1), 172–181. DOI: 10.1046/j.1471-4159.2003.01976.x.

(315) Hostetler, E. D.; Walji, A. M.; Zeng, Z.; Miller, P.; Bennacef, I.; Salinas, C.; Connolly, B.; Gantert, L.; Haley, H.; Holahan, M.; Purcell, M.; Riffel, K.; Lohith, T. G.; Coleman, P.; Soriano, A.; Ogawa, A.; Xu, S.; Zhang, X.; Joshi, E.; Della Rocca, J.; Hesk, D.; Schenk, D. J.; Evelhoch, J. L. Preclinical Characterization of 18F-MK-6240, a Promising PET Tracer for in Vivo Quantification of Human Neurofibrillary Tangles. *J. Nucl. Med.* **2016**, *57* (10), 1599–1606. DOI: 10.2967/jnumed.115.171678.

(316) Declercq, L.; Rombouts, F.; Koole, M.; Fierens, K.; Mariën, J.; Langlois, X.; Andrés, J. I.; Schmidt, M.; MacDonald, G.; Moechars, D.; Vanduffel, W.; Tousseyn, T.; Vandenberghe, R.; Van Laere, K.; Verbruggen, A.; Bormans, G. Preclinical Evaluation of 18F-JNJ64349311, a Novel PET Tracer for Tau Imaging. *J. Nucl. Med.* **2017**, *58* (6), 975–981. DOI: 10.2967/jnumed.116.185199.

(317) Kumar, J. S. D.; Molotkov, A.; Kim, J.; Carberry, P.; Idumonyi, S.; Castrillon, J.; Duff, K.; Shneider, N. A.; Mintz, A. Preclinical Evaluation of a Microtubule PET Ligand [11C]MPC-6827 in Tau and Amyotrophic Lateral Sclerosis Animal Models. *Pharmacol. Rep.* **2022**, *74* (3), 539–544. DOI: 10.1007/s43440-022-00359-y.

(318) Choi, S. R.; Golding, G.; Zhuang, Z.; Zhang, W.; Lim, N.; Hefti, F.; Benedum, T. E.; Kilbourn, M. R.; Skovronskyhank, D.; Kung, H. F. Preclinical Properties of 18F-AV-45: A PET Agent for Aβ Plaques in the Brain. *J. Nucl. Med.* **2009**, *50* (11), 1887–1894. DOI: 10.2967/jnumed.109.065284.

(319) Yang, Y.; Zhang, X.; Cui, M.; Zhang, J.; Guo, Z.; Li, Y.; Zhang, X.; Dai, J.; Liu, B. Preliminary Characterization and in Vivo Studies of Structurally Identical 18 F-and 125 I-Labeled Benzyloxybenzenes for PET/SPECT Imaging of β-Amyloid Plaques. *Sci. Rep.* **2015**, *5* (1), 12084. DOI: 10.1038/srep12084.

(320) Yang, Y.; Fu, H.; Cui, M.; Peng, C.; Liang, Z.; Dai, J.; Zhang, Z.; Lin, C.; Liu, B. Preliminary Evaluation of Fluoro-Pegylated Benzyloxybenzenes for Quantification of β-Amyloid Plaques by Positron Emission Tomography. *Eur. J. Med. Chem.* **2015**, *104*, 86–96. DOI: 10.1016/j.ejmech.2015.09.028.

(321) Dezutter, N. A.; De Groot, T. J.; Busson, R. H.; Janssen, G. A.; Verbruggen, A. M. Preparation of 99mTc-N2S2 Conjugates of Chrysamine G, Potential Probes for the Beta-Amyloid Protein of Alzheimer's Disease. *J. Label. Compd. Radiopharm.* **1999**, *42* (4), 309–324. DOI: 10.1002/(SICI)1099-1344(199904)42:4<309::AID-JLCR192>3.0.CO;2-O.

(322) Björk, L.; Bäck, M.; Lantz, L.; Ghetti, B.; Vidal, R.; Klingstedt, T.; Nilsson, K. P. R. Proteophenes – Amino Acid Functionalized Thiophene-Based Fluorescent Ligands for Visualization of Protein Deposits in Tissue Sections with Alzheimer's Disease Pathology. *Chem. – Eur. J.* **2022**, *28* (62), e202201557. DOI: 10.1002/chem.202201557.

(323) Ono, M.; Hayashi, S.; Kimura, H.; Kawashima, H.; Nakayama, M.; Saji, H. Push–Pull Benzothiazole Derivatives as Probes for Detecting β-Amyloid Plaques in Alzheimer's Brains. *Bioorg. Med. Chem.* **2009**, *17* (19), 7002–7007. DOI: 10.1016/j.bmc.2009.08.032.

(324) Choi, J. W.; Ju, Y. H.; Ju, Y. H.; Choi, Y.; Choi, Y.; Hyeon, S. J.; Gadhe, C. G.; Park, J. H.; Kim, M. S.; Baek, S.; Kim, Y.; Park, K. D.; Park, K. D.; Pae, A. N.; Pae, A. N.; Ryu, H.; Ryu, H.; Lee, C. J.; Lee, C. J.; Cho, B. R.; Cho, B. R.; Cho, B. R. PyrPeg, a Blood-Brain-Barrier-Penetrating Two-Photon Imaging Probe, Selectively Detects Neuritic Plaques, Not Tau Aggregates. *ACS Chem. Neurosci.* **2020**, *11* (12), 1801–1810. DOI: 10.1021/acschemneuro.0c00211.

(325) Qu, W.; Kung, M. P.; Hou, C.; Oya, S.; Kung, H. F. Quick Assembly of 1,4-Diphenyltriazoles as Probes Targeting β-Amyloid Aggregates in Alzheimer's Disease. *J. Med. Chem.* **2007**, *50* (14), 3380–3387. DOI: 10.1021/jm070467l.

(326) Okamura, N.; Suemoto, T.; Furumoto, S.; Suzuki, M.; Shimadzu, H.; Akatsu, H.; Yamamoto, T.; Fujiwara, H.; Nemoto, M.; Maruyama, M.; Arai, H.; Yanai, K.; Sawada, T.; Kudo, Y. Quinoline and Benzimidazole Derivatives: Candidate Probes for in Vivo Imaging of Tau Pathology in Alzheimer's Disease. *J. Neurosci.* **2005**, *25* (47), 10857–10862. DOI: 10.1523/JNEUROSCI.1738-05.2005.

(327) Qu, W.; Kung, M. P.; Hou, C.; Jin, L. W.; Kung, H. F. Radioiodinated Aza-Diphenylacetylenes as Potential SPECT Imaging Agents for β-Amyloid Plaque Detection. *Bioorg. Med. Chem. Lett.* **2007**, *17* (13), 3581–3584. DOI: 10.1016/j.bmcl.2007.04.062.

(328) Cui, M.; Ono, M.; Kimura, H.; Kawashima, H.; Liu, B. L.; Saji, H. Radioiodinated Benzimidazole Derivatives as Single Photon Emission Computed Tomography Probes for Imaging of α-Amyloid Plaques in Alzheimer's Disease. *Nucl. Med. Biol.* **2011**, *38* (3), 313–320. DOI: 10.1016/j.nucmedbio.2010.09.012.
(329) Yang, Y.; Cui, M.; Zhang, X.; Dai, J.; Zhang, Z.; Lin, C.; Guo, Y.; Liu, B. Radioiodinated Benzyloxybenzene Derivatives: A Class of Flexible Ligands Target to β-Amyloid Plaques in Alzheimer's Brains. *J. Med. Chem.* **2014**, *57* (14), 6030–6042. DOI: 10.1021/jm5004396.
(330) Ono, M.; Yoshida, N.; Ishibashi, K.; Haratake, M.; Arano, Y.; Mori, H.; Nakayama, M. Radioiodinated Flavones for in Vivo Imaging of β-Amyloid Plaques in the Brain. *J. Med. Chem.* **2005**, *48* (23), 7253–7260. DOI: 10.1021/jm050635e.
(331) Kung, M. P.; Hou, C.; Zhuang, Z. P.; Skovronsky, D. M.; Zhang, B.; Gur, T. L.; Trojanowski, J. Q.; Lee, V. M. Y.; Kung, H. F. Radioiodinated Styrylbenzene Derivatives as Potential SPECT Imaging Agents for Amyloid Plaque Detection in Alzheimer's Disease. *J. Mol. Neurosci.* **2002**, *19* (1–2), 7–10. DOI: 10.1007/s12031-002-0003-9.
(332) Yang, Y.; Cui, M. Radiolabeled Bioactive Benzoheterocycles for Imaging β-Amyloid Plaques in Alzheimer's Disease. *Eur. J. Med. Chem.* **2014**, *87*, 703–721. DOI: 10.1016/j.ejmech.2014.10.012.
(333) Cai, L.; Innis, R.; Pike, V. Radioligand Development for PET Imaging of β-Amyloid (Aβ)-Current Status. *Curr. Med. Chem.* **2006**, *14* (1), 19–52. DOI: 10.2174/092986707779313471.
(334) Cao, Y.; Xu, S.; Liu, J.; Zhao, S.; Yan, J. Rational Construction and Evaluation of a Dual-Functional near-Infrared Fluorescent Probe for the Imaging of Amyloid-β and Mitochondrial Viscosity. *Spectrochim. Acta. A. Mol. Biomol. Spectrosc.* **2024**, *306*, 123564. DOI: 10.1016/j.saa.2023.123564.
(335) Soloperto, A.; Quaglio, D.; Baiocco, P.; Romeo, I.; Mori, M.; Ardini, M.; Presutti, C.; Sannino, I.; Ghirga, S.; Iazzetti, A.; Ippoliti, R.; Ruocco, G.; Botta, B.; Ghirga, F.; Di Angelantonio, S.; Boffi, A. Rational Design and Synthesis of a Novel BODIPY-Based Probe for Selective Imaging of Tau Tangles in Human iPSC-Derived Cortical Neurons. *Sci. Rep.* **2022**, *12* (1), 5257. DOI: 10.1038/s41598-022-09016-z.
(336) Sutharsan, J.; Dakanali, M.; Capule, C.; Haidekker, M.; Yang, J.; Theodorakis, E. Rational Design of Amyloid Binding Agents Based on the Molecular Rotor Motif. *ChemMedChem* **2010**, *5* (1), 56–60. DOI: 10.1002/cmdc.200900440.
(337) Yue, N.; Fu, H.; Chen, Y.; Gao, X.; Dai, J.; Cui, M. Rational Design of Molecular Rotor-Based Fluorescent Probes with Bi-Aromatic Rings for Efficient in Vivo Detection of Amyloid-β Plaques in Alzheimer's Disease. *Eur. J. Med. Chem.* **2022**, *243*, 114715. DOI: 10.1016/j.ejmech.2022.114715.
(338) Fu, W.; Yan, C.; Guo, Z.; Zhang, J.; Zhang, H.; Tian, H.; Zhu, W. H. Rational Design of Near-Infrared Aggregation-Induced-Emission-Active Probes: In Situ Mapping of Amyloid-β Plaques with Ultrasensitivity and High-Fidelity. *J. Am. Chem. Soc.* **2019**, *141* (7), 3171–3177. DOI: 10.1021/jacs.8b12820.
(339) Liu, X.-Y.; Wang, X.-J.; Shi, L.; Liu, Y.-H.; Wang, L.; Li, K.; Bu, Q.; Cen, X.-B.; Yu, X.-Q. Rational Design of Quinoxalinone-Based Red-Emitting Probes for High-Affinity and Long-Term Visualizing Amyloid-β In Vivo. *Anal. Chem.* **2022**, *94* (21), 7665–7673. DOI: 10.1021/acs.analchem.2c01046.
(340) Wang, B.; Shi, J.; Guo, N.; Shao, L.; Zhai, W.; Jiang, L.; Zhao, F.; Wang, J.; Wang, J.; Du, L.; Pang, X.; Yan, L. Rational Design Synthesis and Evaluation of a Novel Near-Infrared Fluorescent Probe for Selective Imaging of Amyloid-β Aggregates in Alzheimer's Disease. *Anal. Chim. Acta* **2023**, *1281*, 341900. DOI: 10.1016/j.aca.2023.341900.
(341) Lee, J.-H.; Lee, I.-H.; Choe, Y.-J.; Kang, S.; Kim, H. Y.; Gai, W.-P.; Hahn, J.-S.; Paik, S. R. Real-Time Analysis of Amyloid Fibril Formation of α-Synuclein Using a Fibrillation-State-Specific Fluorescent Probe of JC-1. *Biochem. J.* **2009**, *418* (2), 311–323. DOI: 10.1042/BJ20081572.
(342) Liu, Y.; Zhuang, D.; Wang, J.; Huang, H.; Li, R.; Wu, C.; Deng, Y.; Hu, G.; Guo, B. Recent Advances in Small Molecular Near-Infrared Fluorescence Probes for a Targeted Diagnosis of the Alzheimer Disease. *Analyst* **2022**, *147* (21), 4701–4723. DOI: 10.1039/D2AN01327D.
(343) Chen, K.; Cui, M. Recent Progress in the Development of Metal Complexes as β-Amyloid Imaging Probes in the Brain. *MedChemComm* **2017**, *8* (7), 1393–1407. DOI: 10.1039/c7md00064b.

(344) Gabr, M. T.; Pigge, F. C. Rhenium Complexes of Bis(Benzothiazole)-Based Tetraarylethylenes as Selective Luminescent Probes for Amyloid Fibrils. *Chem. – Eur. J.* **2018**, *24* (45), 11729–11737. DOI: 10.1002/chem.201801801.
(345) Ono, M.; Hayashi, S.; Matsumura, K.; Kimura, H.; Okamoto, Y.; Ihara, M.; Takahashi, R.; Mori, H.; Saji, H. Rhodanine and Thiohydantoin Derivatives for Detecting Tau Pathology in Alzheimer's Brains. *ACS Chem. Neurosci.* **2011**, *2* (5), 269–275. DOI: 10.1021/cn200002t.
(346) Bulic, B.; Pickhardt, M.; Khlistunova, I.; Biernat, J.; Mandelkow, E.-M.; Mandelkow, E.; Waldmann, H. Rhodanine-Based Tau Aggregation Inhibitors in Cell Models of Tauopathy. *Angew. Chem. Int. Ed.* **2007**, *46* (48), 9215–9219. DOI: 10.1002/anie.200704051.
(347) Ruiz-Arias, Á.; Jurado, R.; Fueyo-González, F.; Herranz, R.; Gálvez, N.; González-Vera, J. A.; Orte, A. Selecting FRET Pairs for Visualizing Amyloid Aggregation. *Results Chem.* **2022**, *4*, 100275. DOI: 10.1016/j.rechem.2021.100275.
(348) Rojo, L. E.; Alzate-Morales, J.; Saavedra, I. N.; Davies, P.; MacCioni, R. B. Selective Interaction of Lansoprazole and Astemizole with Tau Polymers: Potential New Clinical Use in Diagnosis of Alzheimer's Disease. *J. Alzheimers Dis.* **2010**, *19* (2), 573–589. DOI: 10.3233/JAD-2010-1262.
(349) Babu, E.; Muthu Mareeswaran, P.; Sathish, V.; Singaravadivel, S.; Rajagopal, S. Sensing and Inhibition of Amyloid-β Based on the Simple Luminescent Aptamer-Ruthenium Complex System. *Talanta* **2015**, *134*, 348–353. DOI: 10.1016/j.talanta.2014.11.020.
(350) Verwilst, P.; Kim, H. S.; Kim, S.; Kang, C.; Kim, J. S. Shedding Light on Tau Protein Aggregation: The Progress in Developing Highly Selective Fluorophores. *Chem. Soc. Rev.* **2018**, *47* (7), 2249–2265. DOI: 10.1039/c7cs00706j.
(351) Sharma, A. K.; Kim, J.; Prior, J. T.; Hawco, N. J.; Rath, N. P.; Kim, J.; Mirica, L. M. Small Bifunctional Chelators That Do Not Disaggregate Amyloid β Fibrils Exhibit Reduced Cellular Toxicity. *Inorg. Chem.* **2014**, *53* (21), 11367–11376. DOI: 10.1021/ic500926c.
(352) Manook, A.; Yousefi, B. H.; Willuweit, A.; Platzer, S.; Reder, S.; Voss, A.; Huisman, M.; Settles, M.; Neff, F.; Velden, J.; Schoor, M.; von der Kammer, H.; Wester, H. J.; Schwaiger, M.; Henriksen, G.; Drzezga, A. Small-Animal PET Imaging of Amyloid-Beta Plaques with [11c]PiB and Its Multi-Modal Validation in an APP/PS1 Mouse Model of Alzheimer's Disease. *PLoS ONE* **2012**, *7* (3), e31310. DOI: 10.1371/journal.pone.0031310.
(353) Zhou, K.; Bai, H.; Feng, L.; Dai, J.; Cui, M. Smart D-π-A Type Near-Infrared Aβ Probes: Effects of a Marked π Bridge on Optical and Biological Properties. *Anal. Chem.* **2017**, *89* (17), 9432–9437. DOI: 10.1021/acs.analchem.7b02246.
(354) Cui, M.; Ono, M.; Watanabe, H.; Kimura, H.; Liu, B.; Saji, H. Smart Near-Infrared Fluorescence Probes with Donor-Acceptor Structure for in Vivo Detection of β-Amyloid Deposits. *J. Am. Chem. Soc.* **2014**, *136* (9), 3388–3394. DOI: 10.1021/ja4052922.
(355) Cao, K. J.; Elbel, K. M.; Cifelli, J. L.; Cirera, J.; Sigurdson, C. J.; Paesani, F.; Theodorakis, E. A.; Yang, J. Solvation-Guided Design of Fluorescent Probes for Discrimination of Amyloids. *Sci. Rep.* **2018**, *8*, 6950. DOI: 10.1038/s41598-018-25131-2.
(356) Volkova, K. D.; Kovalska, V. B.; Balanda, A. O.; Losytskyy, M. Y.; Golub, A. G.; Vermeij, R. J.; Subramaniam, V.; Tolmachev, O. I.; Yarmoluk, S. M. Specific Fluorescent Detection of Fibrillar α-Synuclein Using Mono- and Trimethine Cyanine Dyes. *Bioorg. Med. Chem.* **2008**, *16* (3), 1452–1459. DOI: 10.1016/j.bmc.2007.10.051.
(357) Kung, H. F.; Kung, M.-P.; Zhuang, Z.-P. Stilbene Derivatives and Their Use for Binding and Imaging Amyloid Plaques. US7807135B2, 2010.
(358) Su, D.; Diao, W.; Li, J.; Pan, L.; Zhang, X.; Wu, X.; Mao, W. Strategic Design of Amyloid-β Species Fluorescent Probes for Alzheimer's Disease. *ACS Chem. Neurosci.* **2022**, *13* (5), 540–551. DOI: 10.1021/acschemneuro.1c00810.

(359) Tago, T.; Furumoto, S.; Okamura, N.; Harada, R.; Adachi, H.; Ishikawa, Y.; Yanai, K.; Iwata, R.; Kudo, Y. Structure-Activity Relationship of 2-Arylquinolines as PET Imaging Tracers for Tau Pathology in Alzheimer Disease. *J. Nucl. Med.* **2016**, *57* (4), 608–614. DOI: 10.2967/jnumed.115.166652.
(360) Ono, M.; Hori, M.; Haratake, M.; Tomiyama, T.; Mori, H.; Nakayama, M. Structure-Activity Relationship of Chalcones and Related Derivatives as Ligands for Detecting of β-Amyloid Plaques in the Brain. *Bioorg. Med. Chem.* **2007**, *15* (19), 6388–6396. DOI: 10.1016/j.bmc.2007.06.055.
(361) Zhuang, Z. P.; Kung, M. P.; Wilson, A.; Lee, C. W.; Plössl, K.; Hou, C.; Holtzman, D. M.; Kung, H. F. Structure-Activity Relationship of Imidazo[1,2-a]Pyridines as Ligands for Detecting β-Amyloid Plaques in the Brain. *J. Med. Chem.* **2003**, *46* (2), 237–243. DOI: 10.1021/jm020351j.
(362) Yoshimura, M.; Ono, M.; Matsumura, K.; Watanabe, H.; Kimura, H.; Cui, M.; Nakamoto, Y.; Togashi, K.; Okamoto, Y.; Ihara, M.; Takahashi, R.; Saji, H. Structure-Activity Relationships and in Vivo Evaluation of Quinoxaline Derivatives for PET Imaging of β-Amyloid Plaques. *ACS Med. Chem. Lett.* **2013**, *4* (7), 596–600. DOI: 10.1021/ml4000707.
(363) Ono, M.; Doi, Y.; Watanabe, H.; Ihara, M.; Ozaki, A.; Saji, H. Structure-Activity Relationships of Radioiodinated Diphenyl Derivatives with Different Conjugated Double Bonds as Ligands for α-Synuclein Aggregates. *RSC Adv.* **2016**, *6* (50), 44305–44312. DOI: 10.1039/c6ra02710e.
(364) Chia, S.; Faidon Brotzakis, Z.; Horne, R. I.; Possenti, A.; Mannini, B.; Cataldi, R.; Nowinska, M.; Staats, R.; Linse, S.; Knowles, T. P. J.; Habchi, J.; Vendruscolo, M. Structure-Based Discovery of Small-Molecule Inhibitors of the Autocatalytic Proliferation of α-Synuclein Aggregates. *Mol. Pharm.* **2023**, *20* (1), 183–193. DOI: 10.1021/acs.molpharmaceut.2c00548.
(365) Zhou, K.; Li, Y.; Peng, Y.; Cui, X.; Dai, J.; Cui, M. Structure-Property Relationships of Polyethylene Glycol Modified Fluorophore as Near-Infrared Aβ Imaging Probes. *Anal. Chem.* **2018**, *90* (14), 8576–8582. DOI: 10.1021/acs.analchem.8b01712.
(366) Zeng, Q.; Liu, S.; Cui, M. Structure–Activity Relationships of Cyano-Substituted Indole Derivatives as Ligands for α-Synuclein Aggregates. *ACS Med. Chem. Lett.* **2023**, *14* (10), 1467–1471. DOI: 10.1021/acsmedchemlett.3c00384.
(367) Nakagawa, K.; Watanabe, H.; Kaide, S.; Ono, M. Structure–Activity Relationships of Styrylquinoline and Styrylquinoxaline Derivatives as α-Synuclein Imaging Probes. *ACS Med. Chem. Lett.* **2022**, *13* (10), 1598–1605. DOI: 10.1021/acsmedchemlett.2c00279.
(368) Li, Q.; Min, J.; Ahn, Y.-H.; Namm, J.; Kim, E. M.; Lui, R.; Kim, H. Y.; Ji, Y.; Wu, H.; Wisniewski, T.; Chang, Y.-T. Styryl-Based Compounds as Potential in Vivo Imaging Agents for β-Amyloid Plaques. *ChemBioChem* **2007**, *8* (14), 1679–1687. DOI: 10.1002/cbic.200700154.
(369) Cabrera, P.; Jara-Guajardo, P.; Oyarzún, M. P.; Parra-Muñoz, N.; Campos, A.; Soler, M.; Álvarez, A.; Morales-Zavala, F.; Araya, E.; Minniti, A. N.; Aldunate, R.; Kogan, M. J. Surface Enhanced Fluorescence Effect Improves the in Vivo Detection of Amyloid Aggregates. *Nanomedicine Nanotechnol. Biol. Med.* **2022**, *44*, 102569. DOI: 10.1016/j.nano.2022.102569.
(370) Wang, Y.; Mathis, C. A.; Huang, G. F.; Holt, D. P.; Debnath, M. L.; Klunk, W. E. Synthesis and 11c-Labelling of (E,E)-1-(3′,4′-Dihydroxystyryl)-4-(3′-Methoxy-4 ′-Hydroxystyryl) Benzene for PET Imaging of Amyloid Deposits. *J. Label. Compd. Radiopharm.* **2002**, *45* (8), 647–664. DOI: 10.1002/jlcr.585.
(371) Zhen, W.; Han, H.; Anguiano, M.; Lemere, C. A.; Cho, C. G.; Lansbury, P. T. Synthesis and Amyloid Binding Properties of Rhenium Complexes: Preliminary Progress toward a Reagent for SPECT Imaging of Alzheimer's Disease Brain. *J. Med. Chem.* **1999**, *42* (15), 2805–2815. DOI: 10.1021/jm990103w.
(372) Ono, M.; Haratake, M.; Nakayama, M.; Kaneko, Y.; Kawabata, K.; Mori, H.; Kung, M. P.; Kung, H. F. Synthesis and Biological Evaluation of (E)-3-Styrylpyridine Derivatives as Amyloid Imaging Agents for Alzheimer's Disease. *Nucl. Med. Biol.* **2005**, *32* (4), 329–335. DOI: 10.1016/j.nucmedbio.2005.01.006.
(373) Fu, H.; Yu, L.; Cui, M.; Zhang, J.; Zhang, X.; Li, Z.; Wang, X.; Jia, J.; Yang, Y.; Yu, P.; Jia, H.; Liu, B. Synthesis and Biological Evaluation of 18F-Labled 2-Phenylindole Derivatives as PET Imaging Probes for β-Amyloid Plaques. *Bioorg. Med. Chem.* **2013**, *21* (13), 3708–3714. DOI: 10.1016/j.bmc.2013.04.028.

(374) Watanabe, H.; Ono, M.; Iikuni, S.; Kimura, H.; Okamoto, Y.; Ihara, M.; Saji, H. Synthesis and Biological Evaluation of 123I-Labeled Pyridyl Benzoxazole Derivatives: Novel β-Amyloid Imaging Probes for Single-Photon Emission Computed Tomography. *RSC Adv.* **2015**, *5*, 1009–1015. DOI: 10.1039/c4ra10742j.
(375) Gan, C.; Hu, J.; Nan, D. D.; Wang, S.; Li, H. Synthesis and Biological Evaluation of Curcumin Analogs as β-Amyloid Imaging Agents. *Future Med. Chem.* **2017**, *9* (14), 1587–1596. DOI: 10.4155/fmc-2017-0079.
(376) Cui, M.; Ono, M.; Kimura, H.; Liu, B. L.; Saji, H. Synthesis and Biological Evaluation of Indole-Chalcone Derivatives as β-Amyloid Imaging Probe. *Bioorg. Med. Chem. Lett.* **2011**, *21* (3), 980–982. DOI: 10.1016/j.bmcl.2010.12.045.
(377) Watanabe, H.; Ono, M.; Kimura, H.; Matsumura, K.; Yoshimura, M.; Okamoto, Y.; Ihara, M.; Takahashi, R.; Saji, H. Synthesis and Biological Evaluation of Novel Oxindole Derivatives for Imaging Neurofibrillary Tangles in Alzheimer's Disease. *Bioorg. Med. Chem. Lett.* **2012**, *22* (17), 5700–5703. DOI: 10.1016/j.bmcl.2012.06.086.
(378) Watanabe, H.; Ariyoshi, T.; Ozaki, A.; Ihara, M.; Ono, M.; Saji, H. Synthesis and Biological Evaluation of Novel Radioiodinated Benzimidazole Derivatives for Imaging α-Synuclein Aggregates. *Bioorg. Med. Chem.* **2017**, *25* (24), 6398–6403. DOI: 10.1016/j.bmc.2017.10.010.
(379) Chen, C. J.; Bando, K.; Ashino, H.; Taguchi, K.; Shiraishi, H.; Fujimoto, O.; Kitamura, C.; Matsushima, S.; Fujinaga, M.; Zhang, M. R.; Kasahara, H.; Minamizawa, T.; Jiang, C.; Ono, M.; Higuchi, M.; Suhara, T.; Yamada, K.; Ji, B. Synthesis and Biological Evaluation of Novel Radioiodinated Imidazopyridine Derivatives for Amyloid-β Imaging in Alzheimer's Disease. *Bioorg. Med. Chem.* **2014**, *22* (15), 4189–4197. DOI: 10.1016/j.bmc.2014.05.043.
(380) Matsumura, K.; Ono, M.; Yoshimura, M.; Kimura, H.; Watanabe, H.; Okamoto, Y.; Ihara, M.; Takahashi, R.; Saji, H. Synthesis and Biological Evaluation of Novel Styryl Benzimidazole Derivatives as Probes for Imaging of Neurofibrillary Tangles in Alzheimer's Disease. *Bioorg. Med. Chem.* **2013**, *21* (11), 3356–3362. DOI: 10.1016/j.bmc.2013.02.054.
(381) Watanabe, H.; Ono, M.; Ikeoka, R.; Haratake, M.; Saji, H.; Nakayama, M. Synthesis and Biological Evaluation of Radioiodinated 2,5-Diphenyl-1,3,4-Oxadiazoles for Detecting β-Amyloid Plaques in the Brain. *Bioorg. Med. Chem.* **2009**, *17* (17), 6402–6406. DOI: 10.1016/j.bmc.2009.07.020.
(382) Fuchigami, T.; Kobashi, N.; Haratake, M.; Kawasaki, M.; Nakayama, M. Synthesis and Biological Evaluation of Radioiodinated Quinacrine-Based Derivatives for SPECT Imaging of Aβ Plaques. *Eur. J. Med. Chem.* **2013**, *60*, 469–478. DOI: 10.1016/j.ejmech.2012.12.020.
(383) Lengyel-Zhand, Z.; Ferrie, J. J.; Janssen, B.; Hsieh, C. J.; Graham, T.; Xu, K. Y.; Haney, C. M.; Lee, V. M. Y.; Trojanowski, J. Q.; Petersson, E. J.; Mach, R. H. Synthesis and Characterization of High Affinity Fluorogenic α-Synuclein Probes. *Chem. Commun.* **2020**, *56* (24), 3567–3570. DOI: 10.1039/c9cc09849f.
(384) Watanabe, H.; Ono, M.; Haratake, M.; Kobashi, N.; Saji, H.; Nakayama, M. Synthesis and Characterization of Novel Phenylindoles as Potential Probes for Imaging of β-Amyloid Plaques in the Brain. *Bioorg. Med. Chem.* **2010**, *18* (13), 4740–4746. DOI: 10.1016/j.bmc.2010.05.013.
(385) Ono, M.; Maya, Y.; Haratake, M.; Nakayama, M. Synthesis and Characterization of Styrylchromone Derivatives as β-Amyloid Imaging Agents. *Bioorg. Med. Chem.* **2007**, *15* (1), 444–450. DOI: 10.1016/j.bmc.2006.09.044.
(386) Lee, I.; Yang, J.; Lee, J. H.; Choe, Y. S. Synthesis and Evaluation of 1-(4-[18F]Fluoroethyl)-7-(4′- Methyl)Curcumin with Improved Brain Permeability for β-Amyloid Plaque Imaging. *Bioorg. Med. Chem. Lett.* **2011**, *21* (19), 5765–5769. DOI: 10.1016/j.bmcl.2011.08.003.
(387) Lv, P.; Xia, C. L.; Wang, N.; Liu, Z. Q.; Huang, Z. S.; Huang, S. L. Synthesis and Evaluation of 1,2,3,4-Tetrahydro-1-Acridone Analogues as Potential Dual Inhibitors for Amyloid-Beta and Tau Aggregation. *Bioorg. Med. Chem.* **2018**, *26* (16), 4693–4705. DOI: 10.1016/j.bmc.2018.08.007.
(388) Wang, Y.; Klunk, W. E.; Huang, G. F.; Debnath, M. L.; Holt, D. P.; Mathis, C. A. Synthesis and Evaluation of 2-(3′-Iodo-4′-Aminophenyl)-6-Hydroxybenzothiazole for in Vivo Quantitation of Amyloid Deposits in Alzheimer's Disease. *J. Mol. Neurosci.* **2002**, *19* (1–2), 11–16. DOI: 10.1007/s12031-002-0004-8.

(389) Swahn, B. M.; Wensbo, D.; Sandell, J.; Sohn, D.; Slivo, C.; Pyring, D.; Malmström, J.; Arzel, E.; Vallin, M.; Bergh, M.; Jeppsson, F.; Johnson, A. E.; Juréus, A.; Neelissen, J.; Svensson, S. Synthesis and Evaluation of 2-Pyridylbenzothiazole, 2-Pyridylbenzoxazole and 2-Pyridylbenzofuran Derivatives as 11C-PET Imaging Agents for β-Amyloid Plaques. *Bioorg. Med. Chem. Lett.* **2010**, *20* (6), 1976–1980. DOI: 10.1016/j.bmcl.2010.01.105.

(390) Harrison, S. T.; Mulhearn, J.; Wolkenberg, S. E.; Miller, P. J.; O'Malley, S. S.; Zeng, Z.; Williams, D. L.; Hostetler, E. D.; Sanabria-Bohórquez, S.; Gammage, L.; Fan, H.; Sur, C.; Culberson, J. C.; Hargreaves, R. J.; Cook, J. J.; Hartman, G. D.; Barrow, J. C. Synthesis and Evaluation of 5-Fluoro-2-Aryloxazolo[5,4- b]Pyridines as β-Amyloid PET Ligands and Identification of MK-3328. *ACS Med. Chem. Lett.* **2011**, *2* (7), 498–502. DOI: 10.1021/ml200018n.

(391) Mathis, C. A.; Wang, Y.; Holt, D. P.; Huang, G. F.; Debnath, M. L.; Klunk, W. E. Synthesis and Evaluation of 11C-Labeled 6-Substituted 2-Arylbenzothiazoles as Amyloid Imaging Agents. *J. Med. Chem.* **2003**, *46*, 2740. DOI: 10.1021/jm030026b.

(392) Yousefi, B. H.; Manook, A.; Drzezga, A.; Reutern, B. V.; Schwaiger, M.; Wester, H. J.; Henriksen, G. Synthesis and Evaluation of 11C-Labeled Imidazo[2,1-b] Benzothiazoles (IBTs) as PET Tracers for Imaging β-Amyloid Plaques in Alzheimer's Disease. *J. Med. Chem.* **2011**, *54* (4), 949–956. DOI: 10.1021/jm101129a.

(393) Neumaier, B.; Deisenhofer, S.; Sommer, C.; Solbach, C.; Reske, S. N.; Mottaghy, F. Synthesis and Evaluation of 18F-Fluoroethylated Benzothiazole Derivatives for in Vivo Imaging of Amyloid Plaques in Alzheimer's Disease. *Appl. Radiat. Isot.* **2010**, *68* (6), 1066–1072. DOI: 10.1016/j.apradiso.2009.12.044.

(394) Serdons, K.; Terwinghe, C.; Vermaelen, P.; Van Laere, K.; Kung, H.; Mortelmans, L.; Bormans, G.; Verbruggen, A. Synthesis and Evaluation of 18F-Labeled 2-Phenylbenzothiazoles as Positron Emission Tomography Imaging Agents for Amyloid Plaques in Alzheimer's Disease. *J. Med. Chem.* **2009**, *52* (5), 1428–1437. DOI: 10.1021/jm8013376.

(395) Kaide, S.; Watanabe, H.; Iikuni, S.; Hasegawa, M.; Ono, M. Synthesis and Evaluation of 18F-Labeled Chalcone Analogue for Detection of α-Synuclein Aggregates in the Brain Using the Mouse Model. *ACS Chem. Neurosci.* **2022**, *13* (20), 2982–2990. DOI: 10.1021/acschemneuro.2c00473.

(396) Rokka, J.; Snellman, A.; Zona, C.; La Ferla, B.; Nicotra, F.; Salmona, M.; Forloni, G.; Haaparanta-Solin, M.; Rinne, J. O.; Solin, O. Synthesis and Evaluation of a 18F-Curcumin Derivate for β-Amyloid Plaque Imaging. *Bioorg. Med. Chem.* **2014**, *22* (9), 2753–2762. DOI: 10.1016/j.bmc.2014.03.010.

(397) Cui, M.; Ono, M.; Kimura, H.; Liu, B.; Saji, H. Synthesis and Evaluation of Benzofuran-2-Yl(Phenyl)Methanone Derivatives as Ligands for β-Amyloid Plaques. *Bioorg. Med. Chem.* **2011**, *19* (13), 4148–4153. DOI: 10.1016/j.bmc.2011.04.049.

(398) Chang, Y. S.; Jeong, J. M.; Lee, Y. S.; Kim, H. W.; Ganesha, R. B.; Kim, Y. J.; Lee, D. S.; Chung, J. K.; Lee, M. C. Synthesis and Evaluation of Benzothiophene Derivatives as Ligands for Imaging β-Amyloid Plaques in Alzheimer's Disease. *Nucl. Med. Biol.* **2006**, *33* (6), 811–820. DOI: 10.1016/j.nucmedbio.2006.06.006.

(399) Li, L.; Xiang, F.; Yao, L.; Zhang, C.; Jia, X.; Chen, A.; Liu, Y. Synthesis and Evaluation of Curcumin-Based near-Infrared Fluorescent Probes for Detection of Amyloid β Peptide in Alzheimer Mouse Models. *Bioorg. Med. Chem.* **2023**, *92*, 117410. DOI: 10.1016/j.bmc.2023.117410.

(400) Fuchigami, T.; Yamashita, Y.; Haratake, M.; Ono, M.; Yoshida, S.; Nakayama, M. Synthesis and Evaluation of Ethyleneoxylated and Allyloxylated Chalcone Derivatives for Imaging of Amyloid β Plaques by SPECT. *Bioorg. Med. Chem.* **2014**, *22*, 2622–2628. DOI: 10.1016/j.bmc.2014.03.032.

(401) Qu, W.; Choi, S. R.; Hou, C.; Zhuang, Z.; Oya, S.; Zhang, W.; Kung, M. P.; Manchandra, R.; Skovronsky, D. M.; Kung, H. F. Synthesis and Evaluation of Indolinyl- and Indolylphenylacetylenes as PET Imaging Agents for β-Amyloid Plaques. *Bioorg. Med. Chem. Lett.* **2008**, *18*, 4823–4827. DOI: 10.1016/j.bmcl.2008.07.077.

(402) Cui, M.; Wang, X.; Yu, P.; Zhang, J.; Li, Z.; Zhang, X.; Yang, Y.; Ono, M.; Jia, H.; Saji, H.; Liu, B. Synthesis and Evaluation of Novel 18f Labeled 2-Pyridinylbenzoxazole and 2-Pyridinylbenzothiazole Derivatives as

Ligands for Positron Emission Tomography (PET) Imaging of β-Amyloid Plaques. *J. Med. Chem.* **2012**, *55* (21), 9283–9296. DOI: 10.1021/jm300973k.

(403) Cui, M. C.; Li, Z. J.; Tang, R. K.; Liu, B. L. Synthesis and Evaluation of Novel Benzothiazole Derivatives Based on the Bithiophene Structure as Potential Radiotracers for β-Amyloid Plaques in Alzheimer's Disease. *Bioorg. Med. Chem.* **2010**, *18*, 2777/2784. DOI: 10.1016/j.bmc.2010.02.002.

(404) Akasaka, T.; Watanabe, H.; Kaide, S.; Iikuni, S.; Hasegawa, M.; Ono, M. Synthesis and Evaluation of Novel Radioiodinated Phenylbenzofuranone Derivatives as α-Synuclein Imaging Probes. *Bioorg. Med. Chem. Lett.* **2022**, *64*, 128679. DOI: 10.1016/j.bmcl.2022.128679.

(405) Swahn, B. M.; Sandell, J.; Pyring, D.; Bergh, M.; Jeppsson, F.; Juréus, A.; Neelissen, J.; Johnström, P.; Schou, M.; Svensson, S. Synthesis and Evaluation of Pyridylbenzofuran, Pyridylbenzothiazole and Pyridylbenzoxazole Derivatives as 18F-PET Imaging Agents for β-Amyloid Plaques. *Bioorg. Med. Chem. Lett.* **2012**, *22* (13), 4332–4337. DOI: 10.1016/j.bmcl.2012.05.011.

(406) Jung, S. J.; Park, Y. D.; Park, J. H.; Yang, S. D.; Hur, M. G.; Yu, K. H. Synthesis and Evaluation of Thioflavin-T Analogs as Potential Imaging Agents for Amyloid Plaques. *Med. Chem. Res.* **2013**, *22* (9), 4263–4268. DOI: 10.1007/s00044-012-0414-2.

(407) Serdons, K.; Van Laere, K.; Janssen, P.; Kung, H. F.; Bormans, G.; Verbruggen, A. Synthesis and Evaluation of Three 18 F-Labeled Aminophenylbenzothiazoles as Amyloid Imaging Agents. *J Med Chem* **2009**, *52*, 7090–7102. DOI: 10.1021/jm900871v.

(408) Cai, L.; Chin, F. T.; Pike, V. W.; Toyama, H.; Liow, J. S.; Zoghbi, S. S.; Modell, K.; Briard, E.; Shetty, H. U.; Sinclair, K.; Donohue, S.; Tipre, D.; Kung, M. P.; Dagostin, C.; Widdowson, D. A.; Green, M.; Gao, W.; Herman, M. M.; Ichise, M.; Innis, R. B. Synthesis and Evaluation of Two 18F-Labeled 6-Iodo-2-(4′-N,N-Dimethylamino)Phenylimidazo[1,2-a]Pyridine Derivatives as Prospective Radioligands for β-Amyloid in Alzheimer's Disease. *J. Med. Chem.* **2004**, *47* (9), 2208–2218. DOI: 10.1021/jm030477w.

(409) Zeng, F.; Southerland, J. A.; Voll, R. J.; Votaw, J. R.; Williams, L.; Ciliax, B. J.; Levey, A. I.; Goodman, M. M. Synthesis and Evaluation of Two 18F-Labeled Imidazo[1,2-a]Pyridine Analogues as Potential Agents for Imaging β-Amyloid in Alzheimer's Disease. *Bioorg. Med. Chem. Lett.* **2006**, *16* (11), 3015–3018. DOI: 10.1016/j.bmcl.2006.02.055.

(410) Zheng, W.; Huang, Y.; Chen, H.; Jiang, Z.; Yu, Z.; Yang, T.; Zhang, L.; Cheng, X.; Liu, Y.; Liu, Q.; Ji, X.; Wu, Z. Synthesis and In Vitro and In Vivo Evaluation of 18F-Labeled Positron Emission Tomography Tracers for Imaging Aβ Plaques. *ACS Chem. Neurosci.* **2023**, *14* (5), 988–1003. DOI: 10.1021/acschemneuro.3c00025.

(411) Ribeiro Morais, G.; Vicente Miranda, H.; Santos, I. C.; Santos, I.; Outeiro, T. F.; Paulo, A. Synthesis and in Vitro Evaluation of Fluorinated Styryl Benzazoles as Amyloid-Probes. *Bioorg. Med. Chem.* **2011**, *19* (24), 7698–7710. DOI: 10.1016/j.bmc.2011.09.065.

(412) Yu, L.; Cui, J.; Padakanti, P. K.; Engel, L.; Bagchi, D. P.; Kotzbauer, P. T.; Tu, Z. Synthesis and in Vitro Evaluation of α-Synuclein Ligands. *Bioorg. Med. Chem.* **2012**, *20* (15), 4625–4634. DOI: 10.1016/j.bmc.2012.06.023.

(413) Ghadami, S. A.; Shevidi, S.; Hosseinzadeh, L.; Adibi, H. Synthesis and in Vitro Quantification of Amyloid Fibrils by Barbituric and Thiobarbituric Acid-Based Chromene Derivatives. *Biophys. Chem.* **2021**, *269*, 106522. DOI: 10.1016/j.bpc.2020.106522.

(414) Vasdev, N.; Cao, P.; Van Oosten, E. M.; Wilson, A. A.; Houle, S.; Hao, G.; Sun, X.; Slavine, N.; Alhasan, M.; Antich, P. P.; Bonte, F. J.; Kulkarni, P. Synthesis and PET Imaging Studies of [18F]2-Fluoroquinolin-8-Ol ([18F]CABS13) in Transgenic Mouse Models of Alzheimer's Disease. *MedChemComm* **2012**, *3* (10), 1228–1230. DOI: 10.1039/c2md20075a.

(415) Cai, L.; Cuevas, J.; Temme, S.; Herman, M. M.; Dagostin, C.; Widdowson, D. A.; Innis, R. B.; Pike, V. W. Synthesis and Structure-Affinity Relationships of New 4-(6-Iodo-H- Imidazo[1,2-a]Pyridin-2-Yl)-N-Dimethylbenzeneamine Derivatives as Ligands for Human β-Amyloid Plaques. *J. Med. Chem.* **2007**, *50* (19), 4746–4758. DOI: 10.1021/jm0702231.

(416) Cui, M.; Ono, M.; Kimura, H.; Liu, B.; Saji, H. Synthesis and Structure-Affinity Relationships of Novel Dibenzylideneacetone Derivatives as Probes for ß-Amyloid Plaques. *J. Med. Chem.* **2011**, *54* (7), 2225–2240. DOI: 10.1021/jm101404k.

(417) Wongso, H.; Ono, M.; Yamasaki, T.; Kumata, K.; Higuchi, M.; Zhang, M.-R.; J. Fulham, M.; Katsifis, A.; A. Keller, P. Synthesis and Structure–Activity Relationship (SAR) Studies of 1,2,3-Triazole, Amide, and Ester-Based Benzothiazole Derivatives as Potential Molecular Probes for Tau Protein. *RSC Med. Chem.* **2023**, *14* (5), 858. DOI: 10.1039/D2MD00358A.

(418) Hausner, S. H.; Alagille, D.; Koren, A. O.; Amici, L.; Staley, J. K.; Cosgrove, K. P.; Baldwin, R. M.; Tamagnan, G. D. Synthesis of 5- and 6-Substituted 2-(4-Dimethylaminophenyl)-1,3-Benzoxazoles and Their in Vitro and in Vivo Evaluation as Imaging Agents for Amyloid Plaque. *Bioorg. Med. Chem. Lett.* **2009**, *19* (2), 543–545. DOI: 10.1016/j.bmcl.2008.05.033.

(419) Serdons, K.; Verduyckt, T.; Vanderghinste, D.; Cleynhens, J.; Borghgraef, P.; Vermaelen, P.; Terwinghe, C.; Van Leuven, F.; Van Laere, K.; Kung, H.; Bormans, G.; Verbruggen, A. Synthesis of 18 F-Labelled 2-(4 0-Fluorophenyl)-1,3-Benzothiazole and Evaluation as Amyloid Imaging Agent in Comparison with [11 C]PIB. *Bioorg. Med. Chem. Lett.* **2008**, *19*, 602–605. DOI: 10.1016/j.bmcl.2008.12.069.

(420) Tavakoli, M.; Ghadami, S. A.; Adibi, H.; Gulcan, H. O. Synthesis of Benzylidene-Benzofuranone Derivatives as Probes for Detection of Amyloid Fibrils in Cells. *J. Biomol. Struct. Dyn.* **2023**, *41* (24), 14989–15002. DOI: 10.1080/07391102.2023.2184635.

(421) Zhuang, Z. P.; Kung, M. P.; Kung, H. F. Synthesis of Biphenyltrienes as Probes for β-Amyloid Plaques. *J. Med. Chem.* **2006**, *49* (9), 2841–2844. DOI: 10.1021/jm051020k.

(422) Parhi, A. K.; Kung, M. P.; Ploessl, K.; Kung, H. F. Synthesis of Fluorescent Probes Based on Stilbenes and Diphenylacetylenes Targeting β-Amyloid Plaques. *Tetrahedron Lett.* **2008**, *49* (21), 3395–3399. DOI: 10.1016/j.tetlet.2008.03.130.

(423) Young, S. C.; Soo, J. L.; Seung, J. O.; Dae, H. M.; Dong, J. K.; Cho, C. G.; Kyung, H. Y. Synthesis of Functionalized Benzoxazoles and Their Binding Affinities to Aβ42 Fibrils. *Bull. Korean Chem. Soc.* **2008**, *29* (9), 1765–1768. DOI: 10.5012/bkcs.2008.29.9.1765.

(424) Kim, S.; Lee, H. J.; Nam, E.; Jeong, D.; Cho, J.; Lim, M. H.; You, Y. Tailoring Hydrophobic Interactions between Probes and Amyloid-β Peptides for Fluorescent Monitoring of Amyloid-β Aggregation. *ACS Omega* **2018**, *3* (5), 5141–5154. DOI: 10.1021/acsomega.8b00286.

(425) Zunbul, Z.; An, J.; Aziz, H.; Shin, J.; Lim, S.; Yu, L.; Kim, Y. K.; Kim, J. S. Tailoring Hydrophobicity of Thioflavin T to Optimize Aβ Fibril Bioimaging. *Adv. NanoBiomed Res.* **2023**, *3* (6), 2200161. DOI: 10.1002/anbr.202200161.

(426) Kudo, Y.; Furumoto, S.; Okamura, N. Tau Imaging Probe. US9452985B2, 2016.

(427) Okamura, N.; Harada, R.; Furumoto, S.; Arai, H.; Yanai, K.; Kudo, Y. Tau PET Imaging in Alzheimer's Disease. *Curr. Neurol. Neurosci. Rep.* **2014**, *14* (11), 500. DOI: 10.1007/s11910-014-0500-6.

(428) Han, H.; Cho, C. G.; Lansbury, P. T. Technetium Complexes for the Quantitation of Brain Amyloid. *J. Am. Chem. Soc.* **1996**, *118* (18), 4506. DOI: 10.1021/ja960207l.

(429) Smid, L. M.; Vovko, T. D.; Popovic, M.; Petrič, A.; Kepe, V.; Barrio, J. R.; Vidmar, G.; Bresjanac, M. The 2,6-Disubstituted Naphthalene Derivative FDDNP Labeling Reliably Predicts Congo Red Birefringence of Protein Deposits in Brain Sections of Selected Human Neurodegenerative Diseases. *Brain Pathol.* **2006**, *16* (2), 124–130. DOI: 10.1111/j.1750-3639.2006.00006.x.

(430) Kang, J.; Han, K. The Amide Derivatives of Chrysamine G Bind to the β-Amyloid Fibril. *Bull. Korean Chem. Soc.* **2001**, *22* (10), 1065. DOI: JAKO200113464477530.

(431) Klunk, W. E.; Wang, Y.; Huang, G. feng; Debnath, M. L.; Holt, D. P.; Shao, L.; Hamilton, R. L.; Ikonomovic, M. D.; DeKosky, S. T.; Mathis, C. A. The Binding of 2-(4′-Methylaminophenyl)Benzothiazole to Postmortem Brain Homogenates Is Dominated by the Amyloid Component. *J. Neurosci.* **2003**, *23* (6), 2086–2092. DOI: 10.1523/jneurosci.23-06-02086.2003.

(432) Josephson, L.; Stratman, N.; Liu, Y.; Qian, F.; Liang, S. H.; Vasdev, N.; Patel, S. The Binding of BF-227-Like Benzoxazoles to Human α-Synuclein and Amyloid β Peptide Fibrils. *Mol. Imaging* **2018**, *17*, 1–6. DOI: 10.1177/1536012118796297.

(433) Okamura, N.; Harada, R.; Ishiki, A.; Kikuchi, A.; Nakamura, T.; Kudo, Y. The Development and Validation of Tau PET Tracers: Current Status and Future Directions. **2018**, *6*, 305–316. DOI: 10.1007/s40336-018-0290-y.

(434) Schmidt, M. L.; Schuck, T.; Sheridan, S.; Kung, M. P.; Kung, H.; Zhuang, Z. P.; Bergeron, C.; Lamarche, J. S.; Skovronsky, D.; Giasson, B. I.; Lee, V. M. Y.; Trojanowski, J. Q. The Fluorescent Congo Red Derivative, (Trans, Trans)-1-Bromo-2,5-Bis-(3-Hydroxycarbonyl-4-Hydroxy)Styrylbenzene (Bsb), Labels Diverse β-Pleated Sheet Structures in Postmortem Human Neurodegenerative Disease Brains. *Am. J. Pathol.* **2001**, *159* (3), 937–943. DOI: 10.1016/S0002-9440(10)61769-5.

(435) Di Nanni, A.; Saw, R. S.; Bowden, G. D.; Bidesi, N. S. R.; Bjerregaard-Andersen, K.; Korat, Š.; Herth, M. M.; Pichler, B. J.; Herfert, K.; Maurer, A. The Structural Combination of SIL and MODAG Scaffolds Fails to Enhance Binding to α-Synuclein but Reveals Promising Affinity to Amyloid β. *Molecules* **2023**, *28* (10), 4001. DOI: 10.3390/molecules28104001.

(436) Zhou, K.; Fu, H.; Feng, L.; Cui, M.; Dai, J.; Liu, B. The Synthesis and Evaluation of Near-Infrared Probes with Barbituric Acid Acceptors for in Vivo Detection of Amyloid Plaques. *Chem. Commun.* **2015**, *51* (58), 11665–11668. DOI: 10.1039/c5cc03662c.

(437) Vermeiren, C.; Motte, P.; Viot, D.; Mairet-Coello, G.; Courade, J.-P.; Citron, M.; Mercier, J.; Hannestad, J.; Gillard, M. The Tau Positron-Emission Tomography Tracer AV-1451 Binds with Similar Affinities to Tau Fibrils and Monoamine Oxidases. *Mov. Disord.* **2018**, *33* (2), 273–281. DOI: 10.1002/mds.27271.

(438) Smith, R.; Capotosti, F.; Schain, M.; Ohlsson, T.; Vokali, E.; Molette, J.; Touilloux, T.; Hliva, V.; Dimitrakopoulos, I. K.; Puschmann, A.; Jögi, J.; Svenningsson, P.; Andréasson, M.; Sandiego, C.; Russell, D. S.; Miranda-Azpiazu, P.; Halldin, C.; Stomrud, E.; Hall, S.; Bratteby, K.; Tampio L'Estrade, E.; Luthi-Carter, R.; Pfeifer, A.; Kosco-Vilbois, M.; Streffer, J.; Hansson, O. The α-Synuclein PET Tracer [18F] ACI-12589 Distinguishes Multiple System Atrophy from Other Neurodegenerative Diseases. *Nat. Commun.* **2023**, *14* (1), 6750. DOI: 10.1038/s41467-023-42305-3.

(439) Leuma Yona, R.; Mazères, S.; Faller, P.; Gras, E. Thioflavin Derivatives as Markers for Amyloid-β Fibrils: Insights into Structural Features Important for High-Affinity Binding. *ChemMedChem* **2008**, *3* (1), 63–66. DOI: 10.1002/cmdc.200700188.

(440) Klunk, W. E.; Jr, C. A. M.; Wang, Y. Thioflavin Derivatives for Use in Antemortem Diagnosis of Alzheimer's Disease and in Vivo Imaging and Prevention of Amyloid Deposition. US10137210B2, 2018.

(441) Klunk, W. E.; Jr, C. A. M.; Wang, Y. Thioflavin Derivatives for Use in Antemortem Diagnosis of Alzheimer's Disease and in Vivo Imaging and Prevention of Amyloid Deposition. US10137210B2, 2017.

(442) Rodríguez-Rodríguez, C.; Telpoukhovskaia, M. A.; Alí-Torres, J.; Rodríguez-Santiago, L.; Manso, Y.; Bailey, G. A.; Hidalgo, J.; Sodupe, M.; Orvig, C. Thioflavin-Based Molecular Probes for Application in Alzheimer's Disease: From in Silico to in Vitro Models. *Metallomics* **2015**, *7* (1), 83–92. DOI: 10.1039/c4mt00167b.

(443) Lantz, L.; Shirani, H.; Ghetti, B.; Vidal, R.; Klingstedt, T.; Nilsson, P. Thiophene-Based Ligands for Histological Multiplex Spectral Detection of Distinct Protein Aggregates in Alzheimer´s Disease. *Chem. – Eur. J.* **2023**, *29* (21), e202203568. DOI: 10.1002/chem.202203568.

(444) Needham, L.-M.; Weber, J.; Varela, J. A.; Fyfe, J. W. B.; Do, D. T.; Xu, C. K.; Tutton, L.; Cliffe, R.; Keenlyside, B.; Klenerman, D.; Dobson, C. M.; Hunter, C. A.; Müller, K. H.; O'Holleran, K.; Bohndiek, S. E.; Snaddon, T. N.; Lee, S. F. ThX – a next-Generation Probe for the Early Detection of Amyloid Aggregates. *Chem. Sci.* **2020**, *11*, 4578–4583. DOI: 10.1039/C9SC04730A.

(445) Zhang, J.; Sandberg, A.; Wu, X.; Nyströ, S.; Lindgren, M.; Konradsson, P.; Hammarströ, P. Trans-Stilbenoids with Extended Fluorescence Lifetimes for the Characterization of Amyloid Fibrils. *ACS Omega* **2017**, *2*, 4693–4704. DOI: 10.1021/acsomega.7b00535.

(446) Kovalska, V. B.; Losytskyy, M. Y.; Tolmachev, O. I.; Slominskii, Y. L.; Segers-Nolten, G. M. J.; Subramaniam, V.; Yarmoluk, S. M. Tri- and Pentamethine Cyanine Dyes for Fluorescent Detection of α-Synuclein Oligomeric Aggregates. *J. Fluoresc.* **2012**, *22* (6), 1441–1448. DOI: 10.1007/s10895-012-1081-x.
(447) Smith, N. W.; Alonso, A.; Brown, C. M.; Dzyuba, S. V. Triazole-Containing BODIPY Dyes as Novel Fluorescent Probes for Soluble Oligomers of Amyloid Aβ1-42 Peptide. *Biochem. Biophys. Res. Commun.* **2010**, *391* (3), 1455–1458. DOI: 10.1016/j.bbrc.2009.12.091.
(448) Li, Y.; Yang, J.; Liu, H.; Yang, J.; Du, L.; Feng, H.; Tian, Y.; Cao, J.; Ran, C. Tuning the Stereo-Hindrance of a Curcumin Scaffold for the Selective Imaging of the Soluble Forms of Amyloid Beta Species. *Chem. Sci.* **2017**, *8*, 7710–7717. DOI: 10.1039/c7sc02050c.
(449) Kim, D.; Moon, H.; Baik, S. H.; Singha, S.; Jun, Y. W.; Wang, T.; Kim, K. H.; Park, B. S.; Jung, J.; Mook-Jung, I.; Ahn, K. H. Two-Photon Absorbing Dyes with Minimal Autofluorescence in Tissue Imaging: Application to in Vivo Imaging of Amyloid-β Plaques with a Negligible Background Signal. *J. Am. Chem. Soc.* **2015**, *137* (21), 6781–6789. DOI: 10.1021/jacs.5b03548.
(450) Klunk, W. E.; Wang, Y.; Huang, G. feng; Debnath, M. L.; Holt, D. P.; Mathis, C. A. Uncharged Thioflavin-T Derivatives Bind to Amyloid-Beta Protein with High Affinity and Readily Enter the Brain. *Life Sci.* **2001**, *69* (13), 1471–1484. DOI: 10.1016/S0024-3205(01)01232-2.
(451) Tooyama, I.; Taguchi, H.; Morikawa, S.; Urushitani, M.; Yanahisawa, W.; Nagae, T.; Shirai, N.; Hirao, K.; Kato, M.; Kimura, H.; Okada, T. Imaging Diagnostic Agent and Extracorporeal Diagnostic Agent for Incurable Neurological Diseases. US8956589B2, 2015
(452) Luke, E. A.; Yadon, M. C.; Cummings, J.; Hudson, F. M.; Lake, T.; Hu, Q.; Cam, J.; Snow, A. D. Compounds for the Treatment of Neurodegenerative Diseases. US9085549B2, 2015.
(453) Walji, A. M.; Hostetler, E.; Grrshock, T. J.; Li, J.; Moore, K. P.; Bennacef, I.; Mulhearn, J.; Selnick, H.; Wang, Y.; Yang, K.; Fu, J. Pyrrolo[2,3-C]Pyridines as Imaging Agents for Neurofibrilary Tangles. US10022461B2, 2018.
(454) Harada, R.; Okamura, N.; Furumoto, S.; Yoshikawa, T.; Arai, H.; Yanai, K.; Kudo, Y. Use of a Benzimidazole Derivative BF-188 in Fluorescence Multispectral Imaging for Selective Visualization of Tau Protein Fibrils in the Alzheimer's Disease Brain. *Mol Imaging Biol* **2014**, *16*, 19–27. DOI: 10.1007/s11307-013-0667-2.
(455) Wey, S.-P.; Weng, C.-C.; Lin, K.-J.; Yao, C.-H.; Yen, T.-C.; Kung, H. F.; Skovronsky, D.; Kung, M.-P. Validation of an (18)F-Labeled Biphenylalkyne as a Positron Emission Tomography Imaging Agent for Beta-Amyloid Plaques. *Nucl. Med. Biol.* **2009**, *36* (4), 411–417. DOI: 10.1016/j.nucmedbio.2009.01.013.
(456) Gharai, P. K.; Khan, J.; Mallesh, R.; Garg, S.; Saha, A.; Ghosh, S.; Ghosh, S. Vanillin Benzothiazole Derivative Reduces Cellular Reactive Oxygen Species and Detects Amyloid Fibrillar Aggregates in Alzheimer's Disease Brain. *ACS Chem. Neurosci.* **2023**, *14* (4), 773–786. DOI: 10.1021/acschemneuro.2c00771.
(457) Zhang, M.; Fu, H.; Hu, W.; Leng, J.; Zhang, Y. Versatile Dicyanomethylene-Based Fluorescent Probes for the Detection of β-Amyloid in Alzheimer's Disease: A Theoretical Perspective. *Int. J. Mol. Sci.* **2022**, *23* (15), 8619. DOI: 10.3390/ijms23158619.
(458) Peng, C.; Wang, X.; Li, Y.; Li, H.-W.; Wong, M. S. Versatile Fluorescent Probes for Near-Infrared Imaging of Amyloid-b Species in Alzheimer's Disease Mouse Model. *J Mater Chem B* **1986**, *7*, 1986–1995. DOI: 10.1039/c9tb00161a.
(459) Lemoine, L.; Saint-Aubert, L.; Marutle, A.; Antoni, G.; Eriksson, J. P.; Ghetti, B.; Okamura, N.; Nennesmo, I.; Gillberg, P. G.; Nordberg, A.; Gillberg, G.; Nordberg, A. Visualization of Regional Tau Deposits Using 3H-THK5117 in Alzheimer Brain Tissue. *Acta Neuropathol. Commun.* **2015**, *3*, 40. DOI: 10.1186/s40478-015-0220-4.
(460) Wang, Y.; Mei, D.; Zhang, X.; Qu, D.-H.; Mei, J. Visualizing Aβ Deposits in Live Young AD Model Mice with a Simple Red/near-Infrared-Fluorescent AIEgen. *Sci. China Chem.* **2022**, *65* (2), 339–352. DOI: 10.1007/s11426-021-1113-0.

(461) Koole, M.; Lewis, D. M.; Buckley, C.; Nelissen, N.; Vandenbulcke, M.; Brooks, D. J.; Vandenberghe, R.; Van Laere, K. Whole-Body Biodistribution and Radiation Dosimetry of 18F-GE067: A Radioligand for in Vivo Brain Amyloid Imaging. *J. Nucl. Med.* **2009**, *50* (5), 818–822. DOI: 10.2967/jnumed.108.060756.

(462) Uzuegbunam, B.; Paslawski, W.; Zhou, Y.; Ågren, H.; Längström, B.; Weber, W.; Yousefi, B. Optimized Disarybisthiazole Derivatives with High Affinity to Alpha-Synuclein Aggregates and Improved Pharmacokinetics. *Nuklearmedizin* **2021**, *60* (02), V22. DOI: 10.1055/S-0041-1726777.

(463) Agdeppa, E. D.; Kepe, V.; Petriĉ, A.; Satyamurthy, N.; Liu, J.; Huang, S. C.; Small, G. W.; Cole, G. M.; Barrio, J. R. In Vitro Detection of (S)-Naproxen and Ibuprofen Binding to Plaques in the Alzheimer's Brain Using the Positron Emission Tomography Molecular Imaging Probe 2-(1-{6-[(2-[18F]Fluoroethyl)(Methyl)Amino]-2- Naphthyl}ethylidene)Malononitrile. *Neuroscience* **2003**, *117* (3), 723–730. DOI: 10.1016/S0306-4522(02)00907-7.

(464) Zhao, Y.; Tietz, O.; Kuan, W.-L. L.; Haji-Dheere, A. K.; Thompson, S.; Vallin, B.; Ronchi, E.; Tóth, G.; Klenerman, D.; Aigbirhio., F. I. A Fluorescent Molecular Imaging Probe with Selectivity for Soluble Tau Aggregated Protein. *Chem. Sci.* **2020**, *11*, 4773–4778. DOI: 10.1039/c9sc05620c.

(465) Ni, R.; Gillberg, P. G.; Bergfors, A.; Marutle, A.; Nordberg, A. Amyloid Tracers Detect Multiple Binding Sites in Alzheimer's Disease Brain Tissue. *Brain* **2013**, *136* (7), 2217–2227. DOI: 10.1093/brain/awt142.

(466) Zheng, M. Q.; Yin, D. Z.; Qiao, J. P.; Zhang, L.; Wang, Y. X. Syntheses and Evaluation of Fluorinated Benzothiazole Anilines as Potential Tracers for β-Amyloid Plaques in Alzheimer's Disease. *J. Fluor. Chem.* **2008**, *129* (3), 210–216. DOI: 10.1016/j.jfluchem.2007.11.005.

(467) Gour, N.; Kshtriya, V.; Gupta, S.; Koshti, B.; Singh, R.; Patel, D.; Joshi, K. B. Synthesis and Aggregation Studies of a Pyridothiazole-Based AIEE Probe and Its Application in Sensing Amyloid Fibrillation. *ACS Appl. Bio Mater.* **2019**, *2* (10), 4442–4455. DOI: 10.1021/acsabm.9b00627.

(468) Cao, Y.; Liu, X.; Zhang, J.; Liu, Z.; Fu, Y.; Zhang, D.; Zheng, M.; Zhang, H.; Xu, M.-H. Design of a Coumarin-Based Fluorescent Probe for Efficient In Vivo Imaging of Amyloid-β Plaques. *ACS Chem. Neurosci.* **2023**, *14* (5), 829–838. DOI: 10.1021/acschemneuro.2c00468.

(469) Kim, H. Y.; Chia, W. K.; Hsieh, C.-J.; Saturnino Guarino, D.; Graham, T. J. A.; Lengyel-Zhand, Z.; Schneider, M.; Tomita, C.; Lougee, M. G.; Kim, H. J.; Pagar, V. V.; Lee, H.; Hou, C.; Garcia, B. A.; Petersson, E. J.; O'Shea, J.; Kotzbauer, P. T.; Mathis, C. A.; Lee, V. M.-Y.; Luk, K. C.; Mach, R. H. A Novel Brain PET Radiotracer for Imaging Alpha Synuclein Fibrils in Multiple System Atrophy. *J. Med. Chem.* **2023**, *66* (17), 12185–12202. DOI: 10.1021/acs.jmedchem.3c00779.

(470) Matsuoka, K.; Ono, M.; Takado, Y.; Hirata, K.; Endo, H.; Ohfusa, T.; Kojima, T.; Yamamoto, T.; Onishi, T.; Orihara, A.; Tagai, K.; Takahata, K.; Seki, C.; Shinotoh, H.; Kawamura, K.; Shimizu, H.; Shimada, H.; Kakita, A.; Zhang, M.-R.; Suhara, T.; Higuchi, M. High-Contrast Imaging of α-Synuclein Pathologies in Living Patients with Multiple System Atrophy. *Mov. Disord.* **2022**, *37* (10), 2159–2161. DOI: 10.1002/mds.29186.

(471) Endo, H.; Ono, M.; Takado, Y.; Matsuoka, K.; Takahashi, M.; Tagai, K.; Kataoka, Y.; Hirata, K.; Takahata, K.; Seki, C.; Kokubo, N.; Fujinaga, M.; Mori, W.; Nagai, Y.; Mimura, K.; Kumata, K.; Kikuchi, T.; Shimozawa, A.; Mishra, S. K.; Yamaguchi, Y.; Shimizu, H.; Kakita, A.; Takuwa, H.; Shinotoh, H.; Shimada, H.; Kimura, Y.; Ichise, M.; Suhara, T.; Minamimoto, T.; Sahara, N.; Kawamura, K.; Zhang, M.-R.; Hasegawa, M.; Higuchi, M. Imaging α-Synuclein Pathologies in Animal Models and Patients with Parkinson's and Related Diseases. *Neuron* **2024**, *112* (15), 2540-2557.e8. DOI: 10.1016/j.neuron.2024.05.006.

(472) Rombouts, F. J. R.; Declercq, L.; Andrés, J.-I.; Bottelbergs, A.; Chen, L.; Iturrino, L.; Leenaerts, J. E.; Mariën, J.; Song, F.; Wintmolders, C.; Wuyts, S.; Xia, C. A.; te Riele, P.; Bormans, G.; Vandenberghe, R.; Kolb, H.; Moechars, D. Discovery of N-(4-[18F]Fluoro-5-Methylpyridin-2-Yl)Isoquinolin-6-Amine (JNJ-64326067), a New Promising Tau Positron Emission Tomography Imaging Tracer. *J. Med. Chem.* **2019**, *62* (6), 2974–2987. DOI: 10.1021/acs.jmedchem.8b01759.

(473) Stern, A. M.; Yang, Y.; Jin, S.; Yamashita, K.; Meunier, A. L.; Liu, W.; Cai, Y.; Ericsson, M.; Liu, L.; Goedert, M.; Scheres, S. H. W.; Selkoe, D. J. Abundant Aβ Fibrils in Ultracentrifugal Supernatants of Aqueous

Extracts from Alzheimer's Disease Brains. *Neuron* **2023**, *111* (13), 2012-2020.e4. DOI: 10.1016/j.neuron.2023.04.007.

(474) Li, Y.; Zhao, C.; Luo, F.; Liu, Z.; Gui, X.; Luo, Z.; Zhang, X.; Li, D.; Liu, C.; Li, X. Amyloid Fibril Structure of α-Synuclein Determined by Cryo-Electron Microscopy. *Cell Res.* **2018**, *28* (9), 897–903. DOI: 10.1038/s41422-018-0075-x.

(475) El Mammeri, N.; Duan, P.; Dregni, A. J.; Hong, M. Amyloid Fibril Structures of Tau: Conformational Plasticity of the Second Microtubule-Binding Repeat. *Sci. Adv.* **2023**, *9* (28), eadh4731. DOI: 10.1126/sciadv.adh4731.

(476) Fu, Z.; Crooks, E. J.; Irizarry, B. A.; Zhu, X.; Chowdhury, S.; Van Nostrand, W. E.; Smith, S. O. An Electrostatic Cluster Guides Aβ40 Fibril Formation in Sporadic and Dutch-Type Cerebral Amyloid Angiopathy. *J. Struct. Biol.* **2024**, *216* (2), 108092. DOI: 10.1016/j.jsb.2024.108092.

(477) Lövestam, S.; Koh, F. A.; van Knippenberg, B.; Kotecha, A.; Murzin, A. G.; Goedert, M.; Scheres, S. H. W. Assembly of Recombinant Tau into Filaments Identical to Those of Alzheimer's Disease and Chronic Traumatic Encephalopathy. *eLife* **2022**, *11*, e76494. DOI: 10.7554/eLife.76494.

(478) Schütz, A. K.; Vagt, T.; Huber, M.; Ovchinnikova, O. Y.; Cadalbert, R.; Wall, J.; Güntert, P.; Bcöckmann, A.; Glockshuber, R.; Meier, B. H. Atomic-Resolution Three-Dimensional Structure of Amyloid b Fibrils Bearing the Osaka Mutation. *Angew. Chem. Int. Ed.* **2015**, *54* (1), 331–335. DOI: 10.1002/anie.201408598.

(479) Xiao, Y.; Ma, B.; McElheny, D.; Parthasarathy, S.; Long, F.; Hoshi, M.; Nussinov, R.; Ishii, Y. Aβ(1-42) Fibril Structure Illuminates Self-Recognition and Replication of Amyloid in Alzheimer's Disease. *Nat. Struct. Mol. Biol.* **2015**, *22* (6), 499–505. DOI: 10.1038/nsmb.2991.

(480) Krotee, P.; Griner, S. L.; Sawaya, M. R.; Cascio, D.; Rodriguez, J. A.; Shi, D.; Philipp, S.; Murray, K.; Saelices, L.; Lee, J.; Seidler, P.; Glabe, C. G.; Jiang, L.; Gonen, T.; Eisenberg, D. S. Common Fibrillar Spines of Amyloid-β and Human Islet Amyloid Polypeptide Revealed by Microelectron Diffraction and Structure-Based Inhibitors. *J. Biol. Chem.* **2018**, *293* (8), 2888–2902. DOI: 10.1074/jbc.M117.806109.

(481) Fan, Y.; Sun, Y.; Yu, W.; Tao, Y.; Xia, W.; Liu, Y.; Zhao, Q.; Tang, Y.; Sun, Y.; Liu, F.; Cao, Q.; Wu, J.; Liu, C.; Wang, J.; Li, D. Conformational Change of α-Synuclein Fibrils in Cerebrospinal Fluid from Different Clinical Phases of Parkinson's Disease. *Structure* **2023**, *31* (1), 78-87.e5. DOI: 10.1016/j.str.2022.11.013.

(482) Zhang, S.; Li, J.; Xu, Q.; Xia, W.; Tao, Y.; Shi, C.; Li, D.; Xiang, S.; Liu, C. Conformational Dynamics of an α-Synuclein Fibril upon Receptor Binding Revealed by Insensitive Nuclei Enhanced by Polarization Transfer-Based Solid-State Nuclear Magnetic Resonance and Cryo-Electron Microscopy. *J. Am. Chem. Soc.* **2023**, *145* (8), 4473–4484. DOI: 10.1021/jacs.2c10854.

(483) Benedikt Pfeiffer, P.; Ugrina, M.; Schwierz, N.; Sigurdson, C. J.; Schmidt, M.; Fändrich, M. Cryo-EM Analysis of the Effect of Seeding with Brain-Derived Aβ Amyloid Fibrils. *J. Mol. Biol.* **2023**, 168422. DOI: 10.1016/j.jmb.2023.168422.

(484) Zielinski, M.; Peralta Reyes, F. S.; Gremer, L.; Schemmert, S.; Frieg, B.; Schäfer, L. U.; Willuweit, A.; Donner, L.; Elvers, M.; Nilsson, L. N. G.; Syvänen, S.; Sehlin, D.; Ingelsson, M.; Willbold, D.; Schröder, G. F. Cryo-EM of Aβ Fibrils from Mouse Models Find Tg-APPArcSwe Fibrils Resemble Those Found in Patients with Sporadic Alzheimer's Disease. *Nat. Neurosci.* **2023**, *26* (12), 2073–2080. DOI: 10.1038/s41593-023-01484-4.

(485) Li, B.; Ge, P.; Murray, K. A.; Sheth, P.; Zhang, M.; Nair, G.; Sawaya, M. R.; Shin, W. S.; Boyer, D. R.; Ye, S.; Eisenberg, D. S.; Zhou, Z. H.; Jiang, L. Cryo-EM of Full-Length α-Synuclein Reveals Fibril Polymorphs with a Common Structural Kernel. *Nat. Commun.* **2018**, *9* (1), 1–10. DOI: 10.1038/s41467-018-05971-2.

(486) Kollmer, M.; Close, W.; Funk, L.; Rasmussen, J.; Bsoul, A.; Schierhorn, A.; Schmidt, M.; Sigurdson, C. J.; Jucker, M.; Fändrich, M. Cryo-EM Structure and Polymorphism of Aβ Amyloid Fibrils Purified from Alzheimer's Brain Tissue. *Nat. Commun.* **2019**, *10* (1), 4760. DOI: 10.1038/s41467-019-12683-8.

(487) Guerrero-Ferreira, R.; Taylor, N. M. I.; Mona, D.; Ringler, P.; Lauer, M. E.; Riek, R.; Britschgi, M.; Stahlberg, H. Cryo-EM Structure of Alpha-Synuclein Fibrils. *eLife* **2018**, *7*, e36402. DOI: 10.7554/eLife.36402.

(488) Sun, C.; Zhou, K.; DePaola, P.; Shin, W. S.; Hillyer, T.; Sawaya, M. R.; Zhu, R.; Peng, C.; Zhou, Z. H.; Jiang, L. Cryo-EM Structure of Amyloid Fibril Formed by α-Synuclein Hereditary A53E Mutation Reveals a Distinct Protofilament Interface. *J. Biol. Chem.* **2023**, *299* (4), 104566. DOI: 10.1016/j.jbc.2023.104566.

(489) Sun, Y.; Hou, S.; Zhao, K.; Long, H.; Liu, Z.; Gao, J.; Zhang, Y.; Su, X. D.; Li, D.; Liu, C. Cryo-EM Structure of Full-Length α-Synuclein Amyloid Fibril with Parkinson's Disease Familial A53T Mutation. *Cell Res.* **2020**, *30* (4), 360–362. DOI: 10.1038/s41422-020-0299-4.

(490) Abskharon, R.; Sawaya, M. R.; Boyer, D. R.; Cao, Q.; Nguyen, B. A.; Cascio, D.; Eisenberg, D. S. Cryo-EM Structure of RNA-Induced Tau Fibrils Reveals a Small C-Terminal Core That May Nucleate Fibril Formation. *Proc. Natl. Acad. Sci.* **2022**, *119* (15), e2119952119. DOI: 10.1073/pnas.2119952119.

(491) Yang, Y.; Arseni, D.; Zhang, W.; Huang, M.; Lövestam, S.; Schweighauser, M.; Kotecha, A.; Murzin, A. G.; Peak-Chew, S. Y.; MacDonald, J.; Lavenir, I.; Garringer, H. J.; Gelpi, E.; Newell, K. L.; Kovacs, G. G.; Vidal, R.; Ghetti, B.; Ryskeldi-Falco, B.; Scheres, S. H. W.; Goedert, M. Cryo-EM Structures of Amyloid-b 42 Filaments from Human Brains. *Science* **2022**, *375* (6577), 167–172. DOI: 10.1126/science.abm7285.

(492) Yang, Y.; Zhang, W.; Murzin, A. G.; Schweighauser, M.; Huang, M.; Lövestam, S.; Peak-Chew, S. Y.; Saito, T.; Saido, T. C.; Macdonald, J.; Lavenir, I.; Ghetti, B.; Graff, C.; Kumar, A.; Nordberg, A.; Goedert, M.; Scheres, S. H. W. Cryo-EM Structures of Amyloid-β Filaments with the Arctic Mutation (E22G) from Human and Mouse Brains. *Acta Neuropathol. (Berl.)* **2023**, *145* (3), 325–333. DOI: 10.1007/s00401-022-02533-1.

(493) Yang, Y.; Murzin, A. G.; Peak-Chew, S.; Franco, C.; Garringer, H. J.; Newell, K. L.; Ghetti, B.; Goedert, M.; Scheres, S. H. W. Cryo-EM Structures of Aβ40 Filaments from the Leptomeninges of Individuals with Alzheimer's Disease and Cerebral Amyloid Angiopathy. *Acta Neuropathol. Commun.* **2023**, *11* (1), 191. DOI: 10.1186/s40478-023-01694-8.

(494) Shi, Y.; Ghetti, B.; Goedert, M.; Scheres, S. H. W. Cryo-EM Structures of Chronic Traumatic Encephalopathy Tau Filaments with PET Ligand Flortaucipir. *J. Mol. Biol.* **2023**, *435* (11), 168025. DOI: 10.1016/j.jmb.2023.168025.

(495) Frieg, B.; Han, M.; Giller, K.; Dienemann, C.; Riedel, D.; Becker, S.; Andreas, L. B.; Griesinger, C.; Schröder, G. F. Cryo-EM Structures of Lipidic Fibrils of Amyloid-β (1-40). *Nat. Commun.* **2024**, *15* (1), 1297. DOI: 10.1038/s41467-023-43822-x.

(496) Fitzpatrick, A. W. P. P.; Falcon, B.; He, S.; Murzin, A. G.; Murshudov, G.; Garringer, H. J.; Crowther, R. A.; Ghetti, B.; Goedert, M.; Scheres, S. H. W.; Anthony Crowther, R.; Ghetti, B.; Goedert, M.; Sjors, §; Scheres, H. W. Cryo-EM Structures of Tau Filaments from Alzheimer's Disease. *Nature* **2017**, *547* (7662), 185–190. DOI: 10.1038/nature23002.

(497) Shi, Y.; Murzin, A. G.; Falcon, B.; Epstein, A.; Machin, J.; Tempest, P.; Newell, K. L.; Vidal, R.; Garringer, H. J.; Sahara, N.; Higuchi, M.; Ghetti, B.; Jang, M. K.; Scheres, S. H. W.; Goedert, M. Cryo-EM Structures of Tau Filaments from Alzheimer's Disease with PET Ligand APN-1607. *Acta Neuropathol. (Berl.)* **2021**, *1*, 3. DOI: 10.1007/s00401-021-02294-3.

(498) Tarutani, A.; Lövestam, S.; Zhang, X.; Kotecha, A.; Robinson, A. C.; Mann, D. M. A.; Saito, Y.; Murayama, S.; Tomita, T.; Goedert, M.; Scheres, S. H. W.; Hasegawa, M. Cryo-EM Structures of Tau Filaments from SH-SY5Y Cells Seeded with Brain Extracts from Cases of Alzheimer's Disease and Corticobasal Degeneration. *FEBS Open Bio* **2023**, *13* (8), 1394–1404. DOI: 10.1002/2211-5463.13657.

(499) Schweighauser, M.; Murzin, A. G.; Macdonald, J.; Lavenir, I.; Crowther, R. A.; Scheres, S. H. W.; Goedert, M. Cryo-EM Structures of Tau Filaments from the Brains of Mice Transgenic for Human Mutant P301S Tau. *Acta Neuropathol. Commun.* **2023**, *11* (1), 160. DOI: 10.1186/s40478-023-01658-y.

(500) Li, L.; Nguyen, B. A.; Mullapudi, V.; Li, Y.; Saelices, L.; Joachimiak, L. A. Disease-Associated Patterns of Acetylation Stabilize Tau Fibril Formation. *Structure* **2023**, *31* (9), 1025-1037.e4. DOI: 10.1016/j.str.2023.05.020.

(501) Lövestam, S.; Li, D.; Wagstaff, J. L.; Kotecha, A.; Kimanius, D.; McLaughlin, S. H.; Murzin, A. G.; Freund, S. M. V.; Goedert, M.; Scheres, S. H. W. Disease-Specific Tau Filaments Assemble via Polymorphic Intermediates. *Nature* **2024**, *625* (7993), 119–125. DOI: 10.1038/s41586-023-06788-w.

(502) Do, T. D.; Sangwan, S.; de Almeida, N. E. C.; Ilitchev, A. I.; Giammona, M.; Sawaya, M. R.; Buratto, S. K.; Eisenberg, D. S.; Bowers, M. T. Distal Amyloid β-Protein Fragments Template Amyloid Assembly. *Protein Sci.* **2018**, *27* (7), 1181–1190. DOI: 10.1002/pro.3375.

(503) Hojjatian, A.; Dasari, A. K. R.; Sengupta, U.; Taylor, D.; Daneshparvar, N.; Yeganeh, F. A.; Dillard, L.; Michael, B.; Griffin, R. G.; Borgnia, M.; Kayed, R.; Taylor, K. A.; Lim, K. H. Distinct Cryo-EM Structure of α-Synuclein Filaments Derived by Tau. bioRxiv January 3, 2021, p 2020.12.31.424989. DOI: 10.1101/2020.12.31.424989 (accessed 2024-05-26).

(504) Gremer, L.; Schölzel, D.; Schenk, C.; Reinartz, E.; Labahn, J.; Ravelli, R. B. G.; Tusche, M.; Lopez-Iglesias, C.; Hoyer, W.; Heise, H.; Willbold, D.; Schröder, G. F. Fibril Structure of Amyloid-β(1–42) by Cryo–Electron Microscopy. *Science* **2017**, *358* (6359), 116–119. DOI: 10.1126/science.aao2825.

(505) Tao, Y.; Sun, Y.; Lv, S.; Xia, W.; Zhao, K.; Xu, Q.; Zhao, Q.; He, L.; Le, W.; Wang, Y.; Liu, C.; Li, D. Heparin Induces α-Synuclein to Form New Fibril Polymorphs with Attenuated Neuropathology. *Nat. Commun.* **2022**, *13* (1), 4226. DOI: 10.1038/s41467-022-31790-7.

(506) Zhang, W.; Falcon, B.; Murzin, A. G.; Fan, J.; Crowther, R. A.; Goedert, M.; Scheres, S. H. W. Heparin-Induced Tau Filaments Are Polymorphic and Differ from Those in Alzheimer's and Pick's Diseases. *eLife* **2019**, *8*, e43584. DOI: 10.7554/eLife.43584.

(507) Chang, A.; Xiang, X.; Wang, J.; Lee, C.; Arakhamia, T.; Simjanoska, M.; Wang, C.; Carlomagno, Y.; Zhang, G.; Dhingra, S.; Thierry, M.; Perneel, J.; Heeman, B.; Forgrave, L. M.; DeTure, M.; DeMarco, M. L.; Cook, C. N.; Rademakers, R.; Dickson, D. W.; Petrucelli, L.; Stowell, M. H. B.; Mackenzie, I. R. A.; Fitzpatrick, A. W. P. Homotypic Fibrillization of TMEM106B across Diverse Neurodegenerative Diseases. *Cell* **2022**, *185* (8), 1346-1355.e15. DOI: 10.1016/j.cell.2022.02.026.

(508) Qi, C.; Hasegawa, M.; Takao, M.; Sakai, M.; Sasaki, M.; Mizutani, M.; Akagi, A.; Iwasaki, Y.; Miyahara, H.; Yoshida, M.; Scheres, S. H. W.; Goedert, M. Identical Tau Filaments in Subacute Sclerosing Panencephalitis and Chronic Traumatic Encephalopathy. *Acta Neuropathol. Commun.* **2023**, *11* (1), 74. DOI: 10.1186/s40478-023-01565-2.

(509) Sokratian, A.; Gram, H.; Jensen, P. H.; West, A. B. Identification of Novel α-Synuclein Assemblies in Lewy Body Disease. *Mov. Disord. Off. J. Mov. Disord. Soc.* **2023**, *38* (1), 21–22. DOI: 10.1002/mds.29272.

(510) Louros, N.; Wilkinson, M.; Tsaka, G.; Ramakers, M.; Morelli, C.; Garcia, T.; Gallardo, R.; D'Haeyer, S.; Goossens, V.; Audenaert, D.; Thal, D. R.; Mackenzie, I. R.; Rademakers, R.; Ranson, N. A.; Radford, S. E.; Rousseau, F.; Schymkowitz, J. Local Structural Preferences in Shaping Tau Amyloid Polymorphism. *Nat. Commun.* **2024**, *15* (1), 1028. DOI: 10.1038/s41467-024-45429-2.

(511) Cerofolini, L.; Ravera, E.; Bologna, S.; Wiglenda, T.; Böddrich, A.; Purfürst, B.; Benilova, I.; Korsak, M.; Gallo, G.; Rizzo, D.; Gonnelli, L.; Fragai, M.; De Strooper, B.; Wanker, E. E.; Luchinat, C. Mixing Aβ(1-40) and Aβ(1-42) Peptides Generates Unique Amyloid Fibrils. *Chem. Commun.* **2020**, *56* (62), 8830–8833. DOI: 10.1039/d0cc02463e.

(512) Sgourakis, N. G.; Yau, W. M.; Qiang, W. Modeling an In-Register, Parallel "Iowa" Aβ Fibril Structure Using Solid-State NMR Data from Labeled Samples with Rosetta. *Structure* **2015**, *23* (1), 216–227. DOI: 10.1016/j.str.2014.10.022.

(513) Wiltzius, J. J. W.; Landau, M.; Nelson, R.; Sawaya, M. R.; Apostol, M. I.; Goldschmidt, L.; Soriaga, A. B.; Cascio, D.; Rajashankar, K.; Eisenberg, D. Molecular Mechanisms for Protein-Encoded Inheritance. *Nat. Struct. Mol. Biol.* **2009**, *16* (9), 973–978. DOI: 10.1038/nsmb.1643.

(514) Ghosh, U.; Thurber, K. R.; Yau, W. M.; Tycko, R. Molecular Structure of a Prevalent Amyloid-β Fibril Polymorph from Alzheimer's Disease Brain Tissue. *Proc. Natl. Acad. Sci. U. S. A.* **2021**, *118* (4), e2023089118. DOI: 10.1073/pnas.2023089118.
(515) Hu, Z. W.; Vugmeyster, L.; Au, D. F.; Ostrovsky, D.; Sun, Y.; Qiang, W. Molecular Structure of an N-Terminal Phosphorylated β-Amyloid Fibril. *Proc. Natl. Acad. Sci. U. S. A.* **2019**, *166* (23), 11253–11258. DOI: 10.1073/pnas.1818530116.
(516) Lu, J. X.; Qiang, W.; Yau, W. M.; Schwieters, C. D.; Meredith, S. C.; Tycko, R. Molecular Structure of β-Amyloid Fibrils in Alzheimer's Disease Brain Tissue. *Cell* **2013**, *154* (6), 1257–1268. DOI: 10.1016/j.cell.2013.08.035.
(517) Yang, Y.; Garringer, H. J.; Shi, Y.; Lövestam, S.; Peak-Chew, S.; Zhang, X.; Kotecha, A.; Bacioglu, M.; Koto, A.; Takao, M.; Spillantini, M. G.; Ghetti, B.; Vidal, R.; Murzin, A. G.; Scheres, S. H. W.; Goedert, M. New SNCA Mutation and Structures of α-Synuclein Filaments from Juvenile-Onset Synucleinopathy. *Acta Neuropathol. (Berl.)* **2023**, *145* (5), 561–572. DOI: 10.1007/s00401-023-02550-8.
(518) Falcon, B.; Zivanov, J.; Zhang, W.; Murzin, A. G.; Garringer, H. J.; Vidal, R.; Crowther, R. A.; Newell, K. L.; Ghetti, B.; Goedert, M.; Scheres, S. H. W. Novel Tau Filament Fold in Chronic Traumatic Encephalopathy Encloses Hydrophobic Molecules. *Nature* **2019**, *568* (7752), 420–423. DOI: 10.1038/s41586-019-1026-5.
(519) Zhang, W.; Tarutani, A.; Newell, K. L.; Murzin, A. G.; Matsubara, T.; Falcon, B.; Vidal, R.; Garringer, H. J.; Shi, Y.; Ikeuchi, T.; Murayama, S.; Ghetti, B.; Hasegawa, M.; Goedert, M.; Scheres, S. H. W. Novel Tau Filament Fold in Corticobasal Degeneration. *Nature* **2020**, *580* (7802), 283–287. DOI: 10.1038/s41586-020-2043-0.
(520) Balana, A. T.; Mahul-Mellier, A.-L.; Nguyen, B. A.; Horvath, M.; Javed, A.; Hard, E. R.; Jasiqi, Y.; Singh, P.; Afrin, S.; Pedretti, R.; Singh, V.; Lee, V. M.-Y.; Luk, K. C.; Saelices, L.; Lashuel, H. A.; Pratt, M. R. O-GlcNAc Forces an α-Synuclein Amyloid Strain with Notably Diminished Seeding and Pathology. *Nat. Chem. Biol.* **2024**, 1–10. DOI: 10.1038/s41589-024-01551-2.
(521) Liu, D.; Wei, Q.; Xia, W.; He, C.; Zhang, Q.; Huang, L.; Wang, X.; Sun, Y.; Ma, Y.; Zhang, X.; Wang, Y.; Shi, X.; Liu, C.; Dong, S. O-Glycosylation Induces Amyloid-β To Form New Fibril Polymorphs Vulnerable for Degradation. *J. Am. Chem. Soc.* **2021**, *143* (48), 20216–20223. DOI: 10.1021/jacs.1c08607.
(522) Zhao, K.; Li, Y.; Liu, Z.; Long, H.; Zhao, C.; Luo, F.; Sun, Y.; Tao, Y.; Su, X. dong; Li, D.; Li, X.; Liu, C. Parkinson's Disease Associated Mutation E46K of α-Synuclein Triggers the Formation of a Distinct Fibril Structure. *Nat. Commun.* **2020**, *11* (1), 1–9. DOI: 10.1038/s41467-020-16386-3.
(523) Zhao, K.; Lim, Y. J.; Liu, Z.; Long, H.; Sun, Y.; Hu, J. J.; Zhao, C.; Tao, Y.; Zhang, X.; Li, D.; Li, Y. M.; Liu, C. Parkinson's Disease-Related Phosphorylation at Tyr39 Rearranges α-Synuclein Amyloid Fibril Structure Revealed by Cryo-EM. *Proc. Natl. Acad. Sci. U. S. A.* **2020**, *117* (33), 20305–20315. DOI: 10.1073/PNAS.1922741117.
(524) Arakhamia, T.; Lee, C. E.; Carlomagno, Y.; Duong, D. M.; Kundinger, S. R.; Wang, K.; Williams, D.; DeTure, M.; Dickson, D. W.; Cook, C. N.; Seyfried, N. T.; Petrucelli, L.; Fitzpatrick, A. W. P. Posttranslational Modifications Mediate the Structural Diversity of Tauopathy Strains. *Cell* **2020**, *180* (4), 633-644.e12. DOI: 10.1016/j.cell.2020.01.027.
(525) Lövestam, S.; Schweighauser, M.; Matsubara, T.; Murayama, S.; Tomita, T.; Ando, T.; Hasegawa, K.; Yoshida, M.; Tarutani, A.; Hasegawa, M.; Goedert, M.; Scheres, S. H. W. Seeded Assembly *in Vitro* Does Not Replicate the Structures of A-synuclein Filaments from Multiple System Atrophy. *FEBS Open Bio* **2021**, *11* (4), 999–1013. DOI: 10.1002/2211-5463.13110.
(526) Tuttle, M. D.; Comellas, G.; Nieuwkoop, A. J.; Covell, D. J.; Berthold, D. A.; Kloepper, K. D.; Courtney, J. M.; Kim, J. K.; Barclay, A. M.; Kendall, A.; Wan, W.; Stubbs, G.; Schwieters, C. D.; Lee, V. M. Y.; George, J. M.; Rienstra, C. M. Solid-State NMR Structure of a Pathogenic Fibril of Full-Length Human α-Synuclein. *Nat. Struct. Mol. Biol.* **2016**, *23* (5), 409–415. DOI: 10.1038/nsmb.3194.

(527) Merz, G. E.; Chalkley, M. J.; Tan, S. K.; Tse, E.; Lee, J.; Prusiner, S. B.; Paras, N. A.; DeGrado, W. F.; Southworth, D. R. Stacked Binding of a PET Ligand to Alzheimer's Tau Paired Helical Filaments. *Nat. Commun.* **2023**, *14* (1), 3048. DOI: 10.1038/s41467-023-38537-y.

(528) Monistrol, J.; Beton, J. G.; Johnston, E. C.; Saibil, H. R. Stepwise Recruitment of Hsc70 by DNAJB1 Produces Ordered Arrays Primed for Bursts of Amyloid Fibre Disassembly. bioRxiv January 25, 2024, p 2024.01.25.577078. DOI: 10.1101/2024.01.25.577078 (accessed 2024-01-25).

(529) Sokratian, A.; Zhou, Y.; Xu, E.; Viverette, E.; Dillard, L.; Yuan, Y.; Li, J. Y.; Matarangas, A.; Bouvette, J.; Borgnia, M.; Bartesaghi, A.; West, A. Structural and Functional Landscape of α-Synuclein Fibril Conformations Amplified from Cerebrospinal Fluid. bioRxiv July 13, 2022, p 2022.07.13.499896. DOI: 10.1101/2022.07.13.499896 (accessed 2024-05-26).

(530) Ni, X.; McGlinchey, R. P.; Jiang, J.; Lee, J. C. Structural Insights into α-Synuclein Fibril Polymorphism: Effects of Parkinson's Disease-Related C-Terminal Truncations. *J. Mol. Biol.* **2019**, *431* (19), 3913–3919. DOI: 10.1016/j.jmb.2019.07.001.

(531) Zhao, Q.; Tao, Y.; Zhao, K.; Ma, Y.; Xu, Q.; Liu, C.; Zhang, S.; Li, D. Structural Insights of Fe3+ Induced α-Synuclein Fibrillation in Parkinson's Disease. *J. Mol. Biol.* **2023**, *435* (1), 167680. DOI: 10.1016/j.jmb.2022.167680.

(532) Dhavale, D. D.; Barclay, A. M.; Borcik, C. G.; Basore, K.; Berthold, D. A.; Gordon, I. R.; Liu, J.; Milchberg, M. H.; O'Shea, J. Y.; Rau, M. J.; Smith, Z.; Sen, S.; Summers, B.; Smith, J.; Warmuth, O. A.; Perrin, R. J.; Perlmutter, J. S.; Chen, Q.; Fitzpatrick, J. A. J.; Schwieters, C. D.; Tajkhorshid, E.; Rienstra, C. M.; Kotzbauer, P. T. Structure of Alpha-Synuclein Fibrils Derived from Human Lewy Body Dementia Tissue. *Nat. Commun.* **2024**, *15*, 2750. DOI: 10.1038/s41467-024-46832-5.

(533) Warmack, R. A.; Boyer, D. R.; Zee, C.-T.; Richards, L. S.; Sawaya, M. R.; Cascio, D.; Gonen, T.; Eisenberg, D. S.; Clarke, S. G. Structure of Amyloid-β (20-34) with Alzheimer's-Associated Isomerization at Asp23 Reveals a Distinct Protofilament Interface. *Nat. Commun.* **2019**, *10* (1), 3357. DOI: 10.1038/s41467-019-11183-z.

(534) Hallinan, G. I.; Hoq, M. R.; Ghosh, M.; Vago, F. S.; Fernandez, A.; Garringer, H. J.; Vidal, R.; Jiang, W.; Ghetti, B. Structure of Tau Filaments in Prion Protein Amyloidoses. *Acta Neuropathol. (Berl.)* **2021**, *142* (2), 227–241. DOI: 10.1007/s00401-021-02336-w.

(535) Duan, P.; Dregni, A. J.; Mammeri, N. E.; Hong, M. Structure of the Nonhelical Filament of the Alzheimer's Disease Tau Core. *Proc. Natl. Acad. Sci.* **2023**, *120* (44), e2310067120. DOI: 10.1073/pnas.2310067120.

(536) Shi, Y.; Zhang, W.; Yang, Y.; Murzin, A. G.; Falcon, B.; Kotecha, A.; van Beers, M.; Tarutani, A.; Kametani, F.; Garringer, H. J.; Vidal, R.; Hallinan, G. I.; Lashley, T.; Saito, Y.; Murayama, S.; Yoshida, M.; Tanaka, H.; Kakita, A.; Ikeuchi, T.; Robinson, A. C.; Mann, D. M. A.; Kovacs, G. G.; Revesz, T.; Ghetti, B.; Hasegawa, M.; Goedert, M.; Scheres, S. H. W. Structure-Based Classification of Tauopathies. *Nature* **2021**, *598* (7880), 359–363. DOI: 10.1038/s41586-021-03911-7.

(537) Griner, S. L.; Seidler, P.; Bowler, J.; Murray, K. A.; Yang, T. P.; Sahay, S.; Sawaya, M. R.; Cascio, D.; Rodriguez, J. A.; Philipp, S.; Sosna, J.; Glabe, C. G.; Gonen, T.; Eisenberg, D. S. Structure-Based Inhibitors of Amyloid Beta Core Suggest a Common Interface with Tau. *eLife* **2019**, *8*, e46924. DOI: 10.7554/eLife.46924.

(538) Seidler, P. M.; Boyer, D. R.; Rodriguez, J. A.; Sawaya, M. R.; Cascio, D.; Murray, K.; Gonen, T.; Eisenberg, D. S. Structure-Based Inhibitors of Tau Aggregation. *Nat. Chem.* **2018**, *10* (2), 170–176. DOI: 10.1038/nchem.2889.

(539) Boyer, D. R.; Li, B.; Sun, C.; Fan, W.; Sawaya, M. R.; Jiang, L.; Eisenberg, D. S. Structures of Fibrils Formed by α-Synuclein Hereditary Disease Mutant H50Q Reveal New Polymorphs. *Nat. Struct. Mol. Biol.* **2019**, *26* (11), 1044–1052. DOI: 10.1038/s41594-019-0322-y.

(540) Falcon, B.; Zhang, W.; Murzin, A. G.; Murshudov, G.; Garringer, H. J.; Vidal, R.; Crowther, R. A.; Ghetti, B.; Scheres, S. H. W.; Goedert, M. Structures of Filaments from Pick's Disease Reveal a Novel Tau Protein Fold. *Nature* **2018**, *561* (7721), 137–140. DOI: 10.1038/s41586-018-0454-y.

(541) Yang, Y.; Shi, Y.; Schweighauser, M.; Zhang, X.; Kotecha, A.; Murzin, A. G.; Garringer, H. J.; Cullinane, P. W.; Saito, Y.; Foroud, T.; Warner, T. T.; Hasegawa, K.; Vidal, R.; Murayama, S.; Revesz, T.; Ghetti, B.; Hasegawa, M.; Lashley, T.; Scheres, S. H. W.; Goedert, M. Structures of α-Synuclein Filaments from Human Brains with Lewy Pathology. *Nature* **2022**, *610* (7933), 791–795. DOI: 10.1038/s41586-022-05319-3.
(542) Schweighauser, M.; Shi, Y.; Tarutani, A.; Kametani, F.; Murzin, A. G.; Ghetti, B.; Matsubara, T.; Tomita, T.; Ando, T.; Hasegawa, K.; Murayama, S.; Yoshida, M.; Hasegawa, M.; Scheres, S. H. W.; Goedert, M. Structures of α-Synuclein Filaments from Multiple System Atrophy. *Nature* **2020**, *585* (7825), 464–469. DOI: 10.1038/s41586-020-2317-6.
(543) Li, X.; Zhang, S.; Liu, Z.; Tao, Y.; Xia, W.; Sun, Y.; Liu, C.; Le, W.; Sun, B.; Li, D. Subtle Change of Fibrillation Condition Leads to Substantial Alteration of Recombinant Tau Fibril Structure. *iScience* **2022**, *25* (12), 105645. DOI: 10.1016/j.isci.2022.105645.
(544) Fowler, S. L.; Behr, T. S.; Turkes, E.; Cauhy, P. M.; Foiani, M. S.; Schaler, A.; Crowley, G.; Bez, S.; Ficulle, E.; Tsefou, E.; O'Brien, D. P.; Fischer, R.; Geary, B.; Gaur, P.; Miller, C.; D'Acunzo, P.; Levy, E.; Duff, K. E.; Ryskeldi-Falcon, B. Tau Filaments Are Tethered within Brain Extracellular Vesicles in Alzheimer's Disease. bioRxiv April 30, 2023, p 2023.04.30.537820. DOI: 10.1101/2023.04.30.537820 (accessed 2024-05-27).
(545) Falcon, B.; Zhang, W.; Schweighauser, M.; Murzin, A. G.; Vidal, R.; Garringer, H. J.; Ghetti, B.; Scheres, S. H. W.; Goedert, M. Tau Filaments from Multiple Cases of Sporadic and Inherited Alzheimer's Disease Adopt a Common Fold. *Acta Neuropathol. (Berl.)* **2018**, *136* (5), 699–708. DOI: 10.1007/s00401-018-1914-z.
(546) Hojjatian, A.; Dasari, A. K. R.; Sengupta, U.; Taylor, D.; Daneshparvar, N.; Yeganeh, F. A.; Dillard, L.; Michael, B.; Griffin, R. G.; Borgnia, M. J.; Kayed, R.; Taylor, K. A.; Lim, K. H. Tau Induces Formation of α-Synuclein Filaments with Distinct Molecular Conformations. *Biochem. Biophys. Res. Commun.* **2021**, *554*, 145–150. DOI: 10.1016/j.bbrc.2021.03.091.
(547) Frieg, B.; Antonschmidt, L.; Dienemann, C.; Geraets, J. A.; Najbauer, E. E.; Matthes, D.; de Groot, B. L.; Andreas, L. B.; Becker, S.; Griesinger, C.; Schröder, G. F. The 3D Structure of Lipidic Fibrils of α-Synuclein. *Nat. Commun.* **2022**, *13* (1), 6810. DOI: 10.1038/s41467-022-34552-7.
(548) Yagi-Utsumi, M.; Itoh, S. G.; Okumura, H.; Yanagisawa, K.; Kato, K.; Nishimura, K. The Double-Layered Structure of Amyloid-β Assemblage on GM1-Containing Membranes Catalytically Promotes Fibrillization. *ACS Chem. Neurosci.* **2023**, *14* (15), 2648–2657. DOI: 10.1021/acschemneuro.3c00192.
(549) Sun, Y.; Long, H.; Xia, W.; Wang, K.; Zhang, X.; Sun, B.; Cao, Q.; Zhang, Y.; Dai, B.; Li, D.; Liu, C. The Hereditary Mutation G51D Unlocks a Distinct Fibril Strain Transmissible to Wild-Type α-Synuclein. *Nat. Commun.* **2021**, *12* (1), 1–10. DOI: 10.1038/s41467-021-26433-2.
(550) Leistner, C.; Wilkinson, M.; Burgess, A.; Lovatt, M.; Goodbody, S.; Xu, Y.; Deuchars, S.; Radford, S. E.; Ranson, N. A.; Frank, R. A. W. The In-Tissue Molecular Architecture of β-Amyloid Pathology in the Mammalian Brain. *Nat. Commun.* **2023**, *14* (1), 2833. DOI: 10.1038/s41467-023-38495-5.
(551) McGlinchey, R. P.; Ni, X.; Shadish, J. A.; Jiang, J.; Lee, J. C. The N Terminus of α-Synuclein Dictates Fibril Formation. *Proc. Natl. Acad. Sci. U. S. A.* **2021**, *118* (35), e2023487118. DOI: 10.1073/pnas.2023487118.
(552) Boyer, D. R.; Li, B.; Sun, C.; Fan, W.; Zhou, K.; Hughes, M. P.; Sawaya, M. R.; Jiang, L.; Eisenberg, D. S. The α-Synuclein Hereditary Mutation E46K Unlocks a More Stable, Pathogenic Fibril Structure. *Proc. Natl. Acad. Sci.* **2020**, *117* (7), 3592–3602. DOI: 10.1073/pnas.1917914117.
(553) Guerrero-Ferreira, R.; Taylor, N. M. I.; Arteni, A. A.; Kumari, P.; Mona, D.; Ringler, P.; Britschgi, M.; Lauer, M. E.; Makky, A.; Verasdock, J.; Riek, R.; Melki, R.; Meier, B. H.; Böckmann, A.; Bousset, L.; Stahlberg, H. Two New Polymorphic Structures of Human Full-Length Alpha-Synuclein Fibrils Solved by Cryo-Electron Microscopy. *eLife* **2019**, *8*, e48907. DOI: 10.7554/eLife.48907.
(554) Long, H.; Zheng, W.; Liu, Y.; Sun, Y.; Zhao, K.; Liu, Z.; Xia, W.; Lv, S.; Liu, Z.; Li, D.; He, K. W.; Liu, C. Wild-Type α-Synuclein Inherits the Structure and Exacerbated Neuropathology of E46K Mutant Fibril Strain by Cross-Seeding. *Proc. Natl. Acad. Sci. U. S. A.* **2021**, *118* (20), e2012435118. DOI: 10.1073/pnas.2012435118.

(555) Frieg, B.; Geraets, J. A.; Strohäker, T.; Dienemann, C.; Mavroeidi, P.; Jung, B. C.; Kim, W. S.; Lee, S.-J.; Xilouri, M.; Zweckstetter, M.; Schröder, G. F. α-Synuclein Polymorphism Determines Oligodendroglial Dysfunction. bioRxiv July 10, 2021, p 2021.07.09.451731. DOI: 10.1101/2021.07.09.451731 (accessed 2024-05-26).
(556) Limorenko, G.; Tatli, M.; Kolla, R.; Nazarov, S.; Weil, M.-T.; Schöndorf, D. C.; Geist, D.; Reinhardt, P.; Ehrnhoefer, D. E.; Stahlberg, H.; Gasparini, L.; Lashuel, H. A. Fully Co-Factor-Free ClearTau Platform Produces Seeding-Competent Tau Fibrils for Reconstructing Pathological Tau Aggregates. *Nat. Commun.* **2023**, *14* (1), 3939. DOI: 10.1038/s41467-023-39314-7.
(557) Berman, H.; Henrick, K.; Nakamura, H. Announcing the Worldwide Protein Data Bank. *Nat. Struct. Mol. Biol.* **2003**, *10* (12), 980–980. DOI: 10.1038/nsb1203-980.
(558) Berman, H. M.; Westbrook, J.; Feng, Z.; Gilliland, G.; Bhat, T. N.; Weissig, H.; Shindyalov, I. N.; Bourne, P. E. The Protein Data Bank. *Nucleic Acids Res.* **2000**, *28* (1), 235–242. DOI: 10.1093/nar/28.1.235.
(559) Chen, S. W.; Barritt, J. D.; Cascella, R.; Bigi, A.; Cecchi, C.; Banchelli, M.; Gallo, A.; Jarvis, J. A.; Chiti, F.; Dobson, C. M.; Fusco, G.; De Simone, A. Structure–Toxicity Relationship in Intermediate Fibrils from α-Synuclein Condensates. *J. Am. Chem. Soc.* **2024**, *146* (15), 10537–10549. DOI: 10.1021/jacs.3c14703.
(560) Tao, Q. Y.; Zhao, Q. Y.; Liu, C.; Li, D. Formed Alpha-Synuclein Fibrils after Incubation with Heparin for 1 Hour (Hep-Remod-1). **2023**. DOI: 10.2210/pdb8hzb/pdb.
(561) Tao, Q. Y.; Zhao, Q. Y.; Liu, C.; Li, D. Formed Alpha-Synuclein Fibrils after Incubation with Heparin for 1 Hour (Hep-Remod-2). **2023**. DOI: 10.2210/pdb8hzc/pdb.
(562) Tao, Q. Y.; Zhao, Q. Y.; Liu, C.; Li, D. Formed Alpha-Synuclein Fibrils after Incubation with Heparin for 1 Hour (Hep-Remod-3). **2023**. DOI: 10.2210/pdb8hzs/pdb.
(563) Tao, Y. Q.; Sun, Y. P.; Xia, W. C.; Zhao, Q. Y.; Liu, C.; Li, D. Heparin-Remodelled Alpha-Synuclein Fibrils. **2022**. DOI: 10.2210/pdb7v4d/pdb.
(564) Wu, K.-P.; Huang. CryoEM Structure of Human Alpha-Synuclein A53T Fibril. **2023**. DOI: 10.2210/pdb7wnz/pdb.
(565) Wu, K.-P.; Huang. CryoEM Structure of Human Alpha-Synuclein A53T Fibril Induced by Calcium Ions. **2023**. DOI: 10.2210/pdb7wo0/pdb.
